# Supplementary material for: A local-to-global emissions inventory of macroplastic pollution
Source: Nature. 2024 Sep 4;633(8028):101–8. doi: 10.1038/s41586-024-07758-6 (PMC11374682; doi:10.1038/s41586-024-07758-6)
Supplement: Supplementary file 1 — This file contains Supplementary Methods, including Supplementary Figs. 1–30, Supplementary Tables 1–40 and Supplementary References. Further Supplementary Data for this article are available from Dryad at https://doi.org/10.5061/dryad.8cz8w9gxb. [file 41586_2024_7758_MOESM1_ESM.pdf]

nature portfolio

https://doi.org/10.1038/s41586-024-07758-6

Supplementary information

A local-to-global emissions inventory of macroplastic pollution

In the format provided by the authors and unedited

Supplementary Information for

A local-to-global emissions inventory of macroplastic pollution

Published in *Nature*: https://doi.org/10.1038/s41586-024-07758-6

Joshua W. Cottom[1], Ed Cook[1], Costas A. Velis[1*]

1. School of Civil Engineering, University of Leeds, Leeds, United Kingdom, LS2 9JT

* Corresponding author: Costas A. Velis (c.velis@leeds.ac.uk)

This PDF file includes:

1. Supplementary Methods, including:

   Fig. S1 to **Fig. S30**

   Table S1 to **Table S41**

   Supplementary references

Other Supplementary information for this manuscript include the following and are available from Dryad: https://doi.org/10.5061/dryad.8cz8w9gxb

Supplementary Data 1: Data cleaning - *Steps undertaken to check data for errors, clean, harmonize and allocate to spatial vectors.*

Supplementary Data 2: System of equations - *System of equations for the material flow system*.

Supplementary Data 3: National material flow analysis outputs - *Data predicted by probabilistic MFA at municipal level are aggregated to national level. Basic statistics (mean, 5th percentile, lower quartile, median, upper quartile & 95th percentile) presented.*

Supplementary Data 4: Global, regional, and income category material flow analysis outputs - *Data predicted by probabilistic MFA at municipal level are aggregated by total (global), UN region, UN sub-region, OECD region, World Bank income category. Basic statistics (mean, 5th percentile, lower quartile, median, upper quartile & 95th percentile) presented.*

Supplementary Data 5: Municipal material flow analysis outputs - *Data predicted by probabilistic MFA at municipal level. Basic statistics (mean, 5th percentile, lower quartile, median, upper quartile & 95th percentile) presented.*

Supplementary Data 6: Material flow analysis inputs - *Input data for probabilistic MFA*

Supplementary Methods – Table of Contents

S.1 Methodology summary ___ ***4***

S.2 Scope ___ ***8***

S.3 Solid waste management data ___ ***9***

S.4 System maps ___ ***9***

S.4.1 Tributary MFA ___ **9**

S.4.2 Full MSW MFA ___ **10**

S.4.3 Plastics MFA ___ **12**

S.5 Data inputs ___ ***16***

S.6 Primary data collection, harmonisation, correction, and cleaning ___ ***20***

S.6.1 Global municipal-level solid waste management *primary input data* sources (MS1a) ___ **20**

S.6.2 National municipal-level solid waste management data sources (MS1b) ___ **21**

S.6.3 Assignment of administrative areas (MS2) ___ **22**

S.6.4 Data harmonisation (MS3a), correction (MS3b) and quality screening (MS3c) ___ **24**

S.6.4.1 Waste Wise Cities Tool (WaCT) ___ 24

S.6.4.2 Wasteaware Benchmark Cities Indicators (WABI) ___ 25

S.6.4.3 What a Waste 2.0 (WaW2.0) ___ 28

S.6.4.4 UNSD City Waste Data ___ 36

S.6.4.5 SIPSN Data ___ 43

S.6.4.6 MoHURD Data ___ 45

S.6.5 Data consolidation and deduplication ___ **46**

S.6.6 Default GADM Level selection ___ **47**

S.6.7 Data cleaning via outlier identification ___ **49**

S.7 Machine learning for prediction of primary data input variables ___ ***54***

S.7.1 Independent variables (MS4a) ___ **54**

S.7.2 Imputation of independent variables (MS4b) ___ **56**

S.7.3 Quantile regression random forest (MS5a and MS5b) ___ **56**

S.8 Secondary data collection and processing (MS6) ___ ***60***

S.8.1 Proportion of plastic that is rigid (C0a) ___ **61**

S.8.2 Informal sector recycling (P14) ___ **62**

S.8.2.1 Informal recycling sector productivity ___ 66

S.8.2.2 Proportion of plastic collected by informal recycling sector (C15) ___ 66

S.8.2.3 Proportion of plastic collected by informal recycling sector that is rigid (C21a) ___ 67

S.8.3 Rejects of rigid and flexible plastic from sorting and reprocessing by formal (C24aa C24ab) and informal (C23aa, C23ab) sectors ____ 68

S.8.3.1 Step 1: Establish baseline plastic waste collected for recycling ____ 68

S.8.3.2 Step 2 and 3: Identify empirical or assumptive data on rejects or use abductive reasoning to estimate 71

S.8.3.3 Step 3: Apply evidenced or assumed reject rates to the mass of plastic collected for recycling ____ 74

S.8.3.4 Mismanagement of rejects from sorting and reprocessing (C25aa, C25ab, C26aa, C26ab) ____ 76

S.8.4 Proportion of plastic in formal sector collection for recycling ____ 77

S.8.5 Uncollected litter (C1) ____ 77

S.8.5.1 Littering rate ____ 78

S.8.5.2 Total litter (L_T) ____ 79

S.8.5.3 Uncollected litter (C1) ____ 81

S.8.6 Proportion of plastic and rigid plastic in uncollected litter (C11 and C11a) ____ 82

S.8.7 Uncollected MSW (C2) ____ 82

S.8.8 Debris emissions from collection system (C3) ____ 82

S.8.9 Debris emissions from uncontrolled disposal of MSW (C9) ____ 84

S.8.10 Plastic (C14) and rigid plastic (C14a) in disposal debris emissions ____ 85

S.8.11 Open burning ____ 86

S.8.11.1 Open burning of uncollected waste (C10) ____ 86

S.8.11.2 Open burning of rejects from sorting and reprocessing (C27aa, C27ab, C28aa, C28 ab) ____ 87

S.8.11.3 Open burning at uncontrolled disposal sites (C8) ____ 89

S.9 Probabilistic material flow analysis (MS7) ____ 89

S.9.1 Data inputs ____ 91

S.9.1.1 Random sampling of primary input data ____ 91

S.9.1.2 Correction of primary input variable predictions by settlement typology ____ 93

S.9.1.3 Sampling of *secondary data inputs* ____ 98

S.9.2 Material flow analysis ____ 98

S.9.2.1 Spatial aggregation ____ 99

S.9.2.2 Uncertainty ____ 100

S.10 Sensitivity analysis ____ 102

S.10.1 Sensitivity of model outputs to model inputs ____ 102

S.10.2 Sensitivity of model outputs to modelling assumptions ____ 106

S.11 Conversion of emission mass to item count ____ 113

Supplementary references ____ 114

S.1 Methodology summary

We present the first of two stages in the 'Spatio-temporal quantification of plastic pollution origins and transport' model (SPOT). This first stage begins when waste is generated (created), meaning the part of the system where products and materials are 'discarded' by their users, and ends when those materials are: recycled; recovered; stored in disposal facilities; or 'emitted'. We use 'emission' to describe the flow of plastic from a state of 'containment' (control) to one where it is 'uncontained' (**Extended data Fig. 1)**. By uncontained we mean that plastic is in the 'environment', both built and natural, and is no longer subject to any form of management; it is unintentionally present. We call the point between the contained and uncontained states, the *'emission boundary'* (**Fig. S1**). For clarification, we do not consider land disposal facilities (landfills or dumpsites), to be in the environment because despite the very poor level of control in some cases (dumpsites), they are nonetheless contained, they are intended to be there. We also consider solid waste which is in sewage (wastewater) to be uncontained because despite its presence in a contained structure, it is unintentionally present, meaning that the sewers were not designed to carry it.

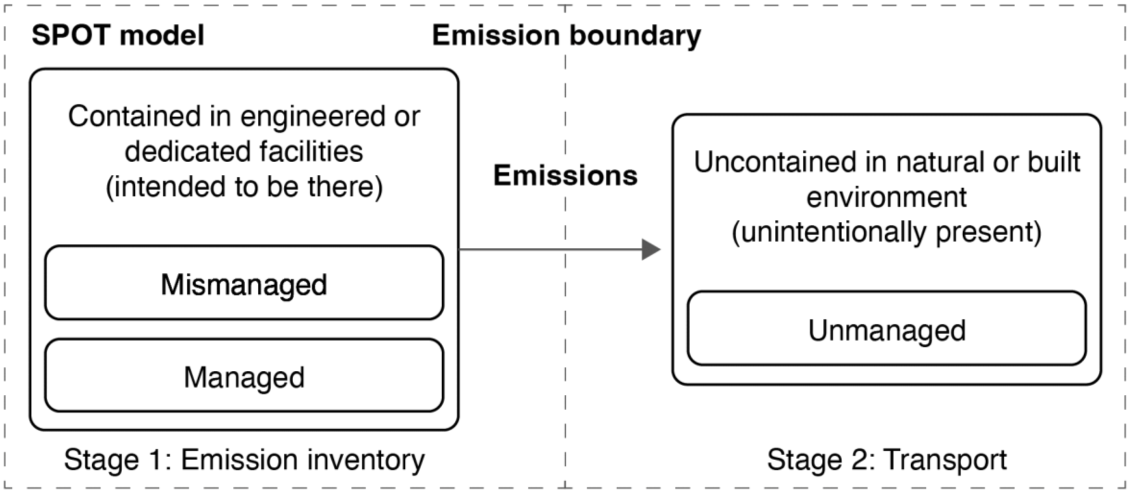

Fig. S1. The boundary between the present 'upstream' part and the next 'downstream' part of the 'Spatio-temporal quantification of plastic pollution origins and transport' model (SPOT).

Emissions of plastic fall into two categories: 1) open burning (combustion in open, uncontrolled fires); and 2) debris (physical material items, objects, and particles). Emissions through open burning (calculated as the mass partially or completely combusted) are considered a system endpoint. Emissions of debris are at risk of further transport through the terrestrial environment (unmanaged system) via the action of wind or surface water, movement which is described in the second stage of the SPOT model, and which will not be discussed further here.

Our objectives were achieved following a seven-step workflow illustrated in **Fig. S2** according to a series of methodological steps (MS).

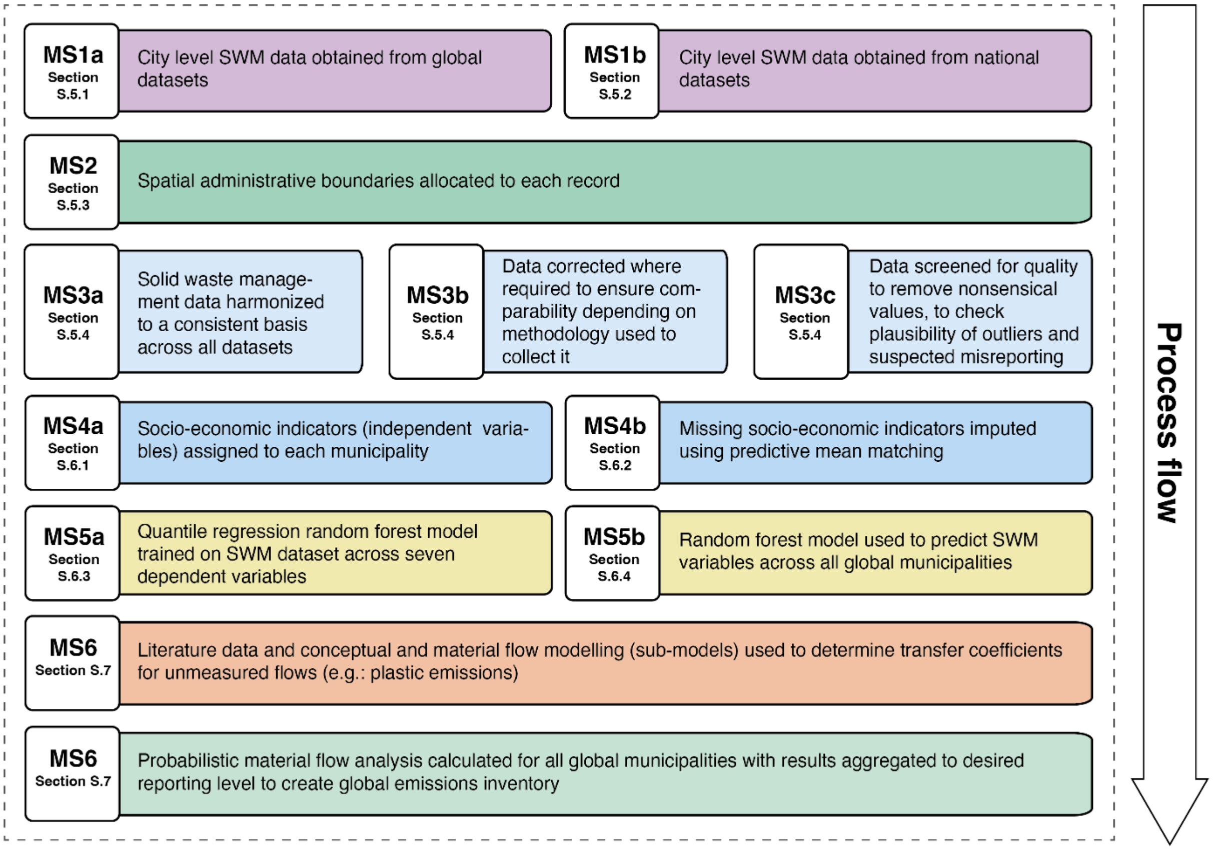

Fig. S2. Overview of steps in methodological process. Abbreviations: Solid waste management (SWM); methodological step (MS).

Municipal level solid waste management data were obtained from both global (**MS1a**, **Section S.6.1**) and national datasets (**MS1b**, **Section S.6.2**). Each record in these datasets was assigned a spatial administrative area according to the area that the data is believed to represent (**MS2**, **Section S.6.3**). Data, termed here *primary input data*, for seven solid waste management variables, termed here *primary input variables*, (**Section S.5**), were extracted from each record and harmonised to the most consistent basis possible (**MS3a**, **Section S.6.4**).

Primary input data were screened and corrected depending on the methodology used to obtain them. This ensured comparability between and within datasets (**MS3b**, **Section S.6.4**). For example, if the waste generation rate was considered to represent only collected waste, the value was corrected to obtain the overall waste generation rate (including uncollected MSW) by dividing it by the collection coverage. Following these necessary corrections, data in each record were screened to remove values that were obviously incorrect, for instance, due to user error during data input (**MS3c**, **Section S.6.4**). Variables, defined in **Section S.5**, such as formal dry recycling, incineration, and other recovery were also manually checked for plausibility based on a review of literature. For example, many cities report a 'recycling rate', but it is often unclear if material is collected by the formal authorities or by informal recycling sector participants. The plausibility review attempted to improve reliability by determining what each data point is likely to represent, and therefore provide a justification for either accepting or rejecting it as formal dry recycling. Further cleaning of the dataset was performed by manually assessing the plausibility

of outlier data points to remove those which were believed to be a result of error rather than measured variation (**Section S.6.4**).

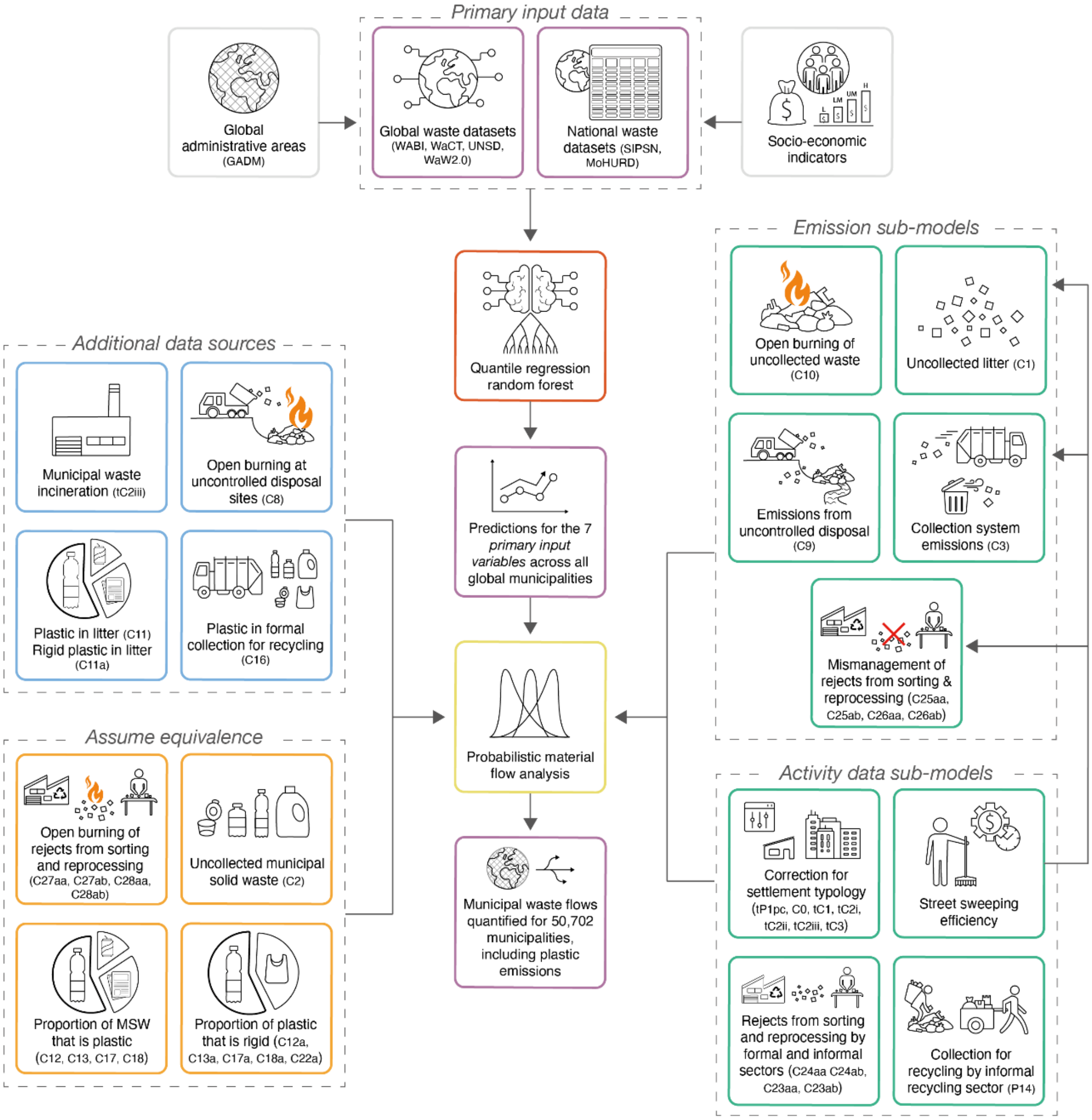

Fig. S3. Derivation of data used in Stage 1 of the Spatio-temporal Quantification of Plastic Pollution Origins and Transport model (SPOT). *Primary input data* are activity data measured at municipal level which have been quality checked, harmonised, and corrected. Blue, orange, and green hoxes represent *secondary data* which are defined as follows: *Additional data sources* are transfer coefficients that have been obtained from sources that are not directly measured at municipal level and are assumed for modelling purposes; *Assumed equivalence* indicates where, in the absence of measured data, we have used a coefficient from another part of the model which is approximately equivalent to the data that would be expected in another; *Emission sub-models* were used to approximate the flow of material from the contained to uncontained state using a combination of activity data and abductive reasoning; *Activity data sub-models* are similar to emission sub-models except that they are used to approximate mass or transfer

coefficients within the model where measured data do not exist. Other definitions can be found in **Table S2** and **Table S3**. Abbreviations: Municipal solid waste (MSW).

Data indicating economic, social, geographical, and cultural status and development (hereafter *socioeconomic indicators*) were assigned to each screened data record and to a global list[1] of administrative areas (**MS4, Section S.7.1**) that were assessed as those most likely to reflect the municipal level data (**Section S.6.6**). These consisted of both national level *socioeconomic indicators* and sub-national *socioeconomic indicators*. Missing *socioeconomic indicators* were imputed using predictive mean matching method (**Section S.7.2**).

Primary input data alongside *socioeconomic indicators* (independent variables) were used to train quantile regression random forest machine learning models for each of the seven *primary input variables* (**MS5a**, **Section S.7.3**). Ten-fold cross validation with five repeats tuned the hyperparameters of each random forest model, before their suitability was assessed against a holdout test dataset. The quantile regression random forest models were then able to be used to predict solid waste management data for all global municipalities, including associated uncertainty (**MS5b**, **Section S.7.3**).

Whereas metrics such as waste generation, waste composition, and less so, waste collection coverage, are routinely measured, there are flows in other parts of the waste management system which are rarely documented. To account for these unrecorded and in some cases, neglected material flows and phenomena, we have developed a series of sub-models which use a combination of indirectly related, measured activity data and objective reasoning to approximate SWM activity and mass (**MS6, Section S.8**). Where appropriate, we have also used data from literature which is assumed to be equivalent to data required in our model (e.g., proportion of plastic that is rigid or flexible). For example, we assume that the open burning of rejects happens at the same rate as the open burning of uncollected waste. These data, termed *secondary data inputs* in combination with the *primary data inputs* allowed detailed information of municipal solid waste (MSW) management and plastic waste to be quantified for every municipality in the world. These sub-models and datasets were used in combination with machine learning outputs to feed into probabilistic material flow analysis as illustrated in **Fig. S3**.

Primary input variables and *secondary input variables* within each administrative boundary were assigned a probability distribution from which 5,000 random samples were drawn from each as part of a probabilistic material flow analysis using Monte Carlo simulation. These samples were drawn independently for each municipality; however, the parameters that define the probability distributions were often stratified according to higher spatial aggregations (e.g. income categories) for many of the *secondary data inputs* due to data constrains. Results of municipal level material flows were aggregated to generate results at multiple spatial scales such as at national, regional, and global level (**SectionS.9.2.1**). The aggregation step included spatial dependency between municipalities and propagation of uncertainty. This provided a harmonised global macroplastic pollution emission inventory suitable for reporting and ongoing monitoring.

S.2 Scope

As with other global plastic pollution models[2-5], our global inventory model focusses on municipal solid waste, meaning the flows of waste generated from households, commerce and trade, small businesses, office buildings and institutions (schools, hospitals, government buildings) following the UN-Habitat[6] definition which excludes construction and demolition, industry and sewage treatment. We exclude textiles; electrical and electronic equipment waste; and waste material arising at sea. We model at municipal scale because that is the resolution at which waste is managed and which waste data are measured. Quantification of municipal waste flows begin at the point of waste generation. We do not consider upstream stages such as production or consumption of goods because our method is focused on the waste management phase.

'Embedded plastics', for example those as part of assemblies of items or appended or adhered to non-plastic items are assumed to be included in our model, despite the uncertainty of their inclusion in measured source data (waste characterisation methodology).

Plastics waste exports from high income countries (HICs) have been justifiably highlighted as a potential contributor to plastic pollution in the Global South where rejects are at higher risk of being mismanaged[7]. However, in recent years the global secondary materials markets have changed substantially and we assert that they have become a distraction from more prevalent emissions sources[8].

We deliberately omit plastic waste exports from our analysis for two reasons: (1) Attributing plastic waste exports to a municipal source and recipient is a complex task and the data to carry out such analysis are not available; and (2) Since the near complete ban on imported plastic waste by China in 2018[9], more recent changes to the Basel Convention[10], and to EU Regulation 1013/2006[11], plastic waste exports from the top OECD exporting countries and regions to the Global South (non-OECD and Turkey combined) have plummeted from nearly 5.4 $Mt \cdot y^{-1}$ in 2017 (prior to the effects of the Chinese import ban[12]) to less than 1.7 $Mt \cdot y^{-1}$ in 2022[13]. Based on our model results which show that the mean plastic waste emitted from recycling system rejects across all the countries in the Global South (approximately 1 Mt), approximately 1.6% of the 63 Mt collected for recycling is emitted into the environment. From this we can approximate an emission burden of 0.03 $Mt \cdot y^{-1}$ from HIC exports; virtually all of which (95%) can be attributed to eight countries: Japan, Netherlands, United States, Germany, Belgium, United Kingdom, Australia, and Italy[13,14]. Although we acknowledge that these emissions may affect the per capita burden in a few HICs, we argue that the overall contribution is negligible in the context of 52.1 $Mt \cdot y^{-1}$ plastic waste emissions worldwide. Therefore, we conclude that the very large and complex task of including exported plastic waste in our model framework is unjustified as the proportion of emissions is comparatively very small.

The concept of 'mismanaged waste' is not used as the basis for modelling here. Instead, we describe the complex flows of waste through the technosphere and the emission of waste plastic from five separate sources into the unmanaged system (**Fig. S1**). Each source considers the type of emission (with open burning of plastic distinct from particles of solid waste, termed here 'debris'), as well as the format of the plastic (rigid versus flexible). Microplastics are omitted from our analysis which focusses on the macroplastic fraction, items and particles >5 mm across any spatial dimension[15].

S.3 Solid waste management data

Solid waste management data vary substantially in both availability and reliability[16]. In the Global South, where waste is seldom weighed, waste generation is often estimated by counting trucks entering the disposal sites and applying assumptions[17]. Aside from the inaccuracy of this method, it does not account for the many other pathways through which waste flows. For example, waste which has not been collected is often burned, buried, dumped into waterways, or deposited on the surface of the land[5]. The informal recycling sector also collect valuable materials, sometimes before they leave the premises of the household or business in which they were generated[18]. The reliability of waste composition data is also highly variable, particularly in parts of the Global South[19]. There is even evidence that some well-funded high income country waste characterisation studies are carried out without consideration of statistical representation of samples[20]. Collection coverage is often estimated because it is not straightforward to measure that which has not been managed. The number of households and businesses which do not receive a service can be used as a proxy. Speculatively, in cases where waste management services are minimal, the resources to make such estimations may also be lacking. Moreover, there may be political interest in under- or over-reporting statistics. For instance in India, official data include only a small proportion of MSW generated, and high collection coverage (95.4%) throughout the country[21]. In practice the data exclude rural areas and many towns and villages, meaning waste generation is underestimated by a factor of between 4 and 7[(21-23)].

As we highlight in this study, measurement of waste generation and management takes place at municipal or sub-municipal level, and in the Global South, it is focused primarily on urban areas. National waste management datasets are created by aggregating these municipal measurements[24]. However, because there are often insufficient resources to keep records in all municipalities, many are interpolated for the purposes of national scale aggregation[17]. Whereas all other plastic pollution models use nationally aggregated data, which are either distributed (allocated) to a finer resolution (top-down approach), our model uses municipal scale data which are scaled upwards (bottom-up approach). By doing so, we aim to represent observable local scale variability between municipal waste management practices. As interventions to tackle plastic pollution often require localised intelligence, our model can identify locations where plastic pollution is most problematic and enable decisionmakers to target their scarce resources.

S.4 System maps

Flows of waste in 50,702 municipalities were mapped according to three distinct system maps (**Fig. S4-Fig. S8**) using material flow analysis (MFA)[25] as described in **Sections S.4.1**, **S.4.2**, and **S.4.3**.

S.4.1 Tributary MFA

The first system map is a simplistic MFA, known hereafter as the '*Tributary MFA'* (**Fig. S4**) because it feeds the subsequent MFA where the results are calculated. This aimed to quantify the major flows of MSW managed by formal systems in every municipality worldwide, using data that is both directly measured by local authorities and commonly reported. For example, municipal waste generation rate (tP1), collection coverage (tC1), controlled disposal (tC3) and

the proportions sent to various treatment and recovery facilities (tC2). Nomenclature is listed in **Supplementary Data 2**.

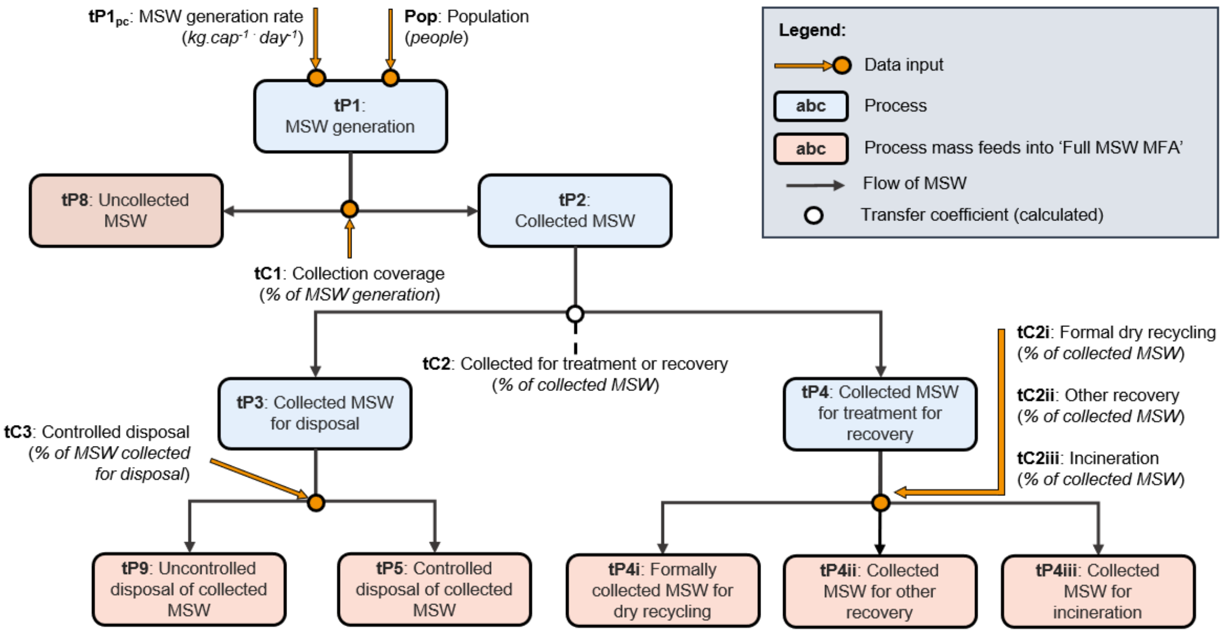

Fig. S4. Tributary material flow analysis (MFA) system map showing the major flows of municipal solid waste (MSW) formally managed in a municipality. Orange arrows represent data input points used to populate the processes and flows. Masses calculated for the pink process boxes feed through into the *Full MSW MFA* (**Fig. S5**).

The population of each municipality was multiplied by the MSW generation rate ($kg \cdot cap^{-1} \cdot y^{-1}$) to arrive at an estimate of waste generation (tP1). The collection coverage (tC1) dictates how much waste is collected (tP2), and therefore enters the waste management system compared to the amount that remains uncollected (tP8) and is assumed to be self-managed by residents and other waste generators. Here, 'self-management' of waste includes ad-hoc activities carried out by individuals (households/workplaces) in order to manage discarded materials (waste) in the absence of formal managed service provision by a community, municipal or private entity. Activities include open burning; burying; scattering (dumping|) on land; and dumping into waterways and coastal waters. The amount of collected waste sent for incineration (tP4iii), dry recycling (tP4i), and other recovery facilities (tP4ii) were summed to calculate the amount of waste going to treatment or recovery (tP4), whereas the remaining collected waste was transferred to land disposal (tP3) where it was further distributed by either controlled (tP5) or uncontrolled (tP9) disposal (defined in **Table S2**, **Section S.5**).

S.4.2 Full MSW MFA

Whereas the *Tributary MFA* (**Section S.4.1**) provides a simplistic overview of the major MSW flows within a municipality, it is not detailed enough to quantify all MSW flows and therefore describe all plastic emission sources.

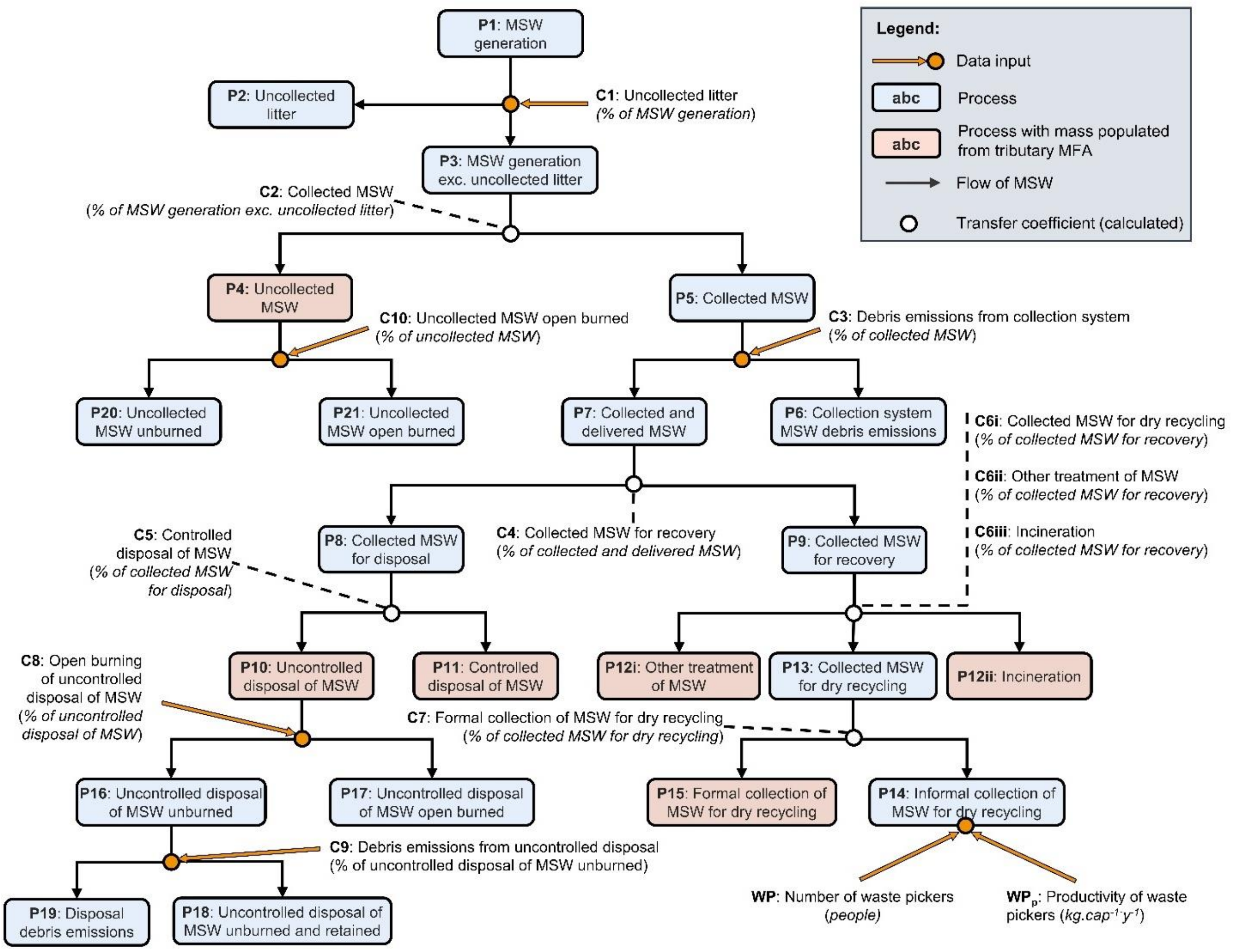

Fig. S5. Full municipal solid waste (MSW) material flow analysis (MFA) system map (*Full MSW MFA*). Orange arrows represent data input points used to populate the processes and flows of the MFA. The masses associated with the pink process boxes are populated from those in the *Tributary MFA* (**Fig. S4**).

Flows such as those which represent the amount of material collected by the informal recycling sector (IRS) (i.e., waste pickers) can be substantial across municipalities in the Global South[26], but are often unreported because they occur outside of the formal waste management system[27]. Emissions of solid waste into the environment are also largely unreported because measuring them is challenging and most municipalities are not compelled or motivated to do so. For example, emissions are often spatially and temporally dispersed, can be orders of magnitude lower in mass than collected flows, and frequently depend on human behaviour and practices which are challenging to quantify (e.g., open burning). Nonetheless, quantification of flows that are neglected from formal reporting are required to estimate plastic emissions into the environment. The '*Full MSW MFA'*, incorporates these neglected flows to provide a more detailed map of MSW flows in each municipality (**Fig. S5**).

The *Full MSW MFA* uses the masses calculated in the *Tributary MFA* as inputs, as shown by the pink process boxes. Assignment of mass in this manner ensured that these processes match as closely as possible to the masses measured by municipalities. The remaining flows and processes

were calculated from these using transfer coefficients as described in **Section S.9**. A full system of equations describing the MFA calculations is presented separately in **Supplementary Data 2**.

S.4.3 Plastics MFA

The final system map is the '*Plastics MFA*', shown in **Fig. S6**, **Fig. S7** and **Fig. S8**. This MFA takes system MSW endpoints from the *Full MSW MFA*, converts them to plastic material flows, and then disaggregates them by rigid and flexible format according to the definitions proposed by Charles and Kimman[28]. Plastic flows are calculated at these system endpoints rather than for the *Full MSW MFA* to incorporate the plastic compositions which vary at different parts of the solid waste management system. For example, the proportion and composition of plastic in litter is likely to be different to the proportion and composition of plastic generated at the household level. Alternatively, if plastic flows were mapped throughout all the system, transfer coefficients on aspects such as the proportion of plastics sent to composting or incineration would need to be sourced. Data to evidence these parts of the system would be challenging to obtain and are largely irrelevant to the overall analysis. However, given the amount of plastic in MSW (C0) is commonly measured, we considered it advantageous to obtain these data to calculate plastic waste generation. Additionally, it provided a reliable proxy for plastic compositions at system ends points in situations where no other data were available.

The plastic sorting processes carried out by the formal and informal recycling sectors were disaggregated into both rigid and flexible plastic formats before assigning transfer coefficients on aspects such as the reject (loss) rate (**Fig. S7**: C23aa, C23ab, C24aa, C24ab). Here we define these reject rates as the amount of plastic collected for recycling that is subsequently discarded during sorting operations at the sorting or reprocessing stages. These transfer coefficients were derived via a sub-model described in **Section S.8.3** which considers recyclability and value of plastics to approximate the probability of material being positively selected for reprocessing.

There are 20 points in the MFA system where plastic is emitted into the environment (uncontrolled system), though these can be simplified to five generic 'emission sources' as shown in **Table S1**.

Table S1. System emissions: generic sources and specific components.

**Generic emission source**		**Generic system emission component**		**Material format and mode of emission**			
				**Debris**		**Burned**	
**ID#**	**Description**			**Rigid**	**Flex**	**Rigid**	**Flex**
GES-01	Uncollected waste	Uncollected plastic		P20a		P21a	
				P20aa	P20ab	P21aa	P21ab
GES-02	Litter	Uncollected plastic litter		P2a		-	
				P2aa	P2ab	-	-
GES-03	Collection system	Collection system plastic emissions		P6a		-	
				P6aa	P6ab	-	-
GES-04	Disposal system	Uncontrolled disposal of plastic		P19a		P17a	
				P19aa	P19ab	P17aa	P17ab
GES-05	Sorting and reprocessing	Mismanaged sorting rejects	Formal	P33aa	P33ab	P32aa	P32ab
			Informal	P31aa	P31ab	P30aa	P31ab

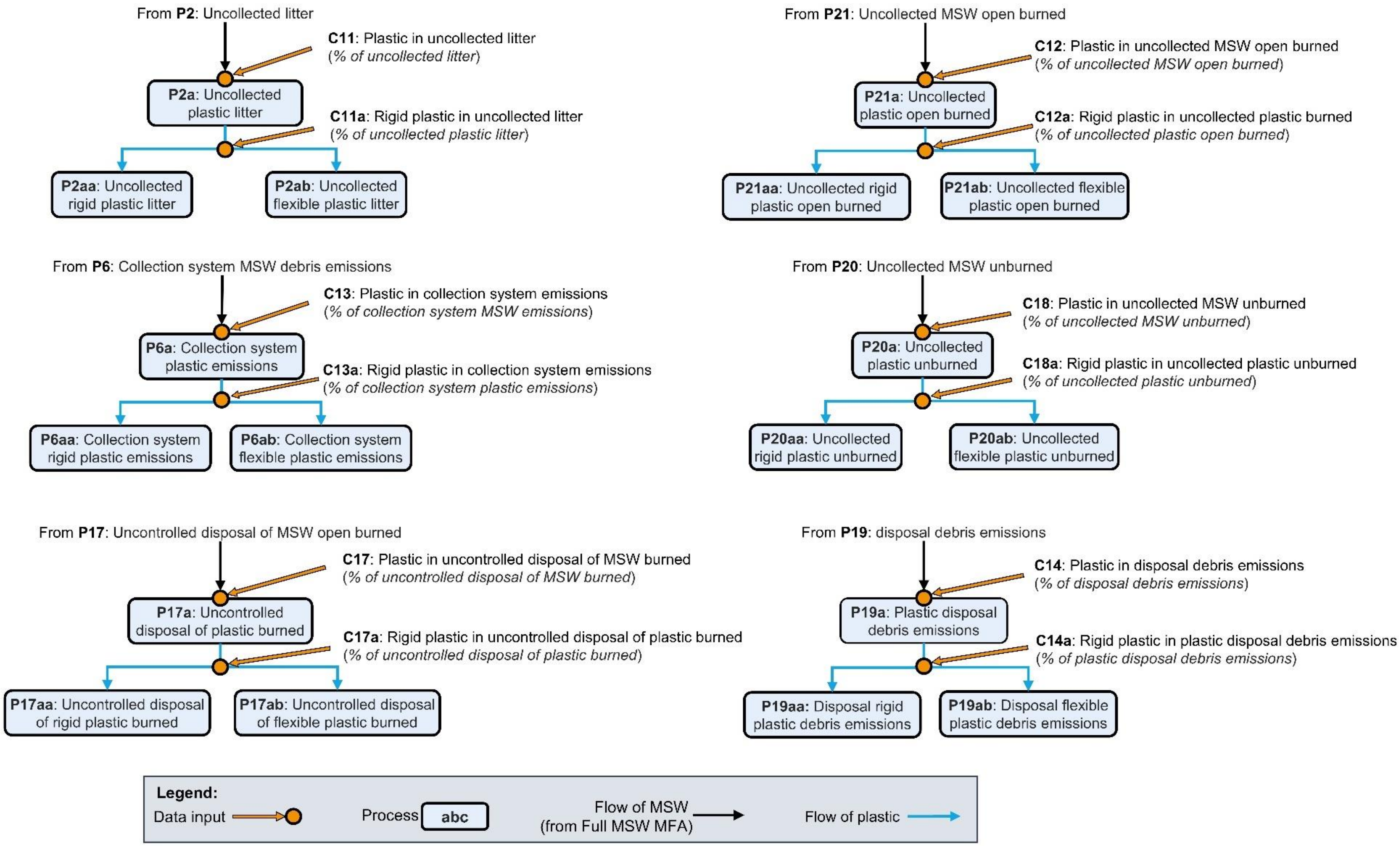

Fig. S6. Plastics material flow analysis (MFA) system map for uncollected litter, uncollected waste, collection system emissions, uncontrolled disposal, and disposal debris emissions. The *Plastics MFA* continues in **Fig. S7** and **Fig. S8** for informal and formal recycling flows respectively.

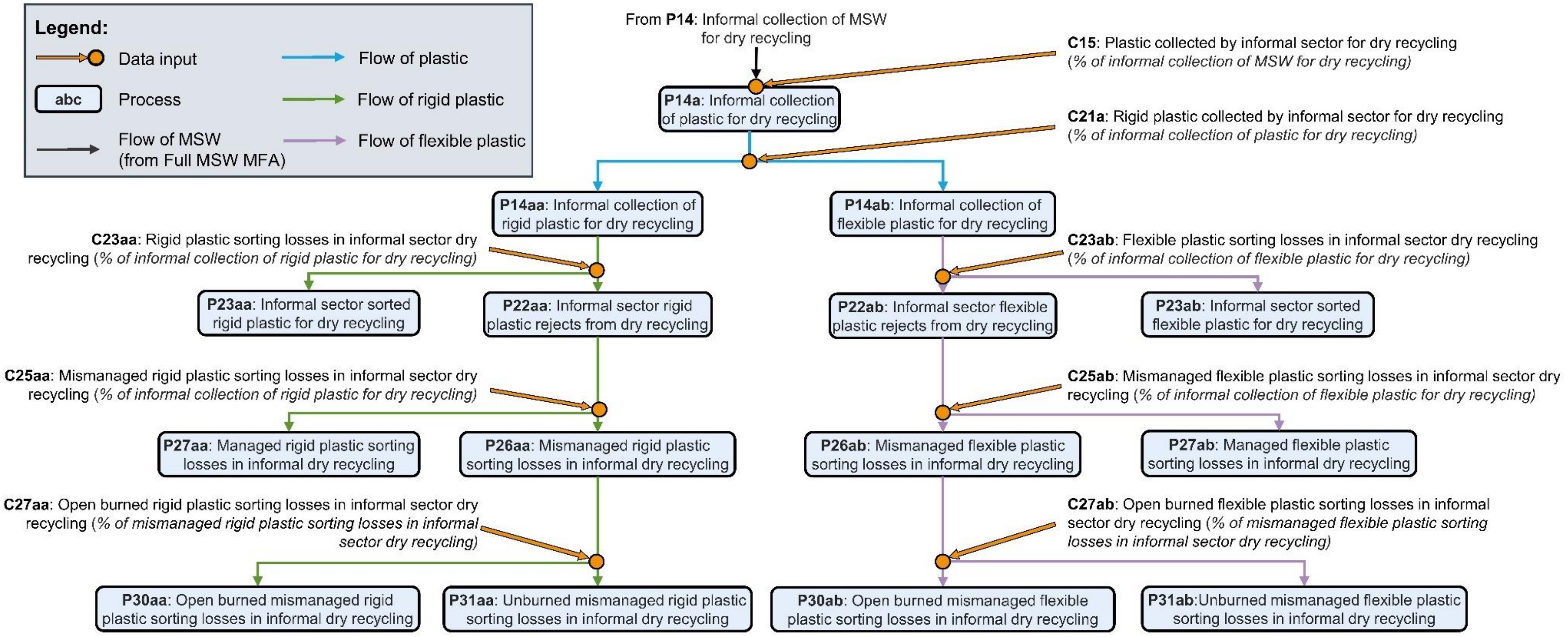

Fig. S7. Plastics material flow analysis (MFA) system map for sorting by the informal recycling sector (IRS).

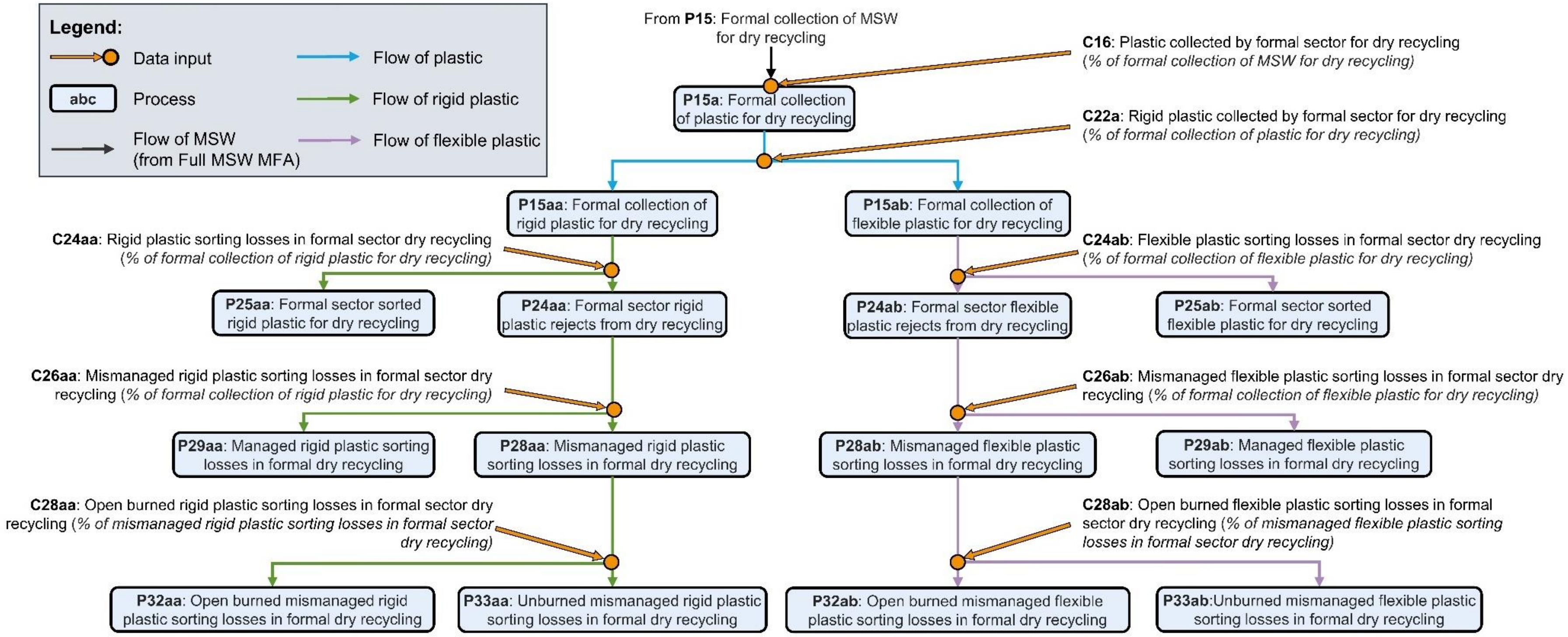

Fig. S8. Plastics material flow analysis (MFA) system map for sorting by the formal recycling sector.

S.5 Data inputs

Data on solid waste management was collected at a municipal level using existing published data sources, as discussed in **Sections S.6.1** and **S.6.2**. This data was required to populate the MFAs from **Section S.4** and can be divided into two main categories:

Primary data inputs	Data on solid waste management that is widely measured by municipalities and of which large amounts of data exist.
Secondary data inputs	Data on solid waste management that are infrequently measured by municipalities, and for which limited data exists yet is critical to include in plastic pollution quantification.

The *Tributary MFA* was populated solely by the *primary data inputs*, as shown in **Table S2**. Further description on the sources and methods use to collect, harmonise, and clean the data is discussed in **Section S.6**.

Table S2. *Primary data inputs* used to populate the *Tributary MFA,* including source of data and scale at which they are derived.

ID	Name	Unit	Description	Scale	Source
Pop	Population	People	Number of people living within a specified boundary	Municipal	29
$tP1_{pc}$	MSW generation rate	$kg \cdot cap^{-1} \cdot d^{-1}$	Waste generated from households, commerce and trade, small businesses, office buildings and institutions (schools, hospitals, government buildings). It also includes bulky waste (e.g., white goods, old furniture, mattresses) and waste from selected municipal services, e.g., waste from park and garden maintenance, waste from street cleaning services (street sweepings, the content of litter containers, market cleansing waste), if managed as waste.	Municipal	30-35
tC1	Collection coverage	% wt. of MSW generated	Waste that has been collected with the intention or purported intention to transport it to a place for treatment or disposal. Waste can be collected by public authorities, commercial entities.	Municipal	
tC2i	Formal collection of MSW for dry recycling	% wt. of formally collected MSW	Waste collection by the formal sector with the intention, or purported intention of delivering it to a facility where it can be sorted and or reprocessed to recover material value.	Municipal	
tC2ii	Formal collection of MSW for other recovery	% wt. of formally collected MSW	Waste collection by the formal sector with the intention, or purported intention of delivering it to a facility where it can be treated or processed through composting, anaerobic digestion, or processes which recover energy or materials other than incineration or recycling.	Municipal	
tC2iii	Formal collection of MSW for incineration	% wt. of formally collected MSW	Waste collection with the intention, or purported intention of delivering it to a combustion facility where it will be processed with or without energy recovery. This definition also includes solid recovered fuel production, regardless of where the combustion takes place.	Municipal	
tC3	Controlled disposal of MSW	% wt. of formally collected MSW for disposal	A facility to which waste is transported for the purposes of material or energetic recovery or disposal. Controlled facilities are operated under basic, improved, or full control according to the Ladder of waste management facilities' control level defined in the UN-Habitat[6] Waste Wise Cities Tool.	Municipal	
C0	Plastic in MSW*	% wt. of MSW generated	Proportion (wt. as received.) of plastic material as proportion of total waste.	Municipal	

C0a	Rigid plastic in MSW*	% wt. of MSW plastic generated	Proportion (wt. as received.) of rigid format plastic material as proportion of all plastic.	Income category

* These inputs were not used in *Tributary MFA* but are still grouped as a *Primary data input* as they widely measured data points and collected from the same datasets as above.

The mass calculated for each process in the *Tributary MFA* was assigned to the *Full MSW MFA* and *Plastics MFA*, with the *secondary data inputs* (**Table S3**) used to populate the remaining flows and processes. Sourcing of the inputs relied on a combination of assigning any existing data to archetypes (e.g., country income categories), modelling based on available data and known relationships, or as a last resort, assumptions. Details of the sources used, and analysis is discussed further in **Section S.7**.

Table S3. *Secondary data inputs* used to populate *Full MSW MFA* and *Plastics MFA*, including source of data and scale at which they are derived.

ID	Name	Unit	Description	Scale	Source
WP	Number of informal waste pickers	People	The number of people engaged in waste collection activities (for the purposes of waste recovery or as a service) who do not operate under contracts with formal authorities or are unlicensed to carry out such activities.	Income category	Modelled based on available data (**Section 0S.7**)
WP_p	Productivity of informal waste pickers	$tonnes \cdot cap^{-1} \cdot y^{-1}$	The average amount of waste that is collected by informal waste pickers.	Global	Modelled based on available data (**Section S.8.2.1**)
C1	Uncollected litter	% of MSW generation	Waste generated on-the-go (in the public domain) that is discarded directly by humans into the environment without having previously been concentrated or containerised and which is not collected and managed.	Income category and rurality	Modelled based on available data (**Section S.8.5**)
C3	Debris emissions from collection system	% of collected MSW	Waste that has been concentrated and presented for collection or which has been collected and which subsequently escapes from containers or vehicles prior to being deposited at a transfer, storage, treatment, or disposal facility.	National / Income category and rurality	Modelled based on quality of collection and street sweeping efficiency (**Section S.8.8**)
C8	Open burning of uncontrolled disposal	% of uncontrolled disposal of MSW	Waste that has been deposited in an uncontrolled disposal facility and which is subsequently combusted in an open uncontrolled fire, accidentally, intentionally, or spontaneously.	Global	Based on [6,36] (**Section S.8.11.3**)
C9	Debris emissions from uncontrolled disposal of MSW	% of uncontrolled disposal of MSW unburned	Waste that has been deposited in an uncontrolled disposal facility which has not been combusted in open uncontrolled fires and which is subsequently emitted from that uncontrolled facility into the environment through the action of wind, surface water or gravity.	Global but linked to municipal level mass disposed	Modelled based on available data (**Section S.8.9**)
C10	Uncollected MSW openly burned	% of uncollected MSW	Material that has not been collected and which is subsequently combusted in an open uncontrolled fire, accidentally, intentionally, or spontaneously.	Income category and rurality	Modelled based on available data (**Section S.8.11.1**)
C11	Plastic in uncollected litter	% of uncollected litter	The proportion of waste material which is characterised as plastic.	Global	[37] (**Section S.8.6**)
C12	Plastic in uncollected MSW openly burned	% of uncollected MSW openly burned	The proportion of uncollected waste material that is characterised as plastic, and which is openly burned.	Municipal	Assumed same as plastic in MSW (C0)

ID	Name	Unit	Description	Scale	Source
C13	Plastic in collection system debris emissions	% of collection system debris emissions	The proportion of collection system debris emissions that is plastic.	Municipal	Assumed same as plastic in MSW (C0) (**Section S.8.8**)
C14	Plastic in disposal debris emissions	% of disposal debris emissions	The proportion of debris emissions from uncontrolled disposal of MSW that is characterised as plastic.	Global	Assumed (**Section S.8.10**)
C15	Plastic collected by informal recycling sector	% of informal sector collection of MSW for dry recycling	The proportion of waste collected by informal waste pickers that is characterised as plastic.	Income category	Modelled based on available data (**Section S.8.2**)
C16	Plastic collected by formal recycling sector	% of formal sector collection of MSW for dry recycling	The proportion of waste collected for recycling by the formal sector that is characterised as plastic.	Global	[38] (**Section S.8.4**)
C17	Plastic in uncontrolled disposal of MSW openly burned	% of uncontrolled disposal of MSW openly burned	The proportion of waste material that is deposited in uncontrolled disposal sites and openly burned and which is characterised as plastic.	Municipal	Assumed same as plastic in MSW (C0)
C18	Plastic in uncollected MSW unburned	% of uncollected MSW unburned	The proportion of waste material that has not been collected and which is dumped as debris into the environment, and which is characterised as plastic.	Municipal	
C11a	Rigid plastic in uncollected litter	% of uncollected plastic litter	The proportion of plastic waste in uncollected litter which we describe as 'rigid', according to the definitions proposed by Charles and Kimman[28].	Global	[37] (**Section S.8.6**)
C12a	Rigid plastic in uncollected plastic openly burned	% of uncollected plastic openly burned	The proportion of uncollected rigid plastic waste that is burned in open uncontrolled fires.	Income category	Assumed same as rigid plastic in MSW (C0a) (**Section S.8.1**)
C13a	Rigid plastic in collection system debris emissions	% of collection system plastic debris emissions	The proportion of collection system plastic debris emissions that is rigid.	Income category	
C14a	Disposal system rigid plastic debris emissions	% of disposal system plastic debris emissions	The proportion of disposal system plastic debris emissions that is rigid.	Global	Assumed (**Section S.8.10**)
C17a	Rigid plastic in uncontrolled disposal of plastic openly burned	% of uncontrolled disposal of plastic openly burned	The proportion of plastic waste in the disposal system which is burned in open uncontrolled fires and which is rigid.	Income category	Assumed same as rigid plastic in MSW (C0a) (**Section S.8.1**)
C18a	Rigid plastic in uncollected plastic unburned	% of uncollected plastic unburned	The proportion of uncollected plastic waste that is rigid.	Income category	
C21a	Rigid plastic in informal collection for recycling	% of informal sector collection of plastic for dry recycling	The proportion of plastic waste collected by the informal recycling that is rigid.	Global	Modelled based on available data (**Section S.8.2.3**)
C22a	Rigid plastic in formal collection for recycling	% of formal sector collection of plastic for dry recycling	The proportion of plastic waste collected by the formal recycling that is rigid.	Income category	Assumed same as rigid plastic in MSW (C0a) (**Section S.8.1**)
C23aa	Informal sector sorting rejects of rigid plastic	% of rigid plastic collected by informal sector for dry recycling	The proportion of informal sector rigid plastics, collected for recycling, which is rejected at the sorting or reprocessing stage.	Global	Modelled based on available data (**Section S.8.3**)

ID	Name	Unit	Description	Scale	Source
C23ab	Informal sector sorting rejects of flexible plastic	% of flexible plastic collected by informal sector for dry recycling	The proportion of informal sector flexible plastics, collected for recycling, which is rejected at the sorting or reprocessing stage.	Global	
C24aa	Formal sector sorting rejects of rigid plastic	% of rigid plastic collected by formal sector for dry recycling	The proportion of formal sector rigid plastics, collected for recycling, which is rejected at the sorting or reprocessing stage.	Global	
C24ab	Formal sector sorting rejects of flexible plastic	% of flexible plastic collected by formal sector for dry recycling	The proportion of formal sector flexible plastics, collected for recycling, which is rejected at the sorting or reprocessing stage.	Global	
C25aa	Unmanaged rigid plastic sorting rejects by informal sector	% of informal sector rigid plastic sorting rejects	The proportion of sorting rejects from rigid plastic waste collected for recycling by the informal sector, which is unmanaged, meaning it is not collected and transferred to a facility (controlled or otherwise).	Municipal	Modelled based on collected coverage and street sweepings (**Section S.8.3**)
C25ab	Unmanaged flexible plastic sorting rejects by informal sector	% of informal sector flexible plastic sorting rejects	The proportion of sorting rejects from flexible plastic waste collected for recycling by the informal sector, which is unmanaged, meaning it is not collected and transferred to a facility (controlled or otherwise).	Municipal	
C26aa	Unmanaged rigid plastic sorting rejects by formal sector	% of formal sector rigid plastic sorting rejects	The proportion of sorting rejects from rigid plastic waste collected for recycling by the formal sector, which is unmanaged, meaning it is not collected and transferred to a facility (controlled or otherwise).	Municipal	
C26ab	Unmanaged flexible plastic sorting rejects by formal sector	% of formal sector flexible plastic sorting rejects	The proportion of sorting rejects from flexible plastic waste collected for recycling by the formal sector, which is unmanaged, meaning it is not collected and transferred to a facility (controlled or otherwise).	Municipal	
C27aa	Open burning of unmanaged rigid plastic sorting rejects by informal sector	% of informal sector unmanaged rigid plastic sorting rejects	The proportion of unmanaged rigid plastic rejected during sorting and reprocessing by the informal recycling sector that is subsequently burned in open uncontrolled fires.	Income category and rurality	Assumed same as C10
C27ab	Open burning of unmanaged flexible plastic sorting rejects by informal sector	% of informal sector unmanaged flexible plastic sorting rejects	The proportion of unmanaged flexible plastic rejected during sorting and reprocessing by the informal recycling sector that is subsequently burned in open uncontrolled fires.	Income category and rurality	
C28aa	Open burning of unmanaged rigid plastic sorting rejects by formal sector	% of formal sector unmanaged rigid plastic sorting rejects	The proportion of unmanaged rigid plastic rejected during sorting and reprocessing by the formal recycling sector that is subsequently burned in open uncontrolled fires.	Income category and rurality	
C28ab	Open burning of unmanaged flexible plastic sorting rejects by formal sector	% of formal sector unmanaged flexible plastic sorting rejects	The proportion of unmanaged flexible plastic rejected during sorting and reprocessing by the formal recycling sector that is subsequently burned in open uncontrolled fires.	Income category and rurality	

S.6 Primary data collection, harmonisation, correction, and cleaning

S.6.1 Global municipal-level solid waste management *primary input data* sources (MS1a)

Solid waste generation and management data for municipalities across the world were obtained from four sources[30-33] as shown in **Table S4**.

Table S4. Global municipal-level solid waste management *primary input data* sources.

Quality assurance hierarchy	Primary input data source	Data year(s)	Scale	Number of locations (records)	Methodology and quality assurance
1	Waste Wise Cities Tool (WaCT)[30]	2019 - 2022	Global	38*	Primary data collection as described in the WaCT user manual[6]. Quality assurance is checked based on data coherence and comparison against other datasets (e.g. What a Waste 2.0 data[31]).
2	Wasteaware Cities Benchmark Indicators (WABI)[32]	2007 - 2018	Global	71	Secondary data used with some quality assurance checks by waste management experts[39]
3	What a Waste 2.0 (WaW2.0) cities data[31]	2018	Global	368	Combination of secondary data collected by literature reviews and questionnaire. Data quality assessment unclear but believed to be via data coherence calculations (e.g. percentages sum to 100).
4	United Nations Statistics Division (UNSD) Cities Waste data[33]	1989 - 2019	Global	237**	Data submitted by cities via a questionnaire provided by UNSD[40]. Data quality assessed via data coherence calculations (e.g. percentages sum to 100).

* As of April 2023; ** Latest available year

Data for 714 municipalities in 180 countries were extracted from the global datasets, although this number reduced to 553 municipalities after removal of duplicate locations or during the screening and cleaning stages (**Section S.6.4**).

All global data sources had variable data years, dating back to 1989 in the case of the UNSD waste data. Data older than 15 years (2006 at time of analysis) was excluded as it was assumed that waste management has changed substantially since then, thereby reducing its relevance. This exclusion had only limited impact as most locations had data for more recent years. Following the data cleaning phase, the mean and median year of the *primary data inputs* was 2015. With further efforts in data collection occurring at a rapid pace in recent years, particularly as part of the UN-Habitat[6] Waste Wise Cities Tool official data collection effort associated with the quantification and monitoring of the SDG target 11.6.1 of environmentally sound management of solid waste in cities, it is envisaged that more up to date data can be harnessed in the future. However, at present we maximised data quantity and quality over data year relevance.

Each global data source had its own methodology for data collection (**Table S4**), which had to be understood so that data could be harmonised and corrected where necessary (**Section S.6.4**). Quality assurance measures implemented by the data source administrators and investigators were also assessed. This enabled us to prioritise records which were duplicated across multiple datasets and to inform the data-cleaning phase. The WaCT data were assumed to have the highest quality because they were recently obtained using a standardised primary sampling

method[6] and then quality checked for coherence by experts. The WABI data were assumed the next highest quality because it was checked by waste management experts alongside wider additional checks[39,41]. The quality assurance for WaW2.0 city data and UNSD city waste data is believed to mainly be via data coherence calculations only, for example, where percentages are checked to sum to 100%. Based on our own assessment of the data quality, we assigned a higher priority to the WaW2.0 data compared to UNSD city waste data.

S.6.2 National municipal-level solid waste management data sources (MS1b)

In addition to the four global-scale data sources (WaW2.0, WaCT, WABI and UNSD), municipal level data were extracted from two national databases as shown in **Table S5**. Specifically, the national waste databases of Indonesia[35] and China[34] were included due to previous works[2,3] highlighting these countries as key contributors to plastic pollution and only limited municipal-level data being available for these from the four global datasets.

Table S5. National municipal-level solid waste management *primary input data* sources.

Primary input data source	Data year(s)	Scale	Number of locations (records)	Methodology and quality assurance
**Sistem Informasi Pengelolaan Sampah Nasional (SIPSN)**[35]	2020	Indonesia	502 (10 records extracted)	Data are uploaded by representatives from municipalities. Data quality assurance is not reported.
**Ministry of Housing and Urban-Rural Development (MoHURD)**[34]	2019	China	676* (47 records extracted)	Data provenance is unclear, though it is assumed that records are submitted to the Ministry by the municipalities. Data quality assurance is not reported.

* Sub-Provincial level

Data record extraction from the national databases of China and Indonesia was limited to 2% of the total national records to avoid overrepresentation and potential biasing in the subsequent machine learning steps (**Section S.7**). Records were chosen at random and filtered according to the following conditions:

- Only data for urban areas was selected (as discussed in **Section S.7.1**).
- Only data with a high level of certainty with regards to administrative area matching were selected (≥60% similarity for China municipalities or score of 1 for Indonesian municipalities, as discussed in **Section S.6.3**)

The motivation behind the selection of only urban data points was to ensure compatibility with the four global datasets, which predominantly included data for urban areas, whereas the other filter was applied to ensure data quality.

The most recent published year was chosen for each of the countries at the time of analysis, giving data from 2020 for Indonesia and 2019 for China. Data quality assurance and provenance for the two datasets was not clearly stated by either. It is assumed that data are uploaded directly by municipal authorities, and assessment of the content infers that only limited quality assurance is carried out in each case. We assessed each of these datasets in full, flagging anomalies and suspected data entry errors; only including data that appeared to be entered correctly.

S.6.3 Assignment of administrative areas (MS2)

The Global Administrative Areas (GADM) dataset V3.6[1] is a geographical information systems (GIS) database including 386,733 polygons that represent up to five administrative area levels within each country.

The number of boundaries used by national administrations to organise their political, economic, and social affairs varies between countries, with some having just a single national boundary (Level 0) and others having many thousands of districts (L04) and sub-districts (L05), as is the case with France or Rwanda.

Although the data extracted from the sources outlined in **Table S4** and **Table S5** were predominantly municipal level data, our analysis found the specific spatial boundary to which these data relate to be unclear in many cases. For example, data provided for 'London' may relate to either the City of London (population ~ 8,000) or Greater London (population ~ 9 million).

Each municipal waste data record (i.e. from WaW2.0, WaCT, WABI, UNSD, SIPSN or MoHURD) was assigned to a GADM administrative area[1] by comparing the similarity between: 1) The population reported alongside the original primary data record and the population calculated by summing GIS population rasters for the years 2010, 2015 and 2020[29,42] across each GADM polygon; and 2) The urban extent of the city on a Google Maps hybrid layer with the GADM polygon boundary. Once a decision had been made about which administrative area best matched the data record, the GADM ID of that boundary was assigned to the data record. Additionally, a 'GADM match' score was assigned to denote how well we believed the data record matched the administrative area (**Table S6**).

Data for China published in the MoHURD dataset[34] were analysed slightly differently to those outlined in **Table S6** because of major discrepancies between those reported by MoHURD and those in the GADM V3.6 dataset[1]. This is for two main reasons: 1) MoHURD reports data in Chinese script for which translations into Roman Script have undergone methodological changes in recent years and are subject to the interpretation of software or human translator[43]; and 2) The Chinese Authority has implemented substantial reclassification of its sub-provincial administrative areas over recent decades[44], resulting in a mismatch between areas reported in MoHURD and in the GADM.

Table S6. Criteria for level of correlation between administrative areas[1] and municipal waste data records.

Administrative area match score	Criteria
1	The difference in population between that reported in data record compared to that calculated via GIS for the administrative area and for the nearest reported year is less than 20% or has plausibly increased or decreased during the intervening years. Additionally, the administrative area correlates well with the urban area based on Google Maps hybrid layer.
2	The difference in population between that reported in data record compared to that calculated via GIS for the administrative area and for the nearest reported year is greater than 20%, but the administrative area correlates well with the urban area based on Google Maps hybrid layer[45].<br>Alternatively, the difference in population between that reported in data record compared to that calculated via GIS for the administrative area and for the nearest reported year is less than 20%, but the administrative area correlates poorly with the urban area based on Google Maps hybrid layer.

Administrative area match score	Criteria
3	The difference in population between that reported in data record compared to that calculated via GIS for the administrative area and for the nearest reported year is greater than 20%, and the administrative area correlates poorly with the urban area based on Google Maps hybrid layer. Despite this, it is reasonable to conclude the data and administrative area refer broadly to the same location.
4	Unable to find appropriate match between the data record and administrative areas.

To address these challenges, the Chinese script names of the administrative areas (n=708) reported by MoHURD were translated into Roman Script using the Google Translate function within Google Sheets. Of these, 32 are reported by MoHURD as provinces and therefore assigned to Level 1, the remaining 676 were assumed to be Level 2 or 3 and were assigned to the closest matching GADM polygon following a four-step approach (**Table S7**).

Table S7. Description of steps taken to assign incineration and collection data from ministry of Housing and Urban Rural Development (MoHURD)[34] into the administrative areas according to the Database of Global Administrative Areas (GADM)[1].

			Number of municipalities		Number of municipalities assigned			
Step		Description	Removed (merged)	Added	L01	L02	L03	Total
	1a	Level 1 names matched			27			27
	1b	Level 1 names adjusted			4			4
1	1c	Level 1 Xinjiang merged with Xinjiang Uygur	-1		0			0
	2a	Translated Roman script names matched with either Level 2 or 3 unique IDs and population within 60%				113	228	341
2	2b	Translated Roman script names matched with either Level 2 or 3 unique IDs. Population below 60% match but correlation of GADM polygon with conurbations indicated the same area				68	96	164
	3a	Translated names in Roman script or original Chinese script compared with Google, Google maps and GADM layer then adjusted as necessary and allocated to Level 2 or 3 if population within 60%				11	107	118
3	3b	Translated names in Roman script or original Chinese script compared with Google, Google maps and GADM layer then adjusted as necessary. Population below 60% match but correlation of GADM polygon with conurbations indicated the same area				6	27	33
	4a	Municipalities listed by MoHURD did not match with GADM but fell within another GADM boundary, therefore records combined with other validated records	-10				0	0
	4b	Municipalities reported by MoHURD (n=4) matched with two GADM municipalities, so data distributed between them by population	-4	8			8	8
4	4c	Reassessment of population in the context of Level 3 municipalities already allocated showed good match at Level 2				6		6
		**Totals**	**-15**	**8**	**31**	**204**	**466**	**701**

Municipalities reported by MoHURD were assigned to GADM V3.6 polygons sequentially according to the steps detailed. The number of municipalities assigned to each Level during each step are listed under L01, L02, L03. Data for some municipalities had to be merged in steps 1c, 4a and 4b as the GADM reported areas that had since been split into smaller administrative areas by the Chinese authorities. Data for other municipalities had to be redistributed into two municipalities in Step 4b because the Chinese authorities have merged municipalities since creation of the GADM. Abbreviations: Global Administrative Database of Municipalities (GADM).

S.6.4 Data harmonisation (MS3a), correction (MS3b) and quality screening (MS3c)

Municipal waste management data reported by each of the six *primary input data* sources (**Table S4** and **Table S5**) were not collected using consistent criteria and therefore had to be harmonised to enable aggregation into a combined dataset that contained parameters with approximately equivalent basis. Within each dataset, we also took steps to assess: the methods by which data were collected; the quality of the data; and whether data quality assurance had already been carried out by the researchers who compiled them. As shown in **Table S4** and **Table S5**, most of the data sources had only limited quality assurance, meaning substantial cleaning was required.

Numerous authors have highlighted that data reported by municipalities is often incorrect[16,17,31,46]. For example, municipalities often estimate MSW generation by measuring the amount of waste that arrives at a disposal site. However, if some waste is uncollected in the municipality, or if the informal recycling sector collect material before it reaches the disposal site, then that measured quantity would be underreported. Therefore, we corrected some reported MSW generation rates to approximately account for unrecorded material.

This section details the harmonisation, correction, and quality screening steps for each of the six *primary input data* sources used in our model.

S.6.4.1 Waste Wise Cities Tool (WaCT)

The WaCT was developed by UN-Habitat[6] to assist and enable consistent and scientific collection of municipal level waste management related data across the world. The tool guides users through a series of steps aimed at quantifying the flows of waste through municipal solid waste systems, including household and commercial surveys. A WaCT Data Collection Application (DCA) assists users with collecting and analysing data. Summary results of data collected are available online via a dedicated data portal[30].

MSW generation rate ($tP1_{pc}$), collection coverage (tC1) and controlled disposal (tC3) were extracted directly from the WaCT DCA to obtain higher precision than the summarised numbers reported in the WaCT portal UN-Habitat[30].

In the present work, we define controlled disposal using the WACT[6] definition of facilities '*operated under basic, improved or full control according to the Ladder of waste management facilities' control level*'. We then made a series of assumptions (**Sections S.6.4.2 - S.6.4.6)** about how we harmonised other data source definitions with ours.

Additional inputs taken from the WaCT DCA include the percentage of plastic in MSW (*C0*) and the percentage of that plastic that is rigid (*C0a*), termed 'dense plastic' in WaCT. As these are only provided for household composition which we assume to be equivalent to MSW plastic composition. This is a reasonable approximation given that households usually produce the bulk of MSW generation (assumed as 70% wt. in WaCT as a default).

The primary inputs of formal collection of MSW for dry recycling (tC2i), formal collection of MSW for other recovery (tC2ii) and formal collection of MSW for incineration (tC2iii) are not directly reported by WaCT as they all fall within the tools aggregated category of 'recovery facilities'. Despite this, an assessment of formal collection of MSW for incineration (tC2iii) can be made by analysing the recovery facility data available in the WaCT DCA and summing the

mass input to any facility classified as incinerators, before dividing this by the collected mass to achieve the correct basis. This approach cannot be applied for formal collection for dry recycling (tC2i) or formal collection of MSW for other recovery (tC2ii) due to many of the sorting and recovery facilities including contributions from both formal and informal collections. As such, no data was extracted for these data points.

The SDG indicator 11.6.1, 'the proportion of municipal solid waste collected and managed in controlled facilities out of total municipal waste generated, by cities', is not a direct input to the MFA's used in this work, but instead is an output calculated from the MFA's. No further harmonisation, screening, or correction of WaCT data was required.

S.6.4.2 Wasteaware Benchmark Cities Indicators (WABI)

The Wasteaware Cities Benchmark Indicators (WABI) were first developed as a means to compare cities waste management performance as part of the UN-Habitat flagship publication *Solid Waste Management in the World's Cities*[47], although not yet under the WABI name and documented by Wilson, et al.[48]. Later adaptions of the methodology saw the development of WABI as a complete framework and set of indicators to enable consistent solid waste data collection and reporting which would enable assessments and comparison of waste management systems around the world for their effectiveness at controlling waste, social inclusion in waste management and environmental sustainability[39]. Since its publication, the indicators have been used as a basis for over 70 studies, examples of which can be found in[39,48-66].

WABI data used in this analysis is available from Velis, et al.[41], with additional data sourced based on reports that used the WABI framework in China[63], Egypt[64], Ethiopia and South Africa[65]. Wasteaware also provided supplementary information on case studies to aid in the analysis, particularly to ensure consistency across the different versions of the tool. Data years for the WABI dataset were assumed 3 years prior to the publication date of the data for each municipality as reported by Velis, et al.[41]. A data year for Ethiopian cities was only provided for Bishoftu, therefore it was assumed all other Ethiopian cities were profiled in the same year.

The MSW generation rate ($tP1_{pc}$) was calculated from the above data by dividing the reported waste generation ($t{\cdot}y^{-1}$), by the population provided in the dataset, and converting the units to $kg{\cdot}cap^{-1}{\cdot}d^{-1}$. Similarly, collection coverage (tC1) and plastic in MSW (C0) was reported as a percentage of MSW generation, therefore no further processing was necessary.

We assumed that the definition of controlled treatment and disposal facilities defined by indicator *2E* used in the WABI[39] is equivalent to the definition of controlled disposal used in this analysis. Although this indicator relates to both treatment and disposal facilities, in practice the indicator is mainly used to describe disposal facilities only. Similarly, as the units of this indicator in WABI are as a percentage of waste destined for treatment or disposal, the units matched closely with that required for the controlled disposal input (tC3), therefore no further processing was needed.

The *primary data inputs* of formal collection of MSW for dry recycling (tC2i), formal collection of MSW for other recovery (tC2ii) and formal collection of MSW for incineration (tC2iii) are not directly reported as part of the WABI. Instead, the WABI reports a recycling rate that includes dry recycling by both formal and informal sectors, plus organics valorisation (e.g., composting, anaerobic digestion and animal feeding). Supplementary information associated with the WABI case studies[41] allowed many of the recycling data points to be disaggregated

between the proportion that was reported as formal recycling compared to that which was informally collected. Though the informal sector is involved in recycling some wet wastes, it is predominantly focused on dry material, therefore, we assumed that all informal recycling reported in WABI was dry recycling. This enabled the WABI recycling rate to be adjusted so that it only included formal recycling, thereby becoming closer to that required by the *primary data inputs*. Importantly, informal recycling rates are included in our analysis, however, these are modelled and added on as part of the *secondary data inputs* (**Section S.7**). Lastly, to enable complete harmonisation with the *primary data inputs* of formal recycling (tC2i) and other recovery (tC2ii), the formal recycling rate was split into the proportion that is related to dry recycling, and the proportion sent for organics valorisation (‘other recovery’). As this was not explicitly recorded for many records in the WABI dataset, we obtained evidence from literature for each municipality to estimate this split (**Table S8**).

Table S8. Review of evidence for municipalities in the Wasteaware Cities Benchmark Indicators (WABI) dataset with reported formal recycling with the aim to understand the split between formal dry recycling and other recovery.

**Municipality**	**Country**	**Proportion of WABI formal recycling that is dry recycling**	**Justification**	**Source**
Adelaide	Australia	77.5%	62% dry recycling and 18% composting reported as a percentage of waste generation	67
Varna	Bulgaria	100%	Evidence of a recycling facility in Varna processing household waste for recycling but no mention of any other recovery facility type, therefore allocated completely to dry recycling	68
Bahrain	Bahrain	100%	Although the reference suggest both dry recycling and composting facility exist, the latter is reported to have negligible flows. As such, dry recycling is assumed to represent the entire amount of the WABI recycling value.	69
Belo Horizonte	Brazil	100%	Evidence of formal cooperative waste pickers working alongside informal waste pickers. No evidence of other recovery such as composting so all assigned to dry recycling.	70
Victoria-Gastez	Spain	100%	Paper, plastics and glass reportedly recycled. No evidence of composting, therefore all recycling assigned to dry recycling.	71
Rotterdam	Netherlands	57.7%	Based on 15% composting and 11% dry recycling in South Holland	72
Belfast	Northern Ireland	59.1%	15.9% dry recycling and 11% composting	73
Athens	Greece	99.6%	99.6% dry recycling with only 0.4% composting of restaurant waste	74
Delhi	India	0%	Dry recycling reportedly performed largely by the informal sector. NGO’s encouraged to perform composting, therefore all formal recycling allocated to composting.	75
Dhaka	Bangladesh	0%	Evidence of a composting plant in operation along with collection services for market waste	47
Castries	St Lucia	2.5%	Evidence of some formal dry recycling facilities present in Castries therefore it is plausible that the 2.5% is formal	76
Singapore	Singapore	81.4%	Approximated from a chart – Singapore includes several non-municipal sources so the reported rate of 59% was adjusted by deducting construction waste (29%) and slag (8.5%) – leaving 21.5%. Of this, the combined proportion of horticultural waste and food waste was 4%; assumed composted or sent for anaerobic digestion. This means the formal dry recycling rate was 81.4% of all formal MSW recycling	77

Municipality	Country	Proportion of WABI formal recycling that is dry recycling	Justification	Source
Curepipe	Mauritius	-	Evidence that although some collection of dry recyclables occurs by the formal sector, this is mixed together with residual waste at the transfer station and taken to disposal sites, therefore omitted.	47
Canete	Peru	100%	Separate collection of inorganic recyclables available in about 15% of the municipality.	47
Jakarta	Indonesia	-	Unable to source reliable data to justify the 5% reported, however both waste banks and compost facilities are reported to exist. Therefore omitted.	78
Ghorahi	Nepal	100%	A small amount of plastics are sorted for recycling formally at the landfill site. Although compost pits are also present at the landfill site, it is reported they have difficulty selling this due to glass contamination. As such, the dry recycling is assumed the dominant part of formal recycling.	47
Quezon City	Philippines	-	Formal *barangay* collectors are reported to have material recovery facilities for dry recycling but also collect biodegradable waste for composting. It is unclear of the relative split between these activities, therefore an equal split is assumed.	47
Managua	Nicaragua	100%	Believed to be due to waste picker cooperatives therefore assigned to dry recycling	47
Luskau	Zambia	100%	Reported there is a strong formal sector with five recycling companies collection paper, plastics and metal.	47
Surat	India	-	Unable to source reliable data therefore omitted	
Bangalore	India	-	Unable to source reliable data therefore omitted	
Warangal	India	-	Unable to source reliable data therefore omitted	
Bishkek	Kyrgyzstan	93.75%	Material flow analysis suggest 500 tonnes per year are composted formally, whereas 7500 tonnes per year of paper goes to recycling factories directly (assumed formal). Therefore 93.75% of formal recycling is dry recycling	79
Lahore	Pakistan	0%	All formal recycling is composting	80
Castries	St Lucia	100%	Dry recyclables reportedly collected. No evidence of composting or other recovery	76
San Francisco	USA	72.2%	72.2% dry recycling with the remainder composting	81
Tompkins county	USA	100%	Evidence of material recovery facilities and mixed dry recyclables collection at source but no mention of other recovery facilities.	82

Abbreviations: Municipal solid waste (MSW); non-governmental organisation (NGO); WasteAware Benchmark Indicators (WABI).

The splits found in **Table S8** were used to disaggregate the WABI formal recycling rate by dry recycling and other recovery. As the units of the WABI recycling rate are as a percentage of waste generation, the values were further divided by the reported collection coverage to convert the units to a percentage of collected waste, thereby matching those required for tC2i and tC2ii.

Lastly, incineration is not directly reported as part of the WABI dataset. To populate the primary data input of ‘collected for incineration’ (tC2iii), we gathered evidence to determine whether incineration was taking place in each municipality. The municipalities in which incineration was found to occur is shown in **Table S9**.

Table S9. Amount of waste incinerated in municipalities profiled using the WABI method.

Municipality	Country	Mass incinerated ($t \cdot y^{-1}$)	Proportion MSW incinerated (% of MSW generation)	Source
Kunming	China	1,382,368	73	
Bengbu	China	369,619	73	
Lanzhou (Lan'Zhou)	China	870,459	100	34
Suzhou	China	1,898,138	77	
Taian (Tai'an)	China	413,755	64	
Xian (Xi'an)	China	140,750	94	
Rotterdam	Netherlands		76.23[a]	47
Singapore	Singapore		38	77

[a] based on the statement that all residual waste is incinerated with only 1% of residues sent to landfill and 23% recycling, anaerobic digestion and composting reported in the WABI dataset Abbreviations: municipal solid waste (MSW).

In all cases, collection coverage reported for municipalities which incinerate waste was 100%, therefore, the units of percentage of MSW generation are equivalent to the units of percentage of MSW collected. As such, no further processing was required and the values in **Table S9** were used directly as input tC2iii.

S.6.4.3 What a Waste 2.0 (WaW2.0)

The What a Waste 2.0 dataset provided by Kaza, et al.[31] reported waste data collected from 367 cities covering nearly every country. Data were obtained by Kaza, et al.[31] from literature and conversations with waste agencies and authorities. Data sources in WaW2.0 are listed in the 'City level codebook' that accompanied the report.

S.6.4.3.1 Collection coverage

The WaW2.0 dataset includes four fields which are used to report collection coverage using different units. For some cities no data are reported in any field, others just one field and others two, three, or four. We assumed they were all equivalent estimates to collection coverage as a percentage of MSW generation by mass (tC1), and selected them for inclusion in our dataset according to the following order of the following preference:

1. % wt. of waste
2. % of population
3. % of households
4. % of geographical area

S.6.4.3.2 MSW generation rate

The amount of MSW generated in each municipality is reported by WaW2.0 in $t \cdot y^{-1}$. We divided these rates by the population reported in the dataset itself and then multiplied by (1000/365) to adjust the units to $kg \cdot cap^{-1} \cdot d^{-1}$.

Approximately 30% of the waste generation entries also report whether scales are used to weigh the mass of waste collected, and the location at which it was measured. For example, of the 100 cities that reported the measurement method, 69 reported scales were used at the point of disposal, five at the point of aggregation (e.g., transfer stations), 16 did not have a measurement method, and ten reported 'other'. It was assumed that the MSW generation rates were based on

measurements taken from these weighbridges when provided. This implies that many of the reported waste generation rates represent collected waste only. Therefore, if collection coverage is less than 100%, the total MSW generation rate has been underreported.

There is evidence that some municipalities and countries may correct their waste generation data on the basis of waste collection and other factors, for instance for some municipalities in Brazil[83]. There is also some evidence that waste generation is reported as that which has been 'collected and transported', for instance by National Bureau of Statistics of China[84]. Without checking each individual record by either re-requesting the information from the municipality or following up the published source, it was not possible to determine whether the data had already been corrected. Moreover, for most records (n = 267) in the WaW2.0 city database, the point of measurement was left blank, creating uncertainty over where the waste was measured and also whether it was corrected.

To address the potential underestimation of waste generation rates, we carried out a cautious adjustment by dividing the waste generation rate by the collection coverage. For cities in high-income countries (HICs), the difference between the reported and adjusted waste generation was negligible because most cities in HICs reported collection coverage at or close to 100%. For cities in upper-middle income countries (UMCs), lower-middle income countries (LMCs) and low income countries (LICs), the difference between the adjusted waste generation rate and the original waste generation rate was progressively greater as the collection coverage negatively correlated with income category, a commonly observed trend[5,31].

Analysis of the central tendency and spread of the adjusted waste generation data showed that for some records, cities in UMCs, LMCs and LICs generated substantially more waste than in many HIC municipalities (**Table S10**). Whilst parts of some wealthier cities in the Global South may approach comparability with some poorer cities in HICs, we assumed that it is unlikely that the median waste generation would exceed that in HICs. Therefore, to control for potentially overestimated waste generation rates, we screened the adjusted waste generation data to assess the plausibility of our corrections according to the following criteria:

1. Adjusted waste generation rates for cities in LIC and LMC countries that were greater than the median waste generation mass for HICs (1.02 $kg \cdot cap^{-1} \cdot d^{-1}$; n = 60) were assumed to be overcorrected and flagged for potential reversion to the original reported figure.
2. Adjusted waste generation rates for cities in UMC and HICs that exceeded 1.5 times the interquartile range from the 75th percentile[85] were assumed to be outliers (n = 5) and flagged for potential reversion to the original reported figure.

Cities flagged for a potential correction were screened to identify plausible explanations for a high waste generation, for instance, for extremely high tourism. Three cities: Hanoi (Vietnam), San Pedro (Belize) and Honiara (Solomon Islands) were identified as being major tourist destinations. For each of these three, tourist arrivals statistics were compared with the resident population to see if there was a substantial inferred increase in population for long enough to affect the waste generation mass. In each case, we decided that the increase was not great enough to warrant the increase. Therefore, all the flagged records were reverted (n = 65), reducing the spread of the data.

Table S10. Side by side comparison of central tendency and spread for waste generation mass reported in the WAW2.0 dataset[31] compared to mass adjusted by collection coverage (kg•cap^{-1}•d^{-1}).

Dataset	Central tendency and spread	LIC	LMC	UMC	HIC
Original data	25th percentile	0.27	0.43	0.66	0.65
	Median	0.48	0.58	1.01	1.01
	75th percentile	0.70	0.85	1.26	1.41
	Inter quartile range	0.43	0.42	0.59	0.76
Adjusted ('corrected')	25th percentile	0.34	0.47	0.74	0.66
	Median	0.67	0.75	1.06	1.02
	75th percentile	1.37	1.32	1.38	1.41
	Inter quartile range	1.03	0.85	0.65	0.75

As shown in **Table S11**, the 75th percentile for cities in LICs and LMCs of adjusted waste generation rate with the 65 outliers removed reduced substantially, whereas the data for UMCs and HICs were barely affected.

Table S11. Central tendency and spread of waste generation mass reported in the WAW2.0 dataset[31], adjusted by collection coverage with the adjustment reverted for some records to control outliers.

Dataset	Central tendency and spread	LIC	LMC	UMC	HIC
Corrected with some corrections reverted	25th percentile	0.34	0.46	0.70	0.66
	Median	0.55	0.64	1.06	1.02
	75th percentile	0.75	0.88	1.34	1.41
	Inter quartile range	0.41	0.43	0.64	0.75

S.6.4.3.3 Plastic in MSW

The composition of MSW is reported in WaW2.0, including a category for plastics. If the summation of the compositions did not equal 100%, values were normalised then assigned to 'plastic in MSW' (*C0*).

S.6.4.3.4 Recovery and controlled disposal

The proportion of waste that was treated and disposed of is reported in WaW2.0 under 12 categories for 247 cities. Although the questionnaire used by WaW2.0 stated that respondents should report these categories as a proportion of waste generation, we assumed that, for the majority of cases, it was reported as a proportion of 'formally collected waste'. Our assumption is further supported by the fact that 59 cities reported informal recycling rates (as a percentage of waste generation), yet only six of these cities ensured that the summation of this informal recycling with the formal treatment and disposal options equalled 100%. By contrast, most of the cities with data on informal recycling reported that the other 12 treatment and disposal options summed to 100% (n = 32), whilst the remainder (n = 21) summed to less than 100%. Examples such as this indicated inconsistencies and errors, which fell into four main groups:

1. In approximately half of cases, the ‘unaccounted for’ category appeared to represent ‘uncollected waste’ rather than material collected and transported. This implies that some municipalities had followed the instructions and reported proportions as a percentages of waste generation, whilst the other half had used it to represent collected waste for which the data to describe the treatment and disposal pathway was not known.
2. Data for informal sector recycling were reported as a proportion of waste generation (n = 59), yet when combined with the 12 other treatment and disposal options, the majority (n = 53) did not sum to 100%.
3. Only recycling was reported (n = 5) and the other categories were left blank.
4. The sum of categories added up to more or less than 100% (n = 50).

To approximately correct the inconsistent use of the ‘unaccounted for’ field **(1)**, we assumed that if the sum of ‘unaccounted for’, ‘waterways marine’ and ‘collection coverage’ fields were within 10 percentage points of 100%, then the ‘unaccounted for’ field represented ‘uncollected waste’ (n = 65). In all other cases we assumed that the ‘unaccounted for’ field represented collected and transported waste that had been deposited in an unknown, uncontrolled facility (n = 302).

If data for informal recycling sector collection **(2)** was within 10% of the reported ‘waste_treatment_recycling_percent’ field, it was assumed both fields represent informal recycling and therefore the data point was removed from the analysis (informal sector recycling was instead estimated using a modelling approach to ensure more consistent estimations.

Where only the ‘recycling’ field was reported **(3)**, data were left intact, and the other categories were left blank exactly as entered. Where the sum of the proportions was less than or greater than 100% **(4)**, we normalised each of the reported categories to 100%. If the summation of the treatment and disposal options prior to normalisation summed to 100%, but some of the inputs were left blank, it was assumed that no other treatment and disposal methods were present in that municipality. The blank treatment and disposal options were therefore allocated zeros instead of blanks. If the pre-normalised values did not sum to 100% the blanks were unchanged.

Each of the treatment and disposal types in WaW2.0 were assigned *primary data variables* according to The World Bank[86] country income category of the municipality (**Table S12**). The *primary data inputs* for formal collection for dry recycling (tC2i) and incineration (tC2iii) each relate to only a single WaW2.0 category, therefore the proportions reported were used following the above corrections. Other recovery (tC2ii) was calculated as the sum of the proportions allocated to the ‘composting’, ‘anaerobic digestion’ and ‘advanced thermal treatment’ WaW2.0 categories. As the units for these were assumed as a percentage of collected waste, no further processing was required. By contrast, the *primary data input* variable of ‘controlled disposal’ (tC3) is a proportion of waste collected for disposal, therefore this input was calculated as the sum of the percentages assigned as controlled disposal, divided by all percentages assigned to disposal.

Table S12. Classification of municipal solid waste treatment and disposal categories reported in What a Waste 2.0 (WaW2.0)[31] by country income categories.

WaW2.0 treatment and disposal categories	Classification assigned in this work by income category of country	
	HIC	UMC, LMC, LIC
Recycling	Formal collection for dry recycling (tC2i)	Formal collection for dry recycling (tC2i)
Compost	Other recovery (tC2ii)	Other recovery (tC2ii)
Anaerobic digestion	Other recovery (tC2ii)	Other recovery (tC2ii)
Advanced thermal treatment	Other recovery (tC2ii)	Other recovery (tC2ii)
Incineration	Incineration (tC2iii)	Incineration (tC2iii)
Landfill gas system	Controlled disposal (tC3)	Controlled disposal (tC3)
Controlled landfill	Controlled disposal (tC3)	Controlled disposal (tC3)
Landfill unspecified	Controlled disposal (tC3)	Uncontrolled disposal
Open dump	Uncontrolled disposal	Uncontrolled disposal
Other	Controlled disposal (tC3)	Uncontrolled disposal
Marine / river	Uncontrolled disposal	Uncontrolled disposal
Unaccounted [1]	Uncontrolled disposal or uncollected	Uncontrolled disposal or uncollected

[1]Analysis of the City Dataset reported in WaW2.0[31] indicates confusion amongst some of the respondents to the survey.

In approximately half of the cases, it appears that the 'unaccounted for' field was used to represent 'uncollected waste', whereas in the other half of cases it was used to represent collected waste for which the data to describe the treatment and disposal pathway was not known. To correct these inconsistencies, we assume that if the sum of 'unaccounted for' and 'collected' waste is within 10 percentage points of 100%, then the 'unaccounted for' field represents uncollected waste. In all other cases, we assume that the 'unaccounted for' field represents collected waste that has been deposited in an uncontrolled facility. Abbreviations: high-income country (HIC); upper-middle income country (UMC); lower-middle income country (LMC); low-income country (LIC); What a Waste 2.0 (WaW2.0).

S.6.4.3.5 Formal dry recycling

On the basis that anaerobic digestion and composting are reported separately in WaW2.0[31], it was assumed that the recycling rate reported is for dry recycling only.

While anaerobic digestion and particularly composting have become more common in LICs, LMCs and UMCs[31], collection of dry recyclate by the formal sector is uncommon or small in comparison to the informal sector[87]. As we will show in this section, this is except for some cities in UMCs that have begun to implement small-scale formal recycling collection systems. Thus, the majority of WaW2.0 records for cities in LICs, LMCs and UMCs that included data for 'recycling' are likely to represent waste collected by the informal sector rather than by the formal sector. We suggest that this may even be the case for the cities where the informal sector recycling field was left blank due to insufficiently defined reporting between the formal and informal sector activities, making disaggregation challenging.

To assess whether the recycling rate in WaW2.0 represents formal collection for recycling, the following assumptions and data verification steps were conducted:

1. Recycling rates reported for cities in HICs were assumed to describe formal collection for dry recycling collection as a proportion of waste collection.
2. Recycling rates reported for cities in LICs and LMCs were assumed to describe informal recycling sector dry recycling collection as a proportion of waste collection. In these cases, formal collection for dry recycling was marked as zero.

3. For cities in UMCs, evidence was collated from municipal websites, reports, and academic articles to determine whether formal collection for dry recycling was being carried out in the municipality (**Table S13**). This consisted of three tests:

    a. Is there evidence that the formal sector recycling is taking place in the municipality?
    b. Is the recycling rate reported so high that it is implausible that it is entirely carried out by the formal sector?
    c. Is the recycling rate low enough that it is implausible that it only represents informal collection and is therefore more likely to represent a small formal operation?

Records marked as ‘plausible’ were assumed to be representative of formal recycling; ‘unlikely’ were assumed to represent informal recycling and marked with a zero; and ‘uncertain’ data points, where it was unclear what the data represented, were removed.

Table S13. Evidence that formal recycling takes places in the municipalities reported by What a Waste 2.0[31].

**Municipality**	**Country**	**Reported recycling rate (% of collected waste)[1]**	**Plausibility that recycling rate is formal**	**Reason**	**Ref**
Vlora	Albania	10	Unlikely	Thriving informal sector and no evidence of formal sector recycling	[88]
Algiers	Algeria	10	Unlikely	No evidence of formal recycling and evidence of strong informal sector	[89]
Cordoba	Argentina	0.68	Plausible	Recycling rate low and evidence that formal recycling takes place	[90]
Ciudada Autonomous De Buenos Aires	Argentina	7.2	Unlikely	Thriving informal sector and little evidence of formal sector recycling	[90]
Grodno	Belarus	0.6	Plausible	Recycling rate low and evidence that formal recycling takes place	[91]
Distrito Federal, Brasilia	Brazil	5.94	Unlikely	Thriving informal sector and little evidence of formal sector recycling	[92]
Rio De Janeiro	Brazil	0.5	Plausible	Recycling rate low and some small evidence that formal recycling takes place	[93]
Bogota	Colombia	17	Plausible	Evidence that informal sector has become fully formalised	[94,95]
Medellin	Colombia	16	Plausible	Evidence that informal sector has become fully formalised	[96]
Cali	Colombia	15	Plausible	Evidence that informal sector has become fully formalised	[97]
San Jose	Costa Rica	5.2	Plausible	Some evidence that formal recycling takes place	[98,99]
Alajuela	Costa Rica	0.42	Plausible	Recycling rate low and some small evidence that formal recycling takes place	[98,99]
Quito	Ecuador	6	Unlikely	No evidence of formal recycling and evidence of strong informal sector	[100,101]
Guatemala City	Guatemala	5	Unlikely	No evidence of formal recycling and evidence of strong informal sector	[102]

Municipality	Country	Reported recycling rate (% of collected waste)[1]	Plausibility that recycling rate is formal	Reason	Ref
Tehran	Iran, Islamic Rep.	4	Plausible	Evidence of formal recycling	[103]
Beirut	Lebanon	5	Unlikely	No evidence of formal recycling and evidence of strong informal sector	[104]
Saida	Lebanon	20	Plausible	Evidence of formal recycling	[105]
Skopje	Macedonia, FYR	3	Unlikely	No evidence of formal recycling and evidence of strong informal sector	[74,106]
Kuala Lumpur	Malaysia	10.4	Plausible	Evidence of formal recycling	[107]
Mexico City	Mexico	14.19	Plausible	Potentially plausible, but recycling rate is perhaps too high to be carried out formally for a UMC. However, as references claim that IRS is prohibited in Mexico City, it was therefore assumed plausible)	[66,98]
Guadalajara	Mexico	8	Unlikely	Evidence that it is informal recycling	[108]
Cusco	Peru	0.3	Plausible	Recycling rate low and evidence that formal recycling takes place	[100]
Cluj-Napoca	Romania	13.72	Uncertain	Evidence for formal recycling is very weak and slightly stronger evidence of a thriving informal sector. Uncertain that such a high recycling rate would be entirely form formal recycling in an UMC	[109]
Bucharest	Romania	9.44	Plausible	Evidence for a strong formal sector recycling effort	[74,110]
Moscow	Russian Federation	4	Unlikely	Evidence for a strong formal sector recycling effort	[111]
St. Petersburg	Russian Federation	10	Unlikely	Evidence of some small scale formal recycling initiatives such as bring sites	[112,113]
Kemerovo	Russian Federation	1.9	Plausible	Evidence for a strong formal sector recycling effort	[114]
Novi Sad	Serbia	2	Unlikely	Evidence that formal recycling is around 0.4% so 2% is assumed too high	[115]
Bangkok	Thailand	11.85	Unlikely	Strong evidence for informal sector and recycling rate likely too high for a UMC	[116]
Vavau	Tonga	5.1	Plausible	Strong evidence for formal recycling system	[117]
Sakarya Mm	Turkey	2.49	Plausible	Evidence for formal recycling system in place	[118]
Caracas	Venezuela, RB	0.9	Plausible	According to source, recycling is the 'responsibility' of the municipality but seems to be limited in scope and coverage –therefore such a small amount seems plausible	[119]

[1] Although recycling rates were supposedly reported as a percentage of waste generation, it is assumed that most municipalities reported their recycling rates as a percentage of collected waste for reasons previously discussed.

S.6.4.3.6 Incineration

Data reported in WaW2.0 dataset under the 'incineration' category were sense checked for plausibility using several databases and other sources[120] listed in **Table S14**. Where incinerators with sufficient capacity to process the amounts likely to be generated in a city existed near the municipality, we considered them plausible. In two cases (Angers-Loire Metropole and Trnava), no incinerator was close-by, however the proportions reported were very small, so it was plausible that small amounts or, perhaps, hazardous waste were being transported to incinerators

which were in nearby municipalities. Therefore, it was considered plausible that the amounts stated were being incinerated.

Table S14. Evidence that incineration takes places in municipalities reported by What a Waste 2.0[31].

Municipality Name	Country Name	Data Year	Incineration rate	Plausibility of incineration	Justification	Reference
Baku	Azerbaijan	2013	39.97	Plausible	Baku waste to energy plant installed 2012 cap 550,000 $t{\cdot}y^{-1}$	120
Liege	Belgium	2014	26.00	Plausible	Intradel Herstal plant installed 2009 cap 320,000 $t{\cdot}y^{-1}$	(98)
Beijing	China	2015	8.00	Plausible	Incineration in 2019 was 54%[34], and although it does not go back to 2015, 8% is commensurate with the general increase in Incineration over the past decade[121].	34
Paris	France	2015	77.50	Plausible	Eight MSW incinerators located in Paris	120
Angers-Loire Metropole	France	2015	0.23	Plausible	Incinerators at Nates and Chinon, far but within reasonable proximity to process such a very small amount of waste	120
Berlin	Germany	2015	65.00	Plausible	Incinerator with 3.6 M $t{\cdot}y^{-1}$ capacity since 1967	120
Budapest	Hungary	2014	52.00	Plausible	Hulladékhasznosító Mű (HHM) has 17 Mt capacity since 2005	120
Delhi	India	2014	52.04	Unlikely	Delhi has one incinerator operational since 2011 with 225,000 $t{\cdot}y^{-1}$, so it cannot be plausible that it has treated half the waste in the city in 2014. At least one is functional since, but it was not ready at the time.	120
Kanpur	India	2016	42.86	Unlikely	No record found of an incinerator here	120
Tehran	Islamic Republic of Iran	2014	2.50	Unlikely	No record found of an incinerator here	120
Milano	Italy	2015	43.47	Plausible	Incinerator with 1.4 $Mt{\cdot}y^{-1}$ capacity reported here	120
Osaka	Japan	2015	78.07	Plausible	Nine incinerators reported to be operational in the municipality	120
Kobe	Japan	2015	72.60	Plausible	Five incinerators reported to be operational in the municipality	120
Naha	Japan	2015	81.50	Plausible	Clean Center Naha Haebaru incinerator operational since 2006 170,00 $t{\cdot}y^{-1}$	120
Toyama	Japan	2015	68.21	Plausible	Clean Center Toyama incinerator 270,000 $t{\cdot}y^{-1}$ operational since 2003	120
Kitakyushu	Japan	2015	64.92	Plausible	Three incinerators operational in the municipality	120
Yokohama	Japan	2015	65.55	Plausible	Four incinerators operational in the municipality	120
Seoul	Korea, Rep.	2012	8.00	Plausible	Five incinerators operational in the municipality	120
Oslo	Norway	2013	57.85	Plausible	Two incinerators operational in the municipality	120
Bergen	Norway	2014	39.10	Plausible	BIR Avfallsenergi AS incinerator operational since 1999 and upgraded in 2010	120
Lahore	Pakistan	2017	6.15	Unlikely	No record found of an incinerator here	120

Municipality Name	Country Name	Data Year	Incineration rate	Plausibility of incineration	Justification	Reference
Trnava	Slovak Republic	2010	0.34	Plausible	Proximity to Bratislava which has an incinerator suggests that such a small quantity could be plausibly transported there	[120]
Bratislava	Slovak Republic	2013	41.02	Plausible	Incinerator with 135,000 $t \cdot y^{-1}$ capacity reported here	[120]
Madrid	Spain	2014	10.00	Plausible	Incinerator with 314,000 $t \cdot y^{-1}$capacity reported here	[120]
Stockholm	Sweden	2013	71.01	Plausible	Incinerator with 700,000 $t \cdot y^{-1}$ capacity reported here	[120]
Boras	Sweden		54.62	Plausible	Incinerator with 109,000 $t \cdot y^{-1}$ capacity reported here	[120]
Kiev	Ukraine	2016	24.57	Plausible	Incinerator with 450,000 $t \cdot y^{-1}$ capacity reported here since 1988	[120]
London	United Kingdom	2012	46.34	Plausible	At least one incinerator and several fuel producing MBT plants reported here during the timescale	[120]
Hanoi	Vietnam	2014	6.59	Unlikely	Nam Son solid waste treatment complex (SWTC) incinerator has 100,000 $t \cdot y^{-1}$ capacity reported here	[120]

Abbreviations: Million tonnes (Mt); mechanical biological treatment (MBT); municipal solid waste (MSW).

S.6.4.3.7 Data Year

The years that data were collected for WaW2.0 records were recorded by the World Bank in a downloadable ‘city level codebook’[122]. Years were provided for both the population and the year of waste generation; however, the other data points were not assigned a data year. Here, we assumed the data year for the waste generation also applies to all other waste data points of that record, albeit we acknowledge there is uncertainty in this assumption. When the year of waste generation was not available, the data year was left blank, but the records were retained in the analysis to maximise the number of data points.

S.6.4.4 UNSD City Waste Data

Municipal solid waste management data[33] was provided by the United Nations Statistical Division (UNSD) on the 23rd April 2021.

The data forms part of the UNSD Environmental Indicators database, populated by national statistic offices and ministries of environment and collected by means of a biennial questionnaire[40]. The raw data includes information for 237 cities across the World for multiple years spanning from 1989 to 2019; however, not all cities submit complete records for all years. According to their operation protocols, data are accepted by UNSD without further adjustment aside from basic data coherence checks (e.g., percentages sum to 100%). As such, some data entries appear to have been erroneously entered by respondents necessitating thorough cleaning, as described in this section.

S.6.4.4.1 Waste generation rate

The municipal waste generation rate of a municipality was calculated using three different methods, prioritised in the following order:

Method 1: The total amount of MSW generated and population of the municipality for the corresponding year were used to calculate the MSW generation rate per capita ($tP1_{pc}$) for the most recent available year.

Method 2: Total MSW collected was divided by the collection coverage to estimate total MSW generated and then divided by the population reported for the corresponding year to calculate the MSW generation rate per capita ($tP1_{pc}$). If the collection coverage was not reported, the total MSW collected was not used as this would exclude any uncollected waste.

Method 3: For cities that did not report data for the total MSW collected, but instead provided information of the amounts entering treatment and disposal facilities, it was assumed that the summation of the amounts entering the treatment and disposal facilities is equal to the total amount of MSW collected. The same process as method two was then repeated.

Only 31 cities reported waste generation according to *Method 1*, of which four of these (Lalitpur, Kathmandu, Biratnagar and Niamey) reported values inconceivably low (< 1.0 $kg \cdot cap^{-1} \cdot y^{-1}$) and were therefore removed. The waste generation rate was estimated using *Method 2* for a further 73 cities, although again four of these data points (Escuintla, Cobán, Huehuetenango, Rusape) were removed during initial screening due to the values being inconceivably high (> 10 $kg \cdot cap^{-1} \cdot d^{-1}$). Lastly, an additional six cities relied on *Method 3* for calculation of waste generation rate, of which one (Masvingo) was removed during screening based on an implausibly low value (0.04 $kg \cdot cap^{-1} \cdot d^{-1}$). In total, this resulted in 101 data points for MSW generation rate.

S.6.4.4.2 Collection coverage

Collection coverage is reported in the UNSD dataset as percentage of population served. The most recent year was taken for this variable when available, resulting in 135 inputs for collection coverage. To increase this further, the collection coverage was also calculated for cities that did not report collection coverage but did report the amounts entering treatment and disposal facilities, and the amounts generated overall. This resulted in a further 7 cases for which the collection coverage had not been previously reported.

S.6.4.4.3 Formal dry recycling

The UNSD waste questionnaire[40] asks respondents to detail the amounts of waste going to ‘recycling’, ‘composting’, ‘incineration’ (with a subset for ‘incineration with energy recovery’), ‘landfill’ (with a subset for ‘controlled landfill’), and ‘other’.

The primary data input in this work of formal collection for recycling (tC2i) has units of percentage of collected waste. Accordingly, the mass entries provided for recycling in the UNSD dataset were divided by the data point for mass of collected waste. However, in many cases, inconsistencies in the reported data meant this had to be done cautiously. The following rules and priorities were used in calculating the recycling rate:

1. If the sum of the five recovery and disposal options summed to within ±20% of the mass reported as collected, the recycling rate was taken as the mass reported for recycling divided by the mass collected. Data calculated in this manner were assumed the most reliable and used as priority.

2. Occasionally, data records reported a mass collected from households but did not provide an overall collected amount. When the sum of the treatment and disposal options were within ±20% of this household collected mass, it was assumed the household collected mass was misaligned and was instead taken as overall mass collected. Recycling rates were then calculated in the same manner as in 1.
3. If mass was provided only for recycling and collected waste (i.e., no other treatment and disposal options were recorded), the recycling rate was calculated based on the recycling mass divided by the collected mass.
4. In cases where the sum of the treated and disposed mass was not within ±20% of the collected waste, the recycling rate was still calculated but instead using the treated and disposed mass as the denominator. Deviation of masses does not necessarily reflect incorrect data as the masses may deviate due to either rounding errors, based on deviations from sampling, or due to import / export of waste between municipalities. As such, recycling was still calculated in this manner, but only used when the above options were not possible.
5. If no mass was provided for recycling, but the sum of the treatment and disposal options were within ±20% of the collected waste, it was assumed that no recycling occurs and therefore the recycling rate was set as 0%.

No distinction is given in the UNSD definition[40] provided for recycling on whether this includes informal sector recycling or not. Given the questionnaire states that the treatment and recovery values should sum up to the amounts of waste collected (minus exports), and that this collected waste is defined as that collected '*on behalf of municipalities (by public or private companies)*'; it is assumed the mass provided for recycling is intended to relate to formal recycling only. It is unclear whether respondents also took this to be the case and therefore whether the recycling rates reported include informally recycled material or not. The recycling rates calculated as per the above were therefore adjusted in the same manner as for the WaW2.0 dataset. Namely, the 28 LMC and LIC cities that had a non-zero recycling were assumed to be reporting informally collected waste for recycling, particularly given many of the rates calculated were comparable to those of HIC. The recycling rates for these cities were therefore set to zero for tC2i – formal collection for recycling. Alternatively, the recycling rates for HIC were assumed to represent formal collection for recycling and therefore taken directly, whilst data points greater than zero in UMC were checked for plausibility by means of gathering evidence (**Table S15**).

Table S15. Evidence that formal recycling takes places in the municipalities reported in UNSD city waste data[33].

Municipality	Country	Reported recycling rate (% of collected waste)	Year	Plausibility	Reason	Reference
Adrar	Algeria	10.00	2015	Unlikely	Some evidence of the formal sector, however, seems that the informal sector still manages the bulk of the countries recycling. Government initiatives in place to increase reuse but seems to be limited focus on recycling.	123,124
Djelfa	Algeria	10.00	2015	Unlikely	Noted as being an area with thriving informal recycling sector. Formal initiatives seem to focus on reuse not recycling.	123,125

Municipality	Country	Reported recycling rate (% of collected waste)	Year	Plausibility	Reason	Reference
Algiers	Algeria	10.00	2015	Unlikely	Little evidence of formal recycling and evidence of strong informal sector. Sorting sites have little structure, and it is reported that many of these are no more than just a landfill.	125,126
Wahran (Oran)	Algeria	10.00	2015	Unlikely	Seem to be some initiatives in Oran for formal recycling but most of these appear to have been reported more recently than this data. Still seems to be a large informal sector in the municipality.	127-129
Qacentina (Constantine)	Algeria	10.00	2015	Unlikely	Shortcomings in any formal processes that are in place and most recycling is done through the informal sector.	130
El Djazair (Algiers)	Algeria	10.00	2015	Unlikely	Little evidence of formal recycling and evidence of strong informal sector. Sorting sites have little structure, and it is reported that many of these are no more than just a landfill.	125,126
Minsk	Belarus	20.28	2019	Plausible	26% recycling rate reported in Minsk. Unclear if this is all from the formal sector but it does seem that the government are trying to provide recycling facilities in the area. On the other hand, there is some evidence of the informal recycling sector in Minsk.	131-133
Zenica	Bosnia and Herzegovina	4.76	2009	Plausible	Evidence 5% recycling rate for formal sector in the municipality.	134
Gaborone	Botswana	0.24	2017	Unlikely	Evidence suggests that all recycling is collected by informal sector.	135
Francistown	Botswana	0.25	2017	Unlikely		
Brasília	Brazil	2.49	2015	Unlikely	Thriving informal sector and little evidence of formal sector recycling.	92,136
Salvador	Brazil	0.48	2011	Unlikely	Evidence that selective collection did not exist in any formal sense before 2014, therefore this is unlikely to be formally collected.	137
São Paulo	Brazil	0.98	2015	Unlikely	25 coops are authorised in Sao Paulo – it is assumed the reported recycling rate relates to these cooperatives	138
Rio de Janeiro	Brazil	0.09	2015	Plausible	Bulk of the recycling is via the informal recycling sector, with formal efforts only at a very small scale – the 0.09% is therefore plausible	95,139
Porto Alegre	Brazil	3.43	2015	Unlikely	Evidence of a strong informal sector. Though there is an indication in the reference that some formal recyclates are collected, however it doesn't appear enough to justify the 3.42% stated.	140
Camagüey	Cuba	3.86	2017	Plausible	Evidence of both government sanctioned and organised recycling and sloe buy-back centres commensurate with a relatively low recycling rate as reported	141
Quito	Ecuador	0.86	2012	Plausible	Evidence that formal recycling takes place and will increase in the future, but also evidence of a strong informal sector across Ecuador. Given the low proportion, too low to represent a large informal sector, it is suggested here that the data represent formal operations rather than informal	142-144

Municipality	Country	Reported recycling rate (% of collected waste)	Year	Plausibility	Reason	Reference
Cuenca	Ecuador	0.50	2012	Plausible	Evidence of Bring sites in the municipality but not formal collection by municipality – the very low rate reported indicates it cannot be the informal sector as too low	145
Tehran	Iran, Islamic Rep.	39.62	2017	Unlikely	References indicate that formal recycling is not carried out and that the informal sector is thriving	146,147
Mashhad	Iran, Islamic Rep.	13.14	2017	Unlikely	Though some evidence of formal recycling exists, it does not appear to be substantial enough to justify 13.14% - therefore this is assumed to be a mixture – but classed as 'unlikely' for this screening process	147,148
Esfahan	Iran, Islamic Rep.	6.72	2017	Plausible	Evidence of a type of mixed waste sorting facility – the mechanism for collection is unclear, but the rate reported is low enough for this to be plausible.	149
Astana	Kazakhstan	16.41	2019	Plausible	The national statistics bureau indicates an 10.9% recycling rate nationwide in 2019 and 20.5% in 2020 - in Astana, a waste and recycling programme was proposed in 2006, so it is plausible that it is functioning now	150,151
Almaty	Kazakhstan	10.21	2019	Plausible	Various government websites extol the countries efforts to recycle one of which repots a 23% recycling rate for Almaty – the rate of 10.21 appears plausible for formal recycling, if a little high for a municipality of 2 million	152
Tripoli	Lebanon	5.47	2012	Unlikely	Though some news articles have indicated that Lebanon has plans to introduce formal recycling and it appear it has been done in some institutions, there is no historical evidence for formal recycling but strong evidence of an informal sector and various charitable initiatives	153,154
Beirut	Lebanon	4.00	2012	Unlikely	Though some news articles have indicated that Lebanon has plans to introduce formal recycling and it appear it has been done in some institutions, there is no historical evidence for formal recycling but strong evidence of an informal sector and various charitable initiatives	104,153,154
Callao	Peru	1.14	2019	Unlikely	Callao Municipality publishes a register of private companies and cooperatives who are licensed to selectively collect waste. |It is suggested that the 1.14% reported equates to their activities as they can't be disaggregated and we consider the cooperatives to be informal, we have scored as 'unlikely'	155
Arequipa	Peru	1.14	2019	Unlikely	Evidence of a sorting station (Yanahuara Recycling Plant) that has been implemented to replace previous waste picker activity on the dumpsite. As they were previously informal workers we will classify as unlikely to be formal here	156
Lima	Peru	0.64	2019	Plausible	Evidence of some formal activity but still dominated by informal sector – some token bring banks are evident as the proportion is very low, it is suggested that it represents formal activities	157,158

Municipality	Country	Reported recycling rate (% of collected waste)	Year	Plausibility	Reason	Reference
Soweto	South Africa	9.82% x (1 – 0.238) = 7.48%	2017	Plausible	Evidence indicates that formal recycling takes place, though: **1)** It is only provided directly by the municipality in about 24% of cases on average across South Africa; and **2)** Only approximately 23% and 16% of the residents of Cape Town and Johannesburg respectively report that they separate material for recycling. These two basic assertions do not seem to justify the quantities reported (11.26%). Therefore, we surmise that the figures reported by UNSD for Soweto and Cape Town include both formal and informal collection. The evidence also includes an estimate that says 23.8% of waste is collected by itinerant buyers. We therefore deducted this proportion from the proportion recycled reported by UNSD approximate the proportion formally collected.	
Cape Town	South Africa	11.26% x (1 - 0.238) = 8.58%	2017	Plausible		159

Often the value for recycling was left blank by the user. In cases where the amounts recorded as going to treatment and disposal options were within 20% of the collected waste (or household collected waste if collected waste was not provided), it was assumed that all mass had been accounted for by the user and therefore this blank was treated as a zero.

S.6.4.4.4 Incineration

The amount of waste going to incineration is a data point in the UNSD waste data[33] along with a subset for the amount of that incineration with energy recovery. A similar approach was taken as with the recycling data point, whereby the incineration rate as a percentage of collected waste (tC2iii) was calculated first by dividing the mass reported incinerated by the mass reported as collected. In a small number of cases, the amount collected was reported as household collection instead of overall collection. In these instances, the incineration rate was calculated as the mass incinerated divided by the amounts collected from households. Lastly, if data on the amount collected were not reported, but data on the amount going to each facility were, it was assumed that the sum of the amount going to recovery and disposal facilities equalled the amount collected. This summed value was then used as the denominator in the calculation of the incineration rate.

In total, 67 records yielded an incineration rate, although only 21 of these reported a non-zero rate. However, analysis of the dataset suggested that some records of MSW incineration may have been because of a misclassification. For instance, small amounts of medical (hazardous waste), or waste that is open burned may have been included. As we were only interested in modelling full scale MSW incineration, we assessed the plausibility that incineration was actually taking place in each of these 21 cities by corroborating the assertion with other sources which we have detailed **Table S16**.

Table S16. Evidence that incineration takes places in the municipalities reported in UNSD city waste data[33].

Municipality	Country	Calculated incineration rate (% of collected waste)	Year	Plausibility	Reason	Reference
Baku	Azerbaijan	44.8	2019	Plausible	Evidence of incineration with energy recovery in Baku.	160
Thimphu	Bhutan	15.0	2017	Unlikely	No evidence of incineration of MSW, but there is for incineration of hazardous medical waste.	161,162
Gaborone	Botswana	0.4	2017	Unlikely	No evidence of incineration. Perhaps confused with open burning which is reported to occur.	135
Francistown	Botswana	0.3	2017	Unlikely		
Brasilia	Brazil	0.3	2009	Unlikely	No evidence of incineration in Brazil. Small percentages here may relate to hazardous waste incineration.	163
Rio de Janeiro	Brazil	0.02	2009	Unlikely		
Shanghai	China	65.6	2019	Plausible	Evidence of incineration for each city in national statistics.	34
Chongqing	China	50.6	2019	Plausible		
Beijing	China	48.9	2019	Plausible		
Macao	China, Macao Special Administrative Region	98.5	2015	Plausible	Evidence of incineration in Macao.	164
Zagrab	Croatia	0.1	2012	Unlikely	Evidence of incineration project being scrapped due to public opposition.	165
Cuenca	Ecuador	0.2	2011	Unlikely	No evidence of incineration. Small percentages here may relate to hazardous waste incineration.	120
Schaan	Liechtenstein	47.1	2019	Plausible	Although there are no incineration plants in Liechtenstein it is reported that much waste is exported to Switzerland for incineration, hence this is assumed plausible.	166
Monaco	Monaco	89.9	2017	Plausible	Original value reported exceeds 100%. It is believed this is a typo and the value of 130,000 tonnes/year was replaced with 30,000 tonnes/year. Regardless, there is evidence of widespread incineration with energy recovery in Monaco.	167
Yangon	Myanmar	1.9	2017	Plausible	Incineration plant opened in 2017 with plans to develop further.	168
Zinder	Niger	1.0	2006	Unlikely	No evidence of incineration. Small percentages here may relate to hazardous waste incineration.	120
Niamey	Niger	1.0	2006	Unlikely		
Kiev	Ukraine	13.8	2019	Plausible	As of 2013, one incineration plant was operation in Kiev although this reportedly incinerating only 1% of MSW in Kiev and was beyond its designed lifespan. It is plausible that this has since been upgraded.	169
Songea	Tanzania	0.8	2015	Unlikely	No evidence of incineration. Small values may represent hazardous waste incineration such as medical waste.	120
Moshi	Tanzania	0.2	2015	Unlikely		

Municipality	Country	Calculated incineration rate (% of collected waste)	Year	Plausibility	Reason	Reference
Kwekwe	Zimbabwe	7.9	2015	Unlikely	No evidence of incineration in 2015 although a plant has recently been approved.	170

Abbreviations: municipal solid waste (MSW).

As with formal recycling, blank values were treated as zero if the sum of the treated and disposed waste summed to within 20% of the collected waste.

S.6.4.4.5 Other recovery

The primary data input 'formal collection of MSW for other recovery' (tC2ii) is composed of two categories from the UNSD waste data, namely 'composting' and 'other treatment methods'. The overall recovery rate as a percentage of collected was first calculated in the same manner as that for incineration. The collected waste was first prioritised as the denominator, followed by household collected waste, and lastly treated and disposed waste. Likewise, blank values were treated as zero if the sum of the treated and disposed waste summed to within 20% of the collected waste.

S.6.4.4.6 Controlled disposal

The definition for 'controlled landfill' in the UNSD waste questionnaire states '*final placement of waste into or onto the land in a controlled landfill site*'[40]. No clarification is provided on what constitutes 'control'. As such, a respondent's decision about whether a disposal site is controlled is likely to be subjective and cannot be directly correlated with the definition used in the present work. In the absence of this clear definition, given the use explicit use of term 'controlled', we assumed that the definition for controlled landfill provided in the UNSD dataset matches that used in the present work.

The proportion of waste collected for disposal that is sent for controlled disposal (tC3) was calculated by dividing 'controlled landfill' by total 'landfill', provided that the sum of the mass going to treatment and disposal facilities was within ± 20% of the mass of collected waste (n = 113). As before, due to the incorrect assignment of values to household collected waste instead of total collected waste by some respondents, 'controlled disposal' was also calculated using the 'household collected waste' as the denominator. This was only used if the previous method was not available (n = 7). This gave 120 records for controlled disposal (tC3) from the UNSD dataset.

If a value for 'landfill' was provided but the value for 'controlled landfill' was left blank by the user, it was assumed that no waste was assigned to 'controlled landfill' and therefore set as zero.

S.6.4.5 SIPSN Data

Municipal level solid waste management data for Indonesia is recorded as part of a national dataset entitled 'Sistem Informasi Pengelolaan Sampah Nasional'[35], hereafter referred to as SIPSN. Data is recorded at the municipality / Regency level of which there are 514 in Indonesia; however, not all of these have data available. Data for the year 2020 was used in this analysis.

The mass of waste generated in tonnes per day is directly recorded in SIPSN. This was converted to a per capita waste generation rate by dividing by the population of the Regency as obtained from the 2020 BPS census[171].

Collection coverage is not reported in the SIPSN data. This may be due to the highly decentralised nature of waste collection in Indonesia meaning collection of waste and transport to transfer stations (*TPS*) is the responsibility of neighbourhood associations (*Ruken Warga*)[172,173]. Despite this, the SIPSN dataset records the amount of waste entering disposal sites (*TPA*) and the amounts recovered at transfer stations with material recovery facilities (*TPS3R*). The collection coverage was therefore estimated for each Regency by summing the amount of waste entering disposal sites with the amount of waste recovered at TPS3R sites, before dividing by the reported mass of waste generation.

To avoid double counting, the recovered mass at TPS3R sites did not include any residual waste that would later be transferred to disposal sites. The SIPSN dataset reports the mass of recyclables collected by informal recyclers at disposal sites. This was subtracted from the mass collected, as informal recycling collection is modelled within this work and added on as part of the *Full MSW MFA*. Again this avoided any double counting.

The mass of recyclate recovered by the formal sector was calculated from the SIPSN data by summing the amounts of ‘dry recycling’ recovered at TPS3R’s by the formal sector with the mass of ‘inert recovery’ recorded at the disposal sites. We chose this summation on the basis that it would be closest to the way that formal recycling is reported in the other datasets (for example: WaW2.0 and UNSD). Informal sector recovery at the disposal sites and ‘organic recovery’ are recorded as separate data points in the SIPSN dataset, therefore it can be assumed that the summed values reflect that of formal dry recycling only. The calculated mass of recyclate recovered by the formal was divided by the mass of collected waste to give the formal dry recycling rate as a percentage of formally collected waste (tC2i).

Similarly, the primary data input for formal collection of MSW for other recovery (tC2ii) was calculated by summing the mass of ‘composting’ occurring at TPS3R’s with the mass of ‘organic recovery’ at the disposal sites, before dividing this by the mass of collected waste.

The composition of MSW is not provided in the SIPSN waste dataset, therefore the primary data input ‘plastic in MSW’ (C0) was unable to be calculated. Small amounts of waste were reported to be processed using ‘waste-to-energy’ in 37 municipalities in the SIPSN. We assumed that all of these were misclassifications as Terzidis[120] reported no operational large scale MSW incinerators in Indonesia.

The level of environmental control at the disposal sites is reported by the SIPSN data according to three categories: ‘sanitary landfill’, ‘controlled landfill’ and ‘open dumping’. It is unclear how these categories are defined, with it perhaps being subjective to the respondent. The definition for controlled disposal of MSW (tC3) used in the present work is ‘basic’, ‘improved’, or ‘full control’ according to the ‘Ladder of control level for landfill sites’ in the Waste Wise Cities Tool[6]. This states that to achieve the status of basic control, amongst other things the site must have a functioning weighbridge in use and have perimeter drainage maintained around the site. The SIPSN dataset details for each disposal site whether a weighbridge is in use and whether the site has drainage, therefore this data was used to cross check the response provided. If the Regency recorded their disposal site as a ‘sanitary landfill’ or ‘controlled landfill’, but also stated

they did not have either a functioning weighbridge or perimeter drainage, then the disposal site class was downgraded to an uncontrolled site. If the disposal site was recorded as ‘open dumping’, this was automatically assigned uncontrolled, regardless of the presence of weighbridges or perimeter drainage, given the WaCT ladder of control also specifies a degree of cover is required for basic control. As such, a disposal site was only classified as controlled if it was recorded as a ‘sanitary landfill’ or ‘controlled landfill’ and had both a functioning weighbridge and perimeter drainage. The mass of waste going to controlled disposal sites in each regency was divided by the total mass of waste going to disposal to arrive at an estimate for tC3: controlled disposal as a percentage of disposed waste.

The entire SIPSN dataset was not used, but instead a sample (n = 10) was extracted to ensure Indonesia was not being overrepresented in the subsequent machine learning steps. Details of this procedure are described in **Section S.6.2**.

S.6.4.6 MoHURD Data

The Ministry of Housing and Rural Development (MoHURD) in China release an annual dataset entitled ‘Urban Construction Statistical Yearbook’[34]. The 2019 version of this was used in this analysis, specifically the data points relating to mass of waste collected and transported by each municipality and the masses incinerated. The other inputs required for this work were either not reported (collection coverage), were unreliable (controlled disposal), or do not feature sufficient distinction (cannot differentiate between recycling and composting).

S.6.4.6.1 Waste generation

To estimate the primary input of waste generation rate ($tP1_{pc}$) the mass collected and transported was used as a starting point. However, this does not include waste that was generated and not collected, and therefore required correction by dividing by the collection coverage. Given the collection coverage is not a variable specified in the MoHURD dataset, an alternative approach was used for this correction. Initially, the collection coverage was estimated for each municipality based on the machine learning random forest process outlined in **Section S.7**. The collected and transported mass were then divided by predicted collection coverages to arrive at an estimate of total waste generation. This could then be divided by the population of the municipality as reported in the MoHURD dataset to arrive at a per capita waste generation rate.

S.6.4.6.2 Incineration

The percentage of collected waste that was incinerated (tC2iii) was derived by dividing the mass of waste going to incineration by the reported mass of waste collected and transported. In some cases, ambiguous administrative boundaries meant that it was difficult to assign incineration data to a specific GADM polygon. In these cases, the amount of waste reported as incinerated for the province was distributed amongst the polygons within it using its population.

The MoHURD dataset provided a full record of incineration for China, so we used these values directly in the probabilistic MFA, replacing any predictions from the machine learning steps (**Section S.9.1.2.7**). In contrast to the waste generation rate, a subset of the China incineration data was not randomly extracted from the from the MoHURD dataset for use in the machine learning steps (**Section S.6.2**). This was to avoid overly influencing (i.e., introduce bias) the training data with data for China, particularly given incineration in other UMCs is uncommon.

S.6.5 Data consolidation and deduplication

Following the initial data collection, harmonisation, correction, and preliminary screening phase described in **Section S.6.4**, data were combined into a single dataset with 691 municipal records. Each data record included:

- A unique data ID, linking the record to the source dataset
- Country name and ISO3 code
- Income category of the country for the year of the data record
- Name of the municipality (as per the original dataset)
- A unique administrative area ID identifying which GADM polygon the data record was assigned to (if any)
- GADM Level, administrative area match score, and any notes associated with the boundary matching

Data records also included one or more of the following:

- Waste generation rate ($tP1_{pc}$) and year (n = 582)
- Collection coverage (tC1) and year (n = 498)
- Plastic in MSW (C0) and year (n = 397)
- Rigid plastic (C0a) and year (n = 38)
- Formal dry recycling (tC2i) and year (n = 422)
- Other recovery (tC2ii) and year (n = 422)
- Incineration (tC2iii) and year (n = 441)
- Controlled disposal (tC3) and year (n = 458)
- SDG11.6.1 – MSW collected and managed in controlled facilities (n = 38)

Following consolidation, municipalities which were unable to be assigned a GADM boundary match (boundary match score of 4 as per **Table S6**) were removed from the analysis (n = 15). Likewise, data points older than 15 years (2006 at time of analysis) were also removed as it was assumed these data points were no longer relevant because waste management is likely to have changed substantially since then (n = 22). As an exception, a minority (n = 13) of WaW2.0 records older than 2006 were retained due to the underlying uncertainty around the year of data collection for data points other than waste generation rate (**Section S.6.4.3.7**) and to maximise data availability. All except one of these data records retained were post-2000.

95 municipalities had more than one record (n = 201) which either had to be merged or removed. Data were prioritised based on most recent year of data collection and dataset quality in the following quality assurance hierarchy 1) WaCT; 2) WABI; 3) WaW2.0; and 4) UNSD, the justification of which is detailed in **Table S4** (**Section S.6.1)**. Most recent data were selected first unless data from a higher quality data point was available within three years. If a record was missing a data point, then one from and older or lower quality dataset was used. Only one duplicate, Taian in China, existed for the records sampled from the national datasets. In this case, the MoHURD data were prioritised over that of the WABI dataset because the year was more recent. Records which were constructed from multiple data sources were given a new data id with prefix ‘CD’.

S.6.6 Default GADM Level selection

Of the 254 countries covered by the GADM dataset[1], 175 of these had at least one data record associated with it. The remaining 79 countries were mainly small countries and island states with small population or entirely uninhabited. Whilst these would be likely to have negligible impact on our global analysis, the lack of data indicates the need for data collection in less populous nations.

For the 175 countries with municipal level waste data, 134 had data records with a consistent GADM Level that had previously been assigned in **MS2** (**Section S.6.3**). In these cases, the consistent GADM Level was assigned as that country's municipal Level, described hereafter as the '*default GADM Level'*. Some countries (n = 41) had data records that were assigned to more than one GADM Level. In these cases, the *default GADM Level* was assigned as the Level for which the majority of that country's data records represented.

Data records that had been assigned a GADM Level that was more granular that the *default GADM Level* were removed from the analysis (n = 4), whereas data records at a less granular level were added alongside the *default GADM Level* by merging the underlying polygons (n = 39) **(Table S17).**

Additionally, a few records (n = 12) were allocated multiple GADM administrative boundaries at the same Level as this better matched the area for which the record represented (e.g., data for Melbourne was better represented by combining multiple Level 2 GADM polygons rather than choosing Level 1 which referred to the wider State). In these cases, the GADM polygons were merged into a single polygon and assigned the unique ID of the lowest numerical unique ID of the merged polygons along with the subscript 'Merged' to highlight changes had occurred compared to the original GADM dataset.

A small number (n = 22) of data records were allocated multiple GADM Levels because the administrative boundary was identical across different Levels. Typically, but not exclusively, this occurred for capital cities that have special administrative areas (e.g., cities that are both provinces and municipalities). In these cases, the data record was assigned the same Level as that of the *default GADM Level.*

Of the 79 countries for which no data existed, the majority of these countries (n=65) were small island states which had either no resident population, no subnational administrative divisions, or only a single subnational administrative division. The *default GADM Level* was therefore assigned for these as the most granular GADM Level available (either Level 0 or Level 1). The remaining countries without data were instead assigned the *default GADM Level* thought most likely to represent the municipal Level. All these allocations of *default GADM Levels* are documented in the **Supplementary Data 1** (cleaning, combining and deduplication steps).

A vector layer was created from the GADM dataset[1] that included the *default GADM Level* assigned for each country as well as the above modifications. In total this resulted in 50,702 *default GADM Level* polygons that represent the municipalities of the world (**Fig. S9**). The *default GADM Levels* varied from Level 0 (national Level) in the case of small island states, to Level 4 for the cases of Finland and Nepal.

Table S17. Municipal records which were assigned to a newly created merged polygon.

Country	Municipality	Default GADM Level	Data record Level	Unique ID of data point
Bangladesh	Dhaka	3	2	BGD.3.1_1
Bangladesh	Chittagong	3	2	BGD.2.4_1
Benin	Porto Novo	2	1	BEN.10_1
Bosnia and Herzegovina	Sarajevo	3	2	BIH.2.6_1
Burundi	Bujumbura	2	1	BDI.2_1
Cambodia	Sihanoukville	2	1	KHM.13_1
Cambodia	Phnom Penh	2	1	KHM.16_1
Cameroon	Douala	3	2	CMR.5.4_1
Cameroon	Yaounde	3	2	CMR.2.7_1
Canada	Vancouver	3	2	CAN.2.14_1
China	Lanzhou	3	2	CHN.5.7_1
China	Suzhou	3	2	CHN.15.7_1
China	Shanghai	3	2	CHN.24.1_1
China	Chongqing	3	2	CHN.3.1_1
China	Beijing	3	2	CHN.2.1_1
Cuba	Havana	2	1	CUB.4_1
Czech Republic	Prague	2	1	CZE.11_1
Egypt	Cairo	2	1	EGY.11_1
Egypt	Suez City	2	1	EGY.15_1
Ethiopia	Addis Ababa	3	2	ETH.1.1_1
France	Paris	3	2	FRA.8.3_1
Greece	Athens	3	2	GRC.3.1_1
Guatemala	Guatemala City	2	1	GTM.7_1
India	Chennai	3	2	IND.31.2_1
India	Greater Mumbai	3	2	IND.20.18_1
Indonesia	Jakarta	2	1	IDN.7_1
Mexico	Mexico City	2	1	MEX.9_1
Nigeria	Lagos	2	1	NGA.25_1
Pakistan	Karachi	3	2	PAK.8.2_1
Peru	Lima	3	2	PER.15.1_1
Peru	Callao	3	2	PER.7.1_1
Russia	Moscow	2	1	RUS.43_1
Rwanda	Kigali	2	1	RWA.5_1
Senegal	Dakar	4	1	SEN.1_1
Serbia	Belgrade	2	1	SRB.3_1
Slovakia	Bratislava	2	1	SVK.2_1
Tajikistan	Dushanbe	3	2	TJK.1.1_1
Tanzania	Dar es Salaam	2	1	TZA.2_1
Thailand	Bangkok	2	1	THA.3_1
Ukraine	Kiev	2	1	UKR.11_1
United Kingdom	London	3	2	GBR.1.36_1
Vietnam	Hanoi	2	1	VNM.27_1
Vietnam	Ho Chi Minh City	2	1	VNM.25_1

S.6.7 Data cleaning via outlier identification

Although initial data screening was performed on each individual dataset as described in **Section S.6.4**, this was primarily checking for obvious errors in the way the data was reported by users (e.g., wrong units) and making educated assumptions around what the data they reported was likely representing (plausibility checks). This section instead describes the checks applied to assess the reliability of the data via outlier identification, and, as such, was only performed once all the data had been combined into a single dataset.

Box and whiskers plots for each of the seven waste related *primary data variables* (**Fig. S10**) enabled visualisation of trends in the data and gave a first indication of potential outliers using the rule proposed by Tukey[85], which states that outliers are those data points which are more than 1.5 times the interquartile range distance from the 25th or 75th percentiles. However, this alone was deemed insufficient for potential outlier detection due to the data being often skewed. For example, waste generation rate is bound by zero therefore tends to have a long positive tail. Similarly, the dependent variables with units of percentages are bound between 0 and 100, therefore also tend to show either skewed distributions or bimodal distributions as many values fall at the limits. Setting outliers as 1.5 times the interquartile range in these situations often causes the whiskers to exceed the bounds of the data therefore failing to identify potential outliers. To overcome this, the fences as proposed by the 1.5 the interquartile range definition were used as guides along with expert opinion of the authors on what values should be crosschecked for potential implausibility. In general, the fences were set more conservatively than that proposed by the interquartile range rule, to ensure all potential outliers were screened for plausibility. This process was carried out for each dependent variable by income category of the country, with details of the fences used shown in **Table S18**.

Data points identified as potential outliers were not automatically removed from the dataset, but instead screened for plausibility (**Fig. S10**). This manual approach to removal of outliers was deemed preferential to automatic outlier removal as the global data was derived from many different socio-economic conditions, therefore one would expect some outlying values to be true values. Plausibility checks were based on expert opinion of the authors alongside as assessment of the data source reliability and context of the municipality that could be potentially resulting in an outlying value (e.g., tourism levels, whether it is a capital city or major commercial hub, and comparison to other values from that country).

Table S18. Fences based on expert opinion with values outside fences screened for plausibility.

ID	Primary data input	Unit	Country income category*	Total data points	Lower fence	Upper fence	Outlier cases below lower fence	Outlier cases above upper fence	Outlier cases removed for implausibility
$tP1_{pc}$	MSW generation rate	$kg \cdot cap^{-1} \cdot d^{-1}$	LIC	80	0.2	1.37	5	5	9 out of 10
			LMC	171	0.3	1.53	4	14	9 out of 18
			UMC	162	0.4	2.07	7	5	5 out of 12
			HIC	82	0.7	2.49	7	5	4 out of 12
tC1	Collection coverage	% of MSW generated	LIC	72	20	80	5	24	14 out of 29
			LMC	173	40	100	13	0	1 out of 13
			UMC	111	70	100	14	0	11 out of 14
			HIC	55	100	100	9	0	3 out of 9
tC2i	Formal collection of MSW for dry recycling	% wt. of formally collected MSW	LIC	65	0	0	0	1	0 out of 1
			LMC	131	0	5	0	0	0 out of 0
			UMC	97	0	5	0	13	0 out of 13
			HIC	71	0	50	0	6	6 out of 6
tC2ii	Formal collection of MSW for other recovery	% wt. of formally collected MSW	LIC	65	0	10	0	1	0 out of 1
			LMC	133	0	15	0	26	11 out of 26
			UMC	97	0	20	0	4	3 out of 4
			HIC	66	0	20	0	5	0 out of 5
tC2iii	Formal collection of MSW for incineration	% wt. of formally collected MSW	LIC	68	0	0	0	1	0 out of 1
			LMC	138	0	0	0	1	0 out of 1
			UMC	104	0	0	0	11	1 out of 11
			HIC	68	0	0	0	26	2 out of 26
tC3	Controlled disposal of MSW	% wt. of formally collected MSW for disposal	LIC	68	0	0	0	13	3 out of 13
			LMC	155	0	50	0	48	7 out of 48
			UMC	106	50	100	47	0	4 out of 47
			HIC	66	100	100	22	0	18 out of 22
C0	Plastic in MSW	% wt. of MSW generated	LIC	69	3	20	7	4	4 out of 11
			LMC	133	3	25	13	1	10 out of 14
			UMC	87	5	25	7	2	4 out of 9
			HIC	69	5	25	6	4	7 out of 10

*Abbreviations: High-income country (HIC); upper-middle income country (UMC); lower-middle income country (LMC); low-income country (LIC).

The cleaning process resulted in the removal of 136 (35%) out of 386 outlier data points. Removal of these data points had minimal impact on the central values (mean and median) or quartiles of input data (**Fig. S10**). Combined with the non-outliers, there were 553 cleaned records (municipalities with data) and 2,688 individual data points. Although the 553 records represent only 1.1% of global municipalities, approximately 904 million people live in them based on 2015 populations. This represents 12.2% of the 2015 global population, with similar coverage levels spanning all four income categories (LIC: 12.0%, LMC: 11.4%, UMC: 13.5%, HIC: 11.2%). Records are distributed across 172 countries and many major cities, as shown in (**Fig. S9**). We are therefore confident that the data collected represents the most widespread and quality checked municipal level data on municipal solid waste management to date. A summary of the data collection and cleaning process is shown in **Fig. S11.**

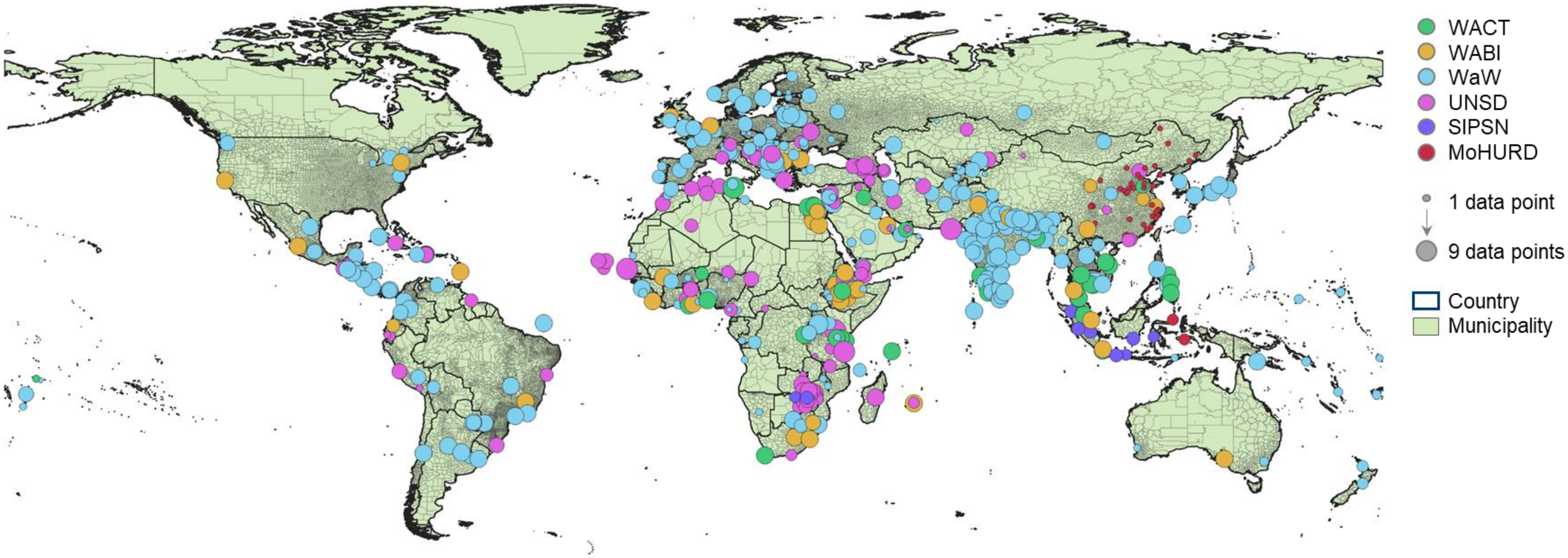

Fig. S9. Locations of *primary input data* by source dataset. Size of circles indicates number of data points in each location.

Fig. S10. Central tendency and spread of *primary data inputs* by country income category prior to outlier removal (red box plots) and post outlier removal (blue box plots). Dots represent outliers according to the 1.5 × interquartile range rule[85]. Crosses represent the mean value. The distribution of data as shown in the box plots was used to set fences around which outliers were identified and checked for plausibility (

Table S18). Abbreviations: high-income country (HIC); upper-middle income country (UMC); lower-middle income country (LMC); low-income country (LIC).

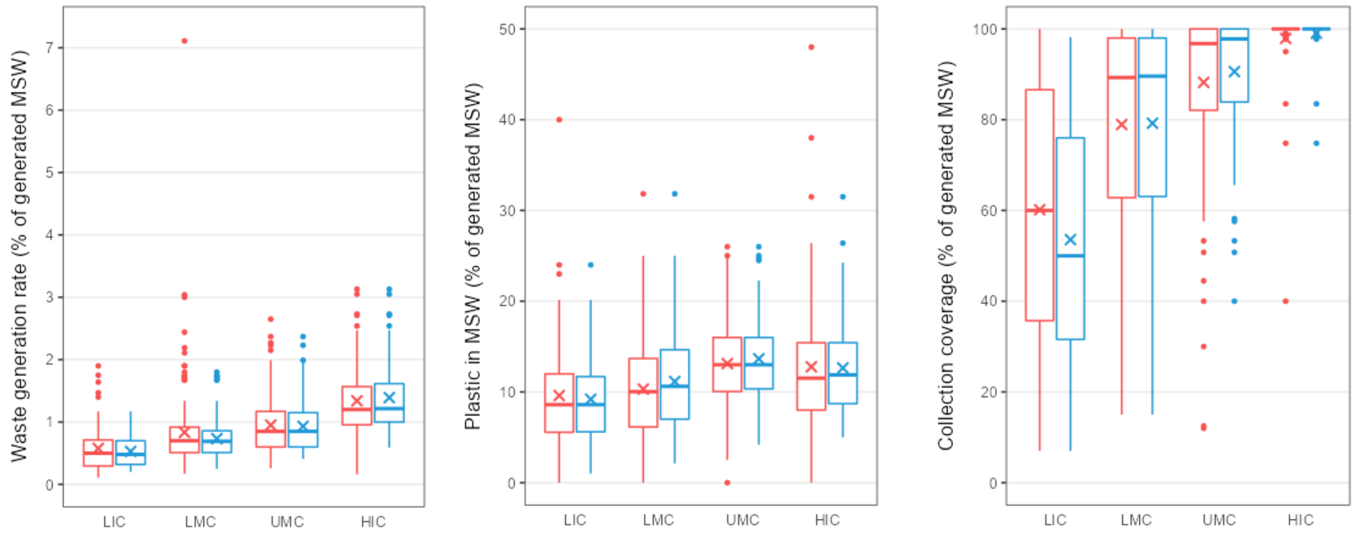

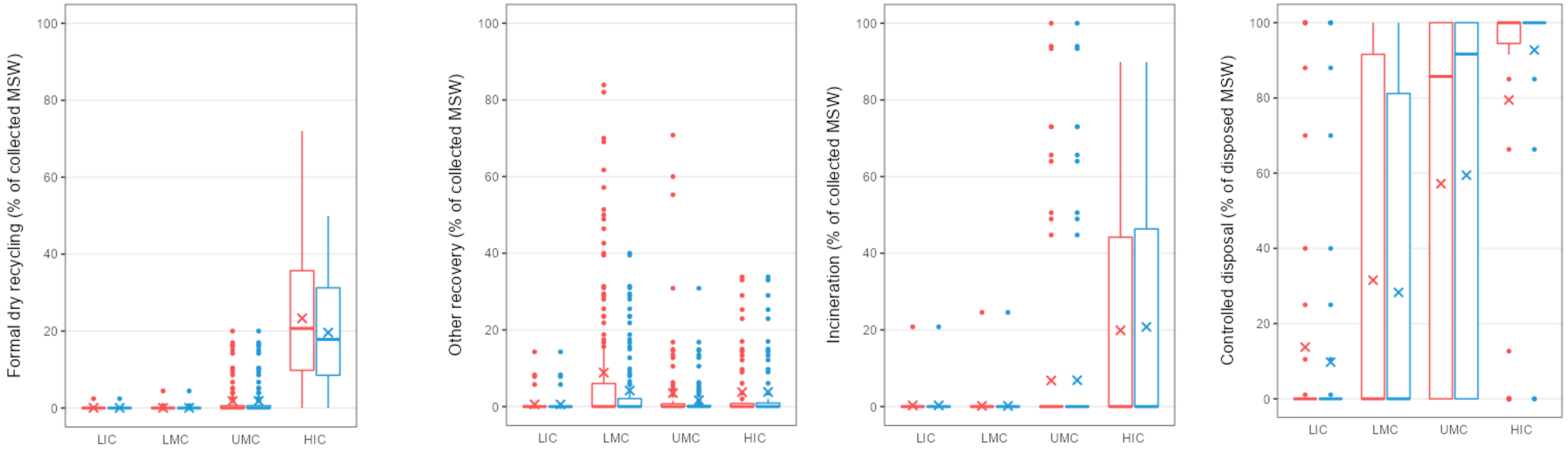

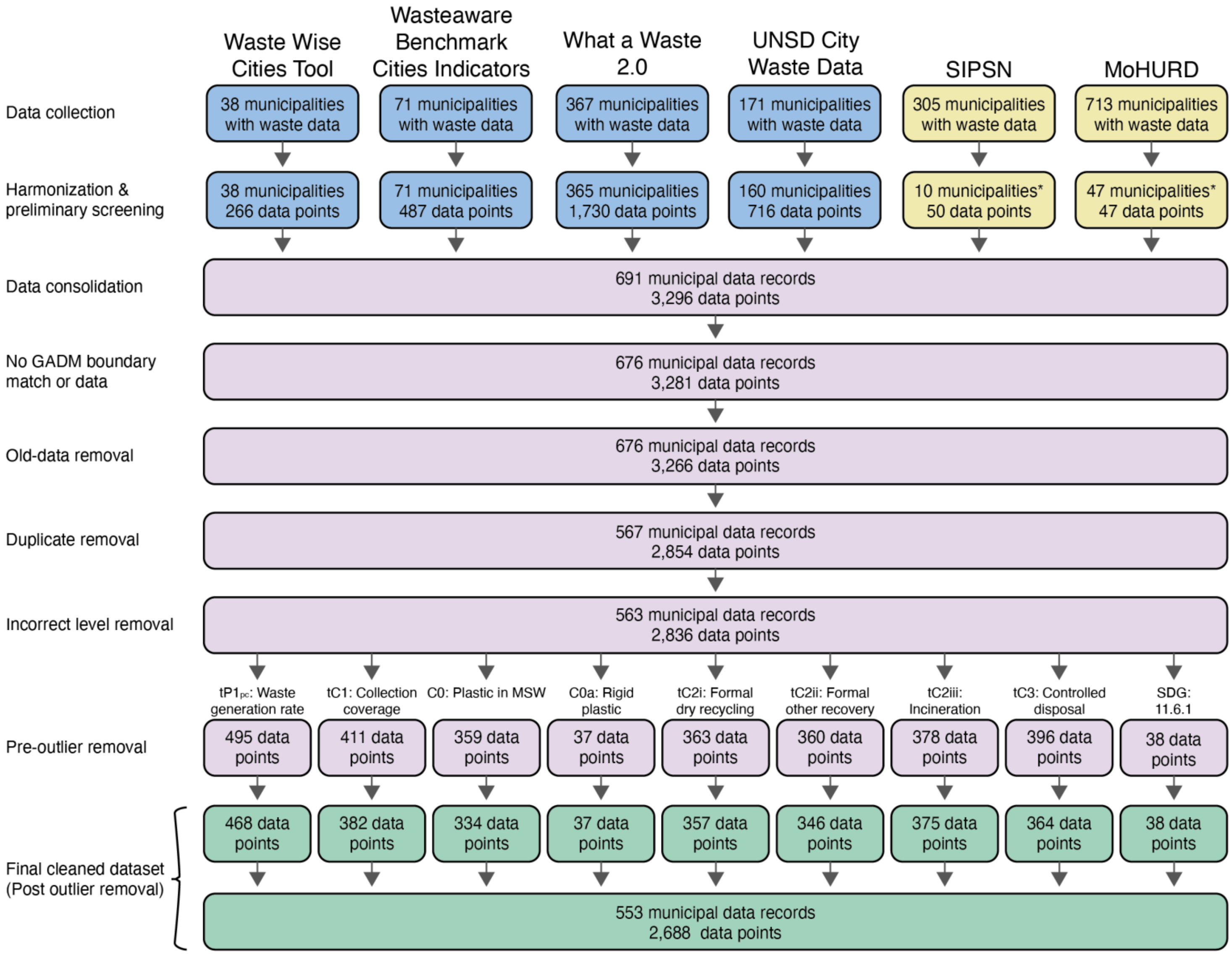

* Municipal records relate to a subset of sampled municipalities

Fig. S11. Summary of data collection, consolidation, and cleaning process. Blue and yellow boxes represent harmonisation and preliminary screening of the raw global and national datasets respectively; purple boxes represent cleaning steps following consolidation of data; and the green boxes represent the final cleaned dataset (**Supplementary Data 1**).

S.7 Machine learning for prediction of primary data input variables

We created a new machine learning model to predict data across all global municipalities using our cleaned dataset (**Supplementary Data 1**).

A commonly used method to estimate municipal solid waste management data is to base the prediction on the socioeconomics of the area. Waste generation rate is the most frequently estimated variable with several studies predicting global MSW generation at a country level using regression analysis and with gross domestic product (GDP) as the independent variable[22,31,174]. Others have expanded this further by using more sophisticated machine learning techniques (for example: artificial neural networks, supported vector machine, decision trees, gradient boosted regression trees, and K-nearest neighbours) to arrive at waste generation predictions, although these have so far been restricted to the national scale or below and often for forecasting time-series waste generation for a single location[175-181].

Aside from MSW generation and composition, very few studies have attempted to assess other aspects of municipal solid waste management performance that relate to the primary inputs in this work (i.e., collection coverage, levels of treatment and recovery, controlled disposal). Lebreton and Andrady[182] used country level data from Waste Atlas[183] (a database of user submitted waste management data, without quality control checks) alongside regression analysis to estimate global plastic waste generation and its mismanagement. ‘Mismanaged plastic waste’ was defined as the waste that goes to ‘unsound disposal’, plus 1% to account for littering. More recently, Velis, et al.[41] demonstrated that variability in cities waste management progress, as measured via Wasteaware Cities Benchmark Indicators, can be modelled by various socio-economic variables using both univariate non-linear regression and multivariate random forest approaches. The variables of waste generation rate, collection coverage, quality of collection services, controlled disposal and environmental protection tested by Velis, et al.[41] are highly relevant to the present work and therefore provide the justification that data gaps can be sufficiently estimated using socioeconomic data (indices) modelled through machine learning approaches.

S.7.1 Independent variables (MS4a)

Independent variables used for predicting gaps in the *primary data inputs* were initially selected based on those that Velis, et al.[41] had found to show high importance. To enable the in-country variability of solid waste management data to be described, sub-national independent variables were also sourced (**Table S19**) to ensure we had explanatory power across a range of economic, cultural, social, touristic, and geographic factors. We restricted our selection of independent variables for the random forest process to those which had near global coverage to minimise data gaps. With the exception of a few data points of independent variable highlighted in **Table S19**, we chose the nearest reference year for each variable to be as close to 2015 as possible because this is the median year of the cleaned *primary data inputs*.

A global spatial raster of population count data at 100 m resolution was sourced for the year 2020 from the Global Human Settlement Population dataset (GHS-POP)[184]. The zonal statistics tool in QGIS version 3.2.1 was used to sum the population count across each administrative area to calculate the 2020 population for each municipality. This was repeated for data from the year

2015 to assess historical populations of municipalities and allow comparison with the populations provided in older data records when performing the administrative area matching process (**Section S.6.3**). Although population was not used as an independent variable in the machine learning, it was still required to calculate other independent variables such as the number of international annual tourists as a percentage of national population.

Table S19. Independent variables and their properties.

Category	Variable	Unit	Format	Year	Type	Scale	Resolution	Ref.
Economic	GDP per capita	GDP per capita PPP in constant 2011 int. USD	Spatial raster	2015	Continuous	Global	Subnational (5 arc-min)	[185]
	Human development index (HDI)	-	Spatial raster	2015	Continuous	Global	Subnational (5 arc-min)	[185,186]
	Gross National Income (GNI) Per Capita, Atlas Method	Current US$	Excel	2015*	Continuous	Global	National	[187]
	Income category	-	Excel	2015	Categorical	Global	National	[86]
	Developing country	Y/N	Excel	2015	Categorical	Global	National	
	Small island developing country	Y/N	Excel	2015	Categorical	Global	National	
Demographic / Social / Cultural	Population density (unconstrained UN-adjusted)	People·km^{-2}	Spatial raster	2015	Continuous	Global	Subnational (30 arc seconds)	[188]
	Corruption Perceptions Index (CPI)	-	Excel	2015*	Continuous	Global	National	[189]
	Social Progress Index (SPI)	-	Excel	2015	Continuous	Global	National	[190]
Touristic	International tourist arrivals as % of population (calculated)	People	Excel	2015*	Continuous	Global	National	[191]
Geographic	Major city	Y/N	Spatial vector	NA	Categorical	Global	Subnational	[192]
	Sub-region	-	Excel	NA	Categorical	Global	National	[193]
	Degree of Urbanisation	-	Spatial vector	2015	Categorical	Global	Subnational (municipal level)	[194]

* Or nearest year to 2015 (up to three years away) if country data point not available for 2015.

We classified each default municipality to characterise its level of urbanisation according to the Global Human Settlement Global Degree of Urbanisation Classification of administrative units (GHS-DUC) methodology[195]. The GHS-DUC provides classification for administrative areas according to two levels. Level 1 includes three classes represented by a numeric ID: (1) rural; (2) town/semi-dense area; and (3) city. Level 2 includes eight classes: (30) city; (23) dense town; (22) semi-dense town; (21) suburban / peri-urban; (13) village; (12) dispersed rural area; (11) mostly uninhabited area; and (10) water.

The GHS-DUC is not available for GADM V3.6 (the version used here), so we applied the GHS-DU-TUC toolkit[194] to calculate urbanisation (for Level 1 and 2) for our own default municipality vectors using the GHS Settlement Model grid (GHS-SMOD)[196] and GHS-POP raster[184] for the years 2015 and 2020.

The Level 1 categorical classifications were used as an independent variable in our machine learning. The Level 2 classifications were used to calculate the proportion of the population that lives each settlement typology in each municipality using the GHS-DU-TUC toolkit[194]. The rural classes (10-13) were combined into a single 'Rural_share' category. The population in the Rural_share category and all of the other Level 2 classes were used to calculate street sweeping efficiency (**Section S.8.5.2**) and the Rural_share alone was used to correct data for rurality (**Section S.9.1.2**).

We also used several other sub-national independent variables to train the random forest model including: sub-national GDP per capita (PPP in constant 2011 international USD) and subnational human development index (HDI) for the latest available year of 2015 as per Kummu, et al.[185]. Additionally, sub-national HDI data was also obtained from Smits and Permanyer[186] for the year 2015 to fill any data gaps in Kummu, et al.[185]. Likewise, population density per km^2 for the year 2015 was further obtained from WorldPop[188]. Each of these independent variables was in raster form therefore the value for each municipality was summarised as the mean value, calculated using the QGIS zonal statistics tool.

Data on whether a municipality was a capital city, world city, or mega city was sourced from the Natural Earth populated places dataset[192]. These were aggregated into one overall indicator termed here 'major city' to reduce the number of independent variables and avoid overly correlated variables as this can impact the measure of variable importance via the permutation method[197].

In addition to the sub-national independent variables, national level independent variables were allocated to each municipality using their ISO3 country code[198] as detailed in **Table S19**. The international annual tourist arrivals were calculated as a percentage of the national population as determined from GHS-POP.

S.7.2 Imputation of independent variables (MS4b)

Occasionally, independent variables were not available for some administrative areas. At national level this was mainly because the World Bank does not recognise certain countries included in GADM (e.g., Taiwan, Kosovo), or does not report data for them (e.g., Small Island Developing States), but also because some data are not collated and published (e.g., international touristic arrivals). Any omissions in an independent variable were small, accounting for 2% of all administrative areas or less.

The random forest process described in **Section S.7.3** requires a complete set of independent variables with no data gaps. Therefore, missing values were imputed using predictive mean matching (pmm) method implemented with the R package '*MICE*' (version 3.14.0). We used the mean of five iterations, however when the imputed values for national level independent variables differed for the same country, we used the median to ensure consistency within a country.

S.7.3 Quantile regression random forest (MS5a and MS5b)

Random forest is a supervised machine learning method developed by Breiman[199]. A random forest is an ensemble of decision trees whereby each tree is grown from a bagged version of the

training dataset and the predictor variables used for splitting are selected at random at each node of the decision tree. In regression problems, the predictions are the average of the response of each tree, whereas in classification problems the majority result is taken.

Since its development, random forest has been used extensively for both classification and regression problems due to their wide suitability, simplicity, ability to deal with small sample sizes, minimal requirement for tuning and reduced risk of overfitting[199,200]. It has also recently been used for modelling solid waste management indices by Velis, et al.[41] who found that it outperformed non-linear regression models in all but one indicator.

Potential drawbacks of random forest regression are that they can be computational demanding; do not allow for extrapolation outside of the training data range; that variable importance metrics can be unreliable when dealing with highly correlated predictors; and that important information on the distribution of responses is neglected when the mean value of responses is taken[201-203]. To overcome this last disadvantage, Meinshausen[201] developed a variant of the random forest model originally presented by Breiman[199] whereby the value of all responses is retained, rather than just the mean. Termed 'quantile regression forests', the comprehensive retention of this information allows the distribution of responses to be expressed as quantiles, and therefore the uncertainty around predictions quantified. Quantification of uncertainty around *primary input* data predictions was used in this work by feeding it into the Monte Carlo probabilistic material flow analysis (**Section S.9**).

We implemented quantile regression random forest independently for each of the seven *primary input variables* in R using the package 'caret' (version 6.0-92). Twelve imputed independent variables shown in **Table S19** were used as the predictor variables. Hyperparameters of the random forest process include the number of trees in the forest (*ntree*), the number of input features to randomly sample at each split (*mtry*) and the minimum number of observations in a terminal node (*min.node.size)*. Probst, et al.[204] performed a literature review on the impact of these parameters on the performance of random forest and concluded that *mtry* is the most important parameter to tune, whereas *ntree* should be set high, but has diminishing value as more trees are added.

To limit potential overfitting and reliably estimate the predictive ability of the random forest models, the dataset was initially split into a training and test dataset (80:20) using the *caret* function *createDataPartition*. Training data was then used to tune the hyperparameters using grid search with 10-fold cross validation and five repeats. Hyperparameters tested were *mtry* between 1 and 12 (the maximum number of predictors), and *min.node.size* between 5 and 10. The number of trees *ntree* was kept constant at the default of 500 trees. Suitability of the random forest models in the tuning process were assessed by calculating the root mean squared error (RMSE), with the optimal model for each dependent variable chosen as the one where RMSE was minimised. The optimised model was then used to predict the unseen test dataset and again the RMSE was calculated. Similar values of RMSE between the cross-validation and testing data signified that the model was not overfitting (**Table S20**). Finally, once the error and overfitting checks were considered acceptable, the random forest model was retrained on the full dataset using the optimum hyperparameters. This process was repeated for each of the dependent *primary input variables*.

Table S20. Results of hyperparameter optimisation including optimum model parameters and root mean squared error (RMSE) values from cross-validation and testing on a holdout dataset.

			Optimum model parameters		Input data range		Cross validation	Test data
**ID**	**Variable**	**Unit**	**mtry**	**min.node.size**	**Min**	**Max**	**RMSE***	**RMSE**
$tP1_{pc}$	MSW generation rate	$kg \cdot cap^{-1} \cdot d^{-1}$	3	5	0.2	3.13	0.32	0.37
C0	Plastic in MSW	% wt. of MSW generated	1	7	1.0	31.8	4.78	5.29
tC1	Collection coverage	% wt. of MSW generated	4	5	7.0	100.0	15.47	13.84
tC2i	Formal collection of MSW for dry recycling	% wt. of formally collected MSW	2	10	0.0	49.9	6.07	5.95
tC2ii	Formal collection of MSW for other recovery	% wt. of formally collected MSW	1	5	0.0	40.0	6.46	5.26
tC2iii	Formal collection of MSW for incineration	% wt. of formally collected MSW	3	6	0.0	100.0	12.97	11.76
tC3	Controlled disposal of MSW	% wt. of formally collected MSW for disposal	2	7	0.0	100.0	35.38	34.92

* Of optimal model from cross-validation. Abbreviations: municipal solid waste (MSW).

The performance of random forest was assessed using the RMSE values presented in **Table S20**. Given RMSE has the same units as the dependent variable, the range of input data for each variable is also provided for comparison. Alternate metrics, such as the mean absolute percentage error (MAPE) or the symmetric mean absolute percentage error (SMAPE), were avoided because much of the data includes zeros, or values close to zero, and these metrics are known to become undefined or unstable respectively in these cases[205]. RMSE values were further compared to the RMSE values reported by Velis, et al.[41] for the comparable variables of waste generation rate (0.31 adjusted to $kg \cdot cap^{-1} \cdot d^{-1}$), collection coverage (10.17) and controlled disposal (27.96). The RMSE values in the present work are broadly comparable to those achieved by Velis, et al.[41], albeit slightly higher. It should be noted, however, that the Velis, et al.[41] analysed a limited dataset from a single primary data generating methodology (WABI), consisting of only 40 cities (maximum), and as such, their dataset was not tested on a holdout dataset and is therefore more at risk of overfitting. Likewise, the dataset used in this work is much larger than that used in Velis, et al.[41]. Although this is useful for improved learning by random forest, it is also likely to exhibit higher levels of noise, especially as it was collated from multiple sources (WaCT, WABI, WaW2.0, UNSD, SIPSN, MoHURD), despite efforts to compatibilize them (**Section S.6**).

The RMSE values presented in **Table S20** were considered acceptable for use in this work, especially given the wide range, noise and complexity of the waste management data that it predicts. Controlled disposal had the worst predictive capability with an RMSE of 35%, however, given its bimodal nature, the method for predicting controlled disposal was adapted to be treated as a classification problem rather than a regression one, as discussed in **Section S.9.1.1**.

Whilst the economic independent variables score highly for importance across all dependent variables, in many cases it is the social, cultural, or touristic independent variables that show the highest importance (**Fig. S12)**. This signifies that models that only use GDP or other economic metrics for prediction are perhaps excluding other important metrics.

Waste generation rate ($tP1_{pc}$)

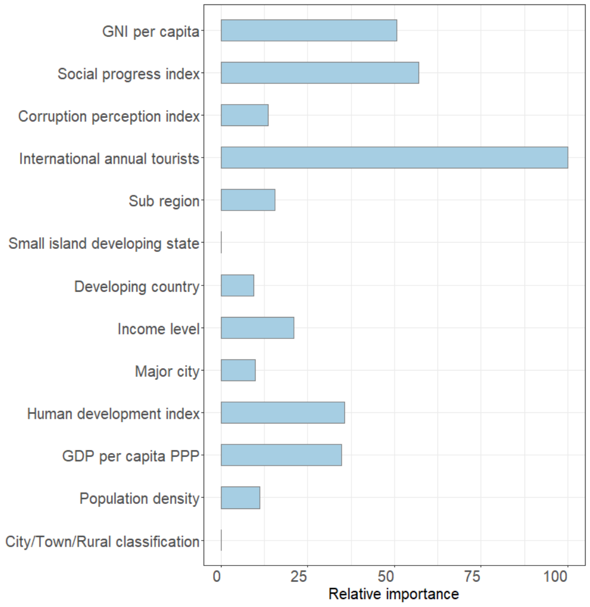

Plastic in MSW (C0)

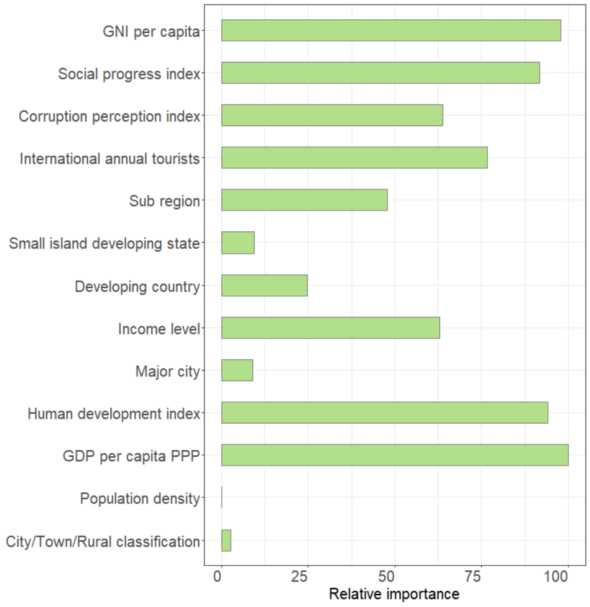

Collection coverage (tC1)

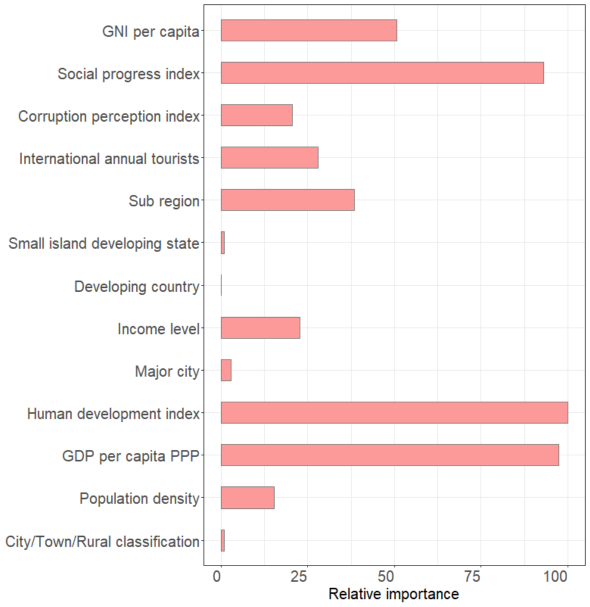

Formal dry recycling (tC2i)

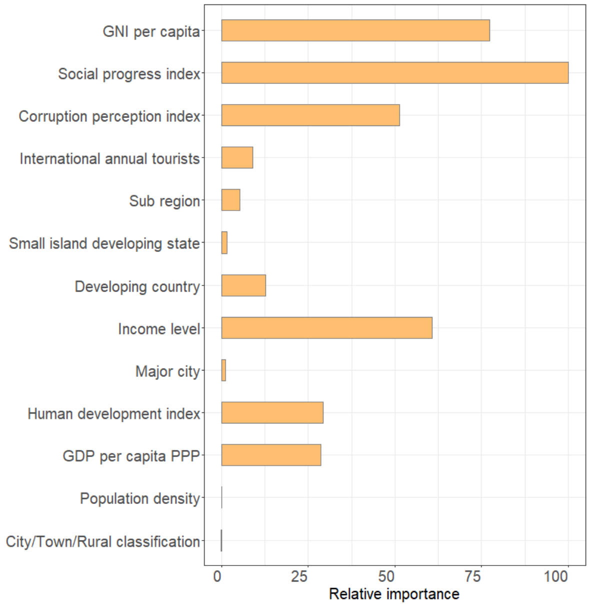

Other recovery (tC2ii)

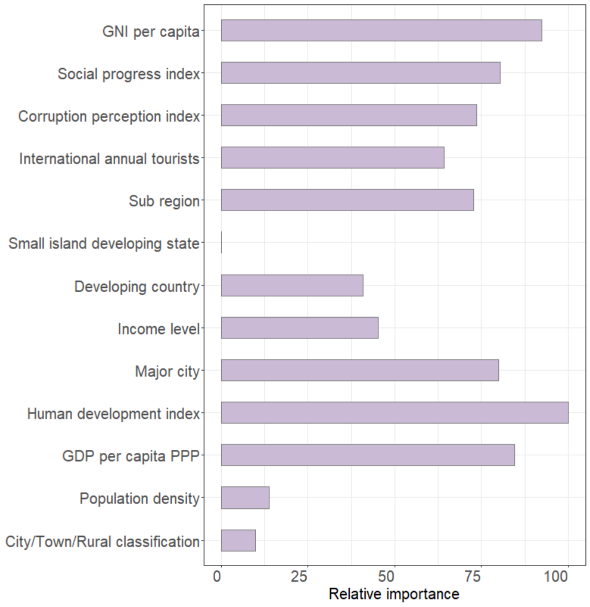

Incineration (tC2iii)

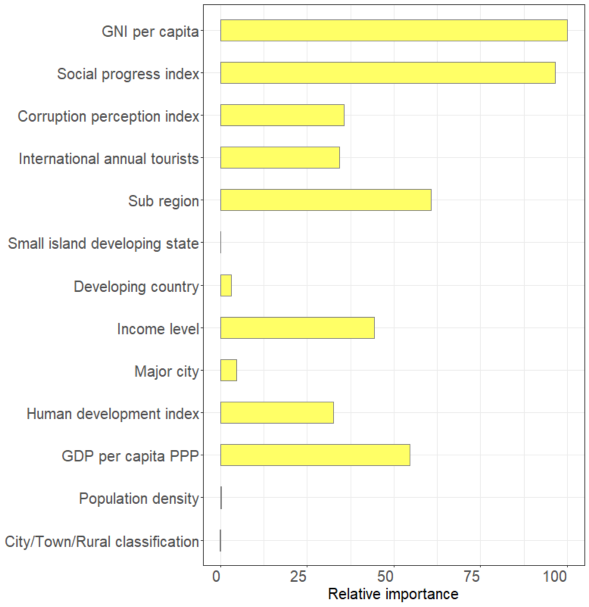

Controlled disposal (tC3)

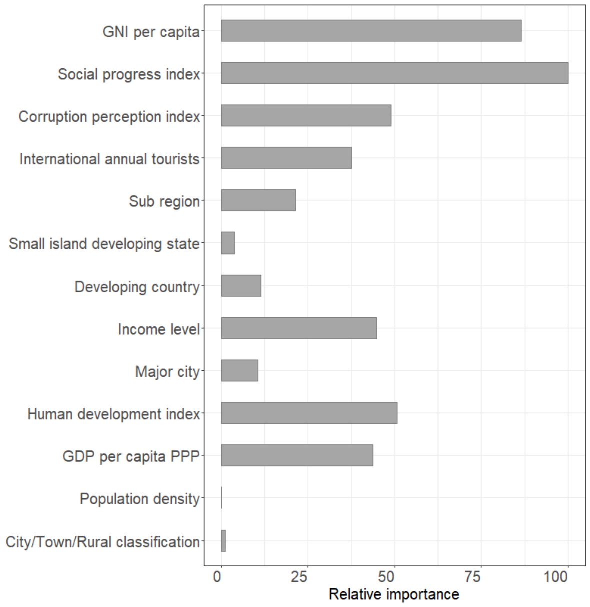

Fig. S12. Relative importance measure for each dependent variable as determined through the permutation method in quantile regression random forest.

S.8 Secondary data collection and processing (MS6)

In addition to the *primary data inputs* used to populate the *Tributary MFA*, secondary data was required to complete the more detailed *Full MSW MFA* and *Plastics MFA*. These secondary inputs build upon the *Tributary MFA* and enable three key areas to be explored in more detail, namely:

1. Converting MSW flows to plastic and rigid plastic flows at the *Tributary MFA* system ends.
2. Allowing further description of the formal and informal recycling processes.
3. Estimating emissions of plastic into the environment at specific parts of the system, including both debris emissions and open burning emissions.

Municipalities rarely report on the *secondary data inputs*, and in some cases, such as emissions of plastic from different parts of the solid waste management system, no reliably measured data yet exists. These data limitations mean that it was not possible to collate a database of *secondary data inputs* per municipality as done with the *primary data inputs*. Instead, available data is summarised either by archetypes (e.g., based on the income category of the country), or by modelling approaches.

Material flow analysis calculations in this work used a probabilistic approach based on Monte Carlo Analysis (**Section S.9**). This relies on the variability of each data input being specified in the form of a probability density function (PDF). Quantile regression random forest enabled the *primary data inputs* to be specified as PDFs (**Section S.7.3**), however, for the *secondary data inputs* different approaches were used, as detailed in **Sections S.8.1 - S.8.5.2**. All PDFs used in our model are available in **Supplementary Data 6** (**MFA Inputs**).

S.8.1 Proportion of plastic that is rigid (C0a)

The ratio of rigid to flexible plastic at different points of the system helps to determine the probability of material being emitted from different system components through the action of wind and surface water and in subsequent terrestrial transport models. In the absence of reliable measured data, we assume that the ratio of rigid to flexible plastic in waste generated is equivalent to C12a, C13a, C17a, C18a and C22a. For LICs, LMCs, and UMCs, the WaCT[30] provides verifiable, quality checked data for 37 municipalities which we used to approximate these proportions as normal distributions (**Table S21**). Due to only four data points being available for LICs, these were combined with LMC data.

Table S21. Proportion of rigid format material in upper-middle (UMC) and lower-middle / low income (LMC / LIC) countries based on household surveys from WaCT[30].

Income category	Number of data points	Rigid plastic (% wt. of plastic generation)	
		**Mean**	**Standard deviation**
UMC	7	44.4	3.9
LMC / LIC	30	41.8	10.3

For HICs, we used a normal distribution based on the mean (61.7%) and standard deviation (8.7%) of composition data from five sources which reported on approximately the same basis (**Table S22**).

Table S22. Proportion of rigid and flexible format material in selected high-income countries.

Source	Geographical context	Data type	Method	Basis	Rigid (% wt.)	Flexible (% wt.)
Chruszcz[206]	Wales	Primary	Waste characterisation	MSW	63.6	36.4
Bridgwater, et al.[207]	England	Secondary	Synthesis	HH	64.0	36.0

Source	Geographical context	Data type	Method	Basis	Rigid (% wt.)	Flexible (% wt.)
Cascadia Consulting Group[208]	California	Primary	Waste characterisation	MSW*	60.9	39.1
BMK[209]	Austria	Secondary	Not stated	MSW*	72.0	28.0
Tetra Tech EBA Inc.[210]	Vancouver	Primary	Waste characterisation	MSW	48.1	51.9
			**Mean**		**61.7**	**38.3**
			**Median**		**63.6**	**36.4**
			**Standard deviation**		**8.7**	**8.7**

* Although it was not specifically described as municipal solid waste (MSW), we assumed it based on the context and narrative in the study report. Abbreviations: Municipal solid waste (MSW); household waste (HH).

S.8.2 Informal sector recycling (P14)

A sub-model was developed to estimate the amount of waste collected by the informal recycling sector (IRS) (P14) worldwide (**Fig. S13**), based on a two-stage process originally developed by Lau, et al.[5]: (1) Estimate the number of informal recyclers in each area; and (2) Estimate the productivity of those recyclers, and hence how much waste they collect and reclaim for recycling.

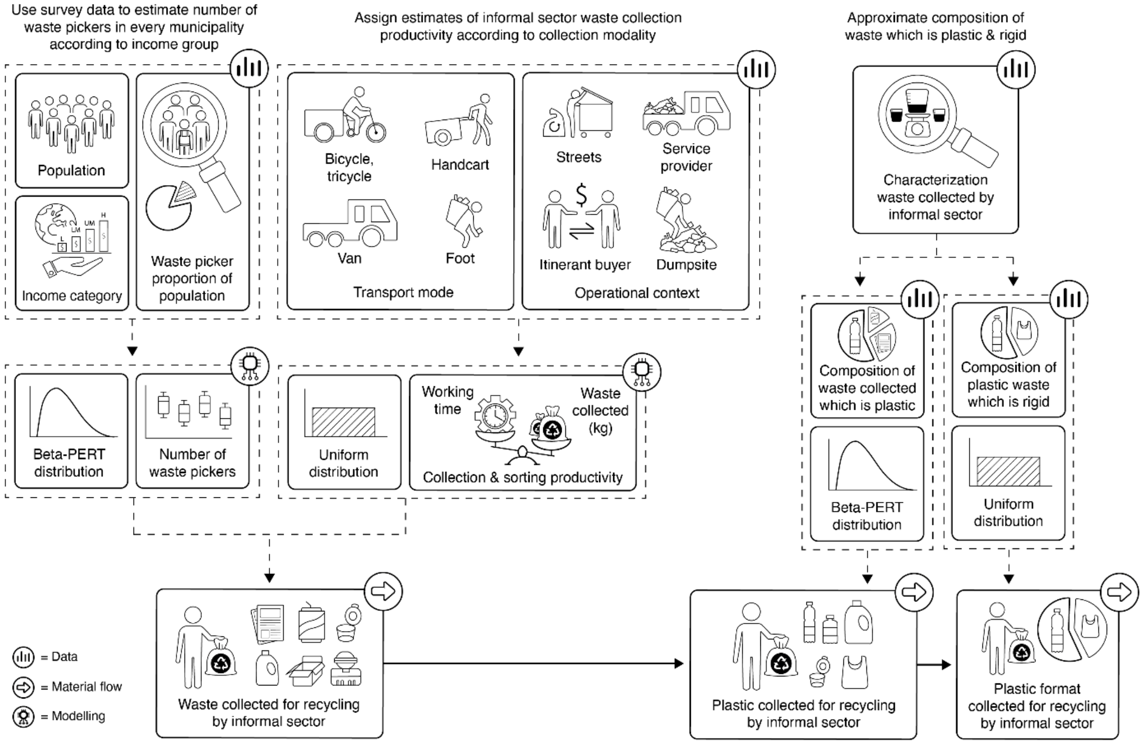

Fig. S13. Sub model used to estimate the quantity of plastic collected for recycling by the informal recycling sector.

Informal recycling sector population

Estimates for the proportion of informal recyclers in the urban populations of 102 municipalities and countries around the world were collated (**Table S23**) and categorised by World Bank income category (**Fig. S14**).

Table S23. Population engaged in informal waste collection as a proportion of total urban population in cities and countries.

ISO3	Country	Income category	Municipality	Proportion of waste pickers in urban population	Source
BRA	Brazil	UMC	Sorocaba	0.194	93
IDN	Indonesia	LMC	Jakarta	0.378	211
BRA	Brazil	UMC		0.192	212
BRA	Brazil	UMC	Esteio	0.186	213
ZAF	South Africa	UMC		0.136	214
PHL	Philippines	LMC	Metro Manila	0.156	215
PHL	Philippines	LMC	Quezon City	0.072	
ARG	Argentina	HIC	Rauch	0.233	
PAK	Pakistan	LMC	Lahore	0.188	216
PAK	Pakistan	LMC	Lahore (UC 16)	0.189	
IND	India	LMC	Tiruchirappalli	0.021	217
CHN	China	UMC	Urban Area	0.668	27
CHN	China	UMC	Beijing	1.373	
CHN	China	UMC	Guangzhou	1.159	
CHN	China	UMC	Shenzhen	2.179	
CHN	China	UMC	Suzhou	1.482	
CHN	China	UMC	Wuhan	0.262	
MNG	Mongolia	LMC	Ulaanbaatar	0.757	
IND	India	LMC	Urban Area	0.412	
IND	India	LMC	Ahmedabad	0.675	
IND	India	LMC	Amritsar	0.281	
IND	India	LMC	Bangalore	0.708	
IND	India	LMC	Delhi	1.280	
IND	India	LMC	Kanpur	0.615	
IND	India	LMC	Kolkata	0.511	
IND	India	LMC	Mumbai	0.694	
IND	India	LMC	Pune	0.248	
IDN	Indonesia	LMC	Bandung	0.133	
IDN	Indonesia	LMC	Jakarta	0.224	
PHL	Philippines	LMC	Manila	0.191	
PHL	Philippines	LMC	Quezon City	0.485	
BGD	Bangladesh	LMC	Dhaka	0.133	
PAK	Pakistan	LMC	Lahore and Allama Iqbal Town	0.333	
VNM	Vietnam	LMC	Ho Chi Minh City	0.338	
KHM	Cambodia	LMC	Phnom Penh	0.134	
MEX	Mexico	UMC	Mexico City	0.121	
MEX	Mexico	UMC	Monterrey	0.038	
PER	Peru	UMC	Urban Area	0.441	
PER	Peru	UMC	Callao	0.178	
PER	Peru	UMC	Canete	0.358	
PER	Peru	UMC	Lima	0.186	
BRA	Brazil	UMC	Urban Area	0.364	
BRA	Brazil	UMC	Belo Horizonte	0.157	
BRA	Brazil	UMC	Rio de Janeiro	1.301	

ISO3	Country	Income category	Municipality	Proportion of waste pickers in urban population	Source
BRA	Brazil	UMC	Santo Andre	0.303	
BRA	Brazil	UMC	Sao Paulo	0.177	
COL	Colombia	UMC	Bogota	0.252	
ARG	Argentina	HIC	Buenos Aires	0.222	
URY	Uruguay	HIC	Montevideo	0.907	
ETH	Ethiopia	LIC	Addis Ababa	0.204	
EGY	Egypt, Arab Rep.	LMC	Cairo	0.321	
TZA	Tanzania	LIC	Dar-es-Salaam	0.024	
ZMB	Zambia	LMC	Lusaka	0.039	
ROU	ROMANIA	UMC	Cluj-Napoca	1.044	
GHA	Ghana	LMC	Accra metropolitan area (GAMA)	0.031	218
MEX	Mexico	UMC	Monterrey	0.033	
MEX	Mexico	UMC	Guadalupe	0.087	
MEX	Mexico	UMC	San Nicolas	0.040	
MEX	Mexico	UMC	Mexico City	0.100	219
MEX	Mexico	UMC	Tultitlán	4.564	
MEX	Mexico	UMC	Nezahualcóyotl	0.055	
MEX	Mexico	UMC	Tultepec	0.026	
BRA	Brazil	UMC	Santo Andre	0.303	220
BRA	Brazil	UMC		0.114	221
SRB	Serbia	UMC		0.339	222
BRA	Brazil	UMC		0.303	223
MEX	Mexico	UMC	Celaya	0.422	224
CHL	Chile	HIC	Santiago de Chile	0.111	225
NIC	Nicaragua	LMC	Managua	0.117	226
GHA	Ghana	LMC	Kpone Katamanso District	0.143	227
IND	India	LMC	Mumbai	1.206	228
PAK	Pakistan	LMC	Halimar Town	0.037	229
PRY	Paraguay	UMC	Asunción	0.096	230
IND	India	LMC	Pune	0.028	231
PAK	Pakistan	LMC	Al Ima Iqbal Town	0.333	232
BGD	Bangladesh	LMC	Khulna	0.134	233
NGA	Nigeria	LMC	Lagos	0.063	234
EGY	Egypt, Arab Rep.	LMC	Cairo	0.227	
ROU	ROMANIA	UMC	Cluj	0.849	
PER	Peru	UMC	Lima	0.227	235
ZMB	Zambia	LMC	Lusaka	0.039	
IND	India	LMC	Pune	0.295	
PHL	Philippines	LMC	Quezon	0.406	
IDN	Indonesia	LMC	Bandung	0.129	236
COL	Colombia	UMC		0.290	237
VNM	Vietnam	LMC	Hanoi	0.136	238
IND	India	LMC	Kanpur	0.226	239
IND	India	LMC	Calcutta	0.167	
PHL	Philippines	LMC	Manila	0.128	240
MEX	Mexico	UMC	Mexico City	0.088	

ISO3	Country	Income category	Municipality	Proportion of waste pickers in urban population	Source
ZWE	Zimbabwe	LIC	Harare	0.084	241
ZWE	Zimbabwe	LIC	Bulawayo	0.296	
IND	India	LMC	New Delhi	0.106	242
BGD	Bangladesh	LMC	Dhaka	0.973	243
BRA	Brazil	UMC	Metropolitan region of São Paulo	0.094	244
IND	India	LMC		0.514	245
PHL	Philippines	LMC	Iloilo City	0.060	246
BGD	Bangladesh	LMC	Rajshahi City	0.156	247
CHN	China	UMC	Beijing-Haidian District (North)	0.757	248
CHN	China	UMC	Urban Area	0.668	249
CHN	China	UMC	Beijing (North)	0.073	250
CHN	China	UMC	Cities in China	0.455	251

We assumed a Beta-PERT distribution for the informal recycling sector population data with a default shape factor of four [252]. The shape factor controls the weighting of the most likely value. We chose the Beta-PERT distribution for two reasons: (1) Beta-PERT distributions require only three, easily obtainable parameters (minimum plausible value, most likely value, maximum plausible value), and are therefore suitable in situations where the available data are not sufficient to provide a more accurate distribution shape or when parameters rely on expert judgement; and (2) Beta-PERT distributions overcome some of the disadvantages of the triangular distribution, often favoured in such situations, because triangular distributions assign higher probabilities to the extremities of fat-tailed distributions[253].

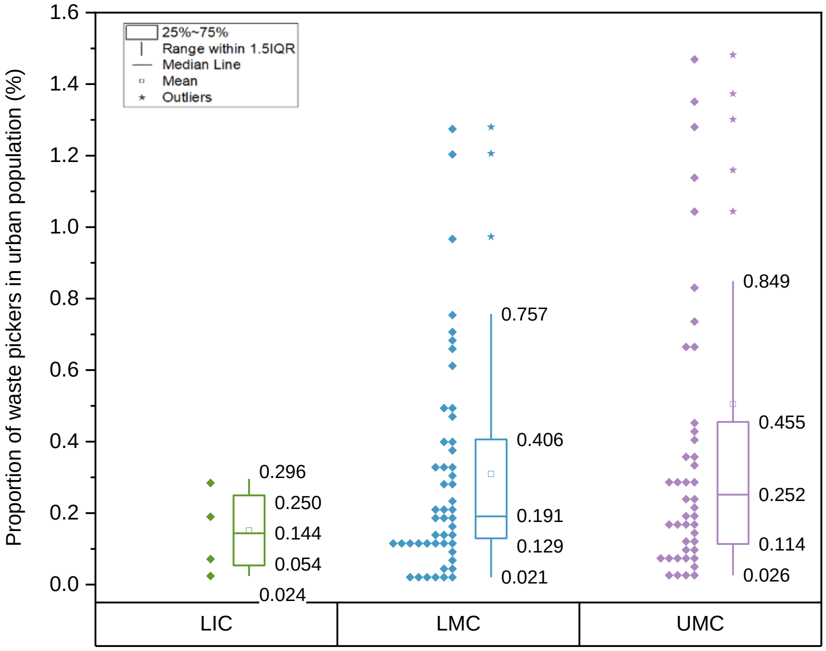

Fig. S14. Central tendency and spread of estimated proportion of waste pickers in municipalities and countries (n = 102).

Informal recycling sector population data were grouped by income category (**Fig. S14**). For the LICs, LMCs and UMCs, the most likely value was taken as the median, and the lower and upper plausible limits were taken as the range of values excluding outliers, defined as being greater than 1.5 times the inter-quartile range distance from each quartile. Four data points were available for HICs, all for countries in South America (Argentina, Chile and Uruguay) which, at the time the data were collected, had relatively recently entered the HIC category. For this reason, we considered that they are not necessarily representative of other countries in HICs, and therefore an assumption used by Lau, et al.[5] of mid-0.005% (range 0.0045-0.0055) was adopted.

S.8.2.1 Informal recycling sector productivity

Productivity data from 18 municipalities first reported by Lau, et al.[5] indicated a range of between 3.525-19.27 $t\cdot y^{-1}$ of waste (all types of recyclate) collected for recycling by selective collectors (**Fig. S15A**). This productivity data was converted to a PDF by assuming a uniform distribution. Multiplication of estimated number of waste pickers in a municipality with the expected productivity of each waste picker and a working year of 235 days, enabled the mass collected by the informal recycling sector to be approximated. This was undertaken within the probabilistic MFA detailed in **Section S.9** to incorporate the uncertainty as represented by the above PDFs.

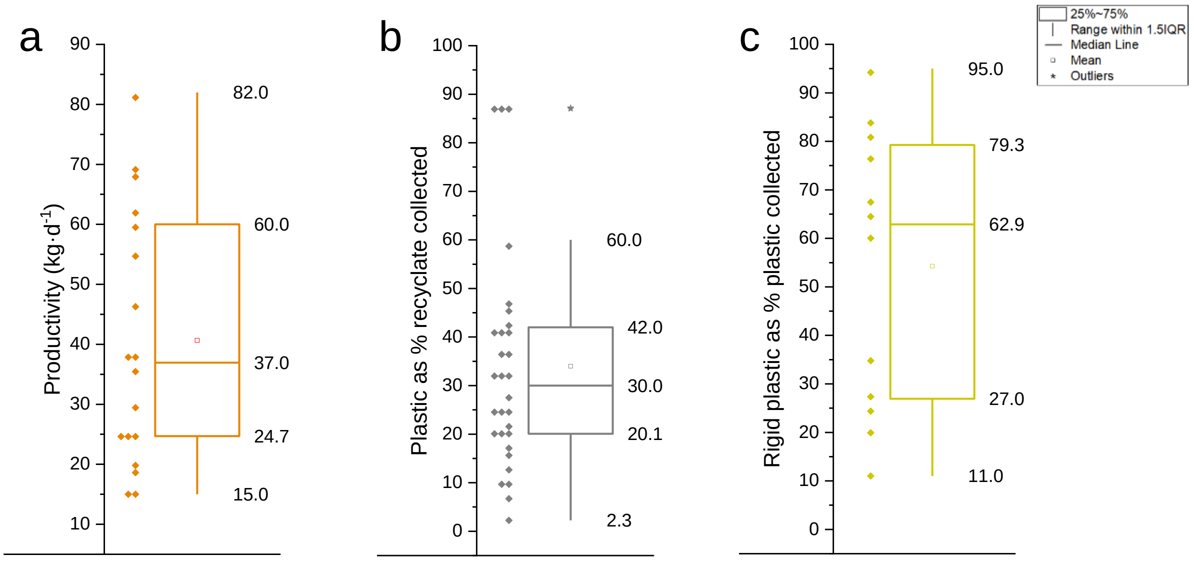

Fig. S15. Central tendency and spread of **(A)** daily productivity of informal recyclers in municipalities (n = 18); **(B)** proportion of waste collected by informal recyclers that is plastic (n = 29); and **(C)** proportion of plastic waste collected by informal recyclers that is rigid format.

S.8.2.2 Proportion of plastic collected by informal recycling sector (C15)

The proportion of waste collected by informal recyclers that was plastic (C15) in UMCs, LMCs, and LICs was based on 30 sources of data collected in 30 municipalities (**Table S24**). A Beta-Pert distribution was assumed with central value of 30% and a range of 2.3-60% (**Fig. S15B**).There is little data available on the proportion of plastic collected by informal recyclers in HICs where plastic recycling is driven by regulation and financial subsidies rather than unsupported market forces[254]. Financial incentives such as producer responsibility[255] are out of reach of informal recyclers and because they are light and have low value (by weight) relative to

the cost of living, we assume they are barely targeted if at all on a weight basis. Using a Beta-Pert distribution as with the Global South Countries, we chose the lower end of the range 2.3% as our central value, multiplied by 2 for the upper and of the range (4.6%) and a zero for the lower end.

Table S24. Plastic proportion of waste collected by informal recyclers.

Country	Municipality	Year data collected	Proportion waste collected by informal recyclers that is plastic (%)	Source
Brazil	Esteio	2017	20.76	213
Indonesia	Bantar Gebang	2014	87.00	256
India	Tiruchirappalli	2010	60.00	217
Brazil	Santa Rita	2012	32.80	257
India	Dhanbad	2018	43.00	179
South Africa	Johannesburg	2017	25.97	258
Egypt	Cairo	2016	13.00	259
Pakistan	Halimar Town	2015	32.00	229
India	Kanpur	2008	33.00	239
Cote d'Ivoire	Abdjan	2016	47.00	260
Bangladesh	Rajshahi City	2012	2.25	247
Brazil	Campinas	2013	24.80	261
China	Beijing-Haidian District (North)	2017	17.80	
China	Beijing-Haidian District (North)	2017	6.80	248
China	Beijing (North)	2010	10.50	262
Ecuador	Cuenca	2020	25.00	263
Ecuador	Cuenca	2019	22.10	264
Bolivia	La Paz	2020	20.70	265
Brazil	Belo Horizonte	2021	28.00	70
Brazil	Londrina, Parana state	2020	20.07	266
Brazil		2020	11.00	267
Indonesia	Bantar Gebang	2020	87.21	268
Ghana	Greater Accra Metropolitan Area	2023	87.12	60
Ecuador	Quito	2015	42.00	
Ecuador	Guayaquil	2015	42.00	
Ecuador	Cuenca	2015	37.00	
Ecuador	Manta	2015	46.00	
Ecuador	Average of 4 cities	2015	42.00	269
Nigeria	Abuja	2021	36.47	270
Brazil	Ribeirão Pires, São Paulo	2013	15.91	244

S.8.2.3 Proportion of plastic collected by informal recycling sector that is rigid (C21a)

The proportion of plastic collected by informal recyclers that is rigid (C21a) was based on 10 sources that presented data on 11 municipalities (**Table S25**). Due to the paucity of data and large spread, we were not confident to assign a central value and therefore chose a uniform distribution between the range 11-95% (**Fig. S15C**) for all countries.

Table S25. Proportion plastic waste collected by informal recyclers that is rigid.

Location of cohort (country)	Location of cohort (municipality)	Year of publication	Rigid (%)	Source
Indonesia	Bantar Gebang	2019	20.0	[271]
Indonesia	Jakarta	2018	95.0	[211]
Indonesia	Bantar Gebang	2014	11.0	[256]
India	Tiruchirappalli	2010	77.0	[217]
India	Dhanbad	2018	81.5	[179]
Pakistan	Halimar Town	2015	84.0	[229]
India	Kanpur	2008	60.6<br>28.2	[239]
Ecuador	Cuenca	2020	35.1	[263]
Ecuador	Cuenca	2019	65.2	[264]
Brazil	na	2020	67.9	[267]
Indonesia	Bantar Gebang	2020	25.7	[268]

S.8.3 Rejects of rigid and flexible plastic from sorting and reprocessing by formal (C24aa C24ab) and informal (C23aa, C23ab) sectors

We estimated plastic mass rejects (sometimes referred to in the literature as 'losses') at the sorting and reprocessing steps by creating a sub-model which used a set of logical assumptions about the economic value and recyclability of different polymers and formats. We used these to assign the probability that different types of plastic waste would be selected for recycling rather than screened for recovery or disposal. As summarised in **Fig. S16**, we applied these reject rates to baseline data for the amount of plastic waste collected for recycling in the Global North and South.

S.8.3.1 Step 1: Establish baseline plastic waste collected for recycling

The OECD provided us with polymer specific data on the amount of MSW plastic waste collected for recycling from their ENV-Linkages model ('Global Plastics Outlook'), which underlies a dataset that is published online in a summarised format[272]. Textiles were excluded for congruence with our model. We developed our assumptions according to three municipal categories: packaging; electrical and electronic; and consumer and institutional. Data for LDPE used in electrical and electronic equipment was excluded, because LDPE is rarely used in electrical and electronic equipment[273,274]. For simplification, we assumed that OECD members are HICs, which collect formally, and non-OECD countries are LMICs, which collect informally.

The ENV-Linkages model does not differentiate between flexible and rigid material collected for recycling. Therefore, we used European plastic packaging consumption data as a proxy, calculating the amount of flexible plastic consumed in each polymer category reported by Nonclercq[275] as a proportion of plastic consumption reported by Cimpan, et al.[276] (**Table S26**). Data to indicate the proportion of each polymer collected for recycling which is flexible were not available for LMICs. Therefore, we calculated a ratio between the mean proportion of flexible packaging for Europe (**Table S26**) and the median proportion of flexible material reported by WaCT data points. We applied this ratio to each of the proportions calculated for Europe.

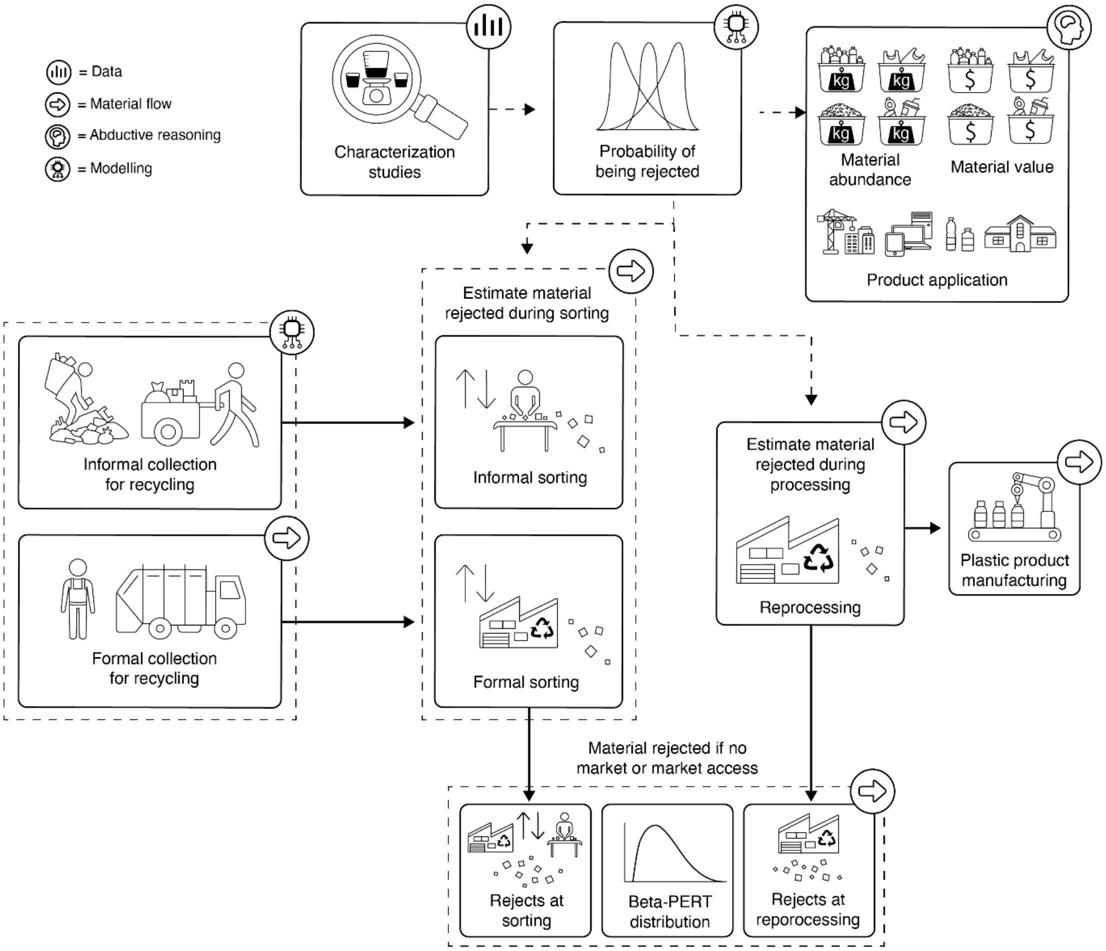

Fig. S16. Sub-model for estimating rejects (sometimes referred to in the literature as 'losses') (wt. as received (ar) reporting basis) from plastic waste that has been collected for recycling.

Table S26. Estimated flexible plastic packaging as a proportion of all plastic packaging.

**Polymer**	**Total consumption (Mt in 2014)[276]**	**Flexible consumption (Mt in 2014)[275]**	**Proportion of total plastic packaging that is flexible in HICs (%)**	**Proportion of total plastic packaging that is flexible in LMICs (%)**
HDPE	3.30	0.23	6.97	9.66
LDPE[a]	5.79	5.79	100.00	100.00
OTHER	1.37	0.24	17.50	24.25
PET	3.29	0.16	4.87	6.75
PP	3.78	0.88	23.31	32.30
PVC	0.38	0.08	20.79	28.82
Total	17.91	6.42	35.86	57.14

[a]LDPE includes LLDEPE. All flexible consumption was reported by Nonclercq[275] except LDPE which was all assumed to be flexible. Abbreviations: Million tonnes (Mt); high density polyethylene (HDPE); low density polyethylene (LDPE); polyethylene terephthalate (PET); polyvinyl chloride (PCV); polypropylene (PP); high income counties (HIC); low- and middle-income countries (LIMIC).

Polyurethane (PUR) collected for recycling is assumed to be used as bonding or coating and therefore rigid, except for in consumer and institutional category where it was assumed to be flexible and used as foam in mattresses and furniture[277]. We assumed that PVC collected under consumer and institutional was entirely rigid. We applied the proportions of flexible plastic packaging (**Table S26**) to the OECD polymer specific data for each category as shown in **Table S27**.

Table S27. Estimated mass of municipal solid waste plastic collected for recycling in high income countries and low-middle income countries based on MSW data underlying the ENV-Linkages model (‘Global Plastics Outlook’)[272]. Rigid and flexible plastics were estimated using European packaging data provided by Cimpan, et al.[276] and Nonclercq[275] as a proxy, as detailed in **Table S26**.

**Sector/ application**	**Plastic type by dominant polymer**	**Rigid & flexible mixed as reported**			**Rigid**	**Flexible**	**Rigid**	**Flexible**
		**HIC (Mt)**	**LMIC (Mt)**	**Total (Mt)**	**HIC (Mt)**		**LMIC (Mt)**	
Consumer & Institutional Products	HDPE	0.86	1.04	1.91	0.86	0.00	1.04	0.00
	LDPE, LLDPE	0.62	0.75	1.38	0.00	0.62	0.00	0.75
	Other	0.01	0.02	0.03	0.01	0.00	0.02	0.00
	PET	0.00	0.00	0.00	0.00	0.00	0.00	0.00
	PP	1.27	1.54	2.82	1.27	0.00	1.54	0.00
	PS	0.16	0.19	0.36	0.16	0.00	0.19	0.00
	PUR	0.07	0.08	0.15	0.00	0.07	0.00	0.08
	PVC	0.08	0.09	0.17	0.08	0.00	0.09	0.00
**Consumer & Institutional Products Total**		**3.08**	**3.72**	**6.80**	2.39	0.69	2.89	0.84
Electrical/ Electronic	HDPE	0.08	0.08	0.16	0.08	0.00	0.08	0.00
	LDPE, LLDPE	0.00	0.00	0.00	0.00	0.00	0.00	0.00
	Other	0.02	0.02	0.03	0.02	0.00	0.02	0.00
	PET	0.00	0.00	0.00	0.00	0.00	0.00	0.00
	PP	0.28	0.27	0.55	0.28	0.00	0.27	0.00
	PS	0.05	0.05	0.10	0.05	0.00	0.05	0.00
	PUR	0.03	0.03	0.05	0.03	0.00	0.03	0.00
	PVC	0.04	0.04	0.09	0.04	0.00	0.04	0.00
**Electrical/Electronic Total**		**0.51**	**0.49**	**1.00**	0.51	0.00	0.49	0.00
Packaging	HDPE	5.20	6.59	11.79	4.84	0.36	5.96	0.64
	LDPE, LLDPE	3.00	3.90	6.90	0.00	3.00	0.00	3.90
	Other	0.01	0.01	0.01	0.00	0.00	0.01	0.00
	PET	4.24	5.39	9.63	4.04	0.21	5.02	0.36
	PP	3.00	3.80	6.80	2.30	0.70	2.57	1.23
	PS	0.21	0.27	0.48	0.21	0.00	0.27	0.00
	PUR	0.01	0.02	0.03	0.01	0.00	0.02	0.00
	PVC	0.13	0.16	0.29	0.10	0.03	0.12	0.05
**Packaging Total**		**15.81**	**20.14**	**35.95**	11.51	4.30	13.96	6.18
**Grand total**		**19.40**	**24.35**	**43.75**	14.40	4.99	17.34	7.01

Abbreviations: Million tonnes (Mt); high density polyethylene (HDPE); low density polyethylene (LDPE); polyethylene terephthalate (PET); polyvinyl chloride (PCV); polypropylene (PP); high income counties (HIC); low- and middle-income countries (LIMIC).

S.8.3.2 Step 2 and 3: Identify empirical or assumptive data on rejects or use abductive reasoning to estimate

S.8.3.2.1 General assumptions, data, and abductive reasoning

We used a combination of empirical data, reported assumptions and abductive reasoning to estimate rejects at the sorting and reprocessing stages. For simplification of this step, plastic waste collected for recycling in LMICs was assumed to be collected exclusively by the informal sector, despite a few examples identified and discussed in **Section S.6.4.3.5** and **Section S.6.4.4.3**. We also simplify what is a complex continuum of processes into two basic stages of: 1) Sorting; and 2) Reprocessing, which would otherwise be overly challenging to model at global scale.

Table S28. Empirical data, assumptions and abductive reasoning underlying decisions made on the mass of rejects for material collected for recycling through formal and informal systems.

Sector / application	Formal collection, sorting	Informal collection and sorting
Packaging	**Assumptions**<br>• Predominantly collected alongside a mixture of non-plastics or, where collected separately, as a mixture of plastics.<br>• Almost never collected as separate stream except for LDPE wrap from commercial sources which is generally collected separately when collected for recycling.<br><br>**Reject rates applied**<br>• PS, PVC, PUR and 'other' plastics not separated for recycling therefore 100% rejects across sorting and reprocessing stages.<br>• Non-LDPE films not separated for recycling therefore 100% rejects across sorting and reprocessing stages.<br>• Reject rates at sorting stage for other plastics are mean reported by Antonopoulos, et al.[278] for European and UK materials recovery facilities:<br>o PET 19%<br>o PP 43%<br>o LDPE 42%<br>o HDPE 24%<br>Rejects at the reprocessing stage are based on analysis of data reported by Roosen, et al.[279], presented in **Section S.8.3.2.2**.	**Assumptions**<br>• Material is manually selected at the point of collection meaning that subsequent rejects are likely to be very small – waste pickers are unlikely to expend effort selecting and carrying substantial amounts of material that is not likely to return value.<br>• Therefore, rejects consist mainly of closures, plastic labels and some soiled material rejected by junkshops.<br><br>**Reject rates applied**<br>• As there are no published studies on this aspect of the informal sector, we assume informal sector rejects as twofold:<br>• 1) We used an assumption from Lau, et al.[5] that 5% of material collected for recycling by the informal sector is rejected during sorting; and<br>2) That rejects at the reprocessing stages are commensurate with analysis of data reported by Roosen, et al.[279] and Antonopoulos, et al.[278] presented in **Section S.8.3.2.2**.
Electrical & electronic	**Assumptions**<br>• The mass of plastic collected for recycling is part of the complex assemblies of items that constitute electrical and electronic equipment and cabling.<br>• Several sorting businesses now exist in Europe[280-283], and presumably elsewhere across HICs, but separation of plastics in these plants is commercially nascent.<br>• Sorting is predominantly by comminution and optical or electrostatic separation[284].<br>• Of the mass collected for recycling, only a very small proportion is likely to be recoverable for reprocessing due to its potentially hazardous characteristics, and the co-processing conditions which hinder purity[285,286].	**Assumptions**<br>• As with formal system, informal reclaimers are focused on the most valuable constituents of WEEE, the metals.<br>• There is some evidence that they recycle plastics in some locations[287], but in others they are simply burned due to lack of market access[288].<br>• Informal recyclers work harder to reclaim more material if it is technically possible. They are also likely to have less awareness of the hazardous nature of some WEEE plastics and therefore are less selective about which plastics to reclaim.<br><br>**Reject rates applied**<br>• PVC is mainly used in cabling in WEEE, and the informal sector is unlikely to strip and recover it due to the extensive time taken. Evidence suggests it is almost

Sector / application	Formal collection, sorting	Informal collection and sorting
	**Reject rates applied**<br>• On the basis of evidence that markets for secondary post-consumer PU and PS packaging are weak and that recovery rates are low when processed[278], we assume that recovery of PU and PS from WEEE are likely to be low or non-existent given that recovery from WEEE sources is more technically challenging. Therefore, we assume 100% reject rate at the sorting stage.<br>• In the absence of strong data, assuming that formal WEEE reclaimers have advanced conservatively in the previous decade, and that the majority of material is too contaminated to be recycled, we apply a 90% reject rate for sorting and reprocessing to all non-PUR and PS WEEE plastics.	always burned in open uncontrolled fires[289]. Therefore, we attribute a 100% reject rate for PVC at the sorting stage.<br>• On the basis that informal sector workers make more effort to recover less concentrated materials but that they have less technical capability to do so, we assume the following:<br>○ For PS and PU, 100% rejects at the sorting stage for the same reason as HICs.<br>○ For HDPE, PP and other plastics, recovery rates slightly higher than HICs of 85% across the sorting and reprocessing stages.
Consumer & institutional	**Assumptions**<br>• Items include all non-packaging plastics consumed domestically, commercially, and institutionally. Examples include toys, garden furniture, household and commercial furniture (i.e., all plastic items that are not electrical and electronic, part of a vehicle, packaging, used in agriculture, or part of a building construction).<br>• If recovered for recycling, these items are likely to exist in a format that is much larger than most packaging items.<br>• All material collected for recycling will be rigid format and many items and objects will be assemblies of items and materials.<br><br>**Reject rates applied**<br>• In the absence of any empirical data, we assumed the same reject rates as plastic packaging across the sorting and reprocessing stages for all materials except the following:<br>○ PUR is mostly collected in foam format as part of mattress collections. In many cases it is likely to be incinerated or landfilled, but there is strong evidence of recycling too, therefore we assign an assumption of 80% reject rate at the sorting stage.<br>○ PVC occurs in this category as furniture, often as a single, un-bonded or assembled material. Therefore, we suggest that the reject rates are relatively low and apply a 50% reject rate at the sorting stage.	**Assumptions**<br>• Unlike electrical and electronic waste, items in this category are unlikely to be collected for recycling unless the collector intends to recycle them. This is because they do not generally occur as bonded assemblies with other more valuable materials such as metals.<br><br>**Reject rates applied**<br>• For this category we apply the same rate of 5% at the sorting stage as for packaging.<br>• We applied rejects at the reprocessing stage using analysis of data reported by Roosen, et al.[279] and Antonopoulos, et al.[278] (for PVC and PS) presented in **Section S.8.3.2.2**.

Abbreviations: Million tonnes (Mt); high density polyethylene (HDPE); low density polyethylene (LDPE); polyethylene terephthalate (PET); polystyrene (PS); polyvinyl chloride (PCV); polypropylene (PP); polyurethane (PUR); waste electrical and electronic equipment (WEEE); high income counties (HIC); low- and middle-income countries (LIMIC).

S.8.3.2.2 Material rejects at reprocessors

Chemical and physical characterisation of plastic packaging item data reported by Roosen, et al.[279] was used to estimate potential rejects at reprocessors for rigid HDPE, PET and PP using a three step process: (1) We calculated the content of the target plastic component, meaning material targeted for recycling, as a proportion of total plastic (**Table S29**); (2) We deducted an assumed 1% process reject rate, to account for spillages and extrusion rejects (wastage); (3) We used the ratio of bottles to pots tubs and trays (excluding black plastics) reported in a weighted

compositional analysis of plastic packaging collected for recycling in the UK[290] to approximate the proportion of each, and hence weight the anticipated rejects during reprocessing (**Table S30**).

Table S29. Non-target (not targeted for recycling) plastics sampled at plastics reprocessors as a proportion of total plastics processed based on item characterisation reported by Roosen, et al.[279].

Item type	Target	Plastic residues	Non-plastic residues	As proportion of plastic excluding non-plastic residues		Reject rates adjusted for 1% wastage	
	**Mean**	**Mean**	**Mean**	**Target (%)**	**Residue (rejects) (%)**	**Target (%)**	**Residue (rejects) (%)**
PET bottle	81.60	11.60	6.80	87.55	12.45	86.55	13.45
PET tray	79.20	12.50	8.30	86.37	13.63	85.37	14.63
PE Bottle	77.50	13.60	8.90	85.07	14.93	84.07	15.93
PP Bottle	76.90	19.60	3.50	79.69	20.31	78.69	21.31
PP tray	91.30	1.00	7.70	98.92	1.08	97.92	2.08
Film	90.8		9.2	100.00	0.00	99.00	1.00

Abbreviations: polyethylene terephthalate (PET); polypropylene (PP); polyethylene (PE).

Table S30. Process of estimating the amount of material which is rejected for each item type listed in **Table S29** at the sorting and reprocessing stages according to typical ratio of bottles to pots, tubs and trays after Chruszcz and Reeve[290].

Dominant polymer	Item type	Colour	Composition reported by Chruszcz and Reeve[290] (%)	Normalised composition (%)	Assigned target rate (%)	Item descriptor from Roosen, et al.[279]	Reject rate per item type (%)
HDPE	Milk bottle	Natural	13.20	61.1	84.07	PE Bottle	9.734
HDPE	Non-milk bottles	Jazz	7.70	35.6	84.07	PE Bottle	5.678
HDPE	Pots, tubs & trays	Natural	0.10	0.5	97.92	PP tray	0.010
HDPE	Pots, tubs & trays	Jazz	0.60	2.8	97.92	PP tray	0.058
		**Total HDPE**	**21.60**	**100.0**		**Weighted average rejects HDPE**	**15.5**
PP	Bottles	Jazz	0.4	4.0	78.69	PP Bottle	0.844
PP	Pots, tubs & trays	Natural	4.4	43.6	97.92	PP tray	0.908
PP	Pots, tubs & trays	Jazz	5.3	52.5	97.92	PP tray	1.093
		**Total PP**	**10.1**	**100.0**		**Weighted average rejects PP**	**2.8**
PET	Bottles	Natural	26.4	65.5	86.55	PET bottle	8.809
PET	Bottles	Jazz	3.1	7.7	86.55	PET bottle	1.034
PET	Pots, tubs & trays	Natural	10.3	25.6	85.37	PET tray	3.740
PET	Pots, tubs & trays	Jazz	0.5	1.2	85.37	PET tray	0.182
		**Total PET**	**40.3**	**100.0**		**Weighted average rejects PET**	**13.8**

Abbreviations: High density polyethylene (HDPE); polyethylene terephthalate (PET); polypropylene (PP).

For PET film, HDPE film, PP film, rigid PS and rigid PVC, we used arithmetic mean reject rates reported by Antonopoulos, et al.[278] (**Table S35**). In the absence of better data, the reject rate for PUR and Other was assumed the same as PVC. We assumed the same reject rates at the reprocessing stage for materials collected for recycling by the formal and informal sectors. **Table S31.** Summary of plastic packaging reject rates at the reprocessing stage.

Plastic type by dominant polymer	Rigid		Flexible	
	**Reject rate (%)**	**Data source**	**Reject rate (%)**	**Data source**
HDPE	15.48	(**Table S30**)	29.00	[278]
LDPE, LLDPE			1.00	(**Table S29**)

Other	20.00	[278]	29.00	[278]
PET	13.76	(**Table S30**)	29.00	[278]
PP	2.84	(**Table S30**)	29.00	[278]
PS	34.00	[278]		
PUR	20.00	[278]		
PVC	20.00	[278]	29.00	[278]

Abbreviations: High density polyethylene (HDPE); low density polyethylene (LDPE); linear low-density polyethylene (LLDPE); polyethylene terephthalate (PET); polystyrene (PS); polyvinyl chloride (PCV); polypropylene (PP); polyurethane (PUR).

S.8.3.3 Step 3: Apply evidenced or assumed reject rates to the mass of plastic collected for recycling

Reject rates at the sorting and reprocessing stages were applied to the mass of plastic under each industrial sector / application and plastic type as shown in **Table S33**. The mass of each category was then summed for rigid and flexible material for the formal and informal sectors to provide weighted average reject rates for each category. The reject rates for each process flow are summarised in **Table S32**.

Table S32. Summary of rejects calculated for each process.

Formality	Format	System component	Proportion of collected for recycling that is rejected (lost) before conversion
	Rigid	C24aa	40.74
Formal	Flexible	C24ab	58.08
	Rigid	C23aa	18.84
Informal	Flexible	C23ab	14.90

Beta-PERT distributions were assigned for rejects taking the value reported in **Table S32** as the most likely value, and assigning a ±20% uncertainty to each for the upper and lower plausible bounds, and assuming a shape factor of four.

Table S33. Reject rates applied to main plastic types for three municipal solid waste industrial sectors / applications.

Industrial sector / application	Plastic type by dominant polymer	Collected for recycling mass (Mt)							Sorting reject rates (%)				Post sorting mass (Mt)				Reprocessing reject rates (%)		Post reprocessing mass (Mt)				Post reprocessing rejects as proportion of collected for recycling (%)			
		Form.	Inf.	Form. + Inf.	Form.		Inf.		Form.		Inf.		Form.		Inf.		Form. + Inf.		Form.		Inf.		Form.		Inf.	
		Rig. & flex.			Rig.	Flex.	Rig.	Flex.	Rig.	Flex.	Rig.	Flex.	Rig.	Flex.	Rig.	Flex.	Rig.	Flex.	Rig.	Flex.	Rig.	Flex.	Rig.	Flex.	Rig.	Flex.
Consumer & institutional	HDPE	0.86	1.04	1.91	0.86	0.00	1.04	0.00	24	100	5	5	0.66	0.00	0.99	0.00	15.48	29.00	0.55	0.00	0.84	0.00	35.76	na	19.71	na
	LDPE*	0.62	0.75	1.38	0.00	0.62	0.00	0.75	v	42	na	5	0.00	0.36	0.00	0.72	na	1.00	0.00	0.36	0.00	0.71	na	42.58	na	5.95
	Other	0.01	0.02	0.03	0.01	0.00	0.02	0.00	100	na	5	na	0.00	0.00	0.01	0.00	na	na	0.00	0.00	0.01	0.00	100.00	na	5.00	na
	PET	0.00	0.00	0.00	0.00	0.00	0.00	0.00	na	na	na	na	0.00	0.00	0.00	0.00	13.76	29.00	0.00	0.00	0.00	0.00	na	na	na	na
	PP	1.27	1.54	2.82	1.27	0.00	1.54	0.00	43	na	5	na	0.73	0.00	1.46	0.00	2.84	29.00	0.71	0.00	1.42	0.00	44.62	na	7.70	na
	PS	0.16	0.19	0.36	0.16	0.00	0.19	0.00	100	na	5	na	0.00	0.00	0.18	0.00	34.00	29.00	0.00	0.00	0.12	0.00	100.00	na	37.30	na
	PUR	0.07	0.08	0.15	0.00	0.07	0.00	0.08	na	80	na	5	0.00	0.01	0.00	0.08	na	29.00	0.00	0.01	0.00	0.06	na	85.80	na	32.55
	PVC	0.08	0.09	0.17	0.08	0.00	0.09	0.00	50	na	5	5	0.04	0.00	0.09	0.00	20.00	29.00	0.03	0.00	0.07	0.00	60.00	na	24.00	na
**Consumer & institutional total**		**3.08**	**3.72**	**6.80**	**2.39**	**0.69**	**2.89**	**0.84**	**na**	**na**	**na**	**na**	**1.42**	**0.37**	**2.74**	**0.79**	**na**	**na**	**1.29**	**0.37**	**2.47**	**0.76**	**45.94**	**46.84**	**14.54**	**8.57**
Electrical/ electronic	HDPE	0.08	0.08	0.16	0.08	0.00	0.08	0.00	90	na	85	na	0.01	0.00	0.01	0.00	na	na	0.01	0.00	0.01	0.00	90.00	na	85.00	na
	LDPE*	0.00	0.00	0.00	0.00	0.00	0.00	0.00	na	na	na	na	0.00	0.00	0.00	0.00	na	na	0.00	0.00	0.00	0.00	na	na	na	na
	Other	0.02	0.02	0.03	0.02	0.00	0.02	0.00	90	na	85	na	0.00	0.00	0.00	0.00	na	na	0.00	0.00	0.00	0.00	90.00	na	85.00	na
	PET	0.00	0.00	0.00	0.00	0.00	0.00	0.00	na	na	na	na	0.00	0.00	0.00	0.00	na	na	0.00	0.00	0.00	0.00	na	na	na	na
	PP	0.28	0.27	0.55	0.28	0.00	0.27	0.00	90	na	85	na	0.03	0.00	0.04	0.00	na	na	0.03	0.00	0.04	0.00	90.00	na	85.00	na
	PS	0.05	0.05	0.10	0.05	0.00	0.05	0.00	100	na	100	na	0.00	0.00	0.00	0.00	na	na	0.00	0.00	0.00	0.00	100.00	na	100.00	na
	PUR	0.03	0.03	0.05	0.03	0.00	0.03	0.00	100	na	100	na	0.00	0.00	0.00	0.00	na	na	0.00	0.00	0.00	0.00	100.00	na	100.00	na
	PVC	0.04	0.04	0.09	0.04	0.00	0.04	0.00	90	na	100	na	0.00	0.00	0.00	0.00	na	na	0.00	0.00	0.00	0.00	90.00	na	100.00	na
**Electrical/ electronic total**		**0.51**	**0.49**	**1.00**	**0.51**	**0.00**	**0.49**	**0.00**	**na**	**na**	**na**	**na**	**0.04**	**0.00**	**0.06**	**0.00**	**na**	**na**	**0.04**	**0.00**	**0.06**	**0.00**	**91.57**	**na**	**88.68**	**na**
Packaging	HDPE	5.20	6.59	11.79	4.84	0.36	5.96	0.64	24	100	5	5	3.68	0.00	5.66	0.61	15.48	29.00	3.11	0.00	4.78	0.43	35.76	100.00	19.71	32.55
	LDPE*	3.00	3.90	6.90	0.00	3.00	0.00	3.90	na	42	na	5	0.00	1.74	0.00	3.71	na	1.00	0.00	1.73	0.00	3.67	na	42.58	na	5.95
	Other	0.01	0.01	0.01	0.00	0.00	0.01	0.00	100	100	5	5	0.00	0.00	0.01	0.00	20.00	29.00	0.00	0.00	0.00	0.00	100.00	100.00	24.00	32.55
	PET	4.24	5.39	9.63	4.04	0.21	5.02	0.36	19	100	5	5	3.27	0.00	4.77	0.35	13.76	29.00	2.82	0.00	4.12	0.25	30.15	100.00	18.08	32.55
	PP	3.00	3.80	6.80	2.30	0.70	2.57	1.23	43	100	5	5	1.31	0.00	2.45	1.17	2.84	29.00	1.27	0.00	2.38	0.83	44.62	100.00	7.70	32.55
	PS	0.21	0.27	0.48	0.21	0.00	0.27	0.00	100	100	5	na	0.00	0.00	0.26	0.00	34.00	na	0.00	0.00	0.17	0.00	100.00	na	37.30	na
	PUR	0.01	0.02	0.03	0.01	0.00	0.02	0.00	100	100	5	na	0.00	0.00	0.01	0.00	20.00	na	0.00	0.00	0.01	0.00	100.00	na	24.00	na
	PVC	0.13	0.16	0.29	0.10	0.03	0.12	0.05	100	100	5	5	0.00	0.00	0.11	0.04	20.00	29.00	0.00	0.00	0.09	0.03	100.00	100.00	24.00	32.55
**Packaging total**		**15.81**	**20.14**	**35.95**	**11.51**	**4.30**	**13.96**	**6.18**	**na**	**na**	**na**	**na**	**8.26**	**1.74**	**13.26**	**5.87**	**na**	**na**	**7.20**	**1.73**	**11.55**	**5.21**	**37.41**	**59.88**	**17.29**	**15.76**
**Grand total**		**19.40**	**24.35**	**43.75**	**14.40**	**4.99**	**17.34**	**7.01**	**na**	**na**	**na**	**na**	**9.72**	**2.12**	**16.06**	**6.66**	**na**	**na**	**8.54**	**2.09**	**14.07**	**5.97**	**40.74**	**58.08**	**18.84**	**14.90**

High income countries are assumed to be formal and non-high-income countries are assumed informal. *LDPE includes LLDPE. Abbreviations: Formal (Form.), informal (Inf.); rigid (rig.); flexible (flex.); million tonnes (Mt); high density polyethylene (HDPE); low density polyethylene (LDPE); linear low-density polyethylene (LLDPE); polyethylene terephthalate (PET); polystyrene (PS); polyvinyl chloride (PCV); polypropylene (PP); polyurethane (PUR).

S.8.3.4 Mismanagement of rejects from sorting and reprocessing (C25aa, C25ab, C26aa, C26ab)

To understand the proportion of rejects which are mismanaged, we created a further sub-model which used collection coverage and street sweeping efficiency to approximate mismanagement activity data (**Fig. S17**).

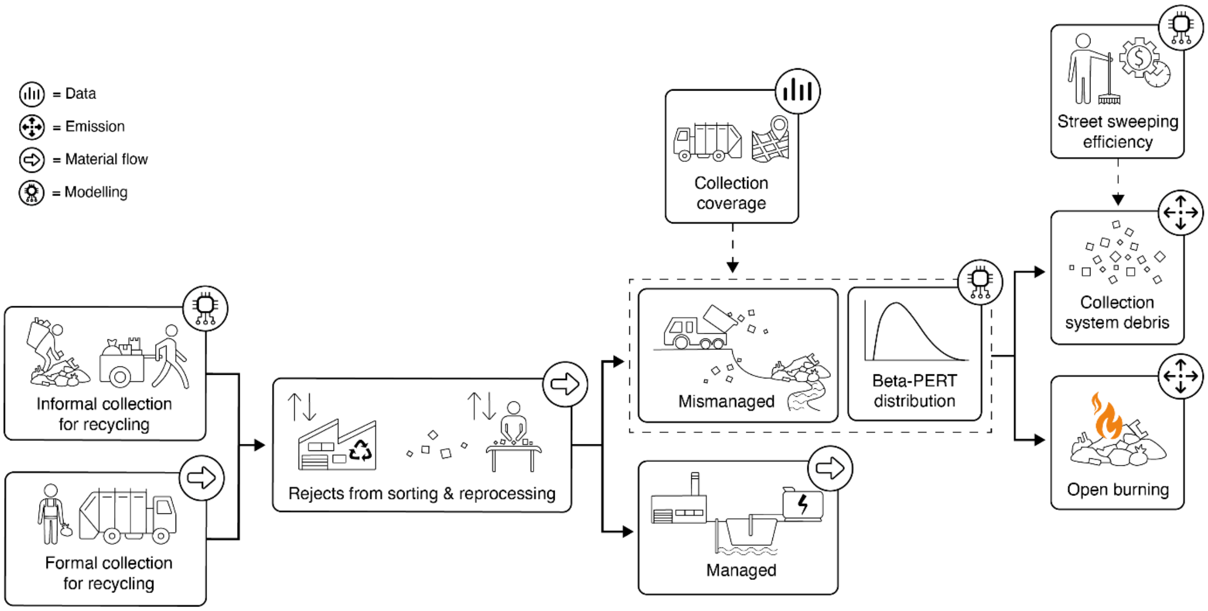

Fig. S17. Sub-model to estimate the quantity of rejects from sorting and reprocessing which are mismanaged.

We assumed that rejects from sorting and reprocessing (C25aa, C25ab, C26aa, C26ab) were connected to waste management collection coverage and street sweeping efficiency using **Equation S1** .

$$M_L = (100 - C2) \times \left(1 - \frac{S}{100}\right) \quad \textbf{Equation S1}$$

Where:

- C2 is the collection coverage from the *Full MSW MFA;*
- S is the assumed street sweeping efficiency (%) as sampled from a Beta-PERT distribution according to the parameters in **Table S35;**
- M_L is the rate of mismanagement of sorting and reprocessing rejects for rigid plastic collected by informal sector (C25aa); flexible plastic collected by the informal sector (C25ab); rigid plastic collected by the formal sector (C26aa); and flexible plastic collected by the formal sector (C26ab).

S.8.4 Proportion of plastic in formal sector collection for recycling

The amount of waste collected by the formal recycling sector is an input (tC2i) to the *Tributary MFA*. The proportion of this waste that is plastic (C16) was estimated at 8.5% based on data for the UK from Department for Environment Food and Rural Affairs (Defra)[38]. As no uncertainty was provided in the original source, an assumed 50% error for both low and high estimates was assigned and modelled with a Beta-PERT distribution. The amount of rigid plastic in formally collected material for recycling as a percentage of plastic collected (C22a) was assumed the same as C0a.

S.8.5 Uncollected litter (C1)

Litter is often used as a generic term to describe waste that is in the environment with no distinction given to its emission source (point of initial release). In this work we adopt a definition which states that litter must originate from littering, defined here as: '*the act of discarding items of waste generated on-the-go (in the public domain) directly into the environment without it having previously been concentrated or containerised'*. This distinguishes more sparsely generated, usually single item deposits from larger deposits into the environment (open dumping), each of which will have different factors affecting the probability of movement, and the magnitude and frequency of their occurrence.

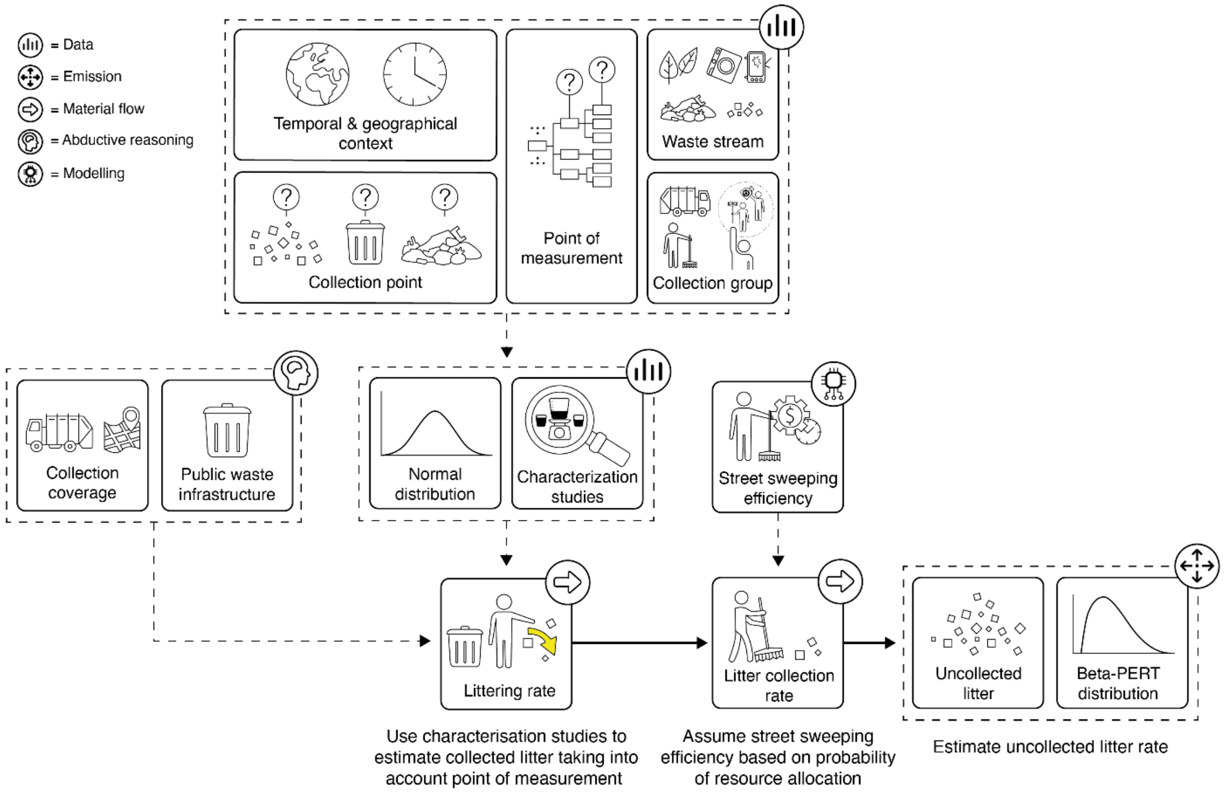

Fig. S18. Sub-model to estimate emissions from uncollected litter.

Published littering data is usually a measure of litter that has been collected, either via street bins, street cleansing (litter picking) or irregular environmental clean-ups (e.g., beach cleaning)[291]. However, the amount of litter which is uncollected is challenging to measure because it does not pass through any system of management and often becomes dispersed soon after it is emitted[292,293].

To estimate the amount of *uncollected litter* (C1), we developed a sub-model as illustrated in **Fig. S18.** First, we calculated the amount of litter deposited on the floor that is subsequently collected by a municipality using measured data from Europe, termed here the *littering rate* (**Section S.8.5.1**). We then corrected the *littering rate* to estimate of *total litter* (L_T) by dividing by an assumed street sweeping efficiency (S) for these European cities (**Section S.8.5.2**). Finally, as we had used European data to calculate the *littering rate,* we had to adjust it to be relevant for the Global South by assuming that waste receptacle provision and collection quality and efficiency was less comprehensive. We then divided the complement of the assumed street sweeping efficiency percentage to calculate the fraction which was *uncollected litter* (C1) (**Section S.8.5.3**).

S.8.5.1 Littering rate

We began by classifying data on collected litter according to the point in the system at which litter was measured, and the temporal and geographical context using a typology proposed by Elliott, et al.[294] as follows:

- **Collection point** – Litter is typically either measured based on what is placed in public waste bins (bin litter), or what is collected from the environment (ground litter, river litter etc.).
- **Waste stream –** Street cleansing teams may collect fly-tipped (informal open dumping on land) waste, side-waste (waste placed alongside bins), green waste (e.g., leaves), or perform street sweeping which will likely have high amounts of soil, vegetation as well as small amounts of litter. Understanding what waste streams are included in a measurement is important for both the mass and the composition.
- **Collection group –** Litter may be collected either by municipal street cleansing crews or by other groups such as commercial operators or volunteer organisations.
- **Area** – In order to extrapolate littering rates, the residential and visiting population of an area must be determined and related to a geographical area.
- **Time** – The time since any previous litter collection is important to understand to be able to infer the rate of littering.

As we required the *littering rate* to be equivalent to litter deposited on the floor, we needed to exclude other wastes which are commonly reported within the same category such as: waste deposited in bins; naturally occurring litter (e.g., leaves); non-littering sources such as fly tipping (informal open dumping); and waste which had overflowed from non-litter bins. Elliott, et al.[294], reported *littering rates* from five European locations, excluding litter deposited in bins, natural litter (for example leaves, tree debris, soil, and insects) and fly-tipping (informal open dumping) (**Table S34**). Waste from overflowing bins was not mentioned therefore is likely included in the measurements, potentially resulting in double counting in our model. However, considering the data was collected across the EU where bins are relatively well managed, it is assumed this contribution is negligible.

For consistency, the *littering rate* was converted from per capita rates to as a proportion of MSW generation as used in other works[2,3]. This was sampled according to a normal distribution with mean of 0.81 and standard deviation 0.15 (**Table S34**).

Table S34. Littering rates in European cities and countries.

Location	Date	Per capita littering rate ($kg \cdot cap^{-1} \cdot y^{-1}$)	MSW generation rate** ($kg \cdot cap^{-1} \cdot y^{-1}$)	Per capita littering rate (% of MSW generation)
Bristol, UK	Approx. 2016	4.8	479	0.99
Scotland, UK	Approx. 2012	3.3	483	0.68
East Lothian, Scotland, UK	Approx. 2012	4.8	483	0.99
Flanders, Belgium	2013	2.72	436	0.62
Flanders, Belgium	2015	3.17	412	0.77
**Mean**	**-**	**3.76**	**-**	**0.81**
**Standard deviation**	**-**	**0.87**	**-**	**0.15**

* as reported by Elliott, et al.[294]; ** linearly interpolated to correct year based on data reported in Eurostat[295]; Abbreviations: Municipal solid waste (MSW).

S.8.5.2 Total litter (L_T)

The *littering rate* discussed in **Section S.8.5.1** relates only to litter that was deposited on the ground and subsequently collected by the municipality; therefore, it excludes litter that remained uncollected in the environment. To better approximate the *total litter*, including the uncollected proportion, we created another sub-model to estimate street sweeping efficiency (S), defined as the amount of litter that is collected as a proportion of *total litter* generation.

In reality, street sweeping efficiency is affected by many factors including: the method used to clean the streets; the frequency and timing of cleaning; access to the waste (including the presence of obstacles such as parked cars and vegetation); environmental conditions (e.g., wind and frequency of rainfall); and the pollutant that is being collected (e.g., litter, sediment, and leaves)[296,297]. However, data to evidence each of these factors are not available at global scale, so we based our model on two broad assumptions:

1. Anecdotally, street sweeping activities are more likely to occur in highly frequented and prominent places such as city centers, around tourist attractions, financial centers and in commercial areas, whilst rural areas may have less frequent street cleansing if at all. We therefore assume that street sweeping is more efficient in urban and less in rural areas.
2. By weight, the cost of street sweeping outweighs that of collection of concentrated waste from containers, particularly if drains are cleansed[298]. Given countries in the Global South often lack the funds to carry out basic waste collection services, it is appropriate to assume that on average, formal street sweeping activities are less comprehensive in lower income countries.

Street sweeping efficiencies and uncertainty assumed in the present work are shown in **Table S35** according to the country income category and the settlement typology of each municipality, as determined via data from the Global Human Settlement – Settlement Model (GHS-SMOD)[196] (**Section S.7.1**). Many of the efficiencies were assigned as negatively skewed (long tails to the

left) to account for the premise that although the majority municipalities will likely recognise the importance of street sweeping, a minority of municipalities may neglect it.

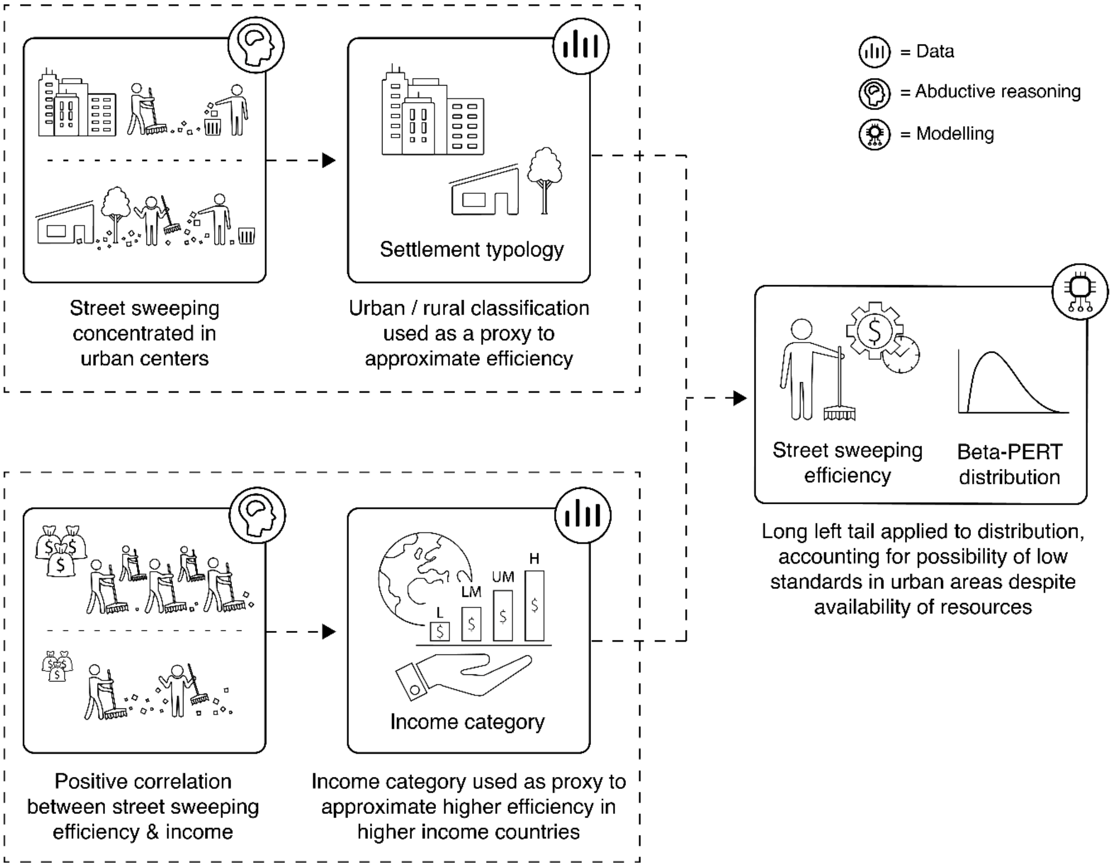

Fig. S19. Sub-model to estimate street sweeping efficiency across the world's municipalities.

Given the street sweeping efficiencies in **Table S35**, the *littering rate*, which is based solely on European data (**Table S34**), was corrected to an estimate of *total litter* by dividing by the street sweeping efficiency, as sampled for a HIC assuming a semi-dense urban settlement typology and a Beta-PERT distribution.

Table S35. Assumed street-sweeping efficiencies (% wt. ar) by country income category and settlement typology[196].

Income category	Settlement typology	Minimum efficiency (%)	Most likely efficiency (%)	Maximum efficiency (%)
HIC	Urban centre	90	99	100
	Dense urban	80	97.5	99
	Semi-dense urban	70	95	97.5
	Suburban	60	92.5	95
	Rural	50	90	92.5
UMC	Urban centre	80	95	100
	Dense urban	50	80	85

Income category	Settlement typology	Minimum efficiency (%)	Most likely efficiency (%)	Maximum efficiency (%)
	Semi-dense urban	20	70	75
	Suburban	0	50	55
	Rural	0	20	25
LMC	Urban centre	50	80	90
	Dense urban	20	60	70
	Semi-dense urban	0	20	30
	Suburban	0	10	20
	Rural	0	5	15
LIC	Urban centre	0	20	30
	Dense urban	0	0	5
	Semi-dense urban	0	0	5
	Suburban	0	0	5
	Rural	0	0	5

Abbreviations: Low-income country (LIC); high income country (HIC); lower middle-income country (LMC); upper middle-income country (UMC).

S.8.5.3 Uncollected litter (C1)

The proportion of *uncollected litter* (C1) for each municipality was divided by the complement of the street sweeping efficiency to calculate *total litter*. Street sweeping efficiencies (S) were in turn calculated for each municipality by sampling from Beta-PERT distributions according to the values in **Table S35** and weighting these by the percentage of the population living in each settlement typology. GHS-SMOD level two rural classifications of 'rural cluster', 'low density rural', 'very low density rural' and 'water' were simplified here to a single 'rural' classification.

The *total litter* calculated in **Section S.8.5.2** is based on European data and cannot be assumed representative of all global municipalities, particularly given many municipalities may provide fewer public waste infrastructure than for the European cities. Accordingly, a further correction was required to estimate the *total litter* for all global municipalities. In the absence of data on the provision of public waste infrastructure, the collection coverage (tC1) of the municipality was used as a proxy. The *uncollected litter* for each municipality was then estimated using **Equation S2.**

$$C1 = L_T \times \left(1 + \log\left(\frac{100}{tC1}\right)\right) \times \left(1 - \frac{S}{100}\right) \qquad \textbf{Equation S2}$$

Where:

- L_T is the *total litter* (% of MSW generation) estimated based on European data as described in **Section S.8.5.2**.
- tC1 is the collection coverage, used here to estimate *total litter* in a global context.
- S is the street sweeping efficiency (%) calculated as the weighted sum of its population by settlement typology as sampled from a Beta-PERT distribution according to the values in **Table S35**.

S.8.6 Proportion of plastic and rigid plastic in uncollected litter (C11 and C11a)

The *secondary data inputs* relating to the proportion of litter that is plastic (C11) and rigid plastic (C11a) were obtained from a study of the composition of litter in Wales[37]. The author sampled litter both in waste bins and that picked from the ground. The composition of litter picked from the ground is likely to be more applicable to the uncollected litter used here, therefore only this data was used in this analysis. On a weight basis and excluding the collection sacks, plastic as a proportion of litter (C11) was on average 17.7% with a minimum of 13.8% and maximum of 20.4%. On the other hand, the proportion of this plastic that is rigid was on average 72.9% with a minimum of 69.1% and a maximum of 76%. These values were converted into PDFs for the probabilistic MFA using a Beta-PERT distribution with shape factor of four.

S.8.7 Uncollected MSW (C2)

Uncollected MSW differs from littering in that it has been concentrated (i.e., not an individual item), usually in a premises (household or business) and occurs in the context where waste collection services are either un-affordable or unavailable. Likewise, unlike littering, uncollected waste may be open burned or purposely dumped in a specific location (e.g., rivers, disused land etc.). The mass of uncollected waste was determined based on the complement of the collection coverage (C2) and as such is calculated directly in the *Full MSW MFA* as part of process P4 (**Fig. S5**). The proportion of uncollected waste that is openly burned compared to dumped into the environment as debris emissions is discussed in **Section S.8.11.1**

S.8.8 Debris emissions from collection system (C3)

The act of storing, collecting, and transporting MSW to recovery or disposal facilities is grouped here by the term ‘collection system’. Emissions of debris can occur at several points in this system; for example, by blowing out of bins, being dropped as it is loaded into vehicles, or by falling from collection vehicles. The authors have found no reliable quantification of these emissions into the environment; therefore, emissions were estimated via a sub-model (**Fig. S20**).

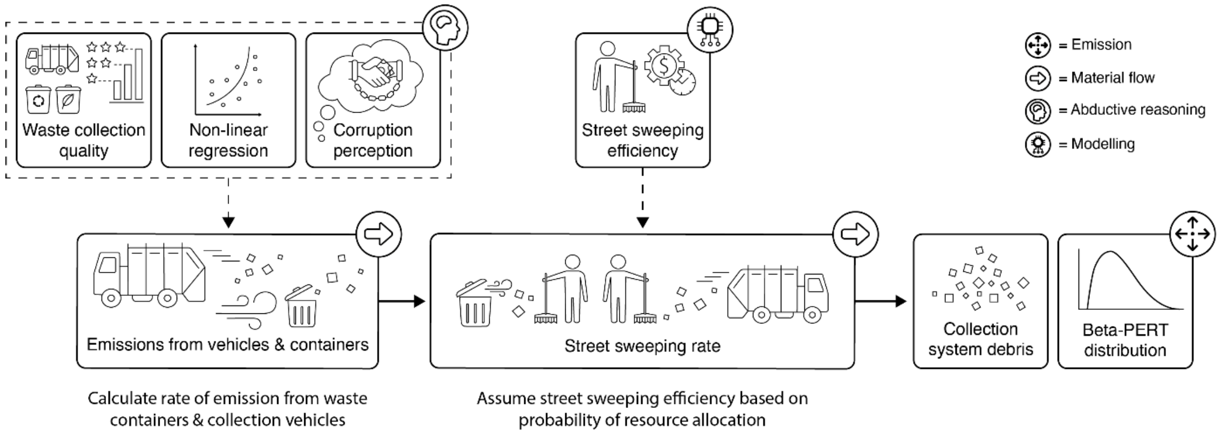

Fig. S20. Sub-model to estimate emissions from collection systems.

Firstly, we assumed that emissions from the collection system were proportional to the quality of the collection. This quality of collection is an indicator measured in the Wasteaware Cities Benchmark Indicators (WABI) toolkit[39] based on assessment of criteria, including the appearance of waste collection points and the effectiveness of transport. Recent analysis has demonstrated the strong link between socio-economic development, as measured through relevant indices, and solid waste management performance as measured by WABI for waste generation, collection coverage, quality of collection, controlled recovery and disposal and environmental protection[41]. Non-linear regression identified the strongest predictor for waste collection quality was that of the corruption perceptions index (CPI). Municipal data on CPI (**Section S.7.1**) was therefore used to predict the quality of collection for all municipalities according to the curve described by **Equation S3,** as derived from Velis, et al.[41]:

$$Quality\ of\ collection = 20.7 + 35.4\log(CPI) \qquad \textbf{Equation S3}$$

The quality of collection was used to predict emissions from the collection system as a proportion of waste collected prior to any street sweepings (C3i) by linearly interpolating between assumed emissions for a best (100% quality collection) and worst (0% quality of collection) scenario. It was estimated that in a best-case scenario, 0% of the waste for collection is emitted into the environment, whereas for a worst-case scenario 5% of waste for collection is emitted (1% low estimate, 15% high estimate). A Beta-PERT distribution was used to model the uncertainty around these emissions.

Lastly, to account for waste which was emitted from the collection system and then subsequently collected, the sampled emission rate (C3i) was multiplied by the complement of the street sweeping efficiency for the relevant settlement typology and income category listed in **Table S35**. This is summarised in **Equation S4**.

$$C3 = C3i \times \left(1 - \frac{S}{100}\right) \qquad \textbf{Equation S4}$$

Where:

- C3 is the emissions from the collection system (after street sweeping) – (% of collected waste)
- C3i is the emissions from the collection system (before street sweeping) – (% of collected waste)
- S is the street sweeping efficiency (% of emitted waste)

The proportion of the collection system emissions that is plastic (C13) was assumed equal to the proportion of MSW that is plastic (C0). Likewise, the proportion of these plastic emissions that are rigid plastic (C13a) was assumed to be the same as the proportion of rigid plastic in MSW (C0a).

S.8.9 Debris emissions from uncontrolled disposal of MSW (C9)

Solid waste is emitted into the environment from uncontrolled disposal sites in two ways: 1) as debris (physical material); and 2) via open burning (combustion in open uncontrolled fires). As far as we are aware, no works have reliably measured these emissions from land disposal sites. Yadav, et al.[299] proposed a conceptual framework for estimating debris emissions from specific land disposal sites based on their physical structure, geographical and topological context and meteorological conditions. Gathering that level of data for all global land disposal sites would be infeasible. Therefore, we developed a simplified conceptual model to estimate the probability of debris emission because of how much plastic waste was exposed to wind and surface water runoff and therefore how much is likely to mobilise and be transported into the environment (**Fig. S21**).

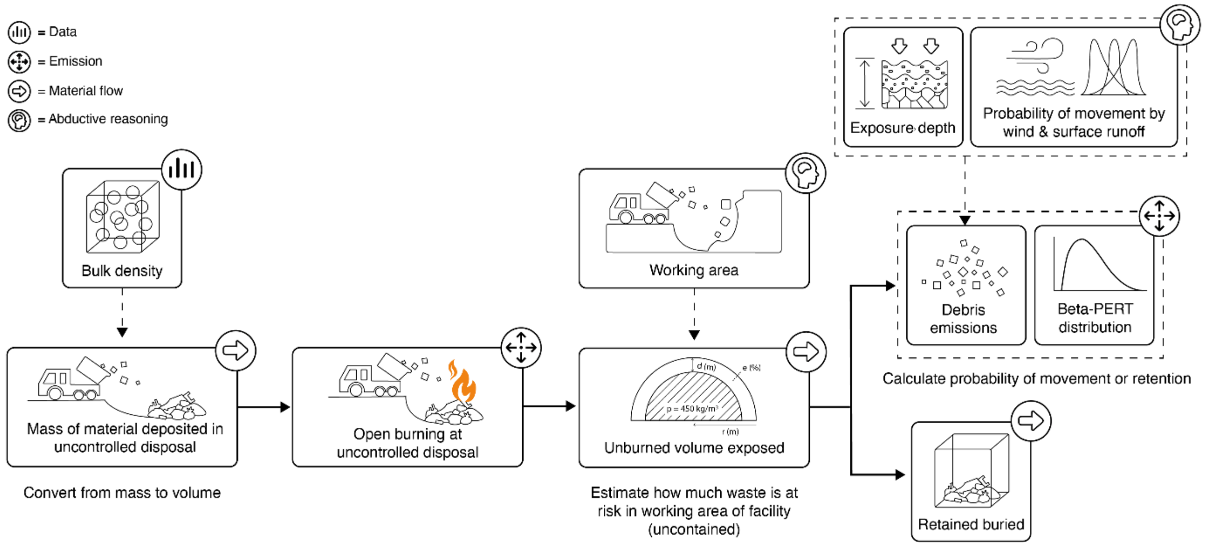

Fig. S21. Sub-model for estimating emissions from uncontrolled disposal.

We assumed that most emissions occur on freshly deposited waste whilst it is still relatively loose and before any settling or compaction (natural or mechanical) takes place. Therefore, only the ‘working area’ (‘working face’) was quantified, meaning the part of the site where waste is deposited, manipulated, or in the case of sites where the informal sector operate, recovered from.

To calculate the proportion of waste that is exposed, assumptions about typical dimensions of the working area of uncontrolled disposal sites were posited. These included the dumpsite shape, working area, bulk density, and exposure depth (**Fig. S22**). Simple geometry calculations enabled the volume, mass, and surface area of the dumpsite to be derived, the latter of which was multiplied by the exposure depth and bulk density to arrive at an approximation for exposed mass. This exposed mass was multiplied by an assumed emission rate to derive the mass emitted, which when divided by the overall mass gives the emissions as a percentage of uncontrolled unburned disposal (C9).

A hemisphere shape was chosen based on its simplicity and broad similarity with dumpsite profiles, whilst the bulk density (ρ) was assumed constant at 450 $kg \cdot m^{-3}$ [300,301]. The working

area radius (r), exposure depth (e) and emission rate for exposed waste are all highly uncertain parameters, and therefore were varied according to best estimates to provide low, mid and high point estimates. For instance, as the working area radius increases, the surface area to volume ratio decreases, leading to lower exposed mass as a percentage of total mass. The low emission estimate had a larger working radius of 50 m, as opposed to 30 m in the central estimate and 10 m in the high estimate. Alternatively, as the exposure depth increases, so do the calculated emissions, therefore a low estimate assumed a value of 10 cm, mid estimate of 20 cm and high estimate of 30 cm. These values are all on the same order of magnitude as typical waste items under the assumption that once an item is covered by another, its exposure to wind and surface water is nullified. Lastly, the emission rate was assumed as 1% in a low estimate, 2% mid-estimate and 3% high estimate. These values gave the overall emissions from uncontrolled disposal as a proportion of disposed waste as: 0.006% (low-estimate), 0.04% (mid-estimate) and 0.45% (high-estimate) which were assigned a Beta-PERT distribution. Although these numbers may seem small, it should be noted that disposal sites contain large amounts of waste, therefore even small emission rates can lead to large overall masses of waste being emitted into the environment. Similarly, the distribution of estimates shows that whilst the central estimate of 0.04% is relatively small, the high estimate leads to a high right-skewed distribution signifying large emission rates may be possible although less likely.

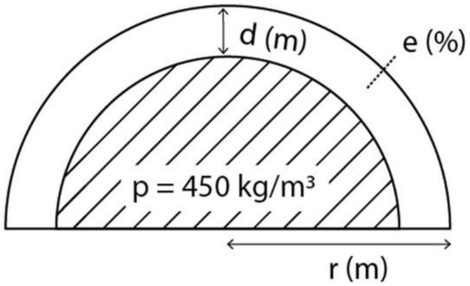

Fig. S22. Conceptual model for calculation of exposed mass in an uncontrolled disposal site. Abbreviations: r is the dumpsite working radius (m), ρ is the bulk density of waste (450 kg·m^{-3}), d is the exposure depth (m) and e is the emission rate (% of exposed waste).

S.8.10 Plastic (C14) and rigid plastic (C14a) in disposal debris emissions

The proportion of the uncontrolled disposal debris emissions that are plastic (C14) was assumed based on the hypothesis that lighter materials are those most susceptible to release, particularly by wind. It is therefore likely that both paper and plastic are the items predominantly released at disposal sites. Without any available data to inform this split, it was assumed 50% of emissions are plastic (40% minimum, 60% maximum). Likewise, given that plastic most susceptible to movement by wind are likely plastic films, the proportion of plastic emissions taken to be rigid plastic (C14a) was assumed as 10% (5% minimum, 15% maximum). Lastly, each of these disposal debris emission variables were converted into PDFs by assuming a Beta-PERT distribution.

S.8.11 Open burning

S.8.11.1 Open burning of uncollected waste (C10)

Data to estimate the mass of MSW burned in open uncontrolled fires (C10) are scarce and seldom robust, being driven by assumptions and expert judgement[302]. Therefore, it was necessary to build a sub-model which combined activity data from census and surveys with income category and settlement typology data to estimate the prevalence of the practice in each of the world's municipalities (**Fig. S23**).

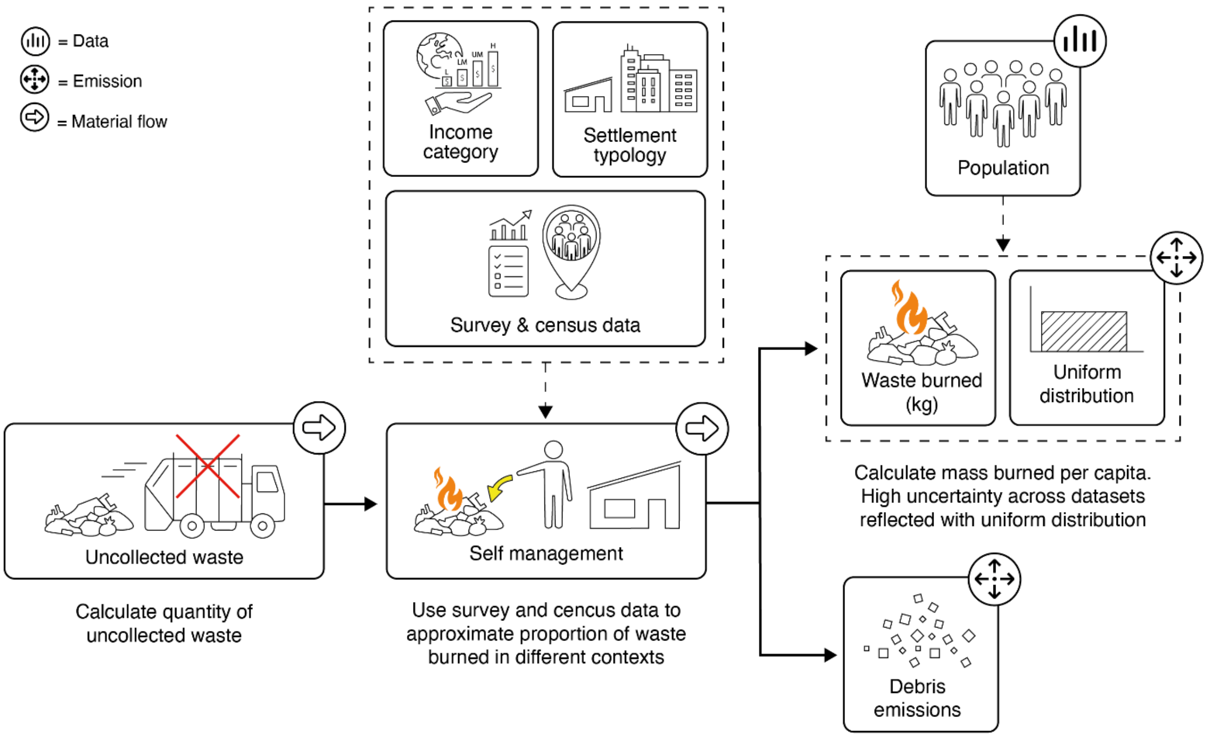

Fig. S23. Sub-model for estimating open burning emissions from uncollected waste.

We collected census and health survey data that queried waste management practices in 44 countries, spanning from 1996-2021[303-335]. In the absence of data on the mass of waste burned in open uncontrolled fires, we used these activity data as a proxy for the amount of waste burned. In agreement with several authors[5,31,291], we found that the amount of uncollected waste in a system reduces as a country's income increases (**Fig. S24A**) and that uncollected waste is far higher in rural areas compared to urban areas (**Fig. S24B**). In this context, we also make three observations about the amount of waste burned in the Global South: **(1)** The range of data for both open burning (as proportion of uncollected waste) and uncollected waste in LMCs is large, indicating huge variation in practices within that income category; **(2)** As a proportion of the total waste generated in LICs (where waste collection rates are generally higher in major cities but virtually absent in many rural areas (**Fig. S24B**), waste burning is slightly lower than LMCs, which in turn

are slightly higher than UMCs (**Fig. S24C**); and **(3)** As a proportion of uncollected waste (**Fig. S24E**), the amount of waste burned appears to increase as collection coverage increases. Observations **(2)** and **(3)** indicate a development of practices and behaviour that approximately correlates with increased wealth. It appears that as economic development progresses, societies focus their efforts on reducing terrestrial and aquatic dumping rather than open burning. Two reasons are suggested: **(A)** That regulators and policy-makers concentrate on reducing terrestrial and aquatic debris due to its visual unsightliness rather than on open-burning which rightly or wrongly is consider to have made the waste 'disappear'; and **(B)** That the open-burning of waste is overlooked by waste authorities and treasuries, because it reduces the cost of collection, treatment and disposal.

The rate of open burning (as proportion of total waste) in LMCs and UMCs is much higher in rural areas (**Fig. S24D**), whereas in LICs, rural burning occurs at a slightly lower rate compared to urban. It is suggested that this is because LICs have less capacity to enforce regulation on open burning in cities, with this only improving once a country has sufficient resources to fund its environmental regulators sufficiently.

The narrative that open burning varies with income category and settlement typology is plausible, and we have substantial data to support it circumstantially[303-335]. However, the data do not fit a normal distribution and the ranges are large in some cases. On the basis that our model requires open burning data using uncollected waste as a denominator, and acknowledging the large range, we applied a uniform distribution between the ranges (excluding outliers defined as values greater than 1.5 times the interquartile range distance from the 25th and 75th percentiles) for each of the income categories and urban-rural contexts presented in **Fig. S24F**. This decision allows for the observed variation between and within countries to be incorporated into the probabilistic MFA, whilst acknowledging the variation between income categories and settlement typology. The uniform distribution for each municipality was weighted by the urban to rural population.

Data to evidence the amount of waste which is open burned in HICs is extremely limited, and we found a large range (1.2-66.7% wt. of uncollected waste) between the three data points we obtained[303,305,309], all of which were for small island states (Anguilla, Trinidad and Tobago and Cook Islands). Urban-rural data were unavailable, and there are arguments that indicate that waste is burned in both cities and the countryside within high income countries. For instance, KANTAR[336] reported similar rates of outdoor burning in the UK between urban and rural areas and the difference between indoor burning. Therefore, we applied the range (1.2-66.7% wt. of uncollected waste) to both urban and rural areas with a uniform distribution for all HICs.

S.8.11.2 Open burning of rejects from sorting and reprocessing (C27aa, C27ab, C28aa, C28 ab)

As the open burning of mismanaged rejects from sorting and reprocessing is generally an illegal practice, there is no data to estimate its prevalence. Therefore, here, as an approximation we assumed that it takes place at the same rate as for open burning of uncollected waste at household level for rigid plastic collected by informal sector (C27aa); flexible plastic collected by the informal sector (C27ab); rigid plastic collected by the formal sector (C28aa); and flexible plastic collected by the formal sector (C28ab).

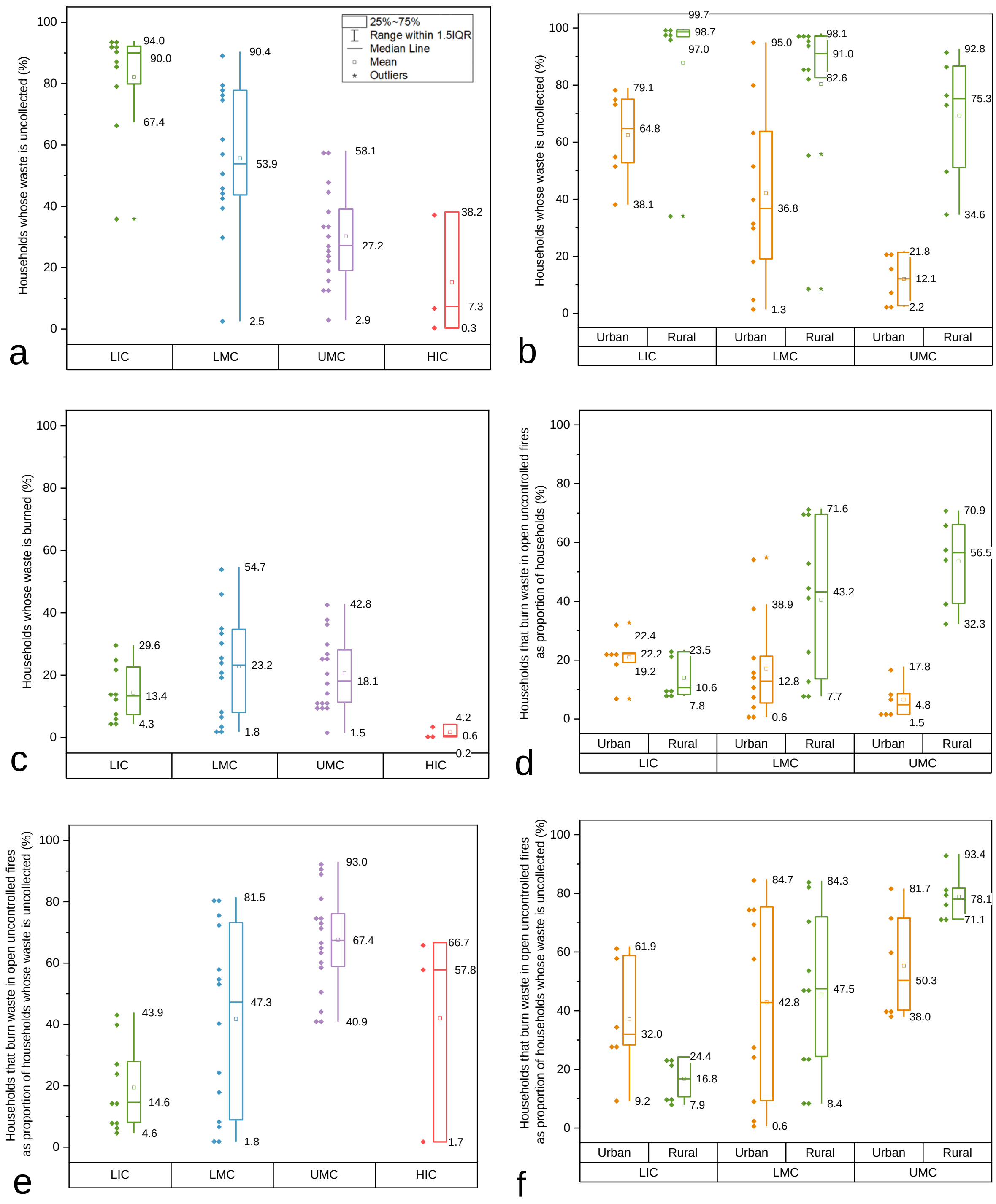

Fig. S24. National average census and survey data (n=44 countries) and country level urban-rural data (n=22 countries) showing: (**A, B**) proportion of householders who reported that waste is uncollected; (**C, D**) proportion of householders who reported burning their waste in open uncontrolled fires; (**E, F**) proportion of householders who reported burning their waste in open uncontrolled fires, as a proportion of households whose waste is uncollected. Abbreviations: Low-income country (LIC); lower middle-income country (LMC); upper middle-income country (UMC); high-income country (HIC); inter-quartile range (IQR)[302].

S.8.11.3 Open burning at uncontrolled disposal sites (C8)

Determining the mass of waste burned at uncontrolled disposal sites is a highly challenging exercise. Landfill / dumpsite fires may be started deliberately or spontaneously[26], with a high variability between events, influenced by management practices which vary substantially between and within countries and regions[6]. Anecdotally, most dumpsites have at least one daily fire, and many are permanently on fire[337]. Even in HICs with highly controlled systems such as the UK, it has been reported that there is at least one fire ablaze on a landfill somewhere[338].

Five estimates of the mass of waste open burned are presented in **Table S36**, alongside the methods used to determine them. All these methods result in highly uncertain outcomes, being strongly driven by assumptions or the judgement of the authors. The Swaziland model[339] is the only one to have modelled at a local scale. The assumptions were based on interviews with the officials who operated the land disposal sites, so the data are considerably more robust than the other models which used assumptions. Moreover, because the data were provided across all the states in the country, we were able to determine the range. We therefore took the mean mass combusted for the whole country (8.6% wt.) and the upper and lower quartiles (0% and 80.2% wt.) and assumed a Beta-PERT distribution.

Table S36. Estimates of waste plastics mass open burned in land disposal sites worldwide.

Country	Year	Income category	Proportion (wt.)	Statistic	Denominator	Method	Source
China	2017		38%	Not stated	Dumpsites	Not stated	340
Global	2014	LMC, LIC, UMC	60%	Mean	Dumpsites	Material flow analysis based on IPCC[341] assumptions	342
		HIC	13%				
India	2010	LMC	10%	Mean	Dumpsites	Interviews with officials	343
Poland	2021	HIC	4.3%	Mean	Landfilled waste	Extrapolation from firefighting service records reported by Bihałowicz, et al.[344] combined	344
Swaziland	2017	LMC	8.6% (0%, 80.2%)	Mean (Upper, lower quartiles of provincial estimates)	Dumpsites	Used waste management data, combustibility estimates based on composition and estimates of how much waste is burned	339

S.9 Probabilistic material flow analysis (MS7)

Material flow analysis is a well-established method for the quantification of material flows within a system. It has been used extensively in many disciplines, for example to quantify the flow of materials through societal systems or for assessing exposure to harmful substances in the environment[345]. A core feature of material flow analysis is the conservation of mass, which requires the modeller to find ways to account for all material within the system boundary[346]. This means a great deal of data may be required to model complex systems, which can be challenging to obtain[347]. Frequently, assumptions are used in place of measured process (activity) data[348] which can result in greater uncertainty in models[349].

Probabilistic material flow analysis overcomes some of these challenges by ascribing uncertainty to the input parameters of a model[350]. This uncertainty is then propagated through the system to

enable the user to assess the probability distribution around the various flows and processes. One way to achieve probabilistic material flow analysis is to perform Monte Carlo analysis, a stochastic method that requires probability density functions to be applied to model inputs. The material flow model is then repeated for many iterations, each one sampling randomly from the input PDFs. Results are output as probability distributions that can be summarised according to the requirements of the user. Probabilistic material flow analysis has been applied successfully to assess plastic pollution, circular economy and many other material and substance flow systems[351-356].

As described in **Section S.4** (**Fig. S4 - Fig. S8**), material flows were quantified across three systems using probabilistic material flow analysis. Predictions from the random forest were used as its inputs to the *Tributary MFA* so that the major, measured, and readily reported formal flows of MSW could be quantified. The process masses calculated in the *Tributary MFA* were then used as inputs into the second MFA, the *Full MSW MFA*. This MFA builds upon the *Tributary MFA* to include flows that are not typically measured by municipalities, such as informal sector collection of recyclables, and emissions of waste into the environment. These extra processes were calculated using the coefficients described in **Section S.8**, informed by sub-models described in Sections **S.8.2, S.8.3, S.8.3.4, S.8.5, S.8.5.2, S.8.8, S.8.9, S.8.11.1** and **S.9.1.2**.

The results of the *Full MSW MFA* were used to populate the *Plastics MFA* which converted the full MSW fraction to plastic in both rigid and flexible formats. These conversions were again achieved using the coefficients described in **Section S.8**. A full list of equations used in all the MFAs is included in **Supplementary Data 2**.

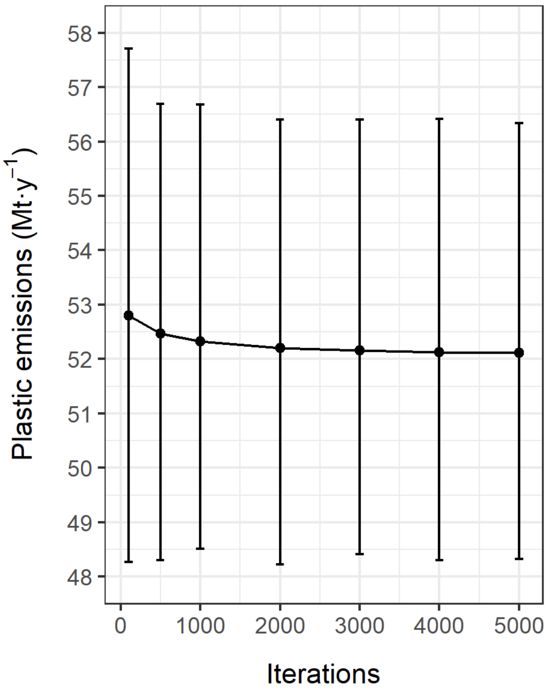

Fig. S25. Comparison of global plastic emissions (Mt·y^{-1}) versus number of iterations used in the probabilistic material flow analysis (MFA) showing results stabilise ~5,000 iterations. Dots represent the mean value, whereas error bars show the 5th and 95th percentile.

The probabilistic nature of the MFA was implemented using Monte Carlo analysis with 5,000 iterations. This meant that each of the 50,702 municipalities had 5,000 separate MFAs generated, whereby the input data for each MFA was randomly sampled from probability density functions or predictions from the random forest models (**Section S.9.1**). The 5th percentile, lower quartile, median, mean, upper quartile and 95th percentile values of the MFA results for each municipality were then used to summarise the outputs and uncertainty.

The number of iterations deemed suitable was deduced by repeatedly implementing the probabilistic MFA with increasing number of iterations and recording the point at which the mean, 5th percentile and 95th percentile of overall plastic emissions into the environment stabilised (**Fig. S25**).

S.9.1 Data inputs

We chose 2020 as the baseline year for our model to enable best relevance to the UN Treaty on Plastic Pollution, agreed in 2022 through Resolution UNEP/EA.5/Res.14[357] and being negotiated by the International Negotiating Committee (INC)[358] in 2023. The choice of year was adopted tentatively as it is towards the top of the range (2006-2021) of our *primary input data*, which would ideally have been more recent. Though our decision introduced some small error to our model because waste management practices and behaviours change over time, we balanced that against the need to apply our data to a contemporary demographic. Therefore, population and settlement typology for the year 2020 was calculated for each municipality from the Global Human Settlement Population dataset (GHS-POP)[184] according to the method described in (**Section S.7.1**). It is anticipated that future iterations of our model will be implemented with more up-to-date primary data collected using the UN-Habitat[6] SDG11.6.1 estimator Waste Wise Cities Tool (WaCT) data collection protocol, which is currently deployed world-wide, and with which our approach is fully compatible.

S.9.1.1 Random sampling of primary input data

The quantile regression random forest method (**Section S.7.3**) was chosen as it allows uncertainty to be incorporated into the random forest predictions used in the *Tributary MFA* (**Section S.4.1**) by retaining the full conditional distribution of each response variable. Samples were randomly drawn from the conditional distribution with replacement equal to the number of iterations. Sample values that were more than 1.5 times the interquartile range from the upper and lower quartiles (i.e., outliers) were replaced with randomly sampled non-outlier values to avoid biasing the probabilistic results, for instance by having an overly large influence on the mean value of bounded variables. Occasionally random samples of the predictions for formal recycling (tC2i), other recovery (tC2ii) and incineration (tC2iii) summed to over 100%. To ensure mass balance in the material flow analysis these values were normalised to 100%.

A demonstrable example of the need for this correction of outliers would be our sampling of collection coverage (tC1) for an affluent urban municipality in a HIC. The input data related to such a municipality would suggest a collection coverage of 100%, and indeed the random forest predictions may predict 100% collection coverage in most samples. However, a few predictions may be below 100%, perhaps because an influential independent variable was not randomly selected during decision tree construction (remembering quantile regression forest retains the full

conditional response). In this example, these few predictions would slightly reduce the mean collection coverage. However, because emissions are sensitive to collection coverage in our model (**Section S.10**), they would be overestimated in some cases. We argue this phenomenon is an inevitable artifact of the stochastic nature of the quantile regression random forest and probabilistic material flow analysis. We therefore believe that the correction is valid. It should be noted that when genuine uncertainty exists in a variable's predictions (e.g., the predictions of collection coverage for a municipality have high variance), the interquartile range would be large therefore the number of outliers would likely be minimal, and this correction would have negligible impact.

The correction for outlier values was applied to all *primary input variables* except for waste generation rate ($tP1_{pc}$) and controlled disposal (tC3). For waste generation rate, a density function of the full conditional response was estimated using the '*density*' function in R and assuming a bandwidth determined by the 'nrd0' method and a Gaussian smoothing kernel. Samples were then randomly drawn from the density function and outliers removed as with other *primary input variables*. This adapted method, applied to the waste generation rate, has the advantage that predictions do not necessarily have to be the same as those supplied in the training data, but instead can vary according to the fitted density function. On the other hand, this approach was not applied to the other *primary input variables* as they are percentages between 0% and 100%, and often have a high frequency of values located near the bounds. For example, many of the data for incineration had a value of 0%, whereas many of the collection coverage values were reported as 100%. Fitting a Gaussian density function to these values would assign high probabilities to the values approaching the bound, leading to these being sampled to a greater extent. Referring again to the example of collection coverage, when most predictions for a municipality equalled 100%, practically this meant values of 99% and above were sampled instead of 100%. Although this was a small difference, even small amounts of uncollected waste can have big implications on the overall emissions predicted; therefore, this approach was avoided.

Of the 361 *primary input data* points for controlled disposal of MSW (tC3), 303 (84%) were either 0% or 100%. This meant that the full conditional response of predictions often spanned the entire range as it was highly probable that at least some trees in the random forest would predict both bounds. To avoid artificially high uncertainty of predictions, as would be the case with a bimodal distribution of the data, the prediction was treated in a similar manner to a classification problem whereby the majority result was used. This meant that uncertainty was not predicted for the uncontrolled disposal variable, however, it resulted in relatively high accuracy with 82% of the predicted values matching the actual value for the random forest test dataset. If this approach had not been used, and instead the full conditional response used as with other *primary input variables*, many of the iterations of the probabilistic MFA would have artificially predicted uncontrolled disposal in countries where this is highly unlikely and vice versa.

The absence of uncertainty quantification for controlled disposal of MSW (tC3) *primary input variable* means that readers should treat this result with caution and not interpret our lack of uncertainty as conviction of the result. In particular, results for specific municipalities in LIC and LMC may occasionally be misclassified as uncontrolled (**Table S37**). We postulate this occurs in municipalities that have received official development finance for construction of a sanitary landfill, and therefore have controlled disposal levels beyond what the socioeconomics of that

municipality would usually suggest. Whether these sites continue operating in controlled manner or revert to uncontrolled techniques is also unclear, as are the very definitions of ‘controlled’ in many of our sources of *primary data input* (**Section S.5.**). Methods such as the UN-Habitat[6] Waste Wise Cities Tool should reduce this uncertainty in future data collection efforts by using standardised definitions, however, at present ‘controlled’ often has to be taken at face value.

Caution is also advised for results of controlled disposal in municipalities of UMC’s as these are often at the point in their development where they transition to environmentally sound practices, and therefore, the UMC income category demonstrates the largest variation in levels of controlled disposal. Whilst this variation makes predictions challenging, the results match the test data reasonably well (**Table S37**). Confidence in the controlled disposal result for municipalities in HIC’s is also high given all eight cases of the test data were successfully predicted.

Table S37. Summary of test dataset cases misclassified for the controlled disposal (tC3) variable when using the majority result of random forest predictions.

Income category	Number of test dataset cases	Number of test dataset cases misclassified	Percentage of test dataset cases misclassified
HIC	8	0	0.0%
UMC	16	2	12.5%
LMC	33	5	15.2%
LIC	15	2	13.3%

S.9.1.2 Correction of primary input variable predictions by settlement typology

Waste management data collection in rural areas of the Global South is a largely neglected endeavour, despite evidence that rural areas have generally poor waste management services and are a source of plastic pollution[359]. As a consequence, most of our *primary input data* were obtained from urban areas (**Section S.6.1-S.6.2**), meaning rural areas were under-represented in our dataset. Given this data paucity, it was infeasible to expand the *primary input data* to include more rural areas. Instead, we corrected each randomly sampled prediction (V_u) using a sub-model (**Fig. S26**).

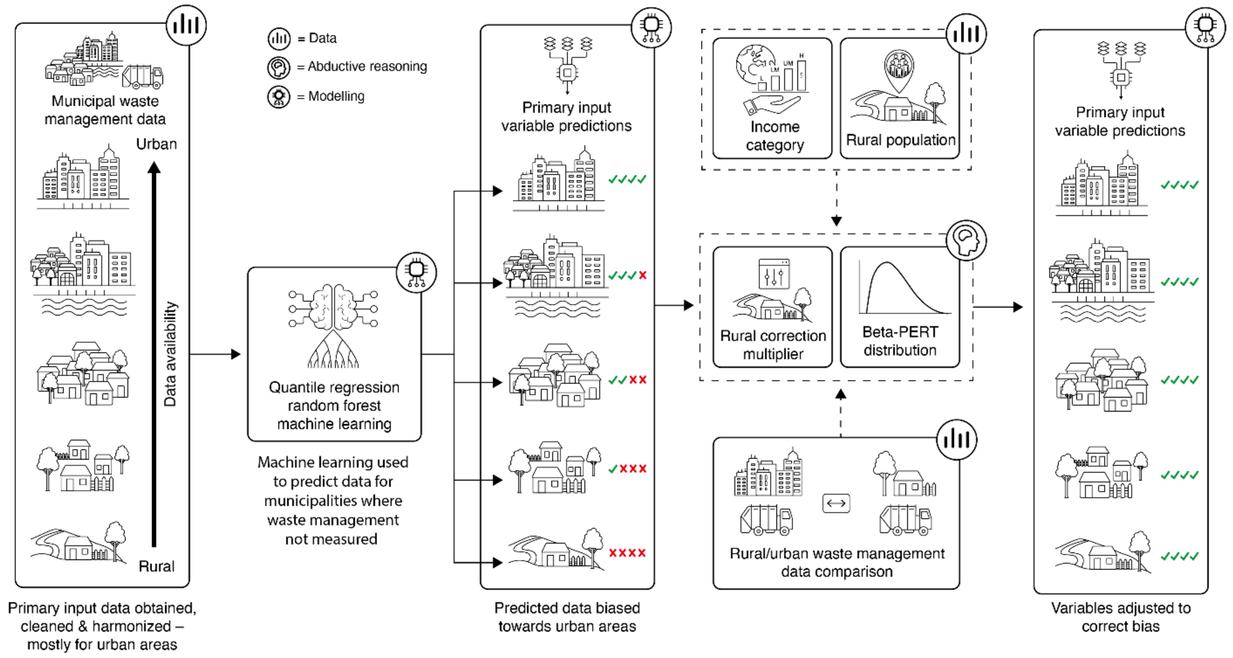

Fig. S26. Sub-model used to correct prediction bias in rural municipalities using correction multipliers based on income category and settlement typology.

We applied **Equation S5** to each *primary input variable* as listed in **Table S38**. Similar corrections have been made in other works [5,182].

$$V_r = V_u \times \left(1 - \left(\frac{Pop_{r,\%}}{100}\right) \times (1 - CF_r)\right) \qquad \textbf{Equation S5}$$

Where:

- V_r is the *primary input variable predictions* after correction for settlement typology
- V_u is the *primary input variable predictions* prior to correction for settlement typology (**Section S.9.1.1**)
- $Pop_{r,\%}$ is the rural population as a percentage of the municipality's population (**Section S.7.1**)
- CF_r is the rural correction multiplier as randomly sampled from the distributions and parameters outlined in **Table S38**.

Table S38. Correction multipliers were used to adjust randomly sampled predictions for selected variables in rural administrative areas. Parameters 1,2 and 3 for Beta-PERT distributions are the minimum, most likely, and maximum respectively, with a default shape factor of 4 used in all cases. For normal distributions, Parameters 1 and 2 are the mean and standard deviation respectively.

ID	Variable name	Income category	PDF	Parameter 1	Parameter 2	Parameter 3
$tP1_{pc}$	MSW generation rate	HIC	Beta-PERT	0.95	1.08	1.15
		UMC	Normal	0.62	0.21	-
		LMC	Normal	0.47	0.25	-
		LIC	Normal	0.47	0.25	-
C0	Plastic in MSW	HIC	Beta-PERT	0.9	1.00	1.00
		UMC	Normal	0.73	0.36	1.00
		LMC	Normal	0.69	0.38	-
		LIC	Normal	0.69	0.38	-
tC1	Collection coverage	HIC	Beta-PERT	1.00	1.00	1.00
		UMC	Beta-PERT	0.43	0.53	0.63
		LMC	Beta-PERT	0.36	0.46	0.56
		LIC	Beta-PERT	0.44	0.54	0.64
tC2i	Formal collection of MSW for dry recycling	HIC	Beta-PERT	0.90	1.00	1.00
		UMC	Beta-PERT	0.40	0.50	0.60
		LMC	Beta-PERT	0.00	0.00	0.00
		LIC	Beta-PERT	0.00	0.00	0.00
tC2ii	Formal collection of MSW for other recovery	HIC	Beta-PERT	0.90	1.00	1.00
		UMC	Beta-PERT	0.40	0.50	0.60
		LMC	Beta-PERT	0.00	0.00	0.00
		LIC	Beta-PERT	0.00	0.00	0.00
tC2iii	Formal collection of MSW for incineration	HIC	Beta-PERT	0.90	1.00	1.00
		UMC	Beta-PERT	0.00	0.00	0.00
		LMC	Beta-PERT	0.00	0.00	0.00
		LIC	Beta-PERT	0.00	0.00	0.00
tC3	Controlled disposal of MSW	HIC	Beta-PERT	1.00	1.00	1.00
		UMC	Beta-PERT	0.90	1.00	1.00
		LMC	Beta-PERT	0.00	0.00	0.00
		LIC	Beta-PERT	0.00	0.00	0.00

Abbreviations: Low-income country (LIC); high income country (HIC); lower middle-income country (LMC); upper middle-income country (UMC); municipal solid waste (MSW).

The correction in **Equation S5** scales the *primary input variable predictions* according to the percentage of the population in each municipality that is classed as rural (**Section S.7.1**) and a *primary input variable* specific correction multiplier (with uncertainty accounted for by representing this as a PDF and randomly sampling from it). The parameters of the rural correction multiplier PDFs for each *primary input variable* are shown in **Table S38** and justified in **Sections S.9.1.2.1-S.9.1.2.6**.

S.9.1.2.1 MSW generation rate (tP1$_{pc}$)

MSW generation rates (tP1$_{pc}$) are thought to vary according to rurality (degree of urbanisation), however the data to evidence this is limited. It is widely assumed that in the Global South, waste generation in rural areas is less, for example both Hoornweg and Bhada-Tata[360] and Kaza, et al.[31] assumed it is approximately 50% less than in urban areas whilst acknowledging that the data to support such an assumption are sparse. This is also supported by much of the data reported in Karak, et al.[361], although considerable variation around this value was demonstrated depending on the case study. On the other hand, Lau, et al.[5] assumed no difference between waste generation in rural and urban areas of HICs and Hidalgo, et al.[362] found only non-significant differences in Spain.

High income countries

For HICs, we classified UK local Unitary and Collection Authorities by Level 1 settlement typology using the GHS-DUC[195] results for GADM V4.1[363], ignoring any blanks due to differences between the local authority and GADM boundaries. We summed local authority collected waste reported by Defra[364] and divided it by the GHS population for 2020[195] to express on a per capita basis.

Rural areas generated approximately 7.6% (central estimate) more waste compared with urban areas. Analysis of the same dataset[364] shows that this difference is largely due to higher rates of 'green' waste (garden/ yard waste) which were 57% higher in rural areas compared to urban areas, accounting for 24% and 18% of household waste generation respectively. We assumed a Beta Pert distribution with a shape factor of 4, an upper limit that was double the central estimate (15%) and rounded the central estimate to 8% (**Table S38**). For the lower limit, we assumed a slightly lower waste generation rate on the basis that the UK is unlikely to be typical for all HICs and that many of them will have lower rural waste generation rates.

Low- and middle-income countries

Robust and granular waste generation data such as that analysed for the UK was not available for countries in the Global South. Therefore, we collected 40 data points (13 from UMCs, 26 from LMCs, and 1 from LICs) from 13 studies[365-377] of 11 countries, where rural waste generation was reported. For 11 of the data points, urban waste generation was calculated so we were able to calculate a ratio directly. For the remaining 29 data points, we calculated the ratio between rural waste generation and the mean urban waste generation for that country from our own cleaned *primary input data*. We grouped countries by income category and calculated the mean and standard deviation for each, assuming a normal distribution for the model input (**Table S38**). As there was only one data point for LICs, we merged LIC and LMC categories.

S.9.1.2.2 Plastic in MSW (C0)

Little data exist to evidence a difference in plastic composition between rural and urban areas in HICS. Lebreton and Andrady[182] also found no statistically significant relationship between per capita GDP and the proportion of plastic in MSW. It is unclear if this lack of relationship with GDP also applies sub-nationally; however, we argue that the amount of plastic in MSW may be lower in rural areas compared to those in cities because of higher proportions of Green (garden yard) (**Section S.9.1.2.1**).

For HICs, we assumed plastic compositions the same as those for urban areas, as the central and maximum estimates (highly unlikely they produce more plastic as % in rural than urban). We chose the lower bound of the BETA-PERT distribution to be 0.9, as HIC may produce more garden waste (**Table S38**). For LMICs, we carried out the same analysis as described in **Section S.9.1.2.1**, using a sub-set of nine of the same articles[365-369,372-374,377] (which reported plastic waste composition).

S.9.1.2.3 Collection coverage (tC1)

Kaza, et al.[31] reported that urban areas have higher collection coverage than rural areas, with this also depending on the income level of the country. HICs for instance had rural collection coverages almost comparable to urban levels (98% of urban collection rate). This proportion decreases for UMCs to 53% of urban collection rates, 46% in LMCs and 54% in LICs (equivalent to 26% rural collection coverage in LICs). These factors were used as the central estimates for the collection coverage settlement typology correction multipliers with ±10% assigned as the uncertainty in all income groups except HIC. For HICs, no correction was made to the predicted values to account for settlement typology as applying the 0.98 multiplier from Kaza, et al.[31] would likely lead to an unrealistic overestimation of uncollected waste in HICs.

S.9.1.2.4 Formal collection of MSW for dry recycling (tC2i) and other recovery (tC2ii)

Both formal collection of MSW for dry recycling (tC2i) and formal collection of MSW for other recovery (tC21ii) were assigned the same settlement typology correction multipliers. This assumed that HICs have the resources and regulatory imperative to extend recycling and recovery operations to rural areas (albeit with a lower uncertainty value assigned of 0.9). Conversely, LIC and LMC countries are highly unlikely to have the resources to implement formal recycling or recovery operations in rural areas, as poor road networks and high transport costs create barriers to doing so[359]. As such, a correction multiplier of zero was applied to these LICs and LMCs, thereby assuming that fully rural municipalities (rural population percentage equal to 100%) have no formal recycling or other recovery. For UMCs we assumed more variation as there is evidence that formal recycling and recovery begins to be implemented along with growing resources (**Table S8**, **Table S13**, **Table S15**), and it is therefore plausible that these activities take place in some UMC rural municipalities (particularly if close to an urban centre). Therefore, a correction multiplier of 0.5 with ±0.1 uncertainty was assigned to sit in-between those of HICs and LICs.

S.9.1.2.5 Formal collection of MSW for incineration (tC2iii)

Incineration in HICs was treated the same as for formal dry recycling and other recovery; however, all other income categories were assigned a settlement typology correction multiplier of zero. Further correction to the incineration data is discussed in **Section S.9.1.2.7**.

S.9.1.2.6 Controlled disposal of MSW (tC3)

No settlement typology correction was applied to controlled disposal in HICs due to regulations often enforcing controlled disposal regardless of their settlement typology, for example Directive 1999/31/EC[378]. A similar assumption was also applied to UMC (albeit with a lower uncertainty value of 0.9), whereas both LMC and LIC had a value of zero assumed for the settlement

typology correction multiplier. Notably, a settlement typology correction multiplier of one does not mean all predictions of controlled disposal are classed as controlled, but instead that the original prediction for the municipality is not altered based on its settlement typology. Accordingly, municipalities in both HICs and UMCs can still be predicted to have uncontrolled disposal.

S.9.1.2.7 Replacement of primary input predictions for formal collection of MSW for incineration (tC2iii)

Both the training and test datasets were generally effective at distinguishing between municipalities which incinerate waste compared to those that do not. However, in a few cases, the *primary input predictions* suggested that a municipality does not incinerate its waste when in fact it does and vice versa.

To correct these anomalies, we used data from OECD[379], Eurostat[380], Ding, et al.[381], and Lu, et al.[382] to assess which countries report more than 1% of their municipal solid waste being incinerated between 2017 and 2020. These were: Austria, Belgium, Canada, China, Croatia, Cyprus, Czech Republic, Denmark, Estonia, Finland, France, Germany, Greece, Hungary, Iceland, Ireland, Israel, Italy, Japan, Latvia, Lithuania, Luxembourg, Netherlands, Norway, Poland, Portugal, Romania, Singapore, Slovak Republic, Slovenia, South Korea, Spain, Sweden, Switzerland, Taiwan, United Kingdom, and United States. We removed predictions for countries reporting less than 1%, assuming that their incinerators were being used to treat hazardous or healthcare waste, neither of which are relevant to the model.

Although there are some incinerators in cities that are not within the countries above, for example, Kyiv in Ukraine has an incineration plant that handles around a quarter of Kyiv's solid waste[383], these countries were purposely not included in the above list. This was to avoid potentially accepting predictions of incineration throughout the whole country when incineration is not widespread.

In the case of China, incineration as a percentage of collected waste was taken directly from the MoHURD dataset[34] and replaced any predictions, as discussed in **Section S.6.4.6.2**. None of the other *primary input variables predictions* were replaced with actual data to ensure consistency in the year of the outputs (2020).

S.9.1.3 Sampling of *secondary data inputs*

Secondary data inputs were sampled according to the probability density functions and parameters as described throughout **Section S.8**, each of which was randomly sampled 5,000 times. A summary of all *secondary data inputs* is shown in **Table S3**.

S.9.2 Material flow analysis

Material flow analysis was carried out for the system maps shown in **Fig. S4 - Fig. S8** according to the equations described in **Supplementary Data 2** and across all 50,702 global municipalities. The probabilistic Monte Carlo analysis approach meant that each municipal MFA result had 5,000 iterations to represent uncertainty. As such, a large amount of raw output data was generated. Ideally, the full set of raw data outputs would have been retained to assess the

probability density functions of all outputs, however, this was too computationally demanding. Instead, the raw results for each iteration were retained for only select municipalities, as specified in *Model Inputs*[384]. These are used to demonstrate the variability and shape of distributions of per capita plastic emissions as shown in **Figure 3**. All raw results were retained for national and international aggregations because the lower number of groupings were within computational capability. For easier interpretation and comparability, all results were summarised by their 5th percentile, lower quartile, median, mean, upper quartile and 95th percentile values as displayed in **Supplementary Data 3**, **4**[384].

In total, for each of the 50,702 municipalities, 81 processes and 42 transfer coefficients were quantified. An additional 59 outputs were also calculated from these results, such as total emissions into the environment, or the number of people without waste collection services. Outputs relate to values calculated from the processes or coefficients, for instance, the summation of all emission source processes to give the overall emissions or the division of an emission source by the overall emissions to represent it as a percentage. To represent the uncertainty of outputs (e.g., by percentiles), these calculations had to be performed on the raw results of 5,000 iteration rather than on the summarised results. As such, we caution the reader against calculating their own outputs based solely on the summarised data. If further outputs are required, all data and code required to run the model are available to download from Dryad [384].

S.9.2.1 Spatial aggregation

A unique aspect of the method described here is that we used a bottom-up approach. Our model simulations run at municipal scale and are then aggregated to national, regional (UN or OECD regions) and World Bank Income Category scale. A core requirement of this step was to aggregate the full distribution of municipal results in a way which reflected the spatial dependency between municipalities within each of the aggregated areas. This means that the variation in municipal simulation results must be reflected in the national aggregated results, acknowledging the similarity between waste generation and management within municipalities as a result of national regulations, policies, or cultural behaviour.

The mass of waste generated (P1) in municipalities for each iteration within a country were extracted and reordered according to the method by Iman and Conover[385] to induce rank correlation. Ranking in this way, retains the distribution of predictions but reorders them so that they approximate a specified correlation, which in this case is used to represent spatial dependency[386]. A correlation coefficient of 0.5 was used for the off-diagonal cross-correlation matrix as similarly applied by models focussed on aggregation within water distribution networks[387,388]. The rank order calculated for waste generation was used to reorder the iterations of all the other processes within each MFA to ensure the mass balance of each iteration was retained. The processes for each municipality within the country were then summed separately for every iteration to arrive at 5,000 MFAs at national level. These were summarised in the same manner as municipal results as described in **Section S.9.2**. Once the probabilistic MFA was completed for all countries, the national level results were aggregated to higher spatial levels, namely UN regions (including sub-regions and intermediate regions)[193], OECD regions[389], income categories[86] and globally**.** These were then summarized in the same manner as for the municipal and national level MFAs. All summarized results are available in **Supplementary Data 3**, **4** and available from Dryad - https://doi.org/10.5061/dryad.8cz8w9gxb[384].

An overview of the probabilistic material flow analysis approach is shown in **Fig. S27**.

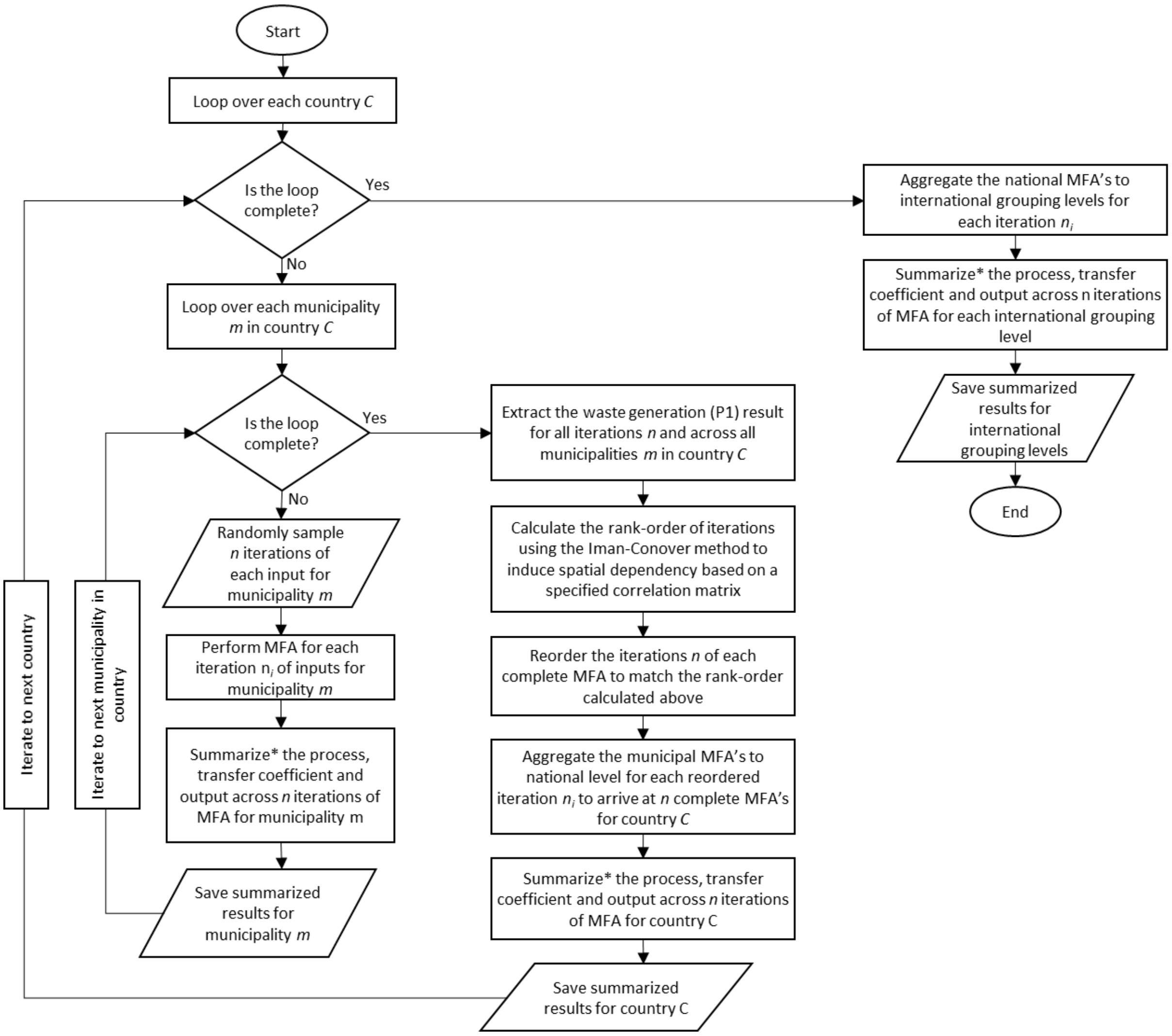

Fig. S27. Procedure for probabilistic material flow analysis with aggregation to higher spatial scales and incorporating spatial dependency up to the national level. *Summary statistics are the mean, median, lower quartile, upper quartile, 5th percentile and 95th percentile.

S.9.2.2 Uncertainty

There are two types of uncertainty in our model: **(1)** Aleatoric uncertainty - observable differences in solid waste management practices between municipalities that may or may not have similar socio-economic characteristics (real world variability); and **(2)** Epistemic uncertainty – due to lack of knowledge that could in principle be known (e.g. lack of data to train the model for a particular set of geographical, socio-economic, or political conditions). In practice, our uncertainty quantification incorporates both types of uncertainty. We have discussed the methods for uncertainty quantification throughout the Supplementary Methods, however we present a summary in this section for ease of reference. Additionally, we note that epistemic uncertainty does not just occur due to lack of data, but also because our model requires simplification of complex real-world phenomena as part of its formulation process. Whilst we

attempt to address model assumptions as part of the sensitivity analysis (**Section S.10**), some epistemic uncertainty undoubtedly remains unquantified.

A core component of incorporating the probabilistic MFA into our approach was the ability to propagate the uncertainty around input parameters into the model outputs across different spatial scales. All inputs to the probabilistic MFA were assigned a PDF which is intended to incorporate both the aleatoric and epistemic uncertainty (**Supplementary Data 6**). These PDFs quantitatively demonstrate the range and distribution (e.g. likelihood) of results simulated by the random forest model for *primary input data* variables (**Section S.7**) or attributed by the sub-models (**Section S.8**) for secondary *input data variables*. As the probabilistic MFA is a bottom-up model, these PDFs are specified for every input and municipality. The exception to this is that PDFs for some *secondary data inputs* were assigned to aggregated groupings, such as income categories, due to the lack of more granular data (**Table S2** and **Table S3**). Likewise, uncertainty was not predicted for the controlled disposal coefficient (tC3) due to limitations of the quantile regression random forest predictive capability (**Section S.9.1.1**).

The probabilistic MFA uses Monte Carlo analysis to iteratively random sample each input PDFs 5,000 times per municipality (**Section S.9**). These sampled inputs are used to calculate 5,000 separate MFAs (iterations) for each municipality. These are summarised for reporting (**Supplementary Data 5**) according to the mean, 5th, 25th, 50th, 75th, and 95th percentiles, however, occasionally the full distribution of outputs is presented (**Figure 3**).

Uncertainty was propagated from the municipal level to national level by summing MFA processes for all municipalities in a country (**Section S.9.2.1**). This was done for each iteration to arrive at 5,000 separate national MFAs. Spatial dependency was induced during this aggregation by using the Iman and Conover[385] method. This involved reordering the iterations of the waste generation process (P1) so that they had a specified correlation of approximately 0.5. The rank-order of these iterations was then transferred to all processes in the MFA to ensure the mass balance of each MFA was retained. The results of each process in each of the 5,000 iterations for each municipality was then summed to national level.

To aggregate to other international spatial scales (e.g. by income categories, UN regions, or globally), the previously aggregated national level results were further aggregated by summing the processes of each national MFA to the relevant aggregation level for all iterations, however, no spatial dependency was assumed for this stage. As with the municipal level results, the full distribution of results were summarised for reporting by calculating the mean, 5th, 25th, 50th, 75th, and 95th percentiles (**Supplementary Data 3-4**).

The bottom-up spatial aggregation of municipal level results causes a decrease in the relative uncertainty (coefficient of variation) at aggregated levels. This is because the extremities of distributions cancel each other out as they are summed together. For example, if we assume a normally distributed input where a high outlier value was sampled from the tail of the distribution. The probability that all other municipalities in that iteration of the spatial aggregation also had a high outlier value sampled decreases as the number of municipalities increases. As such, the spread (relative uncertainty) of the aggregated distribution decreases as the number of municipalities in a spatial aggregation increases.

S.10 Sensitivity analysis

In the absence of measured data to validate emissions into the environment, we carried out sensitivity analysis[390], an approach also carried out by Lau, et al.[5]. Two forms of sensitivity analysis were assessed: (1) Sensitivity of model outputs to model inputs (**Section S.10.1**); and (2) Sensitivity of model outputs to modelling assumptions (**Section S.10.2**)

S.10.1 Sensitivity of model outputs to model inputs

The Sobol method for sensitivity analysis is a global sensitivity variance-based approach suitable for non-linear models[391]. We applied the *sobolmartinez* function within the R-package *sensitivity* version 1.28.1 for Monte Carlo estimation of Sobol' indices using 10,000 iterations. Both first order (main effect) and total effect indices were estimated. Main effect indices demonstrate the influence one input parameter has on the output, whereas the total effect indices demonstrate the impact that an input parameter has on the output (plastic emissions), including all higher-order interactions.

Sobol indices were estimated individually for each of the 50,702 municipalities and all uncertain inputs. To summarise each of these sensitivity analysis results, we aggregated the first and total order indices across all municipalities by calculating the mean value, weighted by the total emissions of the municipality (**Fig. S28**) Inputs with a total effect <0.01 were removed because they have negligible influence of plastic emissions.

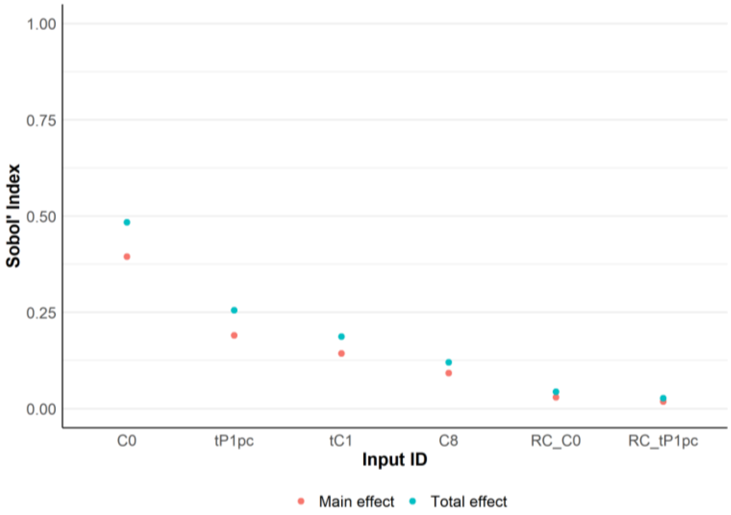

Fig. S28. Main effect and total effect Sobol' indices for total plastic emissions aggregated to the global scale by calculating the mean value weighted by plastic emission. Abbreviations: C0 = plastic in MSW (% of generated MSW), tP1pc = Waste generation rate per capita ($kg{\cdot}cap^{-1}{\cdot}d^{-1}$), tC1 = MSW collection coverage (% of generated MSW), C8 = open burning of uncontrolled disposal (% of uncontrolled disposal), RC_C0 = Settlement typology correction multiplier for plastic in MSW, RC_tP1_{pc} = Settlement typology correction multiplier for waste generation rate.

Six input parameters were found to have an influence on the quantity of plastic emissions, having Sobol indices >0.01. The three most influential of these (C0, tP1pc, and tC1) came from our

cleaned *primary input data* and relate to parameters that can be physically measured and validated, and for which we have obtained numerous data points. As such, we have greater confidence in these parameters and accordingly in the model outputs. Similarly, the parameters used for correcting waste generation rate (RC_$tP1_{pc}$) and plastic percentage (RC_C0) by settlement typology were also influential, albeit to a lesser extent. It is self-evident that these inputs that affect the overall mass of plastic in the system will influence plastic emissions. It also stands to reason that collection coverage (tC1) is highly influential, in agreement with the results of Lau, et al.[5] (though they did not explicitly highlight), because waste collection process takes place early in the system and because uncollected waste (the complement of collection coverage) results in a sparsely distributed mass which is considered an e mission in its entirety.

Open burning at uncontrolled disposal sites (C8) is influential because of the direct impact it has on calculating emissions, and because of the large mass of material that flows through that part of the model. Land disposal is still the predominant system endpoint for solid waste worldwide[31] and therefore it is unsurprising that our model is sensitive to it. We postulate that controlled disposal (tC3) itself is also a highly influential parameter. However, due to the classification problem highlighted in **Section S.9.1.1** and subsequent corrections, no uncertainty was applied to controlled disposal (tC3) meaning we could calculate a Sobol index for it.

In addition to the globally aggregated sensitivity analysis of municipal level simulations, we also aggregated municipal level Sobol indices to income-category scale (**Fig. S29**) This aggregation used the mean of the Sobol indices weighted by plastic emissions, as per the global analysis. The results for LIC, LMC and UMC broadly matched those of the global analysis (**Fig. S**28) with the same six influential parameters (C0, $tP1_{pc}$, tC1, C8, RC_C0, RC_$tP1_{pc}$).

The correction of collection coverage by settlement typology (**Section S.9.1.2**) in UMCs was also highlighted as influential, albeit with a low Sobol index (0.015). This is likely to be influential because municipalities in this income category have begun to approach full collection coverage, therefore any adjustments to rural coverage may result in a large increase in emissions from uncollected waste.

The income category level results for the HIC aggregation were notably different to other income categories in that the settlement typology correction multipliers and open burning at uncontrolled disposal were no longer influential. We postulate this is because HICs have relatively well-functioning waste management systems, even in rural areas, and because the majority of disposal is controlled and therefore has negligible open burning. Unlike other income categories, three additional parameters were also influential on plastic emissions in HIC. These were the street sweeping efficiency (S), emissions from the collection system prior to street sweepings (C3i), and the littering rate (LT). Street sweeping efficiency in particular was highly influential, with only the waste generation rate higher. The influence of these inputs highlights the stark differences between the causes of plastic pollution in HICs compared to the Global South, the former of which is related to comparatively small emissions from littering and escape from the collection system, and the latter of which is predominantly a result of uncollected waste.

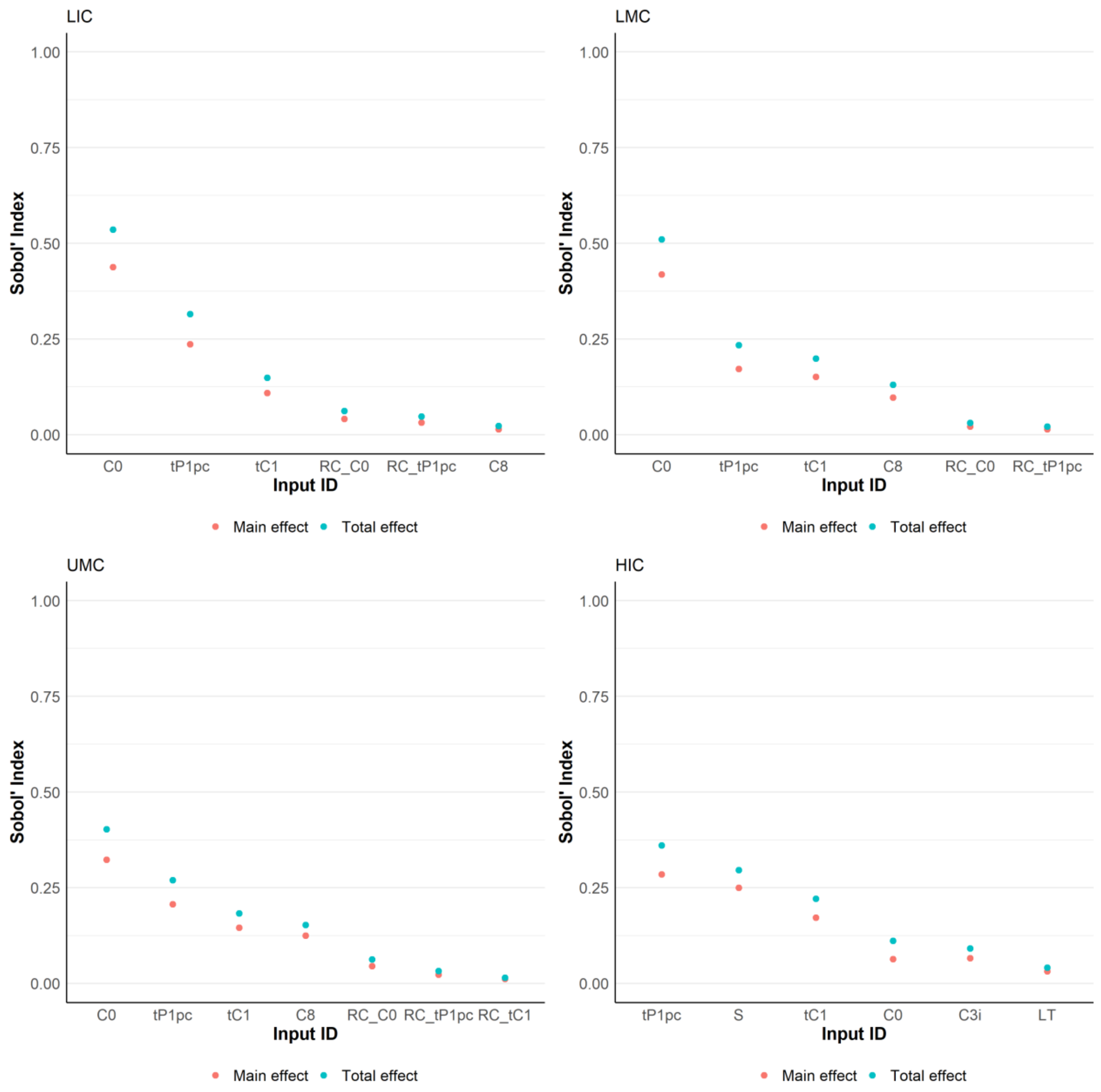

Fig. S29 . Main effect and total effect Sobol' indices > 0.01 for total plastic emissions aggregated according to the income-categories by calculating the mean value weighted by plastic emission. Abbreviations: C0 = plastic in MSW (% of generated MSW), tP1pc = Waste generation rate per capita (kg·cap^{-1}·d^{-1}), tC1 = MSW collection coverage (% of generated MSW), C8 = open burning of uncontrolled disposal (% of uncontrolled disposal), RC_C0 = Settlement typology correction multiplier for plastic in MSW, RC_tP1$_{pc}$ = Settlement typology correction multiplier for waste generation rate, RC_tC1 = Settlement typology correction multiplier for collection coverage, S = street sweeping efficiency (%), C3i = emissions from the collection system prior to street sweepings (% of collected waste), LT = littering rate (% of MSW generation).

To compare the sensitivity analysis results with the level of confidence in our input parameters, we assigned data pedigree to each relevant model coefficient based on the level and quality of

measured data used to populate or model it (**Table S39**). The input parameters that were often the most influential are those relating to plastic waste generation and collection ($tP1_{pc}$, C0, tC1), all of which were allocated the highest data pedigree given these are based on our cleaned and harmonised *primary input data* of solid waste management with hundreds of data points each. As such, we can be confident in these influential parameters, and therefore the model outcomes.

On the other hand, we acknowledge that some of the lower ranked influential input parameters were allocated poor data pedigrees, for example, open burning at uncontrolled disposal sites (C8) and the settlement typology correction multipliers for waste generation (RC_tP1_{pc}) and plastic (RC_C0). Likewise, in HICs, no/limited data was available for the efficiency of street sweepings (S), emissions from the collection system (C3i) and the total littering rate (LT). As such, we recommend data collection efforts in the future to focus on improving the availability and quality of data surrounding these input parameters, particularly in the HIC context where they are more influential. Despite this, the absence of reliable data for these inputs does not remove our confidence in the overall results as any uncertainty in inputs can be accounted for in the probabilistic approach, we take for the material flow analysis. For example, the PDF we assume for open burning at uncontrolled disposal sites has a large range of between 0% and 80.2% to acknowledge the wide uncertainty that exists for this input. Furthermore, as shown in **Section S.10.2**, the model outputs are not sensitive to any assumptions we make.

Table S39. Data pedigree of input parameters. Pedigree is assigned from one (best) to four (worst). A pedigree of one was allocated if multiple data points were available (>40) to construct the input probability density function and this data was from reliable sources and based on physical measurements. A pedigree of two was allocated if the same conditions as pedigree one were met, but if fewer data points were available. A pedigree of three was allocated if data was available, but from limited or potentially unreliable sources, and a pedigree of four was for when no data was available.

Input parameter	Sub-parameter	Input ID	Notes	Data points	Pedigree
Waste generation rate	NA	$tP1_{pc}$	Data from primary input sources. Many data points with wide geographical distribution give these parameters high pedigree.	468	1
Collection coverage	NA	tC1		382	1
Formal sector collection of MSW for dry recycling	NA	tC2i		357	1
Collected MSW for recovery	NA	tC2ii		346	1
Collected MSW for incineration	NA	tC2iii		375	1
Controlled disposal of MSW	NA	tC3		364	1
Plastic in MSW	NA	C0		334	1
Plastic in MSW	HIC	C0a	Limited data as from WaCT only, however available data is reliable	5	2
	UMC			7	2
	LMC / LIC			30	2
Number of waste pickers	HIC	WP	Data from multiple secondary sources, however HIC data is limited to South America and LIC to Africa	4	2
	UMC			45	1
	LMC			49	1
	LIC			4	2
Waste pickers productivity	NA	WP_p	Data from 18 municipalities	18	2
Debris emissions from collection system prior to street sweeping	NA	C3i	Assumed emission factors with magnitude dependent on quality of collection	0	4
Open burning of uncontrolled disposal of MSW	NA	C8	Data from a single source – likely unreliable as difficult to measure	1	3

Input parameter	Sub-parameter	Input ID	Notes	Data points	Pedigree
Debris emissions from uncontrolled disposal of MSW	NA	C9	Emission factors assumed but related to dumpsite topography and exposed material.	0	4
Uncollected MSW open burnt	HIC	C10	Data from 44 countries but many of these come from census data therefore deemed reliable. 2 PDF's used for each income group to account for urban and rural behaviours.	3	2
	UMC			17	2
	LMC			14	2
	LIC			10	2
Plastic in uncollected litter	NA	C11	Secondary data source from single country (Wales), however multiple data points.	4	3
Plastic in debris emissions from uncontrolled disposal of MSW	NA	C14	Assumed	0	4
Material collected by informal sector that is plastic	HIC	C15	Assumed	0	4
	LMIC		Informed based on secondary data sources	30	3
Plastic collected by formal sector	NA	C16	Based on UK data from Defra – deemed reliable but lower pedigree assigned as from only a single country and original value did not state any uncertainty	1	3
Rigid plastic in uncollected litter	NA	C11a	Secondary data source from single country (Wales), however multiple data points.	4	3
Rigid plastic in uncontrolled disposal plastic emissions	NA	C14a	Assumed	0	4
Rigid plastic in plastic collected by informal sector	NA	C21a	Data from secondary data sources with 11 municipalities across 5 countries represented	11	2
Rigid / Flexible plastic sorting losses by informal / formal sector	NA	C23aa, C23ab, C24aa, C24ab	Data 6 data sources used within a sub-model that looked at plastic polymers collected for recycling and linked to rejects at reprocessors	6	3
Littering rate (collected)	NA	L_C	Based on 5 sources but all from Europe.	5	3
Street sweeping efficiency	Income level and degree of urbanisation	S	Assumed	0	4
Settlement typology correction multiplier – Waste generation rate	HIC	RC_tP1	24 rural data points for municipalities in the UK, but no other HIC represented therefore assigned lower pedigree	24	3
	UMC		Although based on multiple data points from multiple countries, the reliability is low	13	3
	LMC / LIC			26	3
Settlement typology correction multiplier – Plastic in MSW	HIC	RC_C0	Assumed	0	4
	UMC		Secondary data sources but deemed low reliability so pedigree reduced	6	3
	LMC			11	3
Settlement typology correction multiplier – Collection coverage	Income level	RC_tC1	Based on analysis in WaW2. Unclear number considered rural therefore lower pedigree	?	3
Settlement typology correction multiplier – treatment and disposal	Income level	RC_tC2, RC_tC3	Assumed	0	4

S.10.2 Sensitivity of model outputs to modelling assumptions

The sensitivity analysis described in **Section S.10.1** assessed which input parameters are most influential on our model's outputs according to their PDFs and associated uncertainty. Although these PDFs were derived from the best available data, we acknowledge that for some, the quality of input data was less robust, and in others there were no measurements meaning we had to rely on abductive reasoning.

To test the reliability of the PDFs, we tested each of the inputs with a data pedigree of three (**Table S39**) by converting its PDF to a uniform distribution between the previously specified range, and therefore removing any assumptions about the central location. We did not consider inputs with higher data pedigree given we have sufficient data to be reasonably confident that their PDFs are representative.

The same approach was also used for inputs with data pedigree of four. However, because we had used abductive reasoning based on limited or no data to derive these PDFs, we also extended the ranges to test the impact on the model. The expansion of the ranges differed per input as shown in **Table S40**. We did not alter ranges to physically impossible values (e.g., percentages >100%), or those we would consider implausible. We then carried out global sensitivity analysis using the Sobol method in the same manner as that described in **Section S.10.1**.

The results of this second sensitivity analysis (**Fig. S30**), termed here the 'counterfactual sensitivity analysis results', were virtually identical to those of **Section S.10.1**, except for a few minor differences. The input for open burning of uncontrolled disposal (C8) was generally ranked as more influential compared to the first sensitivity analysis, although it still was not as influential as plastic in MSW (C0) or waste generation ($tP1_{pc}$). The increase in the Sobol index of C8 is unsurprising given it was allocated a large range (0% to 80.2%) and the previous most-likely value of 8.6% sits at the lower end of this range.

Other differences between Sobol scores for the first and second sensitivity analysis were in the HIC aggregation only, where a further five parameters were found to be influential. Two of these (C8 and RC_tC3) also relate to open burning at uncontrolled disposal sites, and are as a result of the settlement typology correction multiplier for controlled disposal (RC_tC3) enabling HICs to have uncontrolled disposal in rural areas. Likewise, the settlement typology correction for collection coverage (RC_tC1) was also modified in this sensitivity analysis to enable HIC to have uncollected waste in rural areas and as such was influential. Lastly, the *primary data input variables* relating to formal collection of MSW for dry recycling (tC2i) and waste incineration (tC2iii) were also highlighted as influential, although their Sobol indices were still relatively small. Given these have a high data pedigree, we do not consider these to be of concern.

Whilst Sobol indices allow us to assess the influence of parameters on model outputs, they do not convey the magnitude by which the model outputs change. To assess this variation in modelling results, estimates of plastic emissions from the 'counterfactual sensitivity analysis' were compared with those from our main 'baseline' model that used best estimates of parameters PDFs and central location (**Table S41**).

At the global scale, plastic emissions increased by approximately 30% in the counterfactual sensitivity analysis model to 68 $Mt \cdot y^{-1}$ [62.9-73.7], which we suggest is relatively minor given the context that this counterfactual result had all major assumptions removed. The primary driver of the increased emissions was due to a 47% rise in openly burned plastic, almost all of which is from higher prevalence of open burning at uncontrolled disposal sites (C8), in agreement with the Sobol Indices (**Fig. S30a**). By contrast, global plastic debris emissions increased by only 9% to 24.1 $Mt \cdot y^{-1}$ [22.4-26.0]. Whilst the absolute uncertainty of the counterfactual model results increased slightly, the relative uncertainty remained consistent with those of the baseline, this is despite many of the PDF ranges being extended and assumptions on central location removed.

Disaggregating the results by income category (**Table S41**) highlights that the largest relative change in emissions is for open burning emissions in HICs, with over a 2000% increase. However, the absolute emissions are still negligible (0.28 Mt·y^{-1} [0.24-0.33]) in comparison to other income categories (UMC = 13.9 Mt·y^{-1} [12.6-15.3], LMC = 26.3 Mt·y^{-1} [23.1-30.0], LIC = 3.5 Mt·y^{-1} [3.1-3.8]), therefore do not alter our main findings. Similarly, plastic debris emissions also increased by nearly 300% for HIC's, but as before, these emissions were relatively small (0.6 Mt·y^{-1} [0.5-0.7]), and therefore did not overly influence the global picture. The counterfactual results for UMC, LMC and LIC income categories broadly mirrored those at the global level whereby open burning emissions increased compared to the baseline results, and for which the greatest increase was for LMC income category with open burning emissions 52% higher. As before, this increase is largely driven by higher estimates of open burning at uncontrolled dumpsites (C8).

In conclusion, given the Sobol Indices and plastic emission results of this counterfactual sensitivity analysis are similar to those in **Section S.10.1** and our baseline model, we are confident that any assumptions made to our model inputs do not overly influence the main outcomes of the work. Particular areas we recommend improvements to data collection efforts are waste management in rural areas and open burning of uncontrolled disposal. Likewise, we highlight the caveat that predictions of controlled disposal did not include uncertainty quantification due to the issues discussed in **Section S.9.1.1**, and therefore were omitted from this sensitivity analysis. As we expect this to be a sensitive parameter, we also recommend this as a topic for future improvements.

Table S40. Comparison of original distributions to modified distributions for data inputs with data pedigree of three or four as used in the sensitivity analysis for testing model assumptions (**Table S39**). Numbers in brackets for the distributions represent minimum, most likely and maximum for beta-PERT distributions; minimum and maximum for uniform distributions; and mean and standard deviation for normal distributions.

Input parameter	Sub-parameter	Input ID	Data pedigree	Original distribution	Modified distribution for sensitivity analysis
Debris emissions from collection system prior to street sweeping	NA	C3i	4	Beta-PERT (1, 5, 15)	Uniform (1, 15)
Open burning of uncontrolled disposal of MSW	NA	C8	3	Beta-PERT (0, 8.6, 80.2)	Uniform (0, 80.2)
Debris emissions from uncontrolled disposal of MSW	NA	C9	4	Beta-PERT (0.006, 0.04, 0.45)	Uniform (0.003, 0.9)
Plastic in uncollected litter	NA	C11	3	Beta-PERT (13.8, 17.7, 20.4)	Uniform (13.8, 20.4)
Plastic in debris emissions from uncontrolled disposal of MSW	NA	C14	4	Beta-PERT (40, 50, 60)	Uniform (20, 80)
Material collected by informal sector that is plastic	HIC	C15	4	Beta-PERT (0, 2.3, 4.6)	Uniform (0, 9.2)
	LMIC		3	Beta-PERT (2.3, 30, 60)	Uniform (2.3, 60)
Plastic collected by formal sector	NA	C16	3	Beta-PERT (4.3, 8.5, 12.8)	Uniform (4.3, 12.8)
Rigid plastic in uncollected litter	NA	C11a	3	Beta-PERT (69, 72.9, 76)	Uniform (69, 76)
Rigid plastic in uncontrolled disposal plastic emissions	NA	C14a	4	Beta-PERT (5, 10, 15)	Uniform (0, 30)
Rigid plastic sorting losses by informal sector	NA	C23aa	3	Beta-PERT (15.07, 18.84, 22.61)	Uniform (15.07, 22.61)
Flexible plastic sorting losses by informal sector	NA	C23ab	3	Beta-PERT (11.92, 14.90, 17.88)	Uniform (11.92, 17.88)
Rigid plastic sorting losses by formal sector	NA	C24aa	3	Beta-PERT (32.59, 40.74, 48.89)	Uniform (32.59, 48.89)
Flexible plastic sorting losses by formal sector	NA	C24ab	3	Beta-PERT (46.46, 58.08, 69.70)	Uniform (46.46, 69.70)
Littering rate (collected)	NA	L_C	3	Normal (0.81, 0.15)	Uniform (0.51, 1.11)
Street sweeping efficiency - HIC	Urban Centre	S	4	Beta-PERT (90, 99, 100)	Uniform (70, 100)
	Dense urban		4	Beta-PERT (80, 97.5, 99)	Uniform (60, 100)
	Semi-dense urban		4	Beta-PERT (70, 95, 97.5)	Uniform (50, 100)
	Suburban		4	Beta-PERT (60, 92.5, 95)	Uniform (40, 100)
	Rural		4	Beta-PERT (50, 90, 92.5)	Uniform (30, 95)
Street sweeping efficiency - UMC	Urban Centre		4	Beta-PERT (80, 95, 100)	Uniform (60, 100)
	Dense urban		4	Beta-PERT (50, 80, 85)	Uniform (30, 100)
	Semi-dense urban		4	Beta-PERT (20, 70, 75)	Uniform (0, 95)
	Suburban		4	Beta-PERT (0, 50, 55)	Uniform (0, 75)
	Rural		4	Beta-PERT (0, 20, 25)	Uniform (0, 45)
Street sweeping efficiency - LMC	Urban Centre		4	Beta-PERT (50, 80, 90)	Uniform (30, 100)
	Dense urban		4	Beta-PERT (20, 60, 70)	Uniform (0, 90)
	Semi-dense urban		4	Beta-PERT (0, 20, 30)	Uniform (0, 50)
	Suburban		4	Beta-PERT (0, 10, 20)	Uniform (0, 40)
	Rural		4	Beta-PERT (0, 5, 15)	Uniform (0, 25)
Street sweeping efficiency – LIC	Urban Centre		4	Beta-PERT (0, 20, 30)	Uniform (0, 50)

Input parameter	Sub-parameter	Input ID	Data pedigree	Original distribution	Modified distribution for sensitivity analysis
	Other		4	Beta-PERT (0, 0, 5)	Uniform (0, 15)
Settlement typology correction multiplier – Waste generation rate	HIC	RC_tP1	3	Beta-PERT (0.95, 1.08, 1.15)	Uniform (0.95, 1.15)
	UMC		3	Normal (0.62, 0.21)	Uniform (0.41, 0.83)
	LMC / LIC		3	Normal (0.47, 0.25)	Uniform (0.22, 0.72)
Settlement typology correction multiplier – Plastic in MSW	HIC	RC_C0	4	Beta-PERT (0.9, 1.0, 1.0)	Uniform (0.7, 1.1)
	UMC		3	Normal (0.73, 0.36)	Uniform (0.37, 1.09)
	LMC / LIC		3	Normal (0.69, 0.38)	Uniform (0.31, 1.07)
Settlement typology correction multiplier – Collection coverage	HIC	RC_tC1	3	Beta-PERT (1.0, 1.0, 1.0)	Uniform (0.99, 1.0)
	UMC		3	Beta-PERT (0.43, 0.53, 0.63)	Uniform (0.43, 0.63)
	LMC		3	Beta-PERT (0.36, 0.46, 0.56)	Uniform (0.36, 0.56)
	LIC		3	Beta-PERT (0.44, 0.54, 0.64)	Uniform (0.44, 0.64)
Settlement typology correction multiplier – Formal collection of dry recycling / other recovery	HIC	RC_tC2i, RC_tC2ii	4	Beta-PERT (0.9, 1.0, 1.0)	Uniform (0.7, 1.0)
	UMC		4	Beta-PERT (0.4, 0.5, 0.6)	Uniform (0.2, 0.8)
	LMC / LIC		4	Beta-PERT (0, 0, 0)	Uniform (0, 0.2)
Settlement typology correction multiplier – Incineration	HIC	RC_tC2iii	4	Beta-PERT (0.9, 1.0, 1.0)	Uniform (0.7, 1.0)
	LMIC		4	Beta-PERT (0, 0, 0)	Uniform (0, 0.2)
Settlement typology correction multiplier – Disposal	HIC	RC_tC3	4	Beta-PERT (1.0, 1.0, 1.0)	Uniform (0.9, 1.0)
	UMC		4	Beta-PERT (0.9, 1.0, 1.0)	Uniform (0.7, 1.0)
	LMC / LIC		4	Beta-PERT (0, 0, 0)	Uniform (0, 0.2)

Table S41. Plastic emissions (debris, open burned and total) for the baseline result compared to those of the counterfactual sensitivity analysis from **Section S.10.2**.

Aggregation level	Output	Baseline results (Mt·y^{-1})			Counterfactual results (Mt·y^{-1})			Absolute difference (Mt·y^{-1})			Percentage difference (%)		
		5th percentile	Mean	95th percentile	5th percentile	Mean	95th percentile	5th percentile	Mean	95th percentile	5th percentile	Mean	95th percentile
World	Plastic debris emissions	20.6	22.2	24.0	22.4	24.1	26.0	1.8	1.9	2.0	8.9%	8.6%	8.2%
	Plastic open burned emissions	27.6	29.9	32.4	40.4	43.9	47.9	12.8	14.0	15.5	46.2%	46.8%	47.7%
	Plastic emissions	48.3	52.1	56.3	62.9	68.0	73.7	14.6	15.9	17.3	30.2%	30.5%	30.8%
HIC	Plastic debris emissions	0.13	0.15	0.17	0.5	0.6	0.7	0.4	0.4	0.5	289.2%	288.5%	288.4%
	Plastic open burned emissions	0.01	0.01	0.02	0.2	0.3	0.3	0.2	0.3	0.3	2,688.5%	2,217.1%	1,846.4%
	Plastic emissions	0.1	0.2	0.2	0.7	0.9	1.0	0.6	0.7	0.8	429.8%	434.3%	437.6%
UMC	Plastic debris emissions	3.6	4.1	4.5	4.3	4.8	5.3	0.6	0.7	0.8	16.8%	16.7%	17.0%
	Plastic open burned emissions	9.0	10.0	11.0	12.6	13.9	15.3	3.6	3.9	4.3	39.6	39.4%	39.0%
	Plastic emissions	12.7	14.0	15.5	16.9	18.7	20.5	4.2	4.6	5.0	33.2%	32.8%	32.2%
LMC	Plastic debris emissions	11.7	13.1	14.7	12.4	13.9	15.6	0.7	0.8	0.8	5.9%	5.8%	5.8%
	Plastic open burned emissions	15.3	17.3	19.6	23.1	26.3	30.0	7.8	9.0	10.4	50.9%	52.0%	52.9%
	Plastic emissions	27.0	30.4	34.3	35.5	40.1	45.5	8.5	9.7	11.2	31.6%	32.0%	32.6%
LIC	Plastic debris emissions	4.3	4.9	5.4	4.4	4.9	5.4	0.0	0.0	0.0	1.0%	0.7%	0.3%
	Plastic open burned emissions	2.3	2.6	2.9	3.1	3.5	3.8	0.7	0.8	0.9	31.9%	31.4%	31.2%
	Plastic emissions	6.7	7.5	8.3	7.5	8.3	9.2	0.8	0.9	0.9	12.0%	11.5%	11.1%

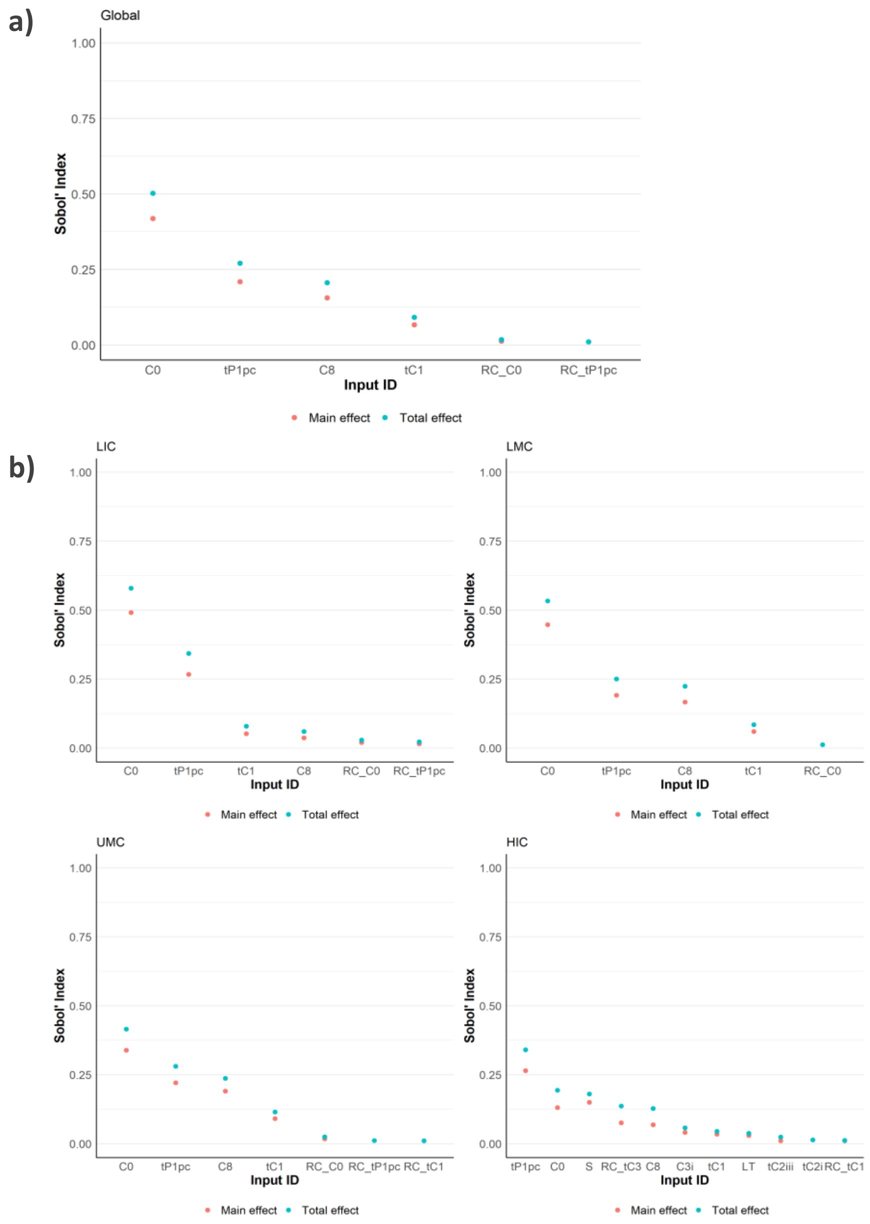

Fig. S30. Main effect and total effect Sobol' indices > 0.01 for total plastic emissions aggregated a) globally and b) by income-categories. Aggregation involved calculating the mean value weighted by plastic emission of all municipalities within that aggregation. Abbreviations: C0 = plastic in MSW (% of generated MSW), tP1pc = Waste generation rate per capita (kg·cap^{-1}·d^{-1}), tC1 = MSW collection coverage (% of generated MSW), C8 = open burning of uncontrolled disposal (% of uncontrolled disposal), RC_C0 = Settlement typology correction multiplier for plastic in MSW, RC_tP1$_{pc}$ = Settlement typology correction multiplier for waste generation rate, RC_tC1 = Settlement typology correction multiplier for collection coverage, S = street sweeping efficiency (%), C3i = emissions from the collection system prior to street sweepings (% of collected waste), LT = littering rate (% of MSW generation), RC_tC3 = Settlement typology correction multiplier for controlled disposal, tC2iii = Incineration (% of collected MSW), tC2i = collected for formal dry recycling (% of collected MSW).

S.11 Conversion of emission mass to item count

Assuming an average plastic item mass of 5-10 g, 52.1 $Mt \cdot y^{-1}$ is equivalent to 5.2-10.4 trillion plastic items released as debris or through open burning every year. Based on a global population of 7.8 billion people, the same mass would be approximately 2-4 plastic items emitted per person per day (note: a large proportion of emissions take place after collection, for example, by open burning at dumpsites).

Supplementary references

1 GADM. GADM database of global administrative areas. https://gadm.org/ (2012).

2 Jambeck, J. R. *et al.* Plastic waste inputs from land into the ocean. *Science* **347**, 768 (2015).

3 Lebreton, L. C. M. *et al.* River plastic emissions to the world's oceans. *Nat. Comm.* **8**, 15611 (2017).

4 Schmidt, C., Krauth, T. & Wagner, S. Export of Plastic Debris by Rivers into the Sea. *Environ. Sci. Technol.* **51**, 12246-12253 (2017).

5 Lau, W. W. Y. *et al.* Evaluating scenarios toward zero plastic pollution. *Science* **369**, 1455-1461 (2020).

6 UN-Habitat. Waste Wise Cities Tool: Step by Step Guide to Assess a City's Municipal Solid Waste Management Performance through SDG indicator 11.6.1 Monitoring. https://unhabitat.org/wwc-tool (2021).

7 Velis, C. A. Global recycling markets: plastic waste: A story for one player – China. http://wedocs.unep.org/handle/20.500.11822/19316 (International Solid Waste Association, Vienna, Austria, 2014).

8 Cook, E. & Velis, C. Plastic waste exports and recycling: Myths, misunderstandings and inconvenient truths. *Waste Manage. Res.* **40**, 1459-1461 (2022).

9 Wen, Z., Xie, Y., Chen, M. & Dinga, C. D. China's plastic import ban increases prospects of environmental impact mitigation of plastic waste trade flow worldwide. *Nat. Comm.* **12**, 425 (2021).

10 Secretariat of the Basel Convention. BC-14/12: Amendments to Annexes II, VIII and IX to the Basel Convention. http://www.basel.int/Portals/4/download.aspx?d=UNEP-CHW-COP.14-BC-14-12.English.pdf (Châtelaine, Switzerland, 2021).

11 European Commission. Commission Delegated Regulation (EU) 2020/2174 of 19 October 2020 amending Annexes IC, III, IIIA, IV, V, VII and VIII to Regulation (EC) No 1013/2006 of the European Parliament and of the Council on shipments of waste (Text with EEA relevance). http://data.europa.eu/eli/reg_del/2020/2174/oj (Official Journal of the European Union, 2020).

12 Brown, A., Laubinger, F. & Börkey, P. Monitoring trade in plastic waste and scrap: Environment Working Paper No. 210. https://dx.doi.org/10.1787/39058031-en (OECD, Paris, France, 2023).

13 United Nations. UN Comtrade Database: 3915-Waste, parings and scrap, of plastics 2022. https://comtradeplus.un.org/ (2024).

14 Iliff, C. Plastic waste and the Basel Convention: Investigation into the impact of the January 2021 amendments to annexes II, VIII and IX. University of Leeds (2023).

15 Verschoor, A. Towards a definition of microplastics: Considerations for the specification of physico-chemical properties. Report No. RIVM Letter report 2015-0116, https://www.rivm.nl/bibliotheek/rapporten/2015-0116.pdf (National Institute for Public Health and the Environment, Bilthoven, The Netherlands, 2015).

16 Velis, C. A., Cook, E. & Cottom, J. Waste management needs a data revolution – Is plastic pollution an opportunity? *Waste Manage. Res.* **39**, 1113-1115 (2021).

17 Wilson, D. C. *et al.* Global waste management outlook. Report No. 9280734792, https://wedocs.unep.org/bitstream/handle/20.500.11822/9672/-Global_Waste_Management_Outlook-2015Global_Waste_Management_Outlook.pdf.pdf?sequence=3&amp%3BisAllowed= (United Nations Environment Programme, Nairobi, Kenya, 2015).

18 Velis, C. Waste pickers in Global South: Informal recycling sector in a circular economy era. *Waste Manage. Res.* **35**, 329-331 (2017).

19 Adedara, M. L., Taiwo, R. & Bork, H.-R. Municipal solid waste collection and coverage rates in sub-Saharan african countries: A comprehensive systematic review and meta-analysis. *Waste* **1**, 389-413 (2023).

20 Wasserman, M., Anshassi, M. & Townsend Timothy, G. Assessing sample number requirements for municipal solid waste composition studies. *J. Hazard. Toxic Radioact. Waste* **26**, 04021038 (2022).

21 Chaudhary, P. *et al.* Underreporting and open burning – the two largest challenges for sustainable waste management in India. *Resour. Conserv. Recycl.* **175**, 105865 (2021).

22 Chen, D. M.-C., Bodirsky, B. L., Krueger, T., Mishra, A. & Popp, A. The world's growing municipal solid waste: trends and impacts. *Environ. Res. Lett.* **15**, 074021 (2020).

23 Sharma, G. *et al.* Gridded Emissions of CO, NOx, SO2, CO2, NH3, HCl, CH4, PM2.5, PM10, BC, and NMVOC from Open Municipal Waste Burning in India. *Environ. Sci. Technol.* **53**, 4765-4774 (2019).

24 Kawai, K. & Tasaki, T. Revisiting estimates of municipal solid waste generation per capita and their reliability. *J. Mater. Cycles Waste Manage.* **18**, 1-13 (2016).

25 Brunner, P. H. & Rechberger, H. *Practical handbook of material flow analysis: For environmental, resource and waste engineers*. Second edn, (CRC Press, Boca Raton, USA, 2017).

26 Cook, E. & Velis, C. Global review on safer end of engineered life. https://doi.org/10.5518/100/58 (Royal Academy of Engineering, London, UK, 2020).

27 Linzner, R. & Lange, U. Role and size of informal sector in waste management – a review. *Proceedings of the Institution of Civil Engineers - Waste and Resource Management* **166**, 69-83 (2013).

28 Charles, D. & Kimman, L. Plastic waste makers index 2023. https://cdn.minderoo.org/content/uploads/2023/02/04205527/Plastic-Waste-Makers-Index-2023.pdf (2023).

29 Schiavina, M., Freire, S. & MacManus, K., GHS population grid multitemporal (1975, 1990, 2000, 2015) R2019A. European Commission Joint Research Centre (JRC) http://doi.org/10.2905/42E8BE89-54FF-464E-BE7B-BF9E64DA5218 (2019).

30 UN-Habitat. Wastewise Cities (WaCT) Data Portal. https://unh.rwm.global/ (2022).

31 Kaza, S., Yao, L., Bhada-Tata, P. & Van Woerden, F. What a waste 2.0: a global snapshot of solid waste management to 2050. https://openknowledge.worldbank.org/bitstream/handle/10986/30317/9781464813290.pdf?sequence=12&isAllowed=y (World Bank Publications, Washington, DC, 2018).

32 Wasteaware. Wasteaware Benchmark Indicators. http://wabi.wasteaware.org/ (2022).

33 United Nations Statistics Division (UNSD). UNSD Environmental Indicators - waste. https://unstats.un.org/unsd/envstats/qindicators (New York, 2020).

34 Ministry of Housing and Urban-Rural Development (MoHURD), 2019 Urban Construction Statistical Yearbook. https://web.archive.org/web/20231203200401/https://www.mohurd.gov.cn/file/old/2020/20201231/w02020123122485271423125000.xls (2019).

35 SIPSN, National Waste Management Information System (Sistem Informasi Pengelolaan Sampah Nasional). https://sipsn.menlhk.go.id/sipsn/public/home (2022).

36 Dlamini, W. M. *et al.* National inventory on open burning practices and unintentional persistent organic pollutants (UPOPS) releases. https://stopopenburning.unitar.org/site/assets/files/1089/eswatini-_inventory_report_for_open_burning_project-_oct2017.pdf (Mbabane, Swaziland, 2017).

37 Wills, P. Composition analysis of litter waste in Wales. https://gov.wales/sites/default/files/publications/2020-01/composition-analysis-of-litter-waste-in-wales.pdf (Welsh Government, Bristol, UK, 2019).

38 Department for Environment Food and Rural Affairs (Defra). Statistics on waste managed by local authorities in England in 2019/20. https://www.gov.uk/government/statistics/local-authority-collected-waste-management-annual-results (UK, 2021).

39 Wilson, D. C. *et al.* 'Wasteaware' benchmark indicators for integrated sustainable waste management in cities. *Waste Manage.* **35**, 329-342 (2015).

40 United Nations Statistics Division (UNSD). Questionnaire 2020 on Environmental Statistics - Waste. https://unstats.un.org/unsd/envstats/questionnaire (United Nations Statistics Division (UNSD), New York, 2020).

41 Velis, C. A., Wilson, D. C., Gavish, Y., Grimes, S. M. & Whiteman, A. Socio-economic development drives solid waste management performance in cities: A global analysis using machine learning. *Sci. Total Environ.* **872**, 161913 (2023).

42 Ciesin Columbia University & Center for International Earth Science Information Network, Gridded Population of the World, Version 4 (GPWv4): Population Count Adjusted to Match 2010, 2015, 2020 Revisions of UN WPP Country Totals, Revision 11. https://doi.org/10.7927/H4PN93PB (2018).

43 Qu, W. & Li, R. Translation of Personal and Place Names from and into Chinese in Modern China: A Lexicographical History Perspective. *International Journal for the Semiotics of Law - Revue internationale de Sémiotique juridique* **28**, 525-557 (2015).

44 Chen, J., Kan, K. & Davis, D. S. Administrative reclassification and neighborhood governance in urbanizing China. *Cities.* **118**, 103386 (2021).

45 Google. Google hybrid map. https://www.google.com/maps (2021).

46 Hogg, D. *et al.* Study on Waste Statistics – A comprehensive review of gaps and weaknesses and key priority areas for improvement in the EU waste statistics. https://www.eunomia.co.uk/reports-tools/study-on-waste-statistics-a-comprehensive-review-of-gaps-and-weaknesses-and-key-priority-areas-for-improvement-in-the-eu-waste-statistics/ (Eunomia Research & Consulting, ENT Environment and Management, & Ekokonsultacijos, Bristol, UK, 2017).

47 UN-Habitat. *Solid waste management in the world's cities*. (UN-HABITAT, 2010).

48 Wilson, D. C., Rodic, L., Scheinberg, A., Velis, C. A. & Alabaster, G. Comparative analysis of solid waste management in 20 cities. *Waste Manage. Res.* **30**, 237-254 (2012).

49 Azevedo, B. D., Scavarda, L. F., Caiado, R. G. G. & Fuss, M. Improving urban household solid waste management in developing countries based on the German experience. *Waste Manage.* **120**, 772-783 (2021).

50 Abdulredha, M., Kot, P., Al Khaddar, R., Jordan, D. & Abdulridha, A. Investigating municipal solid waste management system performance during the Arba'een event in the city of Kerbala, Iraq. *Environ. Dev. Sus.* **22**, 1431-1454 (2020).

51 Ali, M. *et al.* Improvement of waste management practices in a fast expanding sub-megacity in Pakistan, on the basis of qualitative and quantitative indicators. *Waste Manage.* **85**, 253-263 (2019).

52 Kabera, T. & Nishimwe, H., "Systems analysis of municipal solid waste management and recycling system in east Africa: benchmarking performance in Kigali city, Rwanda" in 2018 International Conference on Renewable Energy and Environment Engineering (REEE 2018) (E3S Web Conf, 2019), pp. 03004.

53 Sharma, A., Ganguly, R. & Gupta, A. K. in *Pollutants from Energy Sources: Characterization and Control* (eds Rashmi Avinash Agarwal, Avinash Kumar Agarwal, Tarun Gupta, & Nikhil Sharma) 253-268 (Springer Singapore, Singapore, 2019).

54 Kabera, T., Wilson, D. C. & Nishimwe, H. Benchmarking performance of solid waste management and recycling systems in East Africa: Comparing Kigali Rwanda with other major cities. *Waste Manage. Res.* **37**, 58-72 (2019).

55 Sharma, A., Ganguly, R. & Gupta, A. K. Matrix method for evaluation of existing solid waste management system in Himachal Pradesh, India. *J. Mater. Cycles Waste Manage.* **20**, 1813-1831 (2018).

56 Sharma, A., Ganguly, R. & Gupta, A. K. in *Proceedings of the 1st International Conference on Sustainable Waste Management through Design.* (eds Harvinder Singh, Prashant Garg, & Inderpreet Kaur) 343-352 (Springer International Publishing).

57 Sharma, D. & Ganguly, R. Evaluation of existing solid waste management practices for Solan city-India. *J. Solid Waste Technol. Manage.* **44**, 32-42 (2018).

58 Lupo, T. & Cusumano, M. Towards more equity concerning quality of Urban Waste Management services in the context of cities. *J Clean Prod* **171**, 1324-1341 (2018).

59 Abdulredha, M., al-Khaddar, R., Kot, P., Jordan, D. & Abdulridha, A. *Benchmarking of the Current Solid Waste Management System in Karbala, Iraq, Using Wasteaware Benchmark Indicators*.

60 Oduro-Appiah, K. *et al.* Assessment of the municipal solid waste management system in Accra, Ghana: A 'Wasteaware' benchmark indicator approach. *Waste Manage. Res.* **35**, 1149-1158 (2017).

61 Byamba, B. & Ishikawa, M. Municipal Solid Waste Management in Ulaanbaatar, Mongolia: Systems Analysis. **9**, 896 (2017).

62 Rana, R., Ganguly, R. & Gupta, A. K. Evaluation of solid waste management in satellite towns of Mohali and Panchkula-India. *J. Solid Waste Technol. Manage.* **43**, 280-294 (2017).

63 Whiteman, A. *et al.* Wasteaware Benchmark Indicators for Integrated Sustainable Waste Management in Chinese Cities. https://rwm.global/utilities/documents/wabi.pdf (Deutsche Gesellschaft für Internationale Zusammenarbeit (GIZ) GmbH, Beijing, PR China,, 2019).

64 New center for Integrated studies of Land & Environment (NILE), Zaki, T., Kafafi, A. G., Mina, M. B. & Abd El-Halim, A. E. H. M. Annual report for solid waste management in Egypt, 2013. http://cairoclimatetalks.net/sites/default/files/EN%20Annual%20Report%20on%20Waste%20in%20Egypt_2013.pdf (Ministry of State for Environmental Affairs, Cairo, Egypt, 2013).

65 Association Jeffares & Green (Pty) Ltd & RWA Resources & Waste Advisory Group. The Diversion Of Municipal Solid Waste Away From Landfills In 6 South African Municipalities: Waste Analysis and Composition Survey (Report). Deutsche Gesellschaft für Internationale Zusammenarbeit (GIZ) GmbH, 2016).

66 Gutierrez Galicia, F., Coria Paez, A. L. & Tejeida Padilla, R. A study and factor identification of municipal solid waste management in mexico city. *Sustainability* **11**, 6305 (2019).

67 Zaman, A. U. Measuring waste management performance using the 'Zero Waste Index': the case of Adelaide, Australia. *J Clean Prod* **66**, 407-419 (2014).

68 Waste Management World. Recycling Facility Opens in Varna Bulgaria. https://waste-management-world.com/artikel/recycling-facility-opens-in-varna-bulgaria/ (2011).

69 Al Sabbagh, M. K., Velis, C. A., Wilson, D. C. & Cheeseman, C. R. Resource management performance in Bahrain: a systematic analysis of municipal waste management, secondary material flows and organizational aspects. *Waste Manage. Res.* **30**, 813-824 (2012).

70 Fuss, M., Barros, R. T. V. & Poganietz, W. R. The role of a socio-integrated recycling system in implementing a circular economy – The case of Belo Horizonte, Brazil. *Waste Manage.* **121**, 215-225 (2021).

71 Capital., E. G. Vitoria-Gasteiz towards zero waste. https://ec.europa.eu/environment/europeangreencapital/vg-zero-waste/ (2013).

72 StatLine, municipal waste; quantities, 1993-2015. https://opendata.cbs.nl/#/CBS/en/dataset/7467eng/table?searchKeywords=municipal%20 waste (2016).

73 Department of the Environment. Northern Ireland local authority collected municipal waste management statistics: Annual report 2011/12. https://www.daera-ni.gov.uk/sites/default/files/publications/doe/lac-municipal-waste-2011-12.pdf (Analytical Services Branch Department of the Environment, Belfast, Northern Ireland, 2012).

74 European Commission. Capital factsheet on separate collection. Report No. 070201/ENV/2014/691401/SFRA/A2, https://www.municipalwasteeurope.eu/sites/default/files/EL%20Athens%20Capital%20factsheet.pdf (European Commission, 2014).

75 Agarwal, A., Singhmar, A., Kulshrestha, M. & Mittal, A. K. Municipal solid waste recycling and associated markets in Delhi, India. *Resour. Conserv. Recycl.* **44**, 73-90 (2005).

76 Unifeed. St Lucia Recycling. https://www.unmultimedia.org/tv/unifeed/asset/1153/1153089/ (2014).

77 Zero Waste SG. Singapore waste statistics 2011. http://www.zerowastesg.com/2012/03/27/singapore-waste-statistics-2011/ (2011).

78 Aprilia, A., Tezuka, T. & Spaargaren, G. in *Waste Management-An Integrated Vision* (ed Luis Fernando Marmolejo Rebellon) 71-100 (IntechOpen, 2012).

79 Sim, N. M., Wilson, D. C., Velis, C. A. & Smith, S. R. Waste management and recycling in the former Soviet Union: The City of Bishkek, Kyrgyz Republic (Kyrgyzstan). *Waste Manage. Res.* **31**, 106-125 (2013).

80 Masood, M., Barlow, C. Y. & Wilson, D. C. An assessment of the current municipal solid waste management system in Lahore, Pakistan. *Waste Manage. Res.* **32**, 834-847 (2014).

81 Zaman, A. U. & Lehmann, S. The zero waste index: a performance measurement tool for waste management systems in a 'zero waste city'. *J Clean Prod* **50**, 123-132 (2013).

82 Scheinberg, A. & Simpson, M. A tale of five cities: Using recycling frameworks to analyse inclusive recycling performance. *Waste Manage. Res.* **33**, 975-985 (2015).

83 Albrepe. Panorama of Solid Waste in Brazil 2020 (in Portuguese). https://abrelpe.org.br/panorama/ (Albrepe, 2020).

84 National Bureau of Statistics of China. Explanatory Notes on Main Statistical Indicators. http://www.stats.gov.cn/tjsj/ndsj/2017/indexeh.htm (2017).

85 Tukey, J. W. *Exploratory data analysis*. Vol. 2 (Reading, MA, 1977).

86 The World Bank. World Bank Country and Lending Groups. https://datahelpdesk.worldbank.org/knowledgebase/articles/906519-world-bank-country-and-lending-groups (2021).

87 Wilson, D. C., Araba, A. O., Chinwah, K. & Cheeseman, C. R. Building recycling rates through the informal sector. *Waste Manage.* **29**, 629-635 (2009).

88 Kodra, A. & Milios, L. Municipal waste management in Albania. https://www.eea.europa.eu/publications/managing-municipal-solid-waste/albania-municipal-waste-management (European Environment Agency (EEA), 2013).

89 Ruurd van Schaik, H. B. Business opportunities in waste management in Algeria. Netherlands Enterprise Agency, Prinses Beatrixlaan, 2018).

90 Pegels, A. *et al. How sustainable is recycling? Reconciling the social, ecological, and economic dimensions in Argentina*. (Discussion Paper, 2020).

91 BELTA. Belarus aims to achieve 90% recycling rate of solid municipal waste by 2035. https://eng.belta.by/ (2020).

92 Gunsilius, E. Role of the informal sector in solid waste management and enabling conditions for its integration: Experiences from GTZ. https://www.resource-recovery.net/sites/default/files/gunsilius_gtz_role_of_informal_sector_conditions_for_integration.pdf (2012).

93 Silva de Souza Lima, N. & Mancini, S. D. Integration of informal recycling sector in Brazil and the case of Sorocaba City. *Waste Manage. Res.* **35**, 721-729 (2017).

94 Bermudez, J. F., Montoya-Ruiz, A. M. & Saldarriaga, J. F. Assessment of the current situation of informal recyclers and recycling: case study Bogotá. *Sustainability* **11**, 6342 (2019).

95 Bergman, E. Municipal Solid Waste Management in Informal Settlements–A multiple-case study of challenges and possibilities in the favelas and informal sector of Rio de Janeiro city. Report No. LUTFD2/TFEM-19/5150--SE + (1-92), http://lup.lub.lu.se/student-papers/record/8998685 (Lund University, 2019).

96 Valenzuela-Levi, N. Waste Political Settlements in Colombia and Chile: Power, Inequality and Informality in Recycling. *Dev. Change.* **51**, 1098-1122 (2020).

97 Larochelle, L., Turner, M. & LaGiglia, M. Evaluation of NAMA opportunities in Colombia’s solid waste sector. Colombia Center for clean air policy, Washington, DC, 2012).

98 The Economist Intelligence Unit. Progress and Challenges for Inclusive Recycling: An Assessment of 12 Latin American and Caribbean Cities. EIU, New York, 2017).

99 RRS & Walmart. Pursuing Zero Waste In A Diverse Landscape. http://recycle.com/wp-content/uploads/2018/12/walmart-pursuing-zero-waste-in-a-diverse-landscape.pdf (2018).

100 Holland Circular Hotspot. Waste Management in the LATAM Region: Business Opportunities for the Netherlands in Waste/Circular Economy sector in eight countries of Latin America., https://www.rvo.nl/sites/default/files/2021/02/Report_LATAM_Waste_Management_feb_2021.pdf (Netherlands Enterprise Agency, 2021).

101 Robayo Tapia, L. C. Propuesta para el manejo del reciclaje de desechos sólidos en el Distrito Metropolitano de Quito. PUCE (2016).

102 Mazariegos, C., Constantino, P. & Brolo, J. The power of grassroots solutions in the waste recovery chain. https://www.gt.undp.org/ (2021).

103 Koushki, B., Nasrabadi, T. & Amiri, M. J. Effective Factors in Municipal Solid Waste Minimization and Recovery by Making Use of Citizens' Participation; Case Study of a District in Tehran City. *Pollution* **6**, 367-375 (2020).

104 Ferrero, V. Recycle Beirut: give recycling a chance. https://medium.com/@vittoriaferrero/recycle-beirut-give-recycling-a-chance-14ee66b31d19 (2019).

105 Farah, J. *et al.* Solid Waste Management in Lebanon: Lessons for Decentralisation. (2019).

106 Republic of North Macedonia State Statistical Office. Municipal waste, 2020. https://www.stat.gov.mk/ (2021).

107 Razali, F., Weng Wai, C. & Daud, D. Z. A Review of Malaysia Solid Waste Management Policies to Improve Recycling Practice and Waste Separation Among Households. *Int. J. Built. Env. Sustain.* **6**, 39-45 (2019).

108 Bernache, G. The environmental impact of municipal waste management: the case of Guadalajara metro area. *Resour. Conserv. Recycl.* **39**, 223-237 (2003).

109 Fogarasi, S. *et al.* Dissolution of Base Metals from Waste Printed Circuit Boards. *Environ Eng Manag J* **14** (2015).

110 Almasi, A. M. Municipal waste management in Romania. https://www.eea.europa.eu/publications/managing-municipal-solid-waste/romania-municipal-waste-management (European Environment Agency (EEA), 2013).

111 Euronews. No time to waste? Moscow begins recycling its rubbish. https://www.euronews.com/2020/01/02/no-time-to-waste-moscow-begins-recycling-its-rubbish (2020).

112 Kilpeläinen, M. Evaluating alternative ways to promote recycling and circularity in St. Petersburg’s waste management. Degree Programme in Industrial Engineering and Management. Thesis, LUT University (2020).

113 Owen, C. A Tale of Two Recycling Initiatives: State, Society and Waste Management in St Petersburg and Shanghai. https://fpc.org.uk/a-tale-of-two-recycling-initiatives-state-society-and-waste-management-in-st-petersburg-and-shanghai/ (2019).

114 Government of Russian Federation, "Country Report (Draft) <Russian Federation>" in Eighth Regional 3R Forum in Asia and the Pacific “Achieving Clean Water, Clean Land and Clean Air through 3R and Resource Efficiency- A 21st Century Vision for Asia-pacific Communities” (2018).

115 letsrecycle. Recycling solutions in Serbia. https://www.letsrecycle.com/news/latest-news/recycling-solutions-in-serbia/ (2016).

116 Archer, D. & Trang, N. Closing the Loop. Innovative partnerships with informal workers to recover plastic waste, in an inclusive circular economy approach. https://www.unescap.org/sites/default/files/Closing%20The%20Loop_Regional%20Polic

y%20Guide.pdf (United Nations Economic and Social Commission for Asia and the Pacific (ESCAP), 2018).

117 Woodruff, A. Solid Waste Management in the Pacific: Tonga Country Snapshot. Report No. ARM146616-2, https://www.adb.org/publications/solid-waste-management-pacific-tonga-country-snapshot (Asian Development Bank, 2014).

118 Japan International Cooperation Agency, EX Research Institute Ltd. & Kokusai Kogyo Co., L. Data Collection Survey on Solid Waste Management in Turkey. https://openjicareport.jica.go.jp/pdf/12247094.pdf (Republic of Turkey, 2015).

119 Ramos, C., Vicentini, A. & Ortega, D. Challenges and opportunities of waste collection in Caracas: Sucre municipality case study. *Consilience*, 115-129 (2012).

120 Terzidis, K. Investigation into the current and potential future use of incineration as a form of waste treatment. MSc in Environmental Engineering and Project Management. Thesis, University of Leeds (2022).

121 Ma, W. *et al.* Air Pollutant Emission Inventory of Waste-to-Energy Plants in China and Prediction by the Artificial Neural Network Approach. *Environ. Sci. Technol.* (2022).

122 Kaza, S., Yao, L., Bhada-Tata, P., and Van Woerden, F., City level codebook. World Bank https://datacatalog.worldbank.org/dataset/what-waste-global-database (2018).

123 Djemaci, B. La gestion des déchets municipaux en Algérie: Analyse prospective et éléments d'efficacité. Université de Rouen (2012).

124 Bilal, A. & Abdelkader, O. The problem of Municipal solid waste management in Algeria. *Journal of the New Economy* **12**, 118-131 (2021).

125 Madani, S. Z. in *Sociétés urbaines et déchets: Éclairages internationaux* (eds C. Cirelli & B. Florin) 101-120 (Presses Universitaires François-Rabelais, 2015).

126 van Schaik, R. & Breukelman, H. Business opportunities in waste management in Algeria. https://www.rvo.nl/sites/default/files/2018/06/Business-opportunities-in-waste-management-in-Algeria.pdf (Ministry of Foreign Affairs, Netherlands, 2018).

127 Algerie Presse Service. Recycling and waste treatment fair: opening of the 2nd edition in Oran. https://www.aps.dz/economie/68042-salon-de-recyclage-et-traitement-des-dechets-ouverture-de-la-2e-edition-a-oran (2018).

128 Algerie Eco. Waste sorting in Oran: Plastic bottles for telephone credit tickets. https://www.algerie-eco.com/2020/11/12/tri-des-dechets-a-oran-des-bouteilles-en-plastique-contre-des-tickets-de-credit-telephonique/ (2020).

129 R20 Regions of Climate Action. Integrated Solid Waste Management Oran, Algeria. https://r20paris.org/wp-content/uploads/2016/06/oran-waste-overview301115.pdf (Geneva, Switzerland, 2015).

130 Maha, B. Gestion et Traitement des déchets à la wilaya de Constantine. Université des Frères Mentouri Constantine (2017).

131 Anisovich, N. Minsk And Its Garbage. An Online Map And Modular Sites With Containers Will Appear In The Capital [in Russian]. https://greenbelarus.info/articles/19-

05-2020/minsk-i-yago-smecce-anlayn-karta-i-modulnyya-plyacouki-z-kanteynerami-zyavyacca (2020).

132 Johansson, K. Municipal solid waste management in Minsk-current situation, future development and challenges. Thesis in Environmental Science. Thesis, Lund University (2020).

133 Belta. Collection of secondary raw materials in Belarus up by 3.2% in 2020. https://eng.belta.by/society/view/collection-of-secondary-raw-materials-in-belarus-up-by-32-in-2020-138678-2021/ (2021).

134 PwC Advisory spółka z ograniczoną odpowiedzialnością sp.k. The Green City Action Plan - Zenica: Bosnia and Herzegovina. https://www.ebrdgreencities.com/assets/Uploads/PDF/7018b505ef/Zenica-GCAP_Eng.pdf (2019).

135 Mmereki, D. Current status of waste management in Botswana: A mini-review. *Waste Manage. Res.* **36**, 555-576 (2018).

136 Gerdes, P. G., E. The Waste Experts: Enabling Conditions for Informal Sector Integration in Solid Waste Management: Lessons learned from Brazil, Egypt and India. https://www.giz.de/en/downloads/gtz2010-waste-experts-conditions-is-integration.pdf (GTZ, 2008).

137 Wollmann, C. Análisis de la gestión de los residuos sólidos en Brasil. Una comparativa entre las diez ciudades más grandes del país. Universitat Politècnica de Catalunya (2015).

138 Cidade De Sao Paulo. Cooperativas. http://www.capital.sp.gov.br/cidadao/rua-e-bairro/lixo/cooperativas (2019).

139 Pacheco, E. B. A. V., Ronchetti, L. M. & Masanet, E. An overview of plastic recycling in Rio de Janeiro. *Resour. Conserv. Recycl.* **60**, 140-146 (2012).

140 Cardosa, A. Porto Alegre, Rio Grande do Sul Brazil. https://globalrec.org/city/porto-alegre/ (nd).

141 Ahlheim, M., Becker, M., Trastl, H. & Losada, Y. A. Wasted! Resource recovery and waste management in Cuba. *Int. J. Cuban. Stud.* **11**, 147-173 (2019).

142 EMASEO EP. Quito a Reciclar. http://www.emaseo.gob.ec/gestion-ambiental/quitoareciclar/ (2020).

143 Zabala Celi, J. L. La industria del reciclaje en la ciudad de Quito, propuesta de modelo de negocio para la industria de reciclaje de plástico PET. Universidad Andina Simón Bolívar, Sede Ecuador (2018).

144 Latitud R. Programa “Quito a reciclar” inició en multifamiliares de Quitumbe. https://latitudr.org/programa-quito-a-reciclar-inicio-en-multifamiliares-de-quitumbe/ (2019).

145 Ayuntamiento de Cuenca. Horario del punto limpio. https://medioambiente.cuenca.es/punto-limpio?AspxAutoDetectCookieSupport=1 (2022).

146 Tehran Times. 25% of waste produced in Iran recyclable: environment official. https://www.tehrantimes.com/news/435128/25-of-waste-produced-in-Iran-recyclable-environment-official (2019).

147 Khayamabshi, E. Current Status of Waste Management in Iran and Business Opportunities. http://www.unido.or.jp/files/Iran-updated.pdf (2016).

148 Farzadkia, M., Jorfi, S., Akbari, H. & Ghasemi, M. Evaluation of dry solid waste recycling from municipal solid waste: case of Mashhad city, Iran. *Waste Manage. Res.* **30**, 106-112 (2012).

149 Islamic Republic News Agency. The completion of Isfahan waste complex requires 50 billion tomans of facilities [in Persian]. https://khabarban.com/a/28016140 (2019).

150 Republic of Kazakhstan Strategic planning and reform agency. Waste reuse and recycling. https://stat.gov.kz/search/item/ESTAT368109 (2022).

151 Republic of Kazakhstan of regulatory legal acts informal and legal systems. About the draft of the Decree of the President of the Republic of Kazakhstan "On the strategic plan for the sustainable development of the city of Astana until 2030". https://adilet.zan.kz/kaz/docs/P060000113_ (2006).

152 egov. Information on waste reduction, recycling and reuse. https://egov.kz/cms/kk/articles/ecology/waste_reduction_recycling_and_reuse (2022).

153 Saleh, E. Recycling Policies from the Bottom Up: Waste Work in Lebanon. https://www.arab-reform.net/pdf/?pid=16284&plang=en (2021).

154 Hamdan, H. Is Lebanon's new recycling project a bunch of garbage? https://www.al-monitor.com/originals/2018/05/lebanon-beirut-municipality-hariri-paper-project-garbage.html (2018).

155 Ministerio del Ambiente. Asociaciones de Recicladores autorizadas para iniciar operaciones*. https://cdn.www.gob.pe/uploads/document/file/1234953/Asociaciones_de_Recicladores_formalizados_y_con_plan_operando_en_Lima_y_Callao.08.20.pdf (2020).

156 Zárate, P. M. Planta de Reciclaje en Arequipa, primera especializada en plástico. https://elbuho.pe/2019/12/planta-de-reciclaje-en-arequipa-es-la-primera-a-nivel-nacional-especializada-en-plastico/ (2019).

157 Cruz, A. H. Evaluation of the recycling activity in Lima Norte [in Spanish]. *Revista del Instituto de investigación de la Facultad de minas, metalurgia y ciencias geográficas* **21**, 47-54 (2018).

158 La República. Cercado de Lima: instalarán 8 estaciones de reciclaje. https://larepublica.pe/sociedad/2019/09/21/cercado-de-lima-instalaran-8-estaciones-de-reciclaje-municipalidad-de-lima-plastico/ (2019).

159 Baloy, O. *et al.* National waste information baseline report. http://sawic.environment.gov.za/documents/1880.pdf (Department of Environmental Affairs, Pretoria, 2012).

160 Islamic Development Bank. Waste to Energy: Averting environnmental damage in Azerbaijan. https://www.isdb.org/sites/default/files/media/documents/2020-06/Success_Lflt_Azerbaijan_EN.pdf (Islamic Development Bank, Jeddah, Kingdom of Saudi Arabia, 2020).

161 Bir, R. S. Understanding the Effectiveness of the Current Waste Management System in Thimphu City, Bhutan. Master of Science in International Cooperation Policy (Master of Engineering in International Material Flow Management). Thesis, Ritsumeikan Asia Pacific University, (2015).

162 UNDP. Incinerator for Thimphu's bio-medical and hazardous wastes. https://www.undp.org/bhutan/stories/incinerator-thimphus-bio-medical-and-hazardous-wastes (2021).

163 Silva, L. J. d. V. B. d., Santos, I. F. S. d., Mensah, J. H. R., Gonçalves, A. T. T. & Barros, R. M. Incineration of municipal solid waste in Brazil: An analysis of the economically viable energy potential. *Renew. Energy* **149**, 1386-1394 (2020).

164 Song, Q., Li, J., Duan, H., Yu, D. & Wang, Z. Towards to sustainable energy-efficient city: A case study of Macau. *Renewable Sustainable Energy Rev* **75**, 504-514 (2017).

165 Zero Waste Europe. Big Victory: Under public pressure, waste incinerator was kicked out of the Spatial Plan of Zagreb! https://zerowasteeurope.eu/2017/10/big-victory-under-public-pressure-waste-incinerator-was-kicked-out-of-the-spatial-plan-of-zagreb/ (2017).

166 Bianchi, M., Merger, P. & Cordella, M. CIRCTER Spin-Off: Switzerland and Liechtenstein Case study. https://www.espon.eu/sites/default/files/attachments/CIRCTER%20SPINOFF%20-%20Switzerland%20and%20Liechtenstein.pdf (2021).

167 Bryne, T. International The Waste Management System of the Principality of Monaco. https://wasteadvantagemag.com/international-the-waste-management-system-of-the-principality-of-monaco/ (2015).

168 JFE Engineering Corporation. Waste to Energy Plant for Yangon City in Myanmar Final Report. https://www.env.go.jp/earth/coop/lowcarbon-asia/english/project/data/EN_MMR_2017_03.pdf (2018).

169 Turilova, K. *et al.* Municipal Solid Waste in Ukraine: Development Potential. (2019).

170 Wansi, B.-I. Zimbabwe: Waste-to-energy plant to be built in KweKwe. https://www.afrik21.africa/en/zimbabwe-waste-to-energy-plant-to-be-built-in-kwekwe/ (2022).

171 Badan Pusat Statistik (BPS). Indonesia Census 2020. https://www.bps.go.id/ (2020).

172 Premakumara, D. G. J., Abe, M. & Maeda, T., "Reducing municipal waste through promoting integrated sustainable waste management (ISWM) practices in Surabaya city, Indonesia" in Ecosystems and Sustainable Development VIII (WIT Press, 2011), pp. 457-468.

173 Shekdar, A. V. Sustainable solid waste management: an integrated approach for Asian countries. *Waste Manage.* **29**, 1438-1448 (2009).

174 Maalouf, A. & Mavropoulos, A. Re-assessing global municipal solid waste generation. *Waste Manage. Res.* (2022).

175 Rosecký, M. *et al.* Predictive modelling as a tool for effective municipal waste management policy at different territorial levels. *J. Environ. Manage.* **291**, 112584 (2021).

176 Dissanayaka, D. M. S. H. & Vasanthapriyan, S. Forecast Municipal Solid Waste Generation in Sri Lanka. *2019 International Conference on Advancements in Computing (ICAC)*, 210-215 (2019).

177 Ayeleru, O. O., Fajimi, L. I., Oboirien, B. O. & Olubambi, P. A. Forecasting municipal solid waste quantity using artificial neural network and supported vector machine techniques: A case study of Johannesburg, South Africa. *J Clean Prod* **289**, 125671 (2021).

178 Solano Meza, J. K., Orjuela Yepes, D., Rodrigo-Ilarri, J. & Cassiraga, E. Predictive analysis of urban waste generation for the city of Bogotá, Colombia, through the implementation of decision trees-based machine learning, support vector machines and artificial neural networks. *Heliyon* **5**, e02810 (2019).

179 Kumar, A., Samadder, S., Kumar, N. & Singh, C. Estimation of the generation rate of different types of plastic wastes and possible revenue recovery from informal recycling. *Waste Manage.* **79**, 781-790 (2018).

180 Yang, L. *et al.* Municipal Solid Waste Forecasting in China Based on Machine Learning Models. **9** (2021).

181 Kannangara, M., Dua, R., Ahmadi, L. & Bensebaa, F. Modeling and prediction of regional municipal solid waste generation and diversion in Canada using machine learning approaches. *Waste Manage.* **74**, 3-15 (2018).

182 Lebreton, L. & Andrady, A. Future scenarios of global plastic waste generation and disposal. **5**, 1-11 (2019).

183 Waste Atlas. Waste Atlas. http://www.atlas.d-waste.com/ (2022).

184 Schiavina, M., Freire, S. & MacManus, K., GHS-POP R2022A - GHS population grid multitemporal (1975-2030). Joint Research Centre (JRC) European Commission http://data.europa.eu/89h/d6d86a90-4351-4508-99c1-cb074b022c4a (2022).

185 Kummu, M., Taka, M. & Guillaume, J. H. A. Gridded global datasets for Gross Domestic Product and Human Development Index over 1990–2015. *Sci. Dat.* **5**, 180004 (2018).

186 Smits, J. & Permanyer, I. The Subnational Human Development Database. *Sci. Dat.* **6**, 190038 (2019).

187 The World Bank Group. GNI per capita, Atlas method (current US $). https://data.worldbank.org/indicator/NY.GNP.PCAP.CD (2015).

188 Tatem, A. J. WorldPop, open data for spatial demography. *Sci. Dat.* **4**, 170004 (2017).

189 Transparency International. Corruption Perception Index 2015. https://www.transparency.org/en/cpi/2015 (2015).

190 Social Progress Imperative. Social progress index 2015. https://www.socialprogress.org/ (2015).

191 The World Bank Group. International tourism, number of arrivals. https://data.worldbank.org/indicator/ST.INT.ARVL (2015).

192 Natural Earth. Populated places. https://www.naturalearthdata.com/downloads/10m-cultural-vectors/10m-populated-places/ (2009).

193 United Nations. (United Nations Department of Economic and Social Affairs Statistics Division, 2017).

194 Maffenini, L., Schiavina, M., Melchiorri, M., Pesaresi, M. & Kemper, T. GHS-DU-TUC user guide. Report No. JRC132762, Publications Office of the European Union, Luxembourg, 2023).

195 European Commission & Eurostat. *Applying the Degree of Urbanisation : A Methodological Manual to Define Cities, Towns and Rural Areas for International Comparisons : 2021 Edition*. (Publications Office of the European Union,, 2021).

196 Schiavina, M., Melchiorri, M. & Freire, S., GHS-DUC R2022A - GHS Degree of Urbanisation Classification, application of the Degree of Urbanisation methodology (stage II) to GADM 3.6 layer, multitemporal (1975-2030). European Commission Joint Research Centre (JRC) http://data.europa.eu/89h/f5224214-6b66-43dfa9c6-cc974f17d803 (2022).

197 Gregorutti, B., Michel, B. & Saint-Pierre, P. Correlation and variable importance in random forests. *Stat. Comp.* **27** (2017).

198 International Organization for Standardization. ISO 3166-1:2020 Country Codes. https://www.iso.org/standard/72482.html (International Organization for Standardization, 2020).

199 Breiman, L. Random Forests. *Machin. Learn.* **45**, 5-32 (2001).

200 Biau, G. & Scornet, E. A random forest guided tour. *TEST* **25**, 197-227 (2016).

201 Meinshausen, N. Quantile regression forests. *J. Machin. Learn. Res.* **7**, 983-999 (2006).

202 Francke, T., López-Tarazón, J. A. & Schröder, B. Estimation of suspended sediment concentration and yield using linear models, random forests and quantile regression forests. **22**, 4892-4904 (2008).

203 Tyralis, H., Papacharalampous, G. & Langousis, A. A Brief Review of Random Forests for Water Scientists and Practitioners and Their Recent History in Water Resources. **11**, 910 (2019).

204 Probst, P., Wright, M. N. & Boulesteix, A.-L. Hyperparameters and tuning strategies for random forest. **9**, e1301 (2019).

205 Hyndman, R. J. & Koehler, A. B. Another look at measures of forecast accuracy. *Int. J. Forecasting* **22**, 679-688 (2006).

206 Chruszcz, A. National municipal waste compositional analysis in Wales. http://www.wrapcymru.org.uk/sites/files/wrap/Wales%20Municipal%20Waste%20Composition%202015-16%20FINAL.pdf (WRAP Cymru, Cardiff, Wales, 2016).

207 Bridgwater, E., Fletcher, E., Scholes, R., Tomes, T. & Hedger, J. National household waste composition 2017. https://wrap.org.uk/sites/default/files/2021-10/WRAP-national-household-waste-comparison-2017.pdf (Waste and Resources Action Programme, Bristol, 2019).

208 Cascadia Consulting Group. 2014 disposal-facility-based characterization of solid waste in California. https://www2.calrecycle.ca.gov/Publications/Download/1301 (California Department of Resources Recycling and Recovery (CalRecycle), Sacramento, USA, 2015).

209 BMK. Inventory of waste management in Austria - status report 2021 [in German]. https://www.bmk.gv.at/dam/jcr:04ca87f4-fd7f-4f16-81ec-57fca79354a0/BAWP_Statusbericht2021.pdf (Federal Ministry for Climate Protection Environment Energy Mobility Innovation and Technology, Vienna, 2021).

210 Tetra Tech EBA Inc. 2015 Waste Composition monitoring program. Report No. 704-SWM.SWOP03013-01, http://www.metrovancouver.org/services/solid-waste/SolidWastePublications/2015_Waste_Composition_Report.pdf (Metro Vancouver, Vancouver, 2016).

211 Putri, A. R., Fujimori, T. & Takaoka, M. Plastic waste management in Jakarta, Indonesia: evaluation of material flow and recycling scheme. *J. Mater. Cycles Waste Manage.* **20**, 2140-2149 (2018).

212 Besen, G. R. & Fracalanza, A. P. Challenges for the Sustainable Management of Municipal Solid Waste in Brazil. *Disp* **52**, 45-52 (2016).

213 Sabedot, S. & Pereira Neto, T. Environmental performance of waste pickers in Esteio (RS) [in Portugees]. *Engenharia Sanitaria e Ambiental* **22**, 103-109 (2017).

214 Schenck, C. J., Blaauw, P. F., Swart, E. C., Viljoen, J. M. M. & Mudavanhu, N. The management of South Africa's landfills and waste pickers on them: Impacting lives and livelihoods. *Devel. Southern Africa* **36**, 80-98 (2019).

215 Velis, C. A. *et al.* An analytical framework and tool ('InteRa') for integrating the informal recycling sector in waste and resource management systems in developing countries. *Waste Manage. Res.* **30**, 43-66 (2012).

216 Masood, M. & Barlow, C. Y. Framework for integration of informal waste management sector with the formal sector in Pakistan. *Waste Manage. Res.* **31**, 93-105 (2013).

217 Chandramohan, A., Ravichandran, C. & Sivasankar, V. Solid waste, its health impairments and role of rag pickers in Tiruchirappalli city, Tamil Nadu, Southern India. **28**, 951-958 (2010).

218 Oteng-Ababio, M. The Role of yhe Informal Sector in Solid Waste Management in The Gama, Ghana: Challenges and Opportunities. *Tijdschrift voor economische en sociale geografie* **103**, 412-425 (2012).

219 Medina, M. Serving the unserved: informal refuse collection in Mexico. *Waste Manage. Res.* **23**, 390-397 (2005).

220 Gutberlet, J. & Baeder, A. M. Informal recycling and occupational health in Santo André, Brazil. *Int. J. Environ. Health Res.* **18**, 1-15 (2008).

221 Zolnikov, T. R., da Silva, R. C., Tuesta, A. A., Marques, C. P. & Cruvinel, V. R. N. Ineffective waste site closures in Brazil: A systematic review on continuing health conditions and occupational hazards of waste collectors. *Waste Manage.* **80**, 26-39 (2018).

222 Mrkajić, V., Stanisavljevic, N., Wang, X., Tomas, L. & Haro, P. Efficiency of packaging waste management in a European Union candidate country. *Resour. Conserv. Recycl.* **136**, 130-141 (2018).

223 Conke, L. S. Barriers to waste recycling development: Evidence from Brazil. *Resour. Conserv. Recycl.* **134**, 129-135 (2018).

224 Botello-Álvarez, J. E., Rivas-García, P., Fausto-Castro, L., Estrada-Baltazar, A. & Gomez-Gonzalez, R. Informal collection, recycling and export of valuable waste as transcendent factor in the municipal solid waste management: A Latin-American reality. *J Clean Prod* **182**, 485-495 (2018).

225 Navarrete-Hernandez, P. & Navarrete-Hernandez, N. Unleashing waste-pickers' potential: Supporting recycling cooperatives in Santiago de Chile. *World Devel.* **101**, 293-310 (2018).

226 Hartmann, C. Waste picker livelihoods and inclusive neoliberal municipal solid waste management policies: The case of the La Chureca garbage dump site in Managua, Nicaragua. *Waste Manage.* **71**, 565-577 (2018).

227 Abledu, E. S. & Amfo-Otu, R. Contribution of Informal Sector Recycling Workers to Sustainable Landfill Management : The Case of Kpone Landfill Site in the Greater Accra Region. *PUCG Appl. Res. J.* **4**, 1-11 (2018).

228 Vaidya, P., Kumar, R. & Sharma, D. Economics and Environmental Impacts of Plastic Waste Recycling: A Case Study of Mumbai. *J. Solid. Waste. Tech. Manage.* **42**, 287-297 (2016).

229 Kamran, A., Chaudhry, M. N. & Batool, S. A. Role of the Informal Sector in Recycling Waste in Eastern Lahore. *Pol. J. Environ. Stud.* **24**, 537-543 (2015).

230 Cunningham, R. N., Simpson, C. D. & Keifer, M. C. Hazards faced by informal recyclers in the squatter communities of Asunción, Paraguay. *Int. J. Occup. Environ. Health.* **18**, 181-187 (2012).

231 Bhaskar, A. & Chikarmane, P. The story of waste and its reclaimers: Organising waste collectors for better lives and livelihoods. *Indian Journal of Labour Economics* **55**, 595-620 (2012).

232 Asim, M., Batool, S. A. & Chaudhry, M. N. Scavengers and their role in the recycling of waste in Southwestern Lahore. *Resour. Conserv. Recycl.* **58**, 152-162 (2012).

233 Moniruzzaman, S. M., Bari, Q. H. & Fukuhara, T. Recycling practices of solid waste in Khulna City, Bangladesh. *J. Solid Waste Technol. Manage.* **37**, 1-15 (2011).

234 Nzeadibe, T. C. & Ajaero, C. K. in *Handbook of Environmental Policy* 243-262 (2011).

235 Scheinberg, A., Spies, S., Simpson, M. H. & Mol, A. P. J. Assessing urban recycling in low- and middle-income countries: Building on modernised mixtures. *Habitat. Int.* **35**, 188-198 (2011).

236 Sembiring, E. & Nitivattananon, V. Sustainable solid waste management toward an inclusive society: Integration of the informal sector. *Resour. Conserv. Recycl.* **54**, 802-809 (2010).

237 Hernández Romero, D. A. *et al.* Respiratory symptoms among waste-picking child laborers a cross-sectional study. *Int. J. Occup. Environ. Health.* **16**, 124-135 (2010).

238 Mitchell, C. L. Altered landscapes, altered livelihoods: The shifting experience of informal waste collecting during Hanoi's urban transition. *Geoforum* **39**, 2019-2029 (2008).

239 Zia, H., Devadas, V. & Shukla, S. Assessing informal waste recycling in Kanpur City, India. *Manage. Environ. Qual.* **19**, 597-612 (2008).

240 Medina, M. in *Membership Based Organizations of the Poor* (eds Martha Chen, Renana Jhabvala, Ravi Kanbur, & Carol Richards) Ch. 6, 125-141 (Routledge, London, UK, 2007).

241 Masocha, M. Informal waste harvesting in Victoria Falls town, Zimbabwe: Socio-economic benefits. *Habitat. Int.* **30**, 838-848 (2006).

242 Kumari, S. *et al.* Recovery of consumer waste in India – A mass flow analysis for paper, plastic and glass and the contribution of households and the informal sector. *Resour. Conserv. Recycl.* **101**, 167-181 (2015).

243 Matter, A., Ahsan, M., Marbach, M. & Zurbrügg, C. Impacts of policy and market incentives for solid waste recycling in Dhaka, Bangladesh. *Waste Manage.* **39**, 321-328 (2015).

244 King, M. F. & Gutberlet, J. Contribution of cooperative sector recycling to greenhouse gas emissions reduction: A case study of Ribeirão Pires, Brazil. *Waste Manage.* **33**, 2771-2780 (2013).

245 Ezeah, C., Fazakerley, J. A. & Roberts, C. L. Emerging trends in informal sector recycling in developing and transition countries. *Waste Manage.* **33**, 2509-2519 (2013).

246 Paul, J. G., Arce-Jaque, J., Ravena, N. & Villamor, S. P. Integration of the informal sector into municipal solid waste management in the Philippines - What does it need? *Waste Manage.* **32**, 2018-2028 (2012).

247 Hamidul Bari, Q., Mahbub Hassan, K. & Ehsanul Haque, M. Solid waste recycling in Rajshahi city of Bangladesh. *Waste Manage.* **32**, 2029-2036 (2012).

248 Steuer, B., Ramusch, R., Part, F. & Salhofer, S. Analysis of the value chain and network structure of informal waste recycling in Beijing, China. *Resour. Conserv. Recycl.* **117**, 137-150 (2017).

249 Linzner, R. & Salhofer, S. Municipal solid waste recycling and the significance of informal sector in urban China. *Waste Manage. Res.* **32**, 896-907 (2014).

250 Guo, S., Zhang, H., Minghui, Z. & Lin, A. Y. 京城十万拾荒族忐忑面 对被"收编 [The 100,000 scavengers in the capital are apprehensive about being "incorporated"]. http://www.mbtsg.com/periodical/9e7d9fc1baa9b966e4c452bb5b598c9c.html (2007).

251 Li, X. 拾荒者及其拾荒原因和途径分析 [Analysis of the reasons and ways of scavengers and their scavenging]. *Manage. Engin.*, 58-61 (2013).

252 Vose, D. *Risk Analysis: A Quantitative Guide*. 3rd Ed. edn, (John Wiley & Sons Ltd., Chichester, UK, 2008).

253 Wing Chau, K. The validity of the triangular distribution assumption in Monte Carlo simulation of construction costs: empirical evidence from Hong Kong. *Construct. Manage. Econ.* **13**, 15-21 (1995).

254 SYSTEMIQ & The Pew Charitable Trust. Breaking the Plastic Wave. https://www.pewtrusts.org/-/media/assets/2020/07/breakingtheplasticwave_report.pdf (The Pew Charitable Trust, UK, 2020).

255 Ahlers, J., Hemkhaus, M., Hibler, S. & Hannak, J. Analysis of extended producer responsibility schemes: Assessing the performance of selected schemes in European and EU countries with a focus on WEEE, waste packaging and waste batteries. https://erp-recycling.org/wp-content/uploads/2021/07/adelphi_study_Analysis_of_EPR_Schemes_July_2021.pdf (adelphi consult GmbH, Berlin, Germany, 2021).

256 Sasaki, S. & Araki, T. Estimating the possible range of recycling rates achieved by dump waste pickers: The case of Bantar Gebang in Indonesia. *Waste Manage. Res.* **32**, 474-481 (2014).

257 Vaccari, M., Torretta, V. & Collivignarelli, C. Effect of Improving Environmental Sustainability in Developing Countries by Upgrading Solid Waste Management Techniques: A Case Study. *Sustainability* **4**, 2852-2861 (2012).

258 Simatele, D. M., Dlamini, S. & Kubanza, N. S. From informality to formality: Perspectives on the challenges of integrating solid waste management into the urban development and planning policy in Johannesburg, South Africa. *Habitat. Int.* **63**, 122-130 (2017).

259 Jaligot, R., Wilson, D. C., Cheeseman, C. R., Shaker, B. & Stretz, J. Applying value chain analysis to informal sector recycling: A case study of the Zabaleen. *Resour. Conserv. Recycl.* **114**, 80-91 (2016).

260 Andrianisa, H. A., Brou, Y. O. K. & Séhi bi, A. Role and importance of informal collectors in the municipal waste pre-collection system in Abidjan, Côte d'Ivoire. *Habitat. Int.* **53**, 265-273 (2016).

261 Lino, F. & Ismail, K. Analysis of the potential of municipal solid waste in Brazil. *Environ Dev* **4**, 105-113 (2012).

262 Zhou, Y. & Xiong, H. 北京市垃圾拾荒者的资源贡献及其经济价值估测 [Resource Contribution of Waste Pickers in Beijing and Estimation of Economic Value]. *Ecol. Econom.*, 168-171 (2010).

263 Burneo, D., Cansino, J. M. & Yñiguez, R. Environmental and Socioeconomic Impacts of Urban Waste Recycling as Part of Circular Economy. The Case of Cuenca (Ecuador). *Sustainability* **12** (2020).

264 Cajamarca Cajamarca, E. S., Bueno Sagbaicela, W. R. & y Jimbo Días, J. S. De cero a dinero: La basura como fuente principal para un negocio inclusivo de reciclaje en Cuenca (Ecuador) [From zero to cash: Waste as a main source for an inclusive recycling business in Cuenca (Ecuador)]. *Retos Revista de Ciencias de la Administración y Economía* **9**, 71-87 (2019).

265 Ferronato, N., Preziosi, G., Gorritty Portillo, M. A., Guisbert Lizarazu, E. G. & Torretta, V. Assessment of municipal solid waste selective collection scenarios with geographic information systems in Bolivia. *Waste Manage.* **102**, 919-931 (2020).

266 Miranda, I. T., Fidelis, R., de Souza Fidelis, D. A., Pilatti, L. A. & Picinin, C. T. The Integration of Recycling Cooperatives in the Formal Management of Municipal Solid Waste as a Strategy for the Circular Economy—The Case of Londrina, Brazil. *Sustainability* **12** (2020).

267 Rutkowski, J. E. Inclusive packaging recycling systems: Improving sustainable waste management for a circular economy. *Detritus* **13**, 29-46 (2020).

268 Sasaki, S. *et al.* Recycling contributions of dumpsite waste pickers in Bantar Gebang, Indonesia. *J. Mater. Cycles Waste Manage.* **22**, 1662-1671 (2020).

269 Regional Initiative for Inclusive Recycling. Advances in recycling and in the inclusion of basic recyclers in Ecuador: Diagnosis in the cities of Quito, Guayaquil, Cuenca and Manta [in Spanish]. https://www.dropbox.com/s/duq17pmywqsitbl/Linea_Base_Recicladores_4_ciudades_Ecuador_web.pdf?dl=0 (Regional Initiative for Inclusive Recycling, 2015).

270 Ogwueleka, T. C. & BP, N. Activities of informal recycling sector in North-Central, Nigeria. *Energy. Nex.* **1**, 100003 (2021).

271 Sasaki, S., Watanabe, K., Widyaningsih, N. & Araki, T. Collecting and dealing of recyclables in a final disposal site and surrounding slum residence: the case of Bantar Gebang, Indonesia. *J. Mater. Cycles Waste Manage.* **0**, 0-0 (2018).

272 OECD.Stat, Global Plastics Outlook. https://stats.oecd.org/ (2022).

273 Dimitrakakis, E., Janz, A., Bilitewski, B. & Gidarakos, E. Small WEEE: Determining recyclables and hazardous substances in plastics. *J. Hazard. Mater.* **161**, 913-919 (2009).

274 Stenvall, E. Electronic Waste Plastics Characterisation and Recycling by Melt-processing. Licentiate of Engineering. Thesis, Chalmers University of Technology (2013).

275 Nonclercq, A. Mapping flexible packaging in a Circular Economy [F.I.A.C.E]. PDEng Chemical Product Design. Thesis, Delft University of Technology (2016).

276 Cimpan, C., Bjelle, E. L. & Strømman, A. H. Plastic packaging flows in Europe: A hybrid input-output approach. *J. Ind. Ecol.* **25**, 1572-1587 (2021).

277 Europur. Flexible polyurethane foam in mattresses and furniture: an overview of possible end of life solutions. https://elegant-williamson.46-242-128-94.plesk.page/wp-content/uploads/2022/03/factsheetPU_final.pdf (European Association of Flexible Polyurethane Foam Blocks Manufacturers, Brussels, Belgium, 2016).

278 Antonopoulos, I., Faraca, G. & Tonini, D. Recycling of post-consumer plastic packaging waste in the EU: Recovery rates, material flows, and barriers. *Waste Manage.* **126**, 694-705 (2021).

279 Roosen, M. *et al.* Detailed Analysis of the Composition of Selected Plastic Packaging Waste Products and Its Implications for Mechanical and Thermochemical Recycling. *Environ. Sci. Technol.* **54**, 13282-13293 (2020).

280 MBA Polymers UK Limited. Reliable, Innovative, Sustainable Recycled Plastics. https://www.mbapolymers.co.uk/gb/ (2022).

281 MGG Polymers GmbH. Sustainable plastics production. https://mgg-polymers.com/ (2022).

282 PRAKTIK system s.r.o. Electrical waste recycling. https://www.praktiksystem.cz/electrical-waste-recycling/ (2022).

283 Bage Plastics GmbH. Change perspective. Change the rules Think sustainable. https://bage-plastics.com/de/bage-plastics/ (2022).

284 Yoshida, A. *et al.* E-waste recycling processes in Indonesia, the Philippines, and Vietnam: A case study of cathode ray tube TVs and monitors. *Resour. Conserv. Recycl.* **106**, 48-58 (2016).

285 Haarman, A., Magalini, F. & Courtois, J. Study on the Impacts of Brominated Flame Retardants on the Recycling of WEEE plastics in Europe., https://www.bsef.com/wp-content/uploads/2020/11/Study-on-the-impact-of-Brominated-Flame-Retardants-BFRs-on-WEEE-plastics-recycling-by-Sofies-Nov-2020.pdf (Sofies, 2020).

286 Boudewijn, A. *et al.* Systematic Quantification of Waste Compositions: A Case Study for Waste of Electric and Electronic Equipment Plastics in the European Union. *Sustainability* **14**, 7054 (2022).

287 Butturi, M. A., Marinelli, S., Gamberini, R. & Rimini, B. Ecotoxicity of Plastics from Informal Waste Electric and Electronic Treatment and Recycling. *Toxics* **8**, 99 (2020).

288 Owusu-Sekyere, K., Batteiger, A., Afoblikame, R., Hafner, G. & Kranert, M. Assessing data in the informal e-waste sector: The Agbogbloshie Scrapyard. *Waste Manage.* **139**, 158-167 (2022).

289 Chakraborty, P. *et al.* Baseline investigation on plasticizers, bisphenol A, polycyclic aromatic hydrocarbons and heavy metals in the surface soil of the informal electronic waste recycling workshops and nearby open dumpsites in Indian metropolitan cities. *Environ. Pollut.* **248**, 1036-1045 (2019).

290 Chruszcz, A. & Reeve, S. Composition of plastic waste collected via kerbside: Results of a waste compositional analysis of plastics at MRFs and PRFs. https://wrap.org.uk/resources/report/composition-plastic-waste-collected-

kerbside#download-file (Waste and Resources Action Programme (WRAP), Banbury, 2018).

291 OECD. Global Plastics Outlook: Economic Drivers, Environmental Impacts and Policy Options. https://www.oecd-ilibrary.org/content/publication/de747aef-en (OECD Publishing, Paris, France, 2022).

292 Alencar, M. V. *et al.* How far are we from robust estimates of plastic litter leakage to the environment? *J. Environ. Manage.* **323**, 116195 (2022).

293 Hernandez, J. & Fitzgerald, C. Searching for uncollected litter with computer vision. *arXiv:2211.14743* (2022). https://ui.adsabs.harvard.edu/abs/2022arXiv221114743H.

294 Elliott, T. *et al.* Assessment of measures to reduce marine litter from single use plastics. https://ec.europa.eu/environment/pdf/waste/Study_sups.pdf (European Commission, Luxembourg, 2018).

295 Eurostat. Municipal waste statistics. https://ec.europa.eu/eurostat/statistics-explained/index.php?title=Municipal_waste_statistics (2021).

296 Walker, T., Wong, T. & Wootton, R. *Effectiveness of street sweeping for stormwater pollution control*. (CRC for Catchment Hydrology, 1999).

297 Department for Environment Food and Rural Affairs (Defra). Achieving improvements in street cleansing & related services. London, 2005).

298 Coffey, M. & Coad, A. *Collection of municipal solid waste in developing countries*. (United Nations Human Settlements Programme (UN-HABITAT), 2010).

299 Yadav, V. *et al.* Framework for quantifying environmental losses of plastics from landfills. *Resour. Conserv. Recycl.* **161**, 104914 (2020).

300 Samson, O., Oluwole, A. & Abimbola, S. On the physical composition of solid wastes in selected dumpsites of Ogbomosoland, South-Western Nigeria. *J. Wat. Res. Protect.* **3**, 661-666 (2011).

301 Waste Atlas. The World's 50 Biggest Dumpsites: 2014 Report. http://www.atlas.d-waste.com/Documents/Waste-Atlas-report-2014-webEdition.pdf (2014).

302 Velis, C. A. & Cook, E. Mismanagement of Plastic Waste through Open Burning with Emphasis on the Global South: A Systematic Review of Risks to Occupational and Public Health. *Environ. Sci. Technol.* **55**, 7186-7207 (2021).

303 Minnesota Population Center, Integrated Public Use Microdata Series International: Version 7.3. IPUMS https://international.ipums.org/international-action/extract_requests/download (2020).

304 Brazilian Institute of Geography and Statistics - IBGE. Demographic Census 2010: Characteristics of the population and the domiciles universe results. https://biblioteca.ibge.gov.br/index.php/biblioteca-catalogo?view=detalhes&id=793 (Brazilian Institute of Geography and Statistics - IBGE, Rio de Janeiro, Brazil, 2011).

305 Anguilla Statistics Department. Selected Housing and Household Indicators – Analytical Brief. https://unstats.un.org/UNSD/Demographic/sources/census/wphc/anguilla/AIA-2015-05-22.pdf (Government of Anguilla, Anguilla, 2015).

306 National institute of statistics and economic analysis. Main socio demographic and economic indicators of the borgou department (RGPH-4, 2013) [in French]. https://instad.bj/images/docs/insae-statistiques/demographiques/population/Principaux%20Indicateurs%20avec%20projections%20RGPH4/Principaux%20indicateurs%20socio%20d%C3%A9mographiques%20et%20%C3%A9conomiques%20RGPH-4.pdf (National institute of statistics and economic analysis (INSAE), Benin, 2016).

307 INE – National Statistics Institute, BOLIVIA: HOUSEHOLDS ACCORDING TO DEPARTMENT AND SOLID WASTE TREATMENT, 2011-2021. INE – National Statistics Institute https://www.ine.gob.bo/index.php/estadisticas-sociales/vivienda-y-servicios-basicos/encuestas-de-hogares-vivienda/ (2022).

308 Statistics Botswana. Botswana demographic survey report 2017. https://www.statsbots.org.bw/sites/default/files/publications/Botswana%20Demographic%20Survey%20Report%202017.pdf (Statistics Botswana, Gaborone, Botswana, 2018).

309 Ministry of Finance & Economic management. Census of population and dwellings. http://www.mfem.gov.ck/images/documents/Statistics_Docs/5.Census-Surveys/4.Census-Report/2011_Cook_Islands_Population_Census_Report.pdf (Government of Cook Islands, Rarotonga, Cook Islands, 2011).

310 National Institute of Statistics and Censuses (INEC), Costa Rica: Status indicators and access to basic housing services by canton. National Institute of Statistics and Censuses (INEC) https://admin.inec.cr/sites/default/files/media/repoblaccenso2011-09.xls_0_2.xlsx (2015).

311 National Office of Statistics and Information. Population and Housing Census Cuba 2012. http://www.onei.gob.cu/sites/default/files/informe_nacional_censo_0.pdf (National Office of Statistics and Information, Havana, Cuba, 2014).

312 National Office for National Statistics. National Household Survey: HOME-2015 - General Report [In spanish]. https://archivo.one.gob.do/Multimedia/Download?ObjId=29305 (National Office for National Statistics, Santo Domingo, Dominican Republic, 2016).

313 Ministry of Health and Population, El-Zanaty and Associates & ICF International. Demographic and Health Survey 2014. https://dhsprogram.com/pubs/pdf/FR302/FR302.pdf (Ministry of Health and Population & ICF International, Cairo, Egypt, 2015).

314 General Directorate of Statistics and Census (DIGESTYC). Household Survey 2019. https://www.transparencia.gob.sv/institutions/minec/documents/401354/download (Government of the Republic of El Salvador Ministry of Economy, General Directorate of Statistics and Censuses, Delgado, El Salvador, 2020).

315 Central Statistical Agency. Welfare monitoring survey 2015/16: indicators on Living Standard, Accessibility, Household Assets VOLUME II. https://catalog.ihsn.org//catalog/9223/download/92816 (Central Statistical Agency, Addis Ababa, 2016).

316 Fiji Bureau of Statistics. 2017 Fiji population and housing census. https://www.statsfiji.gov.fj/component/advlisting/?view=download&format=raw&fileId=5970 (Fiji Bureau of Statistics, Suva, Fiji, 2018).

317 The Gambia Bureau of Satistics (GBoS). Environment Statistics Compendium. https://www.gbosdata.org/downloads/environmental-statistics-61 (The Gambia Bureau of Satistics (GBoS), Serrekunda, The Gambia, 2020).

318 Ghana Statistical Service. Population & Housing Census 2010. (2012).

319 National Institute of Statistics Guatemala, General household characteristics. 2018 Census: Table B6.1 - Households by main form of garbage disposal, by department [in Spanish]. National Institute of Statistics Guatemala https://www.censopoblacion.gt/explo/TabB6.xlsx (2018).

320 Sub Directorate of Environmental Statistics. Environmental Care Behavior Indicators 2014. https://www.bps.go.id/publication/2015/12/23/2cdc2ef08c706d6f205c69fc/indikator-perilaku-peduli-lingkungan-hidup-2014.html (Central Bureau of Statistics, Jakarta, Indonesia, 2014).

321 Statistical Institute of Jamaica (SIJ), 2011 Census of Population and Housing - Jamaica. Statistical Institute of Jamaica (SIJ) https://statinja.gov.jm/Census/PopCensus/2011%20Census%20of%20Population%20and%20Housing%20k.pdf (2011).

322 National Statistics Office. 2020 Population and Housing General Report and Results. https://sdd.spc.int/digital_library/republic-kiribati-2020-population-and-housing-general-report-and-results (Ministry of Finance, Tarawa, Republic of Kiribati, 2021).

323 National Statistical Office. 2018 Malawi Population and Housing Census. http://www.nsomalawi.mw/index.php?option=com_content&view=article&id=226:2018-malawi-population-and-housing-census&catid=8:reports&Itemid=6 (National Statistical Office, Zomba, Malawi, 2020).

324 National Institute of Statistics and Geography (INEG). Environmental information compendium. https://www.inegi.org.mx/app/biblioteca/ficha.html?upc=702825189518 (National Institute of Statistics and Geography (INEG), Aguascalientes, Mexico, 2020).

325 Namibia Statistics Agency (NSA). Namibia Population and Housing Census 2011. https://nada.nsa.org.na/index.php/catalog/19/related-materials (Namibia Statistics Agency (NSA), Windhoek, Namibia, 2012).

326 National Institute of Development Information (INIDE). Housing report: Continuous household survey (ECH) 2019 - 2020 [In Spanish]. https://www.inide.gob.ni/docs/Ech/2020/INFORME_DE_CARACTERISTICAS_DE_LAS_VIVIENDAS2019_2020.pdf (National Institute of Development Information (INIDE), Managua, Nicaragua, 2021).

327 National Statistics Institute (INE). Paraguay Statistical Yearbook 2019. https://www.ine.gov.py/resumen/MTcz/anuario-estadistico-2019 (National Statistics Institute (INE), Fernando de la Mora, Paraguay, 2021).

328 Samoa Bureau of statistics. Samoa’s Experimental Solid Waste Accounts FY2013-14 to FY2015-16. https://www.sbs.gov.ws/digi/Samoa's%20Experimental%20Solid%20Waste%20Arrounts_2013-2014%20to%202015-2016.pdf (Samoa Bureau of statistics, Apia, Samoa, 2019).

329 Statistics Sierra Leone. Sierra Leone 2015 Population and Housing Census: National Analytical Report. https://www.statistics.sl/images/StatisticsSL/Documents/Census/2015/2015_census_national_analytical_report.pdf (Statistics Sierra Leone, Freetown, Sierra Leone, 2017).

330 Department of Census and Statistics. Census of Population and Housing 2012: Provisional information based on 5% sample. http://www.statistics.gov.lk/Population/StaticalInformation/CPH2011/Census2012ResultsPopulationHousingBased5Sample (Ministry of Finance and Planning, Colombo, Sri Lanka, 2012).

331 Central Statistics Office. 2010 Population and Housing Census Preliminary Report (Updated April 2011). https://catalog.ihsn.org//catalog/4328/download/56501 (Central Statistics Office, Castries, St Lucia, 2011).

332 National Bureau of Statistics. Housing Condition, Household Amenities and Assets Monograph: 2012 Population and Housing Census Volume IV. https://www.nbs.go.tz/index.php/en/census-surveys/population-and-housing-census?start=10 (National Bureau of Statistics, Zanzibar, Tanzania, 2015).

333 Tonga Statistics Department. Tonga 2016 Census of Population and Housing: Volume 1: Basic Tables and Administrative Report - Second Edition. https://sdd.spc.int/digital_library/tonga-2016-census-population-and-housing-volume-1-basic-tables-and-administrative (Tonga Statistics Department, Nuku’alofa, Tonga, 2018).

334 Uganda Bureau of Statistics. Uganda National Household Survey 2016/2017 Report. https://catalog.ihsn.org//catalog/9249/download/92938 (Uganda Bureau of Statistics, Kampala, Uganda, 2018).

335 Palestinian Central Bureau of Statistics. Population, Housing and Establishments Census 2017: Housing Report – Final Results - Palestine. Report No. 2444, https://www.pcbs.gov.ps/Downloads/book2444.pdf (Palestinian Central Bureau of Statistics, Ramallah, Palestine, 2019).

336 KANTAR. Burning in UK Homes and Gardens. Report No. PB 14644, https://randd.defra.gov.uk/ProjectDetails?ProjectID=20159&FromSearch=Y&Publisher=1&SearchText=AQ1017&SortString=ProjectCode&SortOrder=Asc&Paging=10#Description (Department for Environment Food and Rural Affairs., London, UK, 2020).

337 Copping, S., Quinn, C. & Gregory, R. Review and Investigation of deep-seated fires within landfill sites. Report No. SC010066, https://assets.publishing.service.gov.uk/government/uploads/system/uploads/attachment_data/file/291589/scho0307bmco-e-e.pdf (Environment Agency, Bristol, UK, 2007).

338 Bates, M. Managing landfill site fires in Northamptonshire. http://cfps.org.uk.surface3.vm.bytemark.co.uk/domains/cfps.org.uk/local/media/library/6

77.pdf (Environment and Transport Scrutiny Committee: Northamptonshire County Council, Northampton, 2004).

339 Dlamini, W. M. *et al.* National inventory on open burning practices and unintentional persistent organic pollutants (UPOPS) releases. https://stopopenburning.unitar.org/site/assets/files/1089/eswatini-_inventory_report_for_open_burning_project-_oct2017.pdf (Swaziland Environment Authority (SEA), Swaziland, 2017).

340 Wang, Y. *et al.* Atmospheric emissions of typical toxic heavy metals from open burning of municipal solid waste in China. *Atmos. Environ.* **152**, 6-15 (2017).

341 IPCC, R. IPCC guidelines for national greenhouse gas inventories. *Prepared by the national greenhouse gas inventories programme*, 10-11 (2006).

342 Wiedinmyer, C., Yokelson, R. J. & Gullett, B. K. Global Emissions of Trace Gases, Particulate Matter, and Hazardous Air Pollutants from Open Burning of Domestic Waste. *Environ. Sci. Technol.* **48**, 9523-9530 (2014).

343 National Environmental Engineering Research Institute. Air quality assessment, emissions inventory and source apportionment studies: Mumbai. http://mpcb.gov.in/ereports/pdf/Mumbai_report_cpcb.pdf (Mumbai, 2010).

344 Bihałowicz, J. S., Rogula-Kozłowska, W. & Krasuski, A. Contribution of landfill fires to air pollution – An assessment methodology. *Waste Manage.* **125**, 182-191 (2021).

345 Graedel, T. E. Material flow analysis from origin to evolution. *Environ. Sci. Technol.* **53**, 12188-12196 (2019).

346 Brunner, P. H. & Rechberger, H. *Practical Handbook of Material Flow Analysis: For Environmental, Resource, and Waste Engineers*. (CRC press, 2016).

347 Meylan, G., Reck, B. K., Rechberger, H., Graedel, T. E. & Schwab, O. Assessing the reliability of material flow analysis results: The cases of rhenium, gallium, and germanium in the United States economy. *Environ. Sci. Technol.* **51**, 11839-11847 (2017).

348 Tanzer, J. & Rechberger, H. Setting the common ground: A generic framework for material flow analysis of complex systems. *Recycling* **4**, 23 (2019).

349 Wang, Y. & Ma, H. W. Analysis of uncertainty in material flow analysis. *J Clean Prod* **170**, 1017-1028 (2018).

350 Gottschalk, F., Scholz, R. W. & Nowack, B. Probabilistic material flow modeling for assessing the environmental exposure to compounds: Methodology and an application to engineered nano-TiO2 particles. *Environ. Model. Software* **25**, 320-332 (2010).

351 Kawecki, D., Nowack, B. J. E. s. & technology. Polymer-specific modeling of the environmental emissions of seven commodity plastics as macro-and microplastics. **53**, 9664-9676 (2019).

352 Kawecki, D., Scheeder, P. R. W. & Nowack, B. Probabilistic Material Flow Analysis of Seven Commodity Plastics in Europe. *Environ. Sci. Technol.* **52**, 9874-9888 (2018).

353 Kawecki, D. & Nowack, B. A proxy-based approach to predict spatially resolved emissions of macro- and microplastic to the environment. *Sci. Total Environ.* **748**, 141137 (2020).

354 Bornhöft, N. A., Nowack, B. & Hilty, L. M. Representation, propagation, and interpretation of uncertain knowledge in dynamic probabilistic material flow models. *Environmental Modeling & Assessment* **26**, 709-721 (2021).

355 Sieber, R., Kawecki, D. & Nowack, B. Dynamic probabilistic material flow analysis of rubber release from tires into the environment. *Environ. Pollut.* **258**, 113573 (2020).

356 Kawecki, D., Goldberg, L. & Nowack, B. Material flow analysis of plastic in organic waste in Switzerland. *Soil. Use, Manage.* **37**, 277-288 (2021).

357 UNEP, "Resolution adopted by the United Nations Environment Assembly on 2 March 2022, UNEP/EA.5/Res.14" in United Nations Environment Assembly of the United Nations Environment Programme Fifth session (United Nations Environment Assembly of the United Nations Environment Programme,, 2022).

358 Kantai, T., Hengesbaugh, M., Hovden, K. & Pinto-Bazurco, J. F. Summary of the second meeting of the intergovernmental negotiating committee to develop an international legally binding instrument on plastic pollution: 29 May – 2 June 2023. *Earth. Neg. Bull.* **36**, 1-12 (2023).

359 Mihai, F.-C. *et al.* Plastic Pollution, Waste Management Issues, and Circular Economy Opportunities in Rural Communities. **14**, 20 (2022).

360 Hoornweg, D. & Bhada-Tata, P. WHAT A WASTE: A Global Review of Solid Waste Management. https://siteresources.worldbank.org/INTURBANDEVELOPMENT/Resources/336387-1334852610766/What_a_Waste2012_Final.pdf (World Bank Urban Development & Local Government Unit, Washington, DC, USA, 2012).

361 Karak, T., Bhagat, R. M. & Bhattacharyya, P. Municipal Solid Waste Generation, Composition, and Management: The World Scenario. *Crit. Rev. Environ. Sci. Technol.* **42**, 1509-1630 (2012).

362 Hidalgo, D., Corona, F. & Martín-Marroquín, J. in *Proceedings from 4th International Conference on Sustainable Solid Waste Management, Limassol.* 25.

363 GADM, GADM data (version 4.1). GADM https://gadm.org/data.html (2022).

364 Defra, Local authority collected waste statistics - local authority data. Department for Environment Food & Rural Affairs (Defra) https://assets.publishing.service.gov.uk/government/uploads/system/uploads/attachment_data/file/1144270/LA_and_Regional_Spreadsheet_202122.xlsx (2023).

365 Taghipour, H., Amjad, Z., Aslani, H., Armanfar, F. & Dehghanzadeh, R. Characterizing and quantifying solid waste of rural communities. *J. Mater. Cycles Waste Manage.* **18**, 790-797 (2016).

366 Rajpal, A., Kazmi, A. A. & Tyagi, V. K. Solid waste management in rural areas nearby river Ganga at Haridwar in Uttarakhand, India. *J. Appl. Nat. Sci.* **12**, 592-598 (2020).

367 Syafrudin, S., Masjhoer, J. M. & Maryono, M. Characterization and quantification of solid waste in rural regions. *Glob. J. Environ. Sci. Manag.* **9**, 337-352 (2023).

368 Asgari, A. R. *et al.* Solid waste characterization and management practices in rural communities, Tehran and Alborz (Iran). *J. Solid. Waste. Tech. Manage.* **45**, 111-118 (2019).

369 Elhamdouni, D., Arioua, A., Karaoui, I., Baaddi, A. & Ouhamchich, K. A. Household solid waste sustainable management in the Khenifra region, Morocco. *Arab. J. Geosci.* **12**, 744 (2019).

370 Edjabou, M. E., Møller, J. & Christensen, T. H. Solid waste characterization in Kétao, a rural town in Togo, West Africa. *Waste Manage. Res.* **30**, 745-749 (2012).

371 Rodrigo-Ilarri, J., Vargas-Terranova, C. A., Rodrigo-Clavero, M. E. & Bustos-Castro, P. A. Advances on the implementation of circular economy techniques in rural areas in Colombia under a sustainable development framework. *Sustainability* **13**, 3816 (2021).

372 Taboada-González, P., Aguilar-Virgen, Q., Ojeda-Benítez, S. & Armijo, C. Waste characterization and waste management perception in rural communities in mexico: A case study. *Environ. Eng. Manage. J.* **10**, 1751-1759 (2011).

373 Emara, K. Sustainable solid waste management in rural areas: A case study of Fayoum governorate, Egypt. *Energy. Nex.* **9**, 100168 (2023).

374 Bernardes, C. & Günther, W. M. R. Generation of domestic solid waste in rural areas: Case study of remote communities in the Brazilian Amazon. *Human Ecology* **42**, 617-623 (2014).

375 Ministry of Environment of The Republic of Moldova. National waste management strategy of The Republic of Moldova (2013-2027). https://serviciilocale.md/public/files/deseuri/2013_01_24_NATIONAL_WASTE_MANAGEMENT_STRATEGY_2013-27_ENG.pdf (Chisinau, Moldova, 2013).

376 Collaguazo, G., Badea, A., Stan, C. & Pásztai, Z. Household wastes characterization and seasonal variations in Bihor County, Romania. *Sci. Bullet. Ser. C - Electri. Eng. Comp. Sci.* **78**, 281-290 (2016).

377 Ciuta, S., Apostol, T. & Rusu, V. Urban and rural MSW stream characterization for separate collection improvement. *Sustainability* **7**, 916-931 (2015).

378 European Union. Council Directive 1999/31/EC of 26 April 1999 on the landfill of waste. https://eur-lex.europa.eu/LexUriServ/LexUriServ.do?uri=CELEX:31999L0031:EN:HTML (The Council of the European Union, 1999).

379 OECD. Muncipal waste, Generation and Treatment. https://stats.oecd.org/index.aspx?DataSetCode=MUNW (2022).

380 Eurostat. Treatment of waste by waste category, hazardousness and waste management operations. https://ec.europa.eu/eurostat/databrowser/view/env_wastrt/default/table?lang=en (2022).

381 Ding, Y. *et al.* A review of China's municipal solid waste (MSW) and comparison with international regions: Management and technologies in treatment and resource utilization. *J Clean Prod* **293**, 126144 (2021).

382 Lu, J.-W., Zhang, S., Hai, J. & Lei, M. Status and perspectives of municipal solid waste incineration in China: A comparison with developed regions. *Waste Manage.* **69**, 170-186 (2017).

383 Kovalenko, V. V. *et al.* in *IOP Conference Series: Earth and Environmental Science.*1 edn.

384 DBPR. Data from: A local-to-global emissions inventory of macroplastic pollution. INSERT DOI when created (Dryad, 2023).

385 Iman, R. L. & Conover, W. J. A distribution-free approach to inducing rank correlation among input variables. *Communications in Statistics - Simulation and Computation* **11**, 311-334 (1982).

386 Alvisi, S., Ansaloni, N. & Franchini, M. A Procedure for Spatial Aggregation of Synthetic Water Demand Time Series. *Procedia Engineering* **70**, 51-60 (2014).

387 Tolson Bryan, A., Maier Holger, R., Simpson Angus, R. & Lence Barbara, J. Genetic Algorithms for Reliability-Based Optimization of Water Distribution Systems. *Journal of Water Resources Planning and Management* **130**, 63-72 (2004).

388 Magini, R., Boniforti, M. A. & Guercio, R. Generating Scenarios of Cross-Correlated Demands for Modelling Water Distribution Networks. *Water* **11** (2019).

389 OECD.Stat. OECD.Stat: Regions and cities. https://stats.oecd.org/ (2023).

390 Saltelli, A., Tarantola, S., Campolongo, F. & Ratto, M. *Sensitivity Analysis in Practice: A Guide to Assessing Scientific Models*. (John Wiley & Sons, Ltd, Chichester, UK, 2004).

391 Sobol, I. M. Sensitivity estimates for nonlinear mathematical models. *Math. Model. Comp. Exper.* **4**, 407-414 (1993).
